# An adaptive, youth-centred co-design methodology: place-based co-design centring youth and community participation

**DOI:** 10.1186/s40900-025-00833-w

**Published:** 2026-01-24

**Authors:** Ediane Santana de Lima, Katie Potter, Cristina Preece, Nirandeep Rehill, George Davis, Sophie Bulmer, Kate Allen, Anna March, Tim Hobbs, Peter Fonagy

**Affiliations:** 1 Dartington Service Design Lab, Bristol, UK; 2 Dartington Service Design Lab, Stoke Gabriel, UK; 3 Dartington Service Design Lab, Hereford, UK; 4 UCLPartners and The Department of Primary Care and Population Health, London, UK; 5 Dartington Service Design Lab, Weston-Super-Mare, UK; 6 UCLPartners, London, UK; 7 University of Exeter, Exeter, UK; 8 CEO Dartington Service Design Lab and Visiting Professor at University College London, Totnes, UK; 9 Division of Psychology and Language Sciences at University College London (UCL), Child and Family Programme at the Menninger Department of Psychiatry and Behavioural Sciences at Baylor College of Medicine, London, UK

**Keywords:** Adolescent mental health, Co-design, Community-based participatory research, Group model building, Systems thinking, Systems mapping, Social determinants, Participatory design

## Abstract

**Background:**

Co-design approaches are increasingly used in efforts to address complex challenges, such as adolescent mental health. Benefits of these approaches are multifaceted and include strategies that better reflect local perspectives and needs, as well as the potential for improved outcomes for those involved. However, the literature has highlighted challenges and unintended consequences of co-design, such as issues with inclusivity and undue burden being placed on participants. This paper examines the methods used in the ‘Deeper Discovery and Co-design phase’ of Kailo, a place-based research and design initiative aimed at tackling the social determinants of children’s and young people’s (CYP) mental health in their local communities.

**Methods:**

Over approximately nine months, a co-design approach was used across two pilot sites. This involved more than 69 workshops and several ad hoc engagements with CYP and other community members. The goal was to explore structural challenges and develop strategies to address CYP mental health and wellbeing in their local context. The co-design work also aimed to bring together community knowledge and expertise, systems mapping and academic evidence.

**Results:**

Over 300 local CYP, community members, professionals and practitioners participated or contributed to Kailo’s Deeper Discovery phase across the two pilot sites, via group sessions, individual feedback or surveys. The study identified key barriers (e.g. logistical constraints, limitations in accessibility of processes and participation, safeguarding and participant wellbeing concerns) and enablers (e.g. flexible and accessible participation, inclusive and adaptive facilitation, and participation and influence) that shaped conditions for implementation. Moreover, it explored the requirements and tensions for methodological implementation whilst maintaining a flexible and adaptive approach, centring CYP needs and voices, and integrating different methods into the co-design process.

**Conclusions:**

The Kailo programme prioritised the contributions of CYP and their communities, aiming to foster inclusive and diverse engagement through a distinctive, adaptive, youth-centred approach. This paper outlines the co-design methodology used to support the development of locally relevant strategies addressing the social determinants of mental health. However, balancing flexibility and adaptability and centring CYP needs, voices and agency, whilst maintaining a focus on broader structural issues, remains a challenge.

**Supplementary Information:**

The online version contains supplementary material available at 10.1186/s40900-025-00833-w.

## Abstract (de)

Co-design brings people together to tackle issues that matter to them. It can lead to solutions that better reflect people’s needs, but there are also challenges, such as making sure the process is inclusive and not placing too much responsibility on participants without enough support. This paper looks at how Kailo, a project focused on children and young people’s (CYP) mental health in local communities, used co-design. The project worked in two areas to explore challenges and develop strategies for improving CYP mental health. This process combined a range of research methods and community knowledge. Over 300 young people, community members, and professionals took part through group sessions, feedback, and surveys. We found barriers to co-design (such as logistical constraints, accessibility issues, and safeguarding concerns) as well as enablers (such as flexible participation, supportive environments, and inclusive facilitation). Kailo puts young people and their communities at the centre, using a youth-focused approach to shape local mental health strategies. To make this approach work well, we needed to work closely with community members, allow timelines to change when required, have strong systems in place to keep people safe and allow their full participation, and create opportunities for people and organisations to get involved in different ways.

## Background

### Co-design

Co-design approaches are increasingly used to develop solutions and strategies that address complex challenges across public health and other sectors [1–4]. It has been defined as a participatory methodology that involves the active engagement of diverse groups of participants in collaboratively exploring and developing responses to a shared challenge or defined problem [5, 6]. These approaches centre the lived experiences of those directly affected by, or who benefit from, the services or scenarios being designed, improved, or reimagined [7–9]. Grounded in principles of inclusivity, participation, partnership-building and collaboration, co-design aims to bridge the conventional boundaries that exist between service providers and the people they aim to support [10–13]. Open and honest conversations, along with cycles of iteration, are used to ensure the initiatives and improvements co-designed are grounded in real-life experiences and challenges [14–16].

### Co-design and adolescent mental health

Adolescent mental health represents one such complex challenge, with many children and young people (CYP) experiencing mental health difficulties in the UK [17]. There is a growing recognition of the value of involving them actively in the development of interventions and strategies designed to support their mental health [18]. A recent systematic review from Chinsen et al. [19] reported a substantial increase in the application of co-design methods in adolescent mental health over the past five years. These methods have been applied across various contexts, including the development of school-based mental health interventions [20, 21], the design of digital platforms such as apps [13, 22], the improvement of healthcare services [23, 24] and the creation of social media awareness campaigns [25, 26]. This work and the wider literature have highlighted several benefits and challenges to this approach.

### Benefits and challenges of co-design

The benefits of co-design are multifaceted. It supports the development of processes and strategies that better reflect the wants and needs of the communities it aims to benefit, by encouraging buy-in, promoting a sense of ownership over what is being developed [7, 10, 27], and providing opportunities for individuals to explore shared experiences and help others who face similar difficulties [26, 28, 29, 30].

However, several challenges have been identified when implementing co-design processes in public health [1, 31]. These include:

- Concerns around study quality and reporting, with issues such as the inconsistent use of terminology, frameworks, and methods [19].
- The emotional burden of engaging individuals in co-design projects on sensitive issues (including the potential need for ongoing support and trauma-informed practices) [32].
- A mismatch between the skills needed to implement participatory approaches and the skills currently available in public health settings, along with differing assumptions and strategic priorities between key stakeholder groups. This can create barriers to collaboration and potentially hinder the effective adoption of community-led approaches [10, 33].
- Frequent low-quality processes with limited participation from CYP [19].
- Limited inclusivity and representation, which can reinforce marginalisation and requires navigation of power imbalances [34].
- The risk of overburdening participants with the responsibility of generating solutions, creating undue pressures [35].

These challenges can raise concerns about the efficacy of co-designed solutions, especially when they are not grounded in other existing academic evidence, are not empirically tested, or they may prioritise short-term over long-term outcomes [33]. Co-design processes and their outcomes are also often not evaluated [19] which can limit learning and improvement of processes, as well as the assessment of their outcomes [27, 28, 33]. A thorough evaluation can help identify and replicate approaches, processes and strategies that demonstrate positive impacts, thereby extending their benefits to broader contexts [36].

### Co-design involving CYP

CYP participation should extend beyond involvement in isolated activities to create conditions in which CYPs lived experiences, perspectives, and agency inform design decisions and enable shared decision-making, as described in the Lundy Model of Child Participation [37] and the Ladders of Participation [38, 39]. This requires facilitators to recognise that CYP often hold unequal power or are subject to tokenistic or even harmful processes in many systems [40], and therefore facilitation of co-design spaces needs to draw on safeguarding and trauma-informed principles to ensure the prioritisation of safety, trust and agency [41]. When these are aligned with youth-centred approaches that emphasise the importance of collaboration and reflection, co-design processes can provide CYP with both a platform for insight generation, but also a space for skills and capacity building [40, 42].

### Co-design process

Co-design approaches typically include the following phases: (i) discovery, i.e., scoping and understanding the problems; (ii) design; (iii) ideation; and (iv) prototyping [5]. McKercher [43] highlights common principles when using co-design, namely power sharing, relationship prioritisation, utilising participatory approaches, and capacity building. However, others have identified gaps in our understanding of co-design, specifically in relation to its definitions, constituent elements, and how these function in practice [5, 27, 44, 45]. Consequently, the meaning, application, timing and scope of co-design can differ across contexts and initiatives [6, 33, 46, 47]. Some authors have noted that there is often limited integration of existing evidence and call for co-design approaches to incorporate both lived experience and knowledge with academic evidence [33, 36, 48]. This would ensure that proposed designs acknowledge evidence-informed approaches [49–51], avoid ineffective or potentially harmful interventions and increase their potential to benefit the target population [8].

### Focus of this paper

This paper presents a distinctive, adaptive, youth-centred, co-design methodology developed and tested across two UK sites. The primary aim is to describe the methodology used during the first iteration of the research and design initiative called Kailo (detailed below). Kailo maintains the established benefits of co-design approaches for adolescent mental health while addressing several previously identified challenges and limitations. Although this iteration of the co-design process is focused on adolescent mental health, the learning is intended to be valuable beyond this context, particularly for those interested in the implementation of research and co-design to address issues with communities, rather than for them.

### Context for this co-design process

Kailo is a place-based research and design initiative aimed at addressing the social determinants of CYPs mental health in their local contexts [52]. The social determinants include a broad range of social, economic and environmental factors that impact CYPs lives, such as unemployment, poverty, inequality, discrimination, and relationships [53, 54]. Kailo aims to develop a framework which can be used to help local partnerships, CYP and communities to strengthen, align and co-design evidence-informed strategies, policies and practices that address the underlying drivers of CYPs mental health and wellbeing [55, 56].

The Kailo Framework was initially developed, tested and delivered in two pilot sites: Northern Devon, a rural/coastal region in the South-West of England, and the London Borough of Newham, a densely populated inner-city area. Further applications are underway in other urban contexts in England.

Drawing on approaches that recognise the complexity and requirement for different levels of participation [37, 38, 39, 57], the Kailo framework adopts a nuanced approach that aims to progressively transfer decision-making authority to community members, employing a strategically layered implementation methodology. The phases are broadly informed by participatory design traditions and the structure of iterative phases for strategic design developed by UK Design Council’s Double Diamond framework [58]. These include phases of problem discovery and exploration, and the subsequent development of solutions around them. As illustrated in Table 1, Kailo has three phases [52].

**Table 1:** The Kailo Framework process (to date)

Phase	Aim and Purpose	Proposed Phase Outputs
Early Discovery: Exploratory phase and priority setting	To develop and strengthen relationships across the local area that enable shared priority areas to emerge.	• Identifying and establishing relationships, drawing on local insights and existing research to surface key local drivers of CYPs mental health. • Identifying alignment between CYP and the individuals and organisations that can fund and sustain change.
Deeper Discovery and Co-Design: Strategies and interventions design *(Focus of this manuscript)*	To better understand and design a youth-centred response to the priority areas that have buy-in and support from CYP, community and public service partners.	• Understanding the prioritised area(s) in more depth to enable the codesign of local policy, practice or infrastructure with CYP. • Development of a design response that is supported by funders and delivery organisations as well as CYP. • Planned delivery team identified and incorporated.
Implementation: Delivery, testing and learning phase	To embed co-designed policy or practice interventions into the local community and public service infrastructure and iteratively test and refine.	• Handover of the process from the co-design groups to a delivery team who will implement what is designed, and seek to improve, embed and sustain the designed output within the system. • Support this team through refinement, learning and resource acquisition.

Kailo’s *Early Discovery phase* in the initial two sites was conducted between May 2022 and May 2023 [56]. It involved qualitative engagements with over 500 individuals, including CYP and community professionals. Researchers and local partners collaborated with CYP and community professionals to prioritise areas of focus for the Deeper Discovery and Co-design phase of the programme. These areas of focus, referred to as ‘Opportunity Areas’ (OA), were used to form committed local partnerships for engagement in co-design activities in the “*Deeper Discovery and Co-Design phase*” (name adopted to communicate the idea of further exploration of issues identified and co-design ideas around them).

In Northern Devon, the prioritised OAs identified were: (i) building stronger informal community support networks to promote mental health awareness and literacy (**OA1**); (ii) creating and enhancing access to more diverse opportunities for studies, employment, and recreation (**OA2**). A third OA, fostering a sense of identity and belonging, formed a cross-cutting theme due to its overlap with both OA1 and OA2. In Newham, the prioritised OAs were: (i) reducing the impact of violence and crime and enhancing safety (**OA3**); and (ii) strengthening the role of local community infrastructure and activities for wellbeing (**OA4**).

## Methods

This section describes the first iteration of Kailo’s Deeper Discovery and Co-Design phase (July 2023-June 2024), as implemented in Northern Devon and Newham.

### Deeper discovery and co-design phase overview

The Deeper Discovery and Co-design phase of Kailo was a co-design process of approximately nine months (July 2023-June 2024), delivered over 69 sessions and ad hoc engagements, involving CYP and Community members making decisions about how to respond to local challenges identified by them. It was designed to:

- Build on Early Discovery work and undertake **further research** around the OAs identified and prioritised in the Early Discovery phase **to identify root causes and systemic drivers** of CYPs mental health and wellbeing in the local context. Specifically:
  - ∘ Develop a more nuanced understanding of the prioritised OAs as defined and experienced by CYP and the broader local communities.
  - ∘ Gain a comprehensive understanding of the systemic behaviours driving the identified OAs and pinpoint areas or intervention points, i.e., places within a complex system where small shifts can generate a significant impact [59].
- **Generate ideas and co-design responses** around these OAs to support CYPs mental health and wellbeing within the Kailo pilot sites.
- **Engage and collaborate** with key community members and professionals related to the identified OAs, including CYP, youth and community organisations, local commissioners, and other critical actors.
- **Foster local partnerships’ capacity** to engage in the Deeper Discovery and Co-Design phase by gradually transitioning ownership from the Kailo team to these partnerships, ensuring the process is community-led and owned.

To enable these aims, the Kailo Framework used a blended approach which included (i) integrated research & design elements; (ii) participation infrastructure; and the (iii) co-design process and analysis (detail provided below). These describe initial planning structures and approaches that will be further discussed in the results section, reflecting the adaptive nature of this approach that was continuously shaped and improved by those involved.

#### Integrated research & design components

The Deeper Discovery and Co-Design process was grounded in the use and integration of two broad methodologies, co-design and group model building (each described below), supported by considerations of the extant literature and evidence integration.

##### Co-design

Co-design, as introduced earlier and defined by Blomkamp [5], signifies the active involvement of a diverse range of participants in exploring, developing, and testing responses to shared challenges. This approach enables strategy and intervention design efforts to centre the voices of CYP in shaping and defining supports, interventions, and strategies that are specific and appropriate to their mental health needs [23, 60].

##### Group model building (GMB)

GMB is a participatory approach developed within the field of system dynamics to facilitate group decision-making and problem structuring [61]. It allows for the consideration and better understanding of complex problems through mapping and modelling processes that capture underrepresented perspectives and emphasise consensus building, which is critical for sustainable community-led change [62]. GMB was integrated into the Kailo co-design process (further details and protocols for this element of the Kailo design process are described by Keenan et al. [63]).

##### Research evidence integration

Evidence was incorporated in the Deeper Discovery and Co-design process through evidence briefings (available to download from Anna Freud [64, 65] and a rapid scoping review [66] produced by Anna Freud. These were intended to: (a) offer insight into the current state of academic evidence [67] around the topics explored in the Co-design Circles; (b) surface insights not otherwise considered; and (c) provide a generalised consideration of what prior research indicates is impactful, ineffective, or harmful, which may be considered in relation to the local context, knowledge, and lived experience of co-design teams [67]. Evidence is also foundational for implementation [68] of effective interventions, approaches and strategies, and its integration was intended to support the co-design process to be more effective.

#### Participation infrastructure

Community involvement and participation must be flexible and meaningful [8]. In Kailo, this required diverse approaches to engagement to ensure all community members could participate in ways that suited them, while centring unheard or excluded perspectives [56]. The Deeper Discovery and Co-design participation infrastructure was developed to incorporate various participation levels, emphasising the shift of decision-making power to CYP and community members by positioning them as central co-designers. It involved a layered approach that aimed to effectively balance resources, power, and influence, acknowledging that stakeholders contribute differently based on their unique expertise, experience, or sphere of influence [69]. Thus, these Co-design circles aimed to effectively expand or constrain these factors for different stakeholders.

**Fig. 1:**
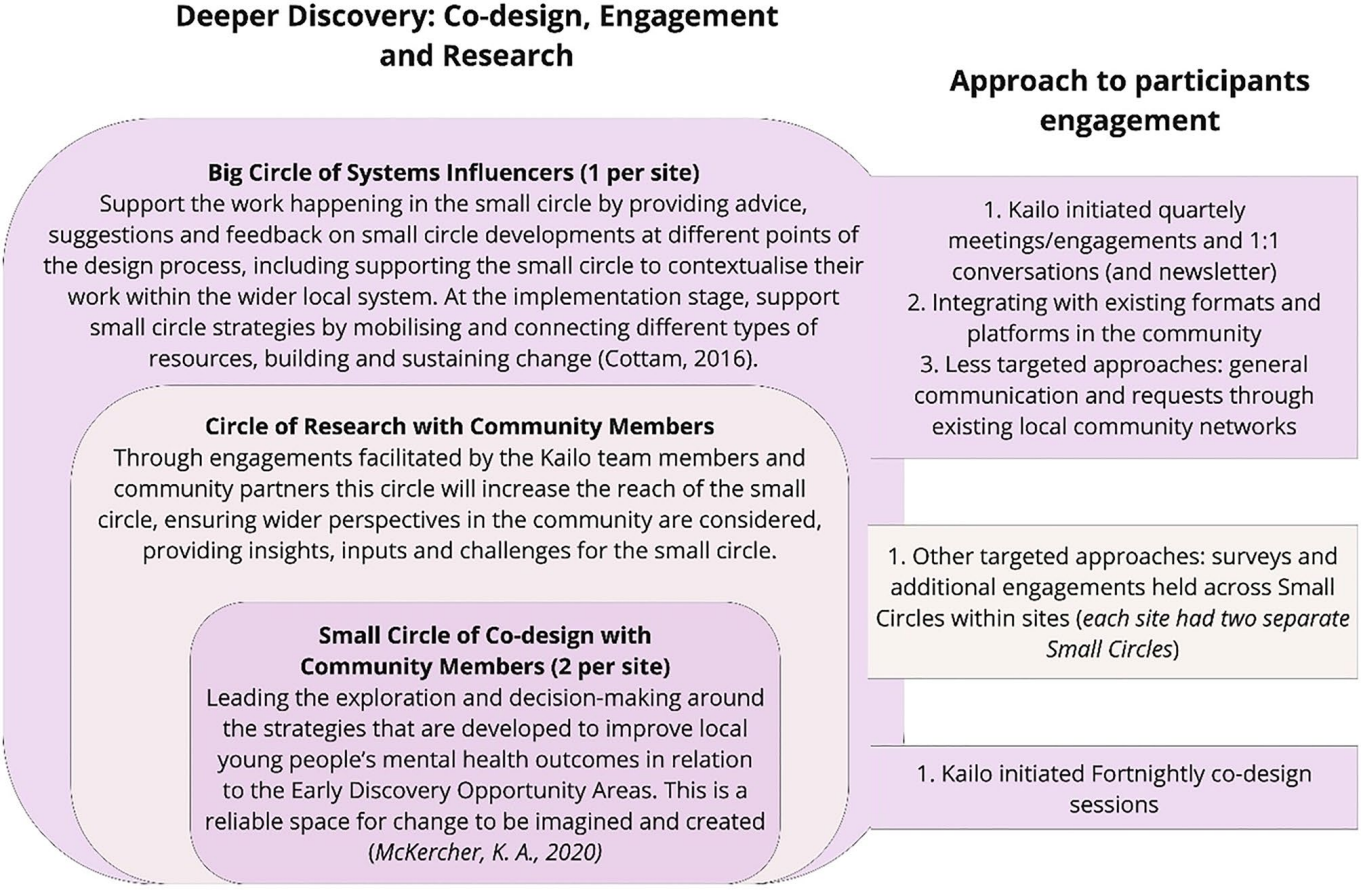
Overview of Deeper Discovery and Co-Design Approach: Co-design, Engagement and Research [43, 70]

As illustrated in Fig. 1, the Deeper Discovery and Co-Design phase comprised of three main groups:

1. **The ‘Small Circle’**: Led the co-design work and allowed for varying levels of participation and commitment to accommodate participants’ preferences, specific needs, and availability [48]. These are based on McKercher’s [43] Small Circles, where psychological safe spaces, relationships and trust are built between co-designers. (Two per site, each focused on one of the OAs identified). In line with the Lundy model [[37]], these circles also provided a space where CYP’s and community members, voices could be expressed.

As part of Kailo’s co-design methodology, wider circles (‘Circle of Research’ and ‘Big Circles’) were established to provide the knowledge, support and resources necessary for Small Circle members to effectively co-design strategies and interventions to address the prioritised OAs and ensure pathways for implementation. In line with the Lundy Model [37], this would also ensure an audience to listen to Small Circle ideas as the process unfolded and influence (through Big Circle members) for views to be acted upon.

2. **The ‘Circle of Research’**: Undertook the supplementary community research as required by the Small Circle.
3. **The ‘Big Circle’**: Comprised of local policy, commissioning, and practice leaders and professionals who provided regular review, input, connection to related work, and championing efforts. (One per site, focused on providing support across OAs)

All Co-design Circles’ adult members had a role - directly or indirectly - in supporting CYPs mental health and wellbeing within local community areas.

**Fig. 2:**
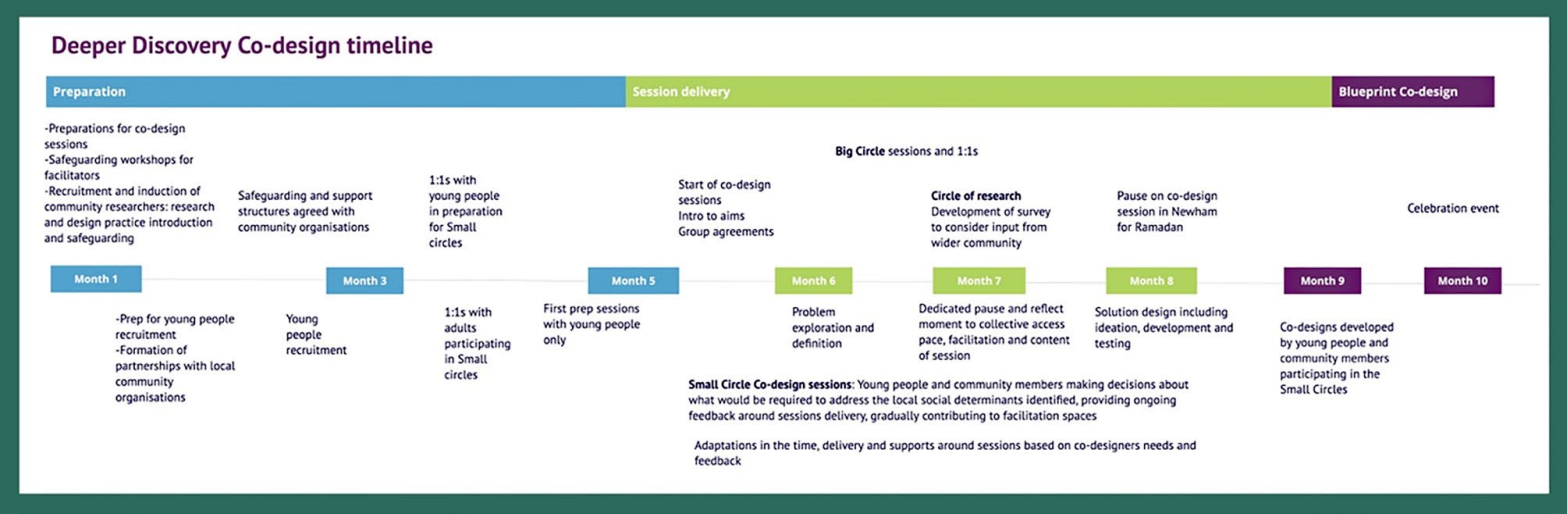
Deeper Discovery and Co-design timeline

These circles were designed and facilitated by Kailo team members and supported by local community partners (See timeline of activities below, Fig. 2). The Kailo team developed partnerships with organisations that brought different areas of expertise to the programme, funded through grant agreements. They worked alongside the Kailo team, contributing their expertise, relationships, and knowledge to strengthen programme delivery. To ensure the process remained community-centred and focused on the specific needs and contexts of the participants, community members and youth community researchers were identified to support the facilitation of Small Circle sessions.

In Northern Devon, this included one Designer, one Researcher, two Community Researchers, and three to five individuals from Kailo Community Partner organisations. In Newham, this included three Researchers (Implementation Specialists), one Public Health Specialist, two Community Researchers and up to three individuals from Kailo Community Partner organisations. The intention was that Kailo Community Partner members would bring their expertise to the co-design process in addition to supporting the involvement and wellbeing of the CYP involved. These trusted local community members played important roles in supporting CYPs mental health and wellbeing (either directly or indirectly), in each site. (See Supplementary Material 1 for specific roles).

#### Sampling and recruitment

##### Small circle of co-design

Kailo placed a particular focus on engaging CYP aged 12–25, who are most impacted and often less heard regarding the OAs identified in each site. The recruitment strategy and sampling were informed by insights that certain groups were not sufficiently engaged during the Early Discovery phase [56]. These groups varied across the sites and included neurodivergent CYP, those identifying as LGBTQIA+, those living in rurally isolated areas in Northern Devon, and CYP from racially and ethnically minoritised groups in Newham. In response to this, the recruitment inclusion criteria for the co-design phase focused on individuals belonging to these underrepresented groups and others with relevant lived experiences of the OAs prioritised in the Early Discovery phase.

To facilitate broader engagement across diverse groups, CYP were recruited through Kailo Community Partners, who approached individuals with whom they had established relationships and/or those they assessed as likely to engage effectively in a group setting. These were CYP involved in youth work activities or members of the communities these organisations worked with. Adult community members participating in the Small Circles were recruited by Kailo Community Partners and Site Teams. These were youth workers and parents involved in the Kailo Early Discovery phase. This relationship-based recruitment aimed to increase trust and support inclusive participation based on knowledge of participants’ needs. Both CYP and adults (when participation took place outside of their usual employment) were compensated for their involvement in the Small Circles, in line with the National Living Wage rate [71].

The Kailo Site Team created a guide based on insights from the Early Discovery phase and Kailo Community Partner’s experiences, which included questions and prompts to support conversations with CYP (Supplementary Material 2). This was used to assess their comfort in the co-design sessions and identify any reasonable adaptations needed to improve session accessibility, alongside other resources such as simplified information sheets (Supplementary Material 3 and 4). Some community members and professionals also played safeguarding roles and provided additional support for specific CYP attending the sessions.

##### Circle of research

In addition to the core ‘Small’ and ‘Big’ Circle members, at specific points in the co-design process the Small Circle, Kailo Site Teams, and Kailo Community Partners consulted with members of the wider local community. Surveys involving other CYP and community members, were used to inform prioritisation or design decisions in the Small Circle sessions. The intention was to ensure that the co-design process was informed by a broader spectrum of community input, enhancing the relevance and impact of the design decisions made by the Small Circle to the local context. Surveys were conducted targeting community members living in the Kailo pilot sites. Participants were recruited through the sharing of surveys online through social media platforms (LinkedIn™, TikTok™ and Instagram™), and via Small Circle participants.

##### Big circle

Big Circle recruitment aimed to represent local systems leadership [72], and “*local people who represent and speak for their community*” [73], encompassing local community leaders, youth and community organisational representatives, public systems leaders, funders and commissioners, education providers, and academics (See Supplementary Material 5 for the types of organisations represented by participating Big Circle members). Members of the Big Circle were identified and engaged through the Early Discovery phase [56], via (a) snowballing recommendations from existing Big Circle members and Kailo Community Partners; (b) invitations to specific Kailo forums and presentations; and (c) memberships to pre-existing forums, such as relevant local authority or health system boards, or professional network meetings.

Participants were typically individuals or representatives from organisations with power, specialism, or influence regarding the specific OA themes or broader CYPs mental health topics.

**Fig. 3:**
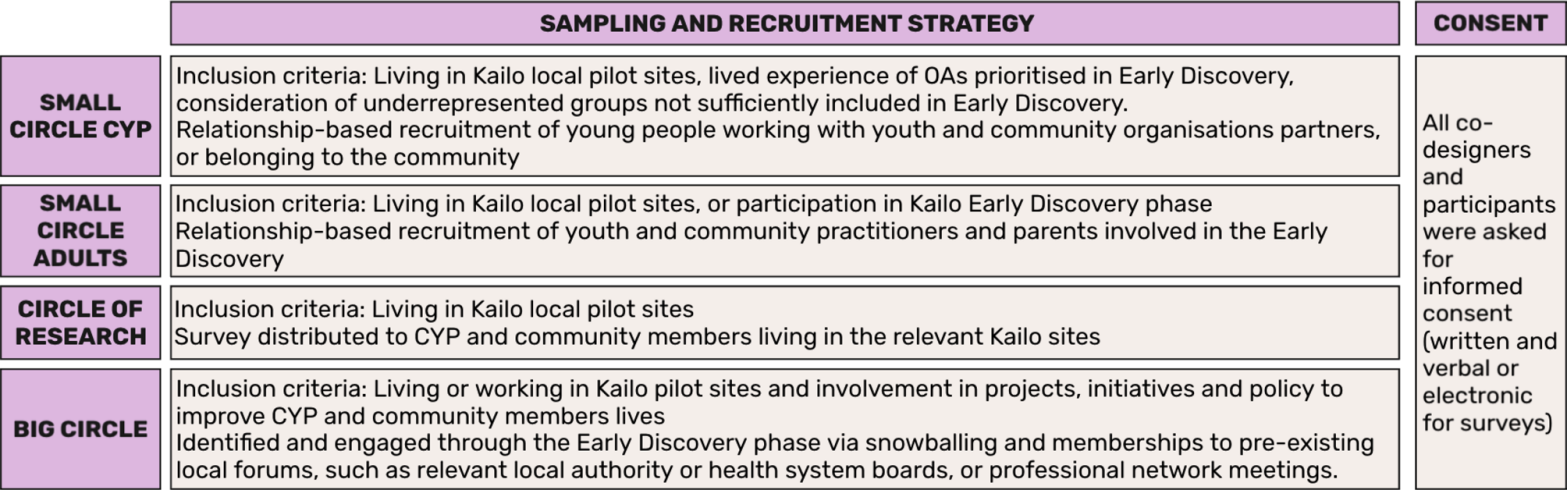
Summary of sampling and recruitment strategy

All participants, across the Deeper Discovery Circles, were asked for informed and written consent. Figure 3 provides a summary of the sampling and recruitment strategy across the Deeper Discovery Circles.

#### Co-design process and analysis

##### Delivery settings

###### Small circles

In Northern Devon, sessions were facilitated at a local non-profit building that offers therapeutic support for CYP and their families, as well as at a local youth centre. In Newham, sessions were facilitated across two youth centres and a community centre. Sessions occurred weekly or fortnightly, depending on participants availability and process needs. Twelve consecutive in-person fortnightly sessions of approximately 2.5 hours were initially planned. Sixteen were actually delivered across each of the two Small Circles in each pilot site.

###### Circle of research

Circle of Research engagements were facilitated online through surveys or through in-person focus groups across the sites. One survey was planned for each site to support the refinement of priorities before the co-design ideation planned sessions.

###### Big circles

Big Circle group sessions were conducted online, with additional one-to-one conversations with Big Circle members who were either unable to attend sessions or wished to support or engage further. These activities were facilitated by Kailo Site Team members, supported by Kailo Community Researchers and Designers. At least four online Big Circle engagements of 2.5 hours were initially planned across the sites. However, due to implementation conditions described in the results section, this was implemented differently across the sites, involving group workshops and one-to-one conversations.

##### Process

Table 2 presents a typology of the sessions conducted within the Small Circles, as well as activities, while Table 3 provides the broad structure that facilitators generally followed during each session. However, due to Kailo’s adaptive and responsive approach, sessions often blended different elements and encompassed aspects that extended beyond the defined typologies (See Table 2).

**Table 2:** Types of Small sessions and activities

Type of Session	Aims and Objectives of the sessions	Session Number	Broad focus and methods	Aims and objectives of the methods and approaches
Framing and scene setting sessions	Used to introduce new members to the Small Circles, as well as familiarising members with the aims of the Co-design process.	Prep sessions 1 and 2	• Small circle individual 121s • CYP induction to the group space	• Setting the space • Building relationships • Creating safe spaces
		Session 1, 2	• Small Circle aims • Group agreements and rules of engagement [74] • Roles and responsibilities • Hopes and Fears [75]	
Research & Design sessions	Focused on specific activities which enabled further development of the co-designs	Sessions 3,4,5	• Presentation of Opportunities areas prioritised in Early Discovery • Group Model Building sessions [76]: • Exploration of trends over time • Connection circles [77]	• Mapping the system associated with the OAs • Understanding structural influences • Building Casual Loop Diagrams [78]
		Session 6	• Considering places to intervene [59]	• Identifying places to design change
Reflection sessions	Used to enable reflections around the process and its outputs. These reflections often shaped following session facilitation style and the approaches taken to co-design strategy development.	Session 7	• Reflections on process and outputs	• Reflecting on sessions process, supports and content to date
Prioritisation sessions	Used to support Small Circle participants in the process of prioritising ideas, approaches and next steps, and build consensus around their decisions.	Sessions 8, 9	• Persona development and connection to social determinants [79; 80] • Experience mapping, • Prioritisation around systems map	• Further prioritisation • Identification of key stakeholders • Introducing members
Research & Design sessions	See above	Sessions 10, 11	• Experience mapping • Relationship building and creating safe spaces and group agreements with new members • Vision and journey mapping based on this	• Building relationships with new members • Creating safe spaces
		Session 12	• Building shared understanding with professionals that joined Small Circles	• Envisioning new futures [81]
		Session 13	• Journey mapping and visioning [82] • Goals and vision/generating designs	• Co-designing change
		Session 14	• Blueprint development [83]	
		Session 15	• Refining of co-design blueprints	
Reflections sessions	See above	Session 16	• Session to prepare for celebration event • Reflections on the process and outputs • Celebration and sharing learning with wider community members	• Celebrating outputs • Sharing learning and considering next steps

**Table 3:** Broad structure of Small Circle sessions

Components	Details
Check-in and boundary setting	All Small Circle sessions included a check-in element, involving assessment of participants’ moods and preferences for that specific session.
Introduction to activities of the day	Activities were introduced to ensure Small Circle participants were aware of the session agenda
Activity 1	Activities were often adapted to be individually completed at first, and then in pairs or smaller groups. Whenever possible, facilitators encouraged these smaller groups to share their thoughts, ideas and outputs with the wider group for further discussion.
Dinner break	Small Circle participants were provided with dinner during the sessions, although the timing of dinner within the schedule was agreed on a circle-by-circle basis.
Activity 2	Followed a similar approach to Activity 1.
Feedback and reflections	Before closing the session, facilitators often encouraged group reflection and or feedback on the session.
Closing	Check-out and time for individual questions (which could be asked confidentially/not in front of the whole group)

Table 2 provides a broad outline of sessions which varied across the sites in terms of the content and pace, in keeping with the adaptive co-design methodological approach. More details are provided in Supplementary Material 6 and the results, which show how this unfolded based on this adaptive approach.

The research team’s positionality, including community researchers’ and partners lived and professional experience of living and working in Northern Devon, and more broadly the Kailo team’s lived experiences of marginalisation and exclusion, and the challenges of participatory approaches, directly informed the delivery of sessions. This shaped decisions around ensuring Small Circles were not burdensome and allowing the process to remain adaptive and inclusive. There was awareness among all involved around the potential extractive and performative nature of research and design, with an emphasis on flexibility and redistribution of power throughout the process.

##### Data collection & analysis

The following data was collected across the Co-design Circles:

- **Photos of ideas and insights** generated through Small Circle activities and their outputs including posters, cards, voice notes, collages, LEGO ©, artwork, mind maps, drawings, personas and experience journeys;
- **Field notes** from those facilitating and observing Deeper Discovery Co-design Circles;
- **Meeting notes** from regular reflections sessions between the Kailo Site Teams and Kailo Community Partners. These included insights and reflections on sessions and their outputs;
- **Whiteboard** notes from Big Circle sessions;
- **Survey data** from the Circle of Research;
- **Audio recordings** of Small Circle sessions to enrich data analysis from other sources and support future evaluation.

The information collected during these sessions was transferred to a digital whiteboard by the Kailo Site Teams, as this provided a collaborative workspace for data collection, remote collaboration, and communication. After each co-design session, various methods were used to analyse and summarise outputs, which supported Small Circle members to build on their discussions and learning. These approaches included clustering and thematic analysis [84]. In some instances, refinement was carried out by the Kailo Site Teams to distil session material into specific outputs such as systems maps, personas, and empathy or journey maps.

At intervals throughout the process, findings were presented back to the Big Circle either through scheduled online sessions or via ad-hoc sessions as required. This enabled sense-checking, feedback, and review. Big Circle members had the opportunity to contribute their reflections, which were incorporated into the Small Circle insights and outputs. These were then presented back to the Small Circle members in subsequent sessions, allowing for multiple cycles of sensemaking and feedback.

Prioritisation exercises were also facilitated within the Small Circle sessions (either during the sessions or following the thematic analysis) to identify areas of focus at different stages of the process. These activities were informed by wider community research, rapid engagements with research literature, surveys, and focus groups, allowing for external input and diverse knowledge and expertise to be incorporated into the prioritisation exercises.

###### Regular cycles of reflection

**Fig. 4:**
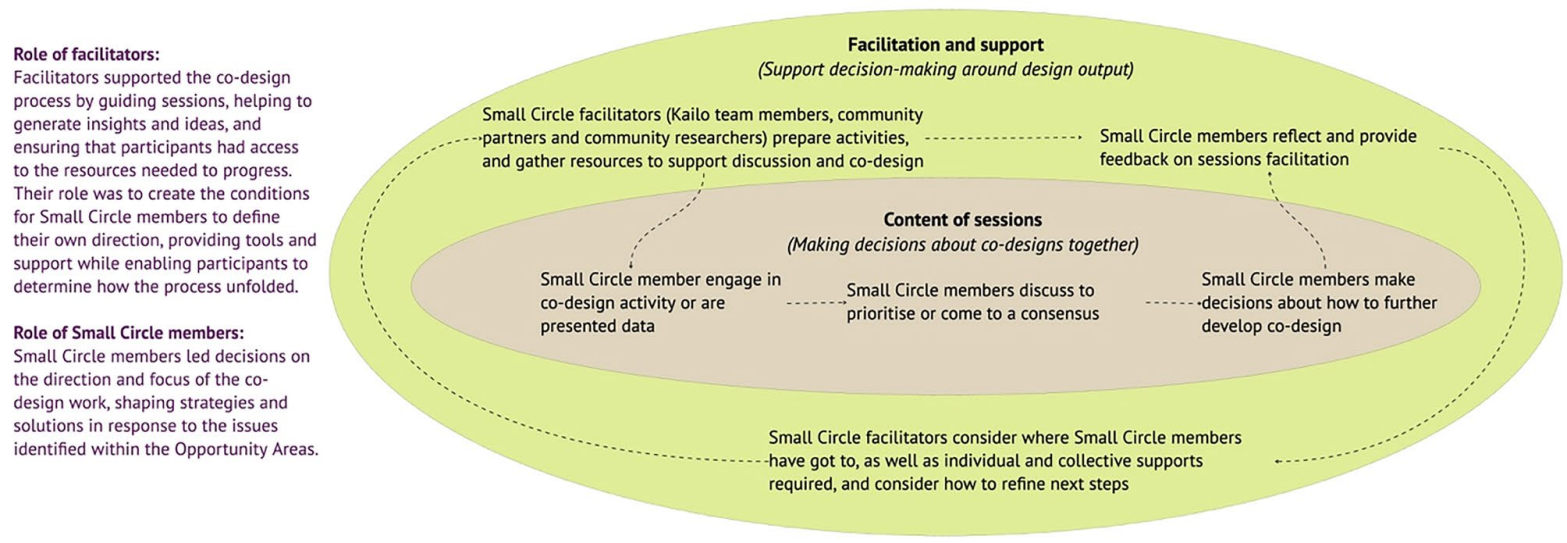
Roles and responsibilities within the co-design process

**Fig. 5:**
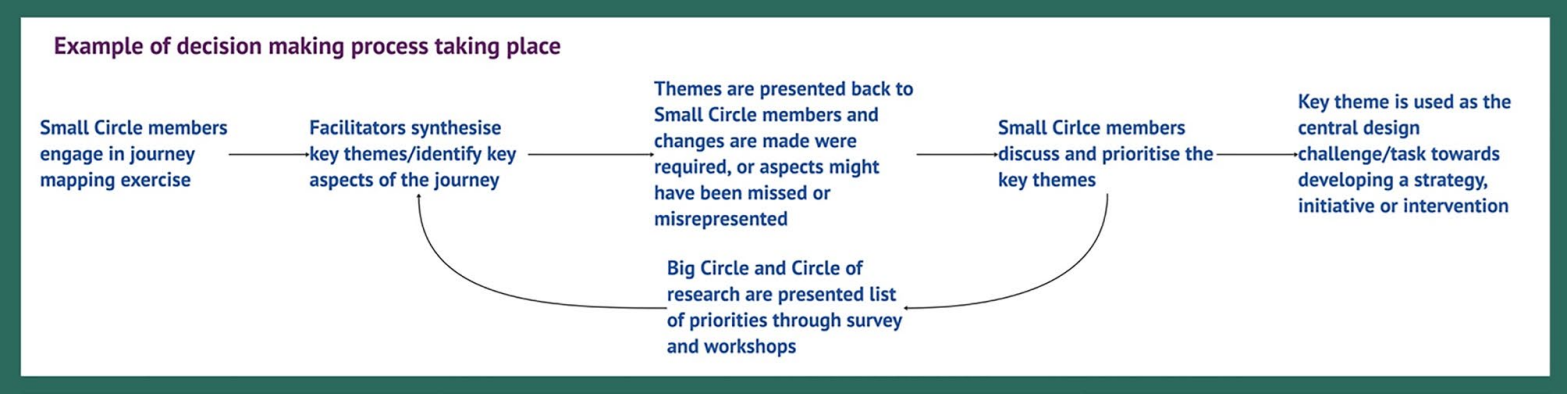
Example of decision-making process taking place

Throughout the Deeper Discovery and Co-Design phase, the Kailo Site and Community Partner teams engaged in regular cycles of review and reflection to analyse outputs and discuss adaptations to the approaches based on CYPs views and needs, and the observations of the delivery and support teams (See Figs. 4 and 5, which include details of facilitators and Small Circle roles, and an example of how the decision-making process worked). This was implemented to ensure facilitators’ views and influence were regularly acknowledged and interrogated. Kailo Community Partners were involved in co-facilitating Small Circle sessions with Kailo Site Teams, as well as reviewing insights and findings from Small and Big Circle sessions. (Supplementary Materials 7, 8, 9 provide samples of feedback forms, discussions and summaries from these sessions).

### Ethical considerations

The ethics process identified several important considerations relevant to co-design. These included the participation of CYP from broad age groups in the Small Circle sessions (12–25 years), and the mixing of adults and CYP in these spaces. This was raised as a challenge in terms of consent processes [85], and power differentials [10]. Similar considerations were also raised by Kailo Community Partners, with concerns surrounding potential safeguarding issues and imbalances in power dynamics, including potential exposure of younger participants to age-inappropriate topics, as well as the potential dominance of older participants’ views (as discussed in the literature [29, 86, 87]. Sendra [88] highlights some of these issues and puts an emphasis on the need for researchers to have sufficient skills to facilitate collective thinking and address power imbalances through existing networks and relationships.

The Kailo Site teams carefully considered these concerns and took steps to mitigate these risks. Small Circle members generally were aged 16–25-year-olds, with younger participants (12–15 years) being selectively included through recommendations from parents/carers and Kailo Community Partners. Recruitment into the Co-design Circles, and particularly Small Circles, was also focused on the selection of CYP and community members who were committed to group welfare, determined via discussion and exploration of group agreements and ground rules with the individuals; CYP led on the formation of these agreements and rules, which helped from the outset to acknowledge and address the potential power imbalances that may occur within a shared space.

To ensure the safety of all participants in the Small Circle sessions, Kailo implemented a comprehensive approach to addressing psychological distress and safeguarding concerns, grounded in researchers’ positionality and experience in applying trauma-informed practices. In practice, this entailed the Kailo Site Teams receiving safeguarding training, developing a risk assessment tool (Supplementary Material 10), and participating in safeguarding reflection sessions every three months throughout the facilitation period of the Small Circles.

A structured process was established for the facilitation team to ensure the necessary supports were in place, both for CYP and the adults and facilitators in the room (Supplementary Material 11). Safeguarding leads from organisations within the Kailo Consortium, as well as designated safeguarding officers for all Kailo Site Team facilitators, were assigned. These officers were tasked with being available to facilitators at the end of co-design sessions to offer support through reflection and debriefing when needed and as appropriate. Through these approaches, the Kailo Team and Community Partners aimed to ensure CYP rights were prioritised (particularly those from unheard groups) but did not limit their participation [89].

Ethical approval for the Kailo study and associated co-design activities was obtained from the University College London (UCL) Research Ethics Committee (REC). Project ID/Title 18,773/002. Further information on researchers’ positionality and expertise in relation to this area of study can be found in Supplementary Material 12.

## Results

Over approximately 9 months, the Kailo team facilitated 66 in-person Small Circle preparation and activity sessions, alongside five Big Circle sessions and a number of ad hoc one-to-one conversations. These involved 330 CYP and community members as part of the Deeper Discovery and Co-design phase across the pilot sites. The number of sessions, and the specific methods and tools used, evolved from the team’s initial plans in response to the feedback and needs of participants in all Co-design Circles. Sessions were designed to build on one another, while remaining flexible to the differing needs of the four Small Circle groups and the wider Big Circle participants. Supplementary Materials 6 and 13 provide details on session outlines, activities and materials. This section presents the conditions that shaped implementation, first through participation results, then through facilitation requirements, and finally, the broad methodology that emerged from this approach.

### Participants

The number of CYP and community members, professionals and researchers who participated to some degree in Kailo Co-design Circles (data consolidated across two sites) is outlined in Table 4.

**Table 4:** Number of participants who contributed to the Co-design Circles

Co-design Circle	Number of participants
Small Circle	• 61 CYP aged 12–25 (36% Northern Devon, 63% Newham) • 21 community members (1% Parents, 99% Youth and Community organisations: youth workers, neurodiversity advocates, health professionals) • 4 community researchers (50% Northern Devon, 50% Newham)
Big Circle	• 57 community professionals and system leaders who regularly engaged in Kailo initiated events (From organisations supporting children, young people and families (30%), education and research (19%), local government (16%), health and statutory services (17%), community and voluntary sector organisations (14%), and local businesses (3%).
Circle of research	• 187 CYP and community members who responded to the surveys (77% Northern Devon, 23% Newham)

Sociodemographic information about the CYP involved in the Small Circles is presented in Supplementary Materials 14. To protect the identities of co-designers, the data has been aggregated across both sites. It is important to note that this information was provided voluntarily by CYP, via a digital survey link. In one of the four Small Circles, no CYP provided this data. This group of predominantly older CYP also experienced some instability in attendance and a relatively high rate of participant dropout over time. Informal feedback suggested this was associated with competing commitments around work and life transitions.

### Co-design circles: conditions shaping participation

Implementation across sites revealed several conditions (barriers and enablers) that shaped participants’ engagement in the Small and Big Circles of co-design (Tables 5 and 6). These insights demonstrate how the methodology operated and what improvements were adopted in the process of methodological implementation.

**Table 5:** Conditions that supported and hindered participation Small Circle sessions (grouped by overall themes)

Theme	Barriers *(hindering participation)*	Enablers (Supporting participation)
Access to (safe) transportation	• CYP living in isolated rural areas had no access to reliable or affordable transport to attend sessions. • Some CYP had concerns about using public transport to cross Newham after dark.	• Session times were changed to fit CYPs needs and safeguarding • Taxis were also offered to enable CYP to safely access session locations.
Time of the sessions	• CYP expressed concerns about session timings, particularly as it got darker earlier due to the clock change in the Autumn (end of Daylight-Saving Time, DST)	• Session times were changed (made earlier) to address seasonal daylight changes, which were impacting CYP mood and motivation (This was also related to the point above around safeguarding.)
Emotional and Mental Readiness	• CYP experiencing mental health challenges may feel unable or unsafe to participate.	• Trusted adults and mental health professionals were present to provide support and continuity during the sessions. • All sessions were facilitated in a building with enough spaces/rooms for CYP to step out of conversations if required. • Sessions had individual and group activities.
Practical Flexibility and Scheduling	• Competing commitments (e.g., exams, work, Ramadan) made it difficult for some CYP to attend regularly or at all.	• Sessions were paused, rescheduled, or time-adjusted to fit around CYPs schedules and key life events.
Accessibility and Comprehension of Content	• Some participants found abstract or technical content (e.g., causal loop diagrams) challenging to engage with or interpret	• Youth-friendly language and relatable, real-life examples were used to support understanding and engagement
Inclusion and Diversity of Needs	• Conflicting needs among CYP with different neurotypes, faiths, or cultural backgrounds could reduce comfort or accessibility.	• Adjustments to timing, facilitation, and activity types (e.g. mix of individual writing and group talking tasks) were made to accommodate a range of participant needs and preferences
Knowledge and Confidence Gaps	• Lack of understanding about how systems work or how sessions could be relevant to mental health discouraged engagement for some	• Community members with relevant insights were involved, and information was shared across groups to support discussion
Motivation and Perceived Value	• While financial incentives were an important and legitimate part of this rights-based approach, they may have carried greater weight for CYP from low-income households or those facing socioeconomic challenges, potentially influencing their motivation for participation more strongly than for others.	• Group agreements, inclusive facilitation, reflection spaces, and opportunities to build relationships with other members were intentionally built in to support participants’ engagement and sense of contribution.
Purpose and Long-Term Impact	• Some CYP were unsure their active participation would make a difference. This uncertainty about the impact of their contributions reduced the level of engagement for some.	• As part of the co-design process, participants had opportunities to speak directly with local influencers and decision-makers, helping to show how their voices could lead to real change

**Table 6:** Conditions that supported and hindered participation within Big Circle sessions (grouped by overall themes)

Theme	Barriers *(hindering participation)*	Enablers (*Supporting participation*)
Time, Capacity, and Flexibility	• Limited time or capacity to engage, especially when engagement options were inflexible or time-consuming, and project timelines could not be extended.	• Offered flexible formats such as group sessions, one-to-one conversations, and varying levels of involvement. Timelines were extended were possible.
Alignment with Current Priorities	• Perception that co-design work didn’t align with immediate needs or priorities.	• Ongoing engagement by site teams to build relationships and align work with community members’ evolving priorities.
Role Clarity and Purpose	• Uncertainty about how their area of expertise related to Small Circle outputs or implementation reduced motivation to engage for some	• Targeted one-to-one conversations to discuss roles and how contributions would shape co-design and implementation.
Commitment to Youth and Systems Change	• Initial motivations among some community members varied regarding their prioritisation of youth participation and systems change goals	• Engagements focused on the value of youth voice and their right to participation, while also exploring potential solutions targeting systems and social determinants to improve adolescent mental health.
Feeling Heard and Valued	• Some community members expressed concerns about feeling unheard or having their contributions overlooked (in previous projects involving participation), which was reported to affect their trust and motivation.	• Efforts were made to acknowledge participants’ input and share how it related to decisions as part of the engagement process
Adaptive and Collaborative Approaches	• Processes described as rigid or siloed created barriers to collaboration and long-term engagement.	• Approaches were adapted and efforts were made to promote connected and coordinated ways of working throughout the process

### Facilitation approaches and requirements for implementation of the methodology

#### Ensuring flexibility and adaptation

Across sites, implementation of the co-design methodology required ongoing flexibility and continuous adaptation of both form and content. The process evolved in response to contextual differences, participant needs, and group dynamics. A central focus throughout was creating conditions that centred CYP perspectives and enabled equitable participation (See Table 5 for specific examples). To achieve this, facilitators offered multiple engagement routes for CYP and community members, including alternative session formats (See Tables 2 and 3 in methods, and Tables 5 and 6 in Results), varied facilitation styles, and asynchronous participation options (e.g., some co-designers were sometimes able to complete tasks at home due to commitments or other needs, and their insights were incorporated into Small Circle sessions).

Adaptations were also made to reflect differences in group composition and needs. Examples included modifying the pace and structure of sessions to support both neurodivergent and neurotypical participants (e.g., conducting activities using collage and drawing, creating spaces for individual, pairs and group discussions) and adjusting the schedule to accommodate religious observances such as Ramadan (See Table 5). The iterative approach allowed the co-design process to remain inclusive and responsive while maintaining progress towards shared objectives.

This flexibility was adopted to sustain participation and trust. Adaptations were typically co-decided within each circle, with facilitators drawing on participants’ feedback and reflections to revise tools and session flow (See Figs. 4 and 5). Over time, participants demonstrated increased confidence in articulating their views and in shaping the design process itself.

#### Integration of different approaches into the co-design methodology

##### Group model building (GMB)

Integration of GMB and evidence inputs from extant literature occurred throughout the process and was used to strengthen the link between lived experience and structural understanding of mental health issues. GMB sessions supported participants to connect personal challenges to broader determinants of wellbeing, facilitating movement from individual narratives to collective analysis. These activities helped establish consensus within each OA’s about the factors most relevant to their context. The maps produced as a result of these GMB sessions in each site provided a shared reference for subsequent design sessions, where strategies and ideas were generated. Detailed maps and accompanying narratives are provided in Supplementary Material 15 (Northern Devon), and Supplementary Material 16 (Newham).

##### Extant evidence

Evidence briefs were produced for one of the Small Circles in each site (OA2 in Northern Devon, and OA4 in Newham). These briefs were used differently by each site, depending on the ongoing process in the Small Circles at the time of their completion (See Evidence Briefs [64, 65], and Small Circle detailed timeline in Supplementary Material 6).

The originally planned rapid systematic reviews for the remaining two Small Circles at each site (OA1 in Northern Devon and OA3 in Newham) were reconsidered due to challenges in aligning research timelines with co-design priorities. Instead, a scoping review was conducted in Northern Devon to support future adaptations of the Kailo Framework in rural settings. In Newham, a more flexible and responsive approach was piloted, involving multiple cycles of targeted evidence appraisals. Throughout the Deeper Discovery and Co-design phases, the Newham team shared specific questions with the Anna Freud team, who conducted rapid literature scans to help address them.

While integration of literature evidence was not systematic, summaries of relevant findings were shared with groups to support reflection on how similar issues had been addressed elsewhere. Small Circle participants used these inputs to consider implications for wider populations beyond their immediate context.

### Inherent tensions of the process

#### Flexibility and pace

There can be inherent tensions between building and maintaining pace and momentum and ensuring flexibility and adaptation in a codesign process. Pace and momentum were achieved by planning a ‘sprint’ of approximately twelve co-design sessions in advance, along with relevant planning and content adaptation. However, to respond to emerging needs and priorities, changing participant availability and requirements for participation (See some of the conditions around this in Tables 5 and 6), or new lines of inquiry emerging from co-designers’ decisions after each session, some aspects of planned sessions often needed to be refined or changed. This resulted in a trade-off with momentum and pace to uphold inclusive and adaptive practice and maintain co-designers’ central role in decision-making. Timelines agreed with funders and community members did not always align with the adaptive nature of the process, creating pressure to balance responsiveness with delivery expectations.

#### Youth-led and youth-centred practice

A central consideration for the Kailo Site Teams across all circles was navigating between being youth-centred, i.e., prioritising the needs and wishes of CYP while collaborating as equals with other stakeholders, and being youth-led, where CYP initiated solutions to problems they had identified or defined themselves and took responsibility for developing and implementing those solutions. Within the context of designing responses to social determinants of mental health and discussing sensitive topics over an extended period, maintaining this balance required ongoing adjustments across sites. Varying levels of confidence, experience, and personal circumstances among CYP influenced the extent to which participants engaged with different stages of solution development. Managing this balance influenced how perspectives from other stakeholders in the community and wider system were incorporated across the sites. This had implications for the insights into structural and social influences in adult systems, which will be discussed below.

To address this, roles and responsibilities were clearly assigned and reinforced throughout the process across CYP, site facilitators, community members, and community partners (Figs. 4 and 5). Adult collaborators provided technical support and continuity, ensuring that CYP contributions were not lost once design activities concluded. Conditions that hindered and supported this process, particularly through the contributions of the Big Circle, are described in Table 6. This approach was used to prevent overburdening CYP and promoted collective ownership of outputs.

#### Co-design and GMB integration

During the integration of co-design and GMB activities, teams navigated differences between the emergent, relational format of co-design sessions and the more structured requirements of GMB. The latter required adherence to pre-defined scripts and the use of specific terminology, which, for some participants, was abstract or inaccessible. Facilitators employed visual prompts, simplified language, and iterative explanations to help participants engage with system-mapping tools while retaining the participatory ethos of co-design.

#### Co-designed and evidence-informed

Efforts to integrate rapid evidence synthesis with ongoing co-design activity highlighted differences in pace and process. Co-design required iterative cycles of exploration, sense-making, and consensus-building, whereas evidence synthesis followed a linear progression from question definition to analysis. In practice, co-design timelines often outpaced the completion of literature reviews, meaning that evidence summaries were sometimes introduced retrospectively rather than prospectively.

Despite this, teams used interim findings and relevant grey literature to inform discussions and refine design directions. This iterative integration was used to enable participants to situate their ideas within broader research and policy contexts. Although some opportunities for real-time evidence use were missed, the experience demonstrated that closer alignment of research and design cycles, with flexibility for asynchronous exchange, may be necessary to achieve a genuinely co-designed and evidence-informed process.

## Discussion

This paper presents a distinctive, adaptive, youth-centred co-design methodology developed and tested across two UK sites. Central to this was a three-layered model of participation (Co-design Circles), combined with a flexible and adaptive approach, and the integration of GMB and other forms of evidence. First, a Deeper Discovery and Co-design methodology is presented, synthesising key learning on a flexible, adaptive approach to co-designing with CYP to address the social determinants of mental health. Second, key methodological implementation themes are explored.

### Deeper discovery and Co-design phase methodology

**Fig. 6:**
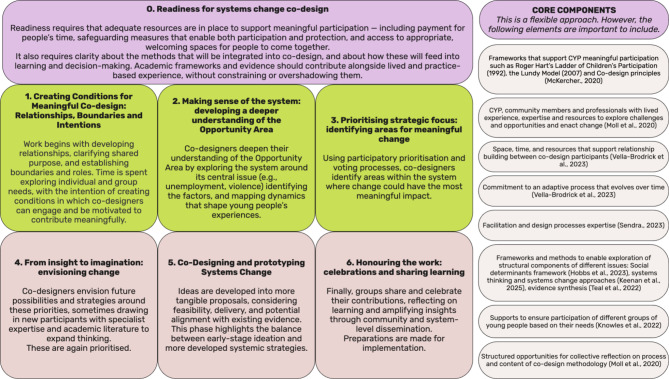
Overview of the Kailo Deeper Discovery and Co-design phases

Although there were some differences in delivery and in the pace at which Small Circles progressed through their milestones, broad phases of work could be identified (see Fig. 6). Together, these phases formed the basis of an adaptive, youth-centred co-design methodology aimed at developing strategies to address the social determinants of CYP mental health. Given the purposeful, flexible and adaptive nature of this process, we also present the core elements required for implementation of the methodology.

### Flexibility, integration and CYP participation

The methodology aimed to address several previously identified gaps, including challenges around engaging CYP in discussions about sensitive issues [32], ensuring inclusivity and representation [34], integrating evidence [33, 36, 48], and establishing clear methods for evaluating both the co-design process and its outcomes [27, 28, 33]. It presents an in-depth description of the methods utilised, demonstrating how co-design, combined with other methods, can be applied to a specific context. Drawing from the findings, three methodological themes were identified: (i) flexibility and adaptation, (ii) integration, and (iii) centring youth voice.

Although there is an increased use of co-design approaches that centre experiences of CYP in the past five years [19], participation may be limited to consultation or the initial phases of co-design, with the ultimate decision about the co-designs (their aims and constituent elements) often reverted to those considered to be in positions of power and influence [90]. Kailo’s participation infrastructure was used to ensure that CYPs perspectives remained central throughout the co-design process and that other perspectives could be integrated.

The Kailo Deeper Discovery and Co-Design methodology positioned CYP and community members, such as youth workers and parents, as lead co-designers within a layered participation structure. Consistent with the Lundy Model [37], CYP were supported with safe and inclusive opportunities to participate in the Small Circles (i.e., space and voice) and engaged through structured mechanisms that ensured these perspectives were actively listened to and acted upon (i.e., audience and influence). The Small Circles, primarily composed of CYP, held decision-making authority over how insights generated both within the circle and through broader activities within the Big Circles and Circles of Research were used to inform the co-design process.

The methodology also reflects the upper levels of participation described in Arnstein’s Ladder [38] and Hart’s Ladder [39], where power is shared, and participants exercise meaningful influence over research and design outcomes. The layered structure enabled the Kailo team to balance different forms of power, expertise and influence, while maintaining a clear emphasis on centring CYP when developing strategies to address the social determinants of mental health associated with the prioritised OAs.

However, prioritising CYP involvement in these circles presented some challenges, also noted in the literature [87], around the applicability and adoptability of youth-designed strategies in adult-led systems [91]. In such systems, adult leadership can sometimes be required to avoid placing the burden of problem-solving on some CYP themselves; power sharing and agency need to be contextually appropriate [92], and CYP need to be appropriately equipped for this (e.g., resources, support and training) [86]. It was therefore recognised that the responsibilities and capabilities required to further develop and implement certain co-designs needed to be shared with other individuals, organisations, and communities (particularly those with adult roles and responsibilities), thereby preventing the overburdening of CYP [93, 94].

The adaptive and reflexive approach illustrated that methodological flexibility, while challenging to manage within conventional research structures [55, 95], is essential to achieving meaningful and equitable participation when applying co-design principles with CYP, as noted in the literature [7, 16, 86]. Adaptation to context, needs, and priorities of CYP was prioritised from recruitment to facilitation of co-design sessions and development of strategies. The process also revealed that adaptation requires multiple lines of feedback for co-designers (e.g., through the recruitment process and within co-design sessions), a willingness among all co-designers involved to share uncertainty and negotiate evolving goals, and the importance of building in sufficient time, flexibility, and resources so the process can reach its potential without being unduly rushed or pressured by fixed timelines [43, 86, 96]. This can support CYP to build confidence in sharing experiences (i.e., building trust) and engaging effectively with reflective practice [97–99].

The methodology’s flexibility and adaptation were also shaped by the research team’s mixed positionality, combining insider knowledge from members who lived and worked in Northern Devon with outsider perspectives (non-residents). This supported reflexive and adaptive facilitation, enabling sensitivity to local contexts and lived experience while allowing critical questioning and interpretation. Positionality shaped decisions about how methods were adapted in response to participants’ needs and how insights were interpreted across sites. However, this may also have impacted the extent to which more efficient and time-saving approaches were explored when adopting adaptations in the process [69].

At the integrated methodological level, combining co-design with GMB and other evidence-gathering approaches offers a way to connect individual lived experiences with social determinants of mental health and wider population experiences [63]. The Kailo approach demonstrated the challenges and contributions of exploring how methods rooted in different epistemological traditions can be combined to support the development of strategies [100]. Importantly, the adaptations made in each site also underscored that there is no single or fixed model for such integration; rather, co-design with CYP in complex contexts requires continuous negotiation between methodological intent, and participant needs, preferences, and decisions [7, 8].

The experience highlights several implications for future co-design practice and research. First, the conditions for meaningful flexibility, time, resourcing, psychological safety, positionality and institutional trust must be designed and negotiated from the outset. Second, integrating approaches such as GMB within co-design processes requires explicit translation work to bridge different languages, logics, and forms of expertise. Third, youth-centred and co-design processes should be supported by infrastructures for shared leadership and distributed responsibility, preventing over-reliance on individual participants while maintaining the integrity of CYP voices in decision-making.

## Conclusion

The co-design methodology developed and adopted through the Kailo Deeper Discovery and Co-design phase prioritised youth and community perspectives and sought to promote inclusive and diverse engagement through the adoption of a distinctive, adaptive youth-centred approach. This facilitated a process focused on locally relevant strategies to address the social determinants of mental health. However, tensions and challenges arose, including around maintaining flexibility and adaptability, integrating rapid community insights with evidence synthesis, balancing youth-centred and youth-led approaches and distributing process roles and responsibilities equitably.

Key conditions for successfully implementing this methodology included strong collaboration with community members, flexible and adaptive timelines, a robust safeguarding infrastructure emphasising both participation and protection, and opportunities for multilevel engagement.

Kailo’s co-design methodology is now informing the development of the Kailo Framework, a community-informed process designed to generate contextually meaningful solutions to complex social challenges. Future evaluations will provide further insight into its impact, wider application and potential for scaling [55].

## supplementary material

Below is the link to the electronic supplementary material.

Supplementary Material 1

Supplementary Material 2

Supplementary Material 3

Supplementary Material 4

Supplementary Material 5

Supplementary Material 6

Supplementary Material 7

Supplementary Material 8

Supplementary Material 9

Supplementary Material 10

Supplementary Material 11

Supplementary Material 12

Supplementary Material 13

Supplementary Material 14

Supplementary Material 15

Supplementary Material 16

Supplementary Material 17

## Data Availability

Data available upon request.

## Abbreviations

CYP: Children and Young People
OAs: Opportunity Areas’
GMB: Group Model Building

## References

[1] Donetto S, Pierri P, Tsianakas V, Robert G. Experience-based co-design and healthcare improvement: realizing participatory design in the public sector. The Design Journal [Internet]. 2015 May 7 [cited 2025 Feb 6];18(2):227–48. Available from: 10.2752/175630615X14212498964312.

[2] CosgraveC, Kennedy A, Dietrich T, Gunn K, MacDonald J, McKay C, et al. From co-design to co-production: approaches, enablers, and constraints in developing a public health, capacity-building solution. Aust J Rural Health [Internet]. 2022 Oct 17 [cited 2025 Feb 6];30(6):738–46. Available from: 10.1111/ajr.12930. 10.1111/ajr.12930 PMC10092375 36250962

[3] LeaskCF, Sandlund M, Skelton DA, Altenburg TM, Cardon G, Chinapaw MJM, et al. Framework, principles and recommendations for utilising participatory methodologies in the co-creation and evaluation of public health interventions. Res Involv Engagem [Internet]. 2019 Jan 9 [cited 2025 Feb 6];5:2. Available from: 10.1186/s40900-018-0136-9. 10.1186/s40900-018-0136-9 PMC6327557 30652027

[4] LeiteH, Hodgkinson IR. Telemedicine co-design and value co-creation in public health care. Aust J Public Adm [Internet]. 2021 Mar 22 [cited 2025 Feb 6];80(3):559–77. Available from: 10.1111/1467-8500.12473.

[5] BlomkampE. The promise of co-design for public policy. Aust J Public Adm [Internet]. 2018 Mar 1 [cited 2025 Feb 6];77(4):729–43. Available from: 10.1111/1467-8500.12310.

[6] VargasC, Whelan J, Brimblecombe J, Allender S. Co-creation, co-design, co-production for public health: a perspective on definition and distinctions. Public Health Res Pract [Internet]. 2022 [cited 2025 Feb 6];32(2). Available from: https://www.phrp.com.au/issues/june-2022-volume-32-issue-2/co-creation-co-design-co-production-for-public-health-a-perspective-on-definition-and-distinctions/. 10.17061/phrp3222211 35702744

[7] O’BrienJ, Fossey E, Palmer VJ. A scoping review of the use of co-design methods with culturally and linguistically diverse communities to improve or adapt mental health services. Health Soc Care Com [Internet]. 2020 Jul 20 [cited 2025 Jul 1];29(1):1–17. Available from: 10.1111/hsc.13025. 10.1111/hsc.13105 32686881

[8] MulvaleG, Moll S, Miatello A, Murray-Leung L, Rogerson K, Sassi RB. Co-designing services for youth with mental health issues: novel elicitation approaches. Int J Qual Methods [Internet]. 2019 Jan 1 [cited 2025 Feb 6];18:160940691881624. Available from: 10.1177/1609406918816244.

[9] BrayEA, Everett B, George A, Salamonson Y, Ramjan LM. Co-designed healthcare transition interventions for adolescents and young adults with chronic conditions: a scoping review. Disabil Rehabil [Internet]. 2021 Oct 1 [cited 2025 Feb 6];43(21):1–22. Available from: 10.1080/09638288.2021.1979667. 10.1080/09638288.2021.1979667 34595986

[10] MollS, Wyndham-West M, Mulvale G, Park S, Buettgen A, Phoenix M, et al. Are you really doing “codesign”? Critical reflections when working with vulnerable populations. BMJ Open [Internet]. 2020 Nov 1 [cited 2025 Feb 6];10(11):e038339. Available from: 10.1136/bmjopen-2020-038339. 10.1136/bmjopen-2020-038339 PMC7640510 33148733

[11] ThabrewH, Fleming T, Hetrick S, Merry S. Co-design of eHealth interventions with children and young people. Front Psychiatry [Internet]. 2018 Oct 18 [cited 2025 Feb 6];9:1–12. Available from: https://www.ncbi.nlm.nih.gov/pmc/articles/PMC6200840/. 10.3389/fpsyt.2018.00481 PMC6200840 30405450

[12] AlbertA, Islam S, Haklay M, McEachan RRC. Nothing about us without us: a co-production strategy for communities, researchers and stakeholders to identify ways of improving health and reducing inequalities. Health Expect [Internet]. 2023 Jan 22 [cited 2025 Feb 6];26(2). Available from: 10.1111/hex.13709. 10.1111/hex.13709 PMC10010091 36683204

[13] Bevan Jones R, Stallard P, Agha SS, Rice S, Werner-Seidler A, Stasiak K, et al. Practitioner review: co-design of digital mental health technologies with children and young people. J Child Psychol Psychiatry [Internet]. 2020 Jun 22 [cited 2025 Feb 6];61(8):928–40. Available from: 10.1111/jcpp.13258. 10.1111/jcpp.13258 PMC7611975 32572961

[14] Veldmeijer L, Terlouw G, Van Os J, Van Dijk O, Van’t Veer J, Boonstra N. The involvement of service users and people with lived experience in mental health care innovation through design: systematic review. JMIR Ment Health [Internet]. 2023 Jul 25 [cited 2025 Feb 6];10:e46590. Available from: 10.2196/46590. 10.2196/46590 PMC10410372 37490326

[15] Luguetti C, Ryan J, Eckersley B, Howard A, Buck S, Osman A, et al. “It wasn’t adults and young people […] we’re all in it together”: co-designing a post-secondary transition program through youth participatory action research. Educ Action Res [Internet]. 2023 Apr 19 [cited 2025 Feb 6];1–18. Available from: 10.1080/09650792.2023.2203408.

[16] Knowles S, Sharma V, Fortune S, Wadman R, Churchill R, Hetrick S. Adapting a codesign process with young people to prioritize outcomes for a systematic review of interventions to prevent self-harm and suicide. Health Expect [Internet]. 2022 May 6 [cited 2025 Feb 6]; Available from: 10.1111/hex.13479. 10.1111/hex.13479 PMC9327872 35521681

[17] NHS Digital. Mental health of children and young people in England, 2023 – wave 4 follow up to the 2017 survey [Internet]. NHS England; 2023 [cited 2025 Nov 10]. Available from: https://digital.nhs.uk/data-and-information/publications/statistical/mental-health-of-children-and-young-people-in-england/2023-wave-4-follow-up.

[18] Prebeg M, Patton M, Desai R, Smith M, Krause K, Butcher N, et al. From participants to partners: reconceptualising authentic patient engagement roles in youth mental health research. Lancet Psychiatry [Internet]. 2023 Feb [cited 2025 Nov 10];10(2):139–45. Available from: https://www.sciencedirect.com/science/article/abs/pii/S2215036622003777. 10.1016/S2215-0366(22)00377-7 36502816

[19] Chinsen A, Berg A, Nielsen S, Trewella K, Cronin TJ, Pace CC, et al. Co-design methodologies to develop mental health interventions with young people: a systematic review. Lancet Child Adolesc Health [Internet]. 2025 Apr 1. [cited 2025 Nov 10]; Available from https://www.thelancet.com/journals/lanchi/article/PIIS2352-4642(25)00063-X/abstract. 10.1016/S2352-4642(25)00063-X 40311649

[20] ChingBCF, Foster A, Schlief M, Lewis G, Rajyaguru P. Co-producing school-based Mental Health Interventions with Young people, teachers, and schools: a Case Study. Res Involv Engagem [Internet]. 2024 Oct 24 [cited 2025 Nov 10];10(1). Available from: https://researchinvolvement.biomedcentral.com/articles/10.1186/s40900-024-00636-5. 10.1186/s40900-024-00636-5 PMC11506262 39449091

[21] Neill RD, Lloyd K, Best P, Williamson J, Allen J, Tully MA. Development and modelling of a school-based mental health intervention: the co-production of the R.E.A.C.T. programme. Curr Psychol [Internet]. 2022 Jun 1 [cited 2025 Nov 10]; Available from: 10.1007/s12144-022-03195-8.

[22] McGovern Ó, Glennon S, Walsh I, Gallagher P, McCashin D. The use of co-design with young people for digital mental health support development: a systematic review. Internet Interv [Internet]. 2025 Sep [cited 2025 Nov 10];41:100835. Available from: https://www.sciencedirect.com/science/article/pii/S2214782925000363#s0150. 10.1016/j.invent.2025.100835 PMC12163163 40521225

[23] Hackett CL, Mulvale G, Miatello A. Co-designing for quality: Creating a user-driven Tool to Improve Quality in Youth Mental Health Services. Health Expectations [Internet]. 2018 Apr 29 [cited 2025 Nov 10];21(6):1013–23. Available from: https://onlinelibrary.wiley.com/doi/full/10.1111/hex.12694. 10.1111/hex.12694 PMC6250867 29707865

[24] Mulvale A, Miatello A, Hackett C, Mulvale G. Applying experience-based co-design with vulnerable populations: lessons from a systematic review of methods to involve patients, families and service providers in child and youth mental health service improvement. Patient Exp J [Internet]. 2016 Apr 28 [cited 2025 Nov 10];3(1):117–29. Available from: https://pxjournal.org/journal/vol3/iss1/15/.

[25] Thorn P, Hill NT, Lamblin M, Teh Z, Battersby-Coulter R, Rice S, et al. Developing a suicide prevention social media campaign with young people (the #Chatsafe project): co-design approach. JMIR Ment Health [Internet]. 2020 [cited 2025 Nov 10];7(5):e17520. Available from: 10.2196/17520. 10.2196/17520 PMC7248803 32391800

[26] Hine R, Gladstone B, Reupert A, O’Dea L, Cuff R, Yates S, et al. StigmaBeat: collaborating with rural young people to co-design films aimed at reducing mental health stigma. Qual Health Res [Internet]. 2023 Nov 29 [cited 2025 Feb 6]; Available from: https://journals.sagepub.com/doi/pdf/10.1177/10497323231211454. 10.1177/10497323231211454 PMC11080393 38029299

[27] Peters S, Guccione L, Francis J, Best S, Tavender E, Curran J, et al. Evaluation of research co-design in health: a systematic overview of reviews and development of a framework. Implement Sci [Internet]. 2024 Sep 11 [cited 2025 Feb 6];19(1). Available from: 10.1186/s13012-024-01394-4. 10.1186/s13012-024-01394-4 PMC11391618 39261956

[28] Javanparast S, Robinson S, Kitson A, Arciuli J. Embedding research codesign knowledge and practice: learnings from researchers in a new research institute in Australia. Res Involv Engagem [Internet]. 2022 Dec 7 [cited 2025 Feb 6];8(1). Available from: 10.1186/s40900-022-00392-4. 10.1186/s40900-022-00392-4 PMC9730560 36476374

[29] Kehoe M, Whitehead R, de Boer K, Meyer D, Hopkins L, Nedeljkovic M. A qualitative evaluation of a co-design process involving young people at risk of suicide. Health Expect [Internet]. 2024 Feb 1 [cited 2025 Feb 6];27(1). Available from: 10.1111/hex.13986. 10.1111/hex.13986 PMC10859657 38343139

[30] Black B, Hendry B, Wright AC, Collings S. Co-design with people with lived experience: designing resources to communicate with children and young people in care about their family time contact visits. Br J Soc Work [Internet]. 2023 Jan 9 [cited 2025 Feb 6];53(3). Available from: 10.1093/bjsw/bcac133.

[31] Cusworth Walker S, Baquero B, Bekemeier B, Parnes MF, Arora K. Strategies for enacting health policy codesign: a scoping review and direction for research. Implement Sci [Internet]. 2023 Sep 21 [cited 2025 Feb 6];18(1). Available from: https://www.ncbi.nlm.nih.gov/pmc/articles/PMC10512571/. 10.1186/s13012-023-01295-y PMC10512571 37735397

[32] Wright LC, Lopez Chemas N, Cooper C. Lived experience codesign of self-harm interventions: a scoping review. BMJ Open [Internet]. 2023 Dec 27 [cited 2025 Feb 6];13(12):e079090. Available from: 10.1136/bmjopen-2023-079090. 10.1136/bmjopen-2023-079090 PMC10753750 38151276

[33] Slattery P, Saeri AK, Bragge P. Research co-design in health: a rapid overview of reviews. Health Res Policy Syst [Internet]. 2020 Feb 11 [cited 2025 Feb 6];18(1). Available from: 10.1186/s12961-020-0528-9. 10.1186/s12961-020-0528-9 PMC7014755 32046728

[34] King PT, Cormack D, Edwards R, Harris R, Paine SJ. Co-design for Indigenous and other children and young people from priority social groups: a systematic review. SSM Popul Health [Internet]. 2022 Jun [cited 2025 Feb 6];18:101077. Available from: 10.1016/j.ssmph.2022.101077. 10.1016/j.ssmph.2022.101077 PMC8983433 35402683

[35] Ní Shé É, Harrison R. Mitigating unintended consequences of co-design in health care. Health Expect [Internet]. 2021 Aug 2 [cited 2025 Feb 6];24(5). Available from: 10.1111/hex.13308. 10.1111/hex.13308 PMC8483209 34339528

[36] Morley C, Jose K, Hall SE, Shaw K, McGowan D, Wyss M, et al. Evidence-informed, experience-based co-design: a novel framework integrating research evidence and lived experience in priority-setting and co-design of health services. BMJ Open [Internet]. 2024 Aug 1 [cited 2025 Feb 6];14(8):e084620. Available from: 10.1136/bmjopen-2024-084620. 10.1136/bmjopen-2024-084620 PMC11404138 39122385

[37] Lundy L. “Voice” is not enough: conceptualising Article 12 of the United Nations Convention on the rights of the child. Br Educ Res J [Internet]. 2007 Dec [cited 2025 Nov 10];33(6):927–42. Available from: https://commission.europa.eu/system/files/2022-12/lundy_model_of_participation_0.pdf. https://commission.europa.eu/system/files/2022-12/lundy_model_of_participation_0.pdf.

[38] Arnstein SR. A Ladder of Citizen Participation. J Am Inst Plann [Internet]. 1969 [cited 2025 Nov 10];35(4):216–24. Available from: https://www.tandfonline.com/doi/abs/10.1080/01944366908977225.

[39] Hart RA. Children’s participation: from tokenism to citizenship [Internet]. Papers. Innocenti Essay; 1992 [cited 2025 Nov 10]. Available from: https://ideas.repec.org/p/ucf/inness/inness92-6.html. https://ideas.repec.org/p/ucf/inness/inness92-6.html.

[40] MacKenzie A. Credible, competent contributors: children and young people as postdigital citizen social scientists. Postdigit Sci Educ [Internet]. 2024 Oct 28 [cited 2025 Nov 10]; Available from: 10.1007/s42438-024-00517-w.

[41] Dietkus R. The call for trauma-informed design research and practice. Des Manag Rev [Internet]. 2022 May [cited 2025 Nov 10];33(2):26–31. Available from: 10.1111/drev.12295.

[42] Wheeler G, Mills N, Ankeny U, Howsley P, Bartlett C, Elphick H, et al. Meaningful involvement of children and young people in health technology development. J Med Eng Technol [Internet]. 2022 Jul 19 [cited 2025 Nov 10];46(6):462–71. Available from: 10.1080/03091902.2022.2089252. 10.1080/03091902.2022.2089252 35852341

[43] McKercher KA. What is co-design? [Internet]. Beyond Sticky Notes; 2020 [cited 2025 Feb 6]. Available from: https://www.beyondstickynotes.com/what-is-codesign.

[44] Blomkamp E. Shades of co-design [Internet]. Emma Blomkamp. 2024 [cited 2025 Jul 1]. Available from: https://emmablomkamp.com/blog/shades-of-co-design.

[45] Rahman A, Nawaz S, Khan E, Islam S. Nothing about us, without us: is for us. Res Involv Engagem [Internet]. 2022 Aug 4 [cited 2025 Feb 6];8(1). Available from: 10.1186/s40900-022-00372-8. 10.1186/s40900-022-00372-8 PMC9351243 35927767

[46] Masterson D, Areskoug Josefsson K, Robert G, Nylander E, Kjellström S. Mapping definitions of co-production and co-design in health and social care: a systematic scoping review providing lessons for the future. Health Expect [Internet]. 2022 Mar 23 [cited 2025 Feb 6];25(3):902–13. Available from: 10.1111/hex.13470. 10.1111/hex.13470 PMC9122425 35322510

[47] Vargas C, Whelan J, Brimblecombe J, Allender S. Co-creation, co-design, co-production for public health – a perspective on definition and distinctions. Public Health Res Pract [Internet]. 2022 [cited 2025 Feb 6];32(2). Available from: https://www.phrp.com.au/issues/june-2022-volume-32-issue-2/co-creation-co-design-co-production-for-public-health-a-perspective-on-definition-and-distinctions/. 10.17061/phrp3222211 35702744

[48] Green T, Bonner A, Teleni L, Bradford N, Purtell L, Douglas C, et al. Use and reporting of experience-based codesign studies in the healthcare setting: a systematic review. BMJ Qual Saf [Internet]. 2019 Sep 23 [cited 2025 Feb 6];29(1):BMJqs-2019-009570. Available from: 10.1136/bmjqs-2019-009570. 10.1136/bmjqs-2019-009570 31548278

[49] Teal G, McAra M, Riddell J, Flowers P, Coia N, McDaid L. Integrating and producing evidence through participatory design. CoDesign [Internet]. 2022 Jul 11 [cited 2025 Feb 6];1–18. Available from: 10.1080/15710882.2022.2096906.

[50] Lynch E, Bulto L, West M, Cadilhac DA, Cooper F, Harvey G. Codesigning implementation strategies to improve evidence-based stroke rehabilitation: a feasibility study. Health Expect [Internet]. 2023 Nov 21 [cited 2025 Feb 6];27(1). Available from: https://www.ncbi.nlm.nih.gov/pmc/articles/PMC10757151/. 10.1111/hex.13904 PMC10757151 37990469

[51] Fylan B, Tomlinson J, Raynor DK, Silcock J. Using experience-based co-design with patients, carers and healthcare professionals to develop theory-based interventions for safer medicines use. Res Soc Adm Pharm [Internet]. 2021 Jun [cited 2025 Feb 6];17(12). Available from: 10.1016/j.sapharm.2021.06.001. 10.1016/j.sapharm.2021.06.004 34187746

[52] Hobbs T, Santana de Lima E, Bevington D, Preece C, Allen K, Barna P, et al. Kailo: a systemic approach to addressing the social determinants of young people’s mental health and wellbeing at the local level. Wellcome Open Res [Internet]. 2023 Nov 13 [cited 2025 Nov 10];8:524. Available from: https://www.ncbi.nlm.nih.gov/pmc/articles/PMC11126905/. 10.12688/wellcomeopenres.20095.1 PMC11126905 38798997

[53] Compton MT, Shim RS. The social determinants of mental health. Focus (Am Psychiatr Publ) [Internet]. 2015 Oct 22 [cited 2025 Feb 6];13(4):419–25. Available from: http://media.morehousetcc.org/RESEARCH_PROJECTS/THRIVE/PUBLICATIONS/Compton%20Shim%202015%20Clinical%20Synthesis%20Social%20Deter%20of%20Mental%20Health.pdf.

[54] Kirkbride JB, Anglin DM, Colman I, Dykxhoorn J, Jones PB, Patalay P, et al. The social determinants of mental health and disorder: evidence, prevention and recommendations. World Psychiatry [Internet]. 2024 Jan 12 [cited 2025 Feb 6];23(1):58–90. Available from: https://www.ncbi.nlm.nih.gov/pmc/articles/PMC10786006/. 10.1002/wps.21160 PMC10786006 38214615

[55] Allen K, March A, Alexandrescu B, Harris J, Stemp R, Li S, et al. Developing programme theory for a place-based, systems change approach to adolescent mental health: a developmental realist evaluation. PLoS Ment Health [Internet]. 2025 Jun 9 [cited 2025 Jun 25];2(6):e0000226. Available from: https://journals.plos.org/mentalhealth/article?id=10.1371/journal.pmen.0000226. 10.1371/journal.pmen.0000226 PMC12798369 41661938

[56] Santana De Lima E, Preece C, Potter K, Goddard E, Edbrooke-Childs J, Hobbs T, et al. A community-based approach to identifying and prioritising young people’s mental health needs in their local communities. Res Involv Engagem [Internet]. 2023 Nov 23 [cited 2025 Feb 6];9(1). Available from: https://kailo.community/wp-content/uploads/2023/11/s40900-023-00510-w.pdf. 10.1186/s40900-023-00510-w PMC10666450 37996912

[57] Mosleh WS, Larsen H. Exploring the complexity of participation. CoDesign [Internet]. 2020 Jul 3 [cited 2025 Aug 7];1–19. Available from: 10.1080/15710882.2020.1789172.

[58] Design Council. The Double Diamond [Internet]. London: Design Council; 2025 [cited 2025 Nov 10]. Available from: https://www.designcouncil.org.uk/our-resources/the-double-diamond/.

[59] Meadows D. Leverage points: places to intervene in a system [Internet]. Hartland, VT: The Sustainability Institute; 1999 [cited 2025 Feb 6]. Available from: . https://donellameadows.org/wp-content/userfiles/Leverage_Points.pdf.

[60] McKercher KA. Beyond sticky notes: co-design for real. Sydney, NSW: Reed; 2020.

[61] Scott RJ, Cavana RY, Cameron D. Recent evidence on the effectiveness of group model building. Eur J Oper Res [Internet]. 2016 Mar [cited 2025 Feb 6];249(3):908–18. Available from: https://www.sciencedirect.com/science/article/abs/pii/S0377221715006323.

[62] Siokou C, Morgan R, Shiell A. Group model building: a participatory approach to understanding and acting on systems. Public Health Res Pract [Internet]. 2014 Nov [cited 2025 Feb 6];25(1). Available from: https://pubmed.ncbi.nlm.nih.gov/25828443/. 10.17061/phrp2511404 25828443

[63] Keenan M, Freeman L, Santana de Lima E, Potter K, Hobbs T, Ballard E, et al. A systemic approach to identifying sustainable community-based interventions for improving adolescent mental health: a participatory group model building and design protocol. Health Res Policy Syst [Internet]. 2025 Jan 13 [cited 2025 Feb 6];23(1). Available from: https://pubmed.ncbi.nlm.nih.gov/39806346/. 10.1186/s12961-024-01247-y PMC11727203 39806346

[64] Anna Freud, Kailo. An evidence briefing on young people’s access to employment in rural communities [Internet]. 2024 [cited 2025 Jul 1]. Available from: https://brandplatform.annafreud.org/share/gBUc9fEtLQa32fPsUFJy/assets/3215.

[65] Anna Freud, Kailo. An evidence briefing on activities available outside school settings for young people [Internet]. 2024 [cited 2025 Jul 1]. Available from: https://brandplatform.annafreud.org/share/WwebLCcamsUFtWcW4WfE/assets/3216.

[66] McDougal E, Sheikh A, Santana de Lima E, Lereya ST, Edbrooke-Childs J, Deighton J, et al. Rural community-based interventions to improve the mental health and wellbeing of children and young people: a rapid scoping review of the quantitative and qualitative evidence. J Community Psychol [Internet]. 2025 Aug 30 [cited 2025 Nov 10];53(7). Available from: https://pubmed.ncbi.nlm.nih.gov/40884842/. 10.1002/jcop.70037 PMC12398394 40884842

[67] Beames JR, Kikas K, O’Gradey-Lee M, Gale N, Werner-Seidler A, Boydell KM, et al. A new normal: integrating lived experience into scientific data syntheses. Front Psychiatry [Internet]. 2021 Oct 29 [cited 2025 Feb 6];12:763005. Available from: https://www.ncbi.nlm.nih.gov/pmc/articles/PMC8585932/pdf/fpsyt-12-763005.pdf. 10.3389/fpsyt.2021.763005 PMC8585932 34777064

[68] Brownson RC, Shelton RC, Geng EH, Glasgow RE. Revisiting Concepts of Evidence in Implementation Science. Implementation Science [Internet]. 2022 Apr 12 [cited 2025 Aug 7];17(1). Available from: https://implementationscience.biomedcentral.com/articles/10.1186/s13012-022-01201-y. 10.1186/s13012-022-01201-y PMC9004065 35413917

[69] O’Donnell M, Collier M, Pineda-Pinto M, Cooper C, Nulty F, Castañeda NR. Redefining co-design for social-ecological Research and practice: a Systematic Literature Review. Environ Sci Policy [Internet]. 2025 Jan 14 [cited 2025 Aug 7];164:103998. Available from: https://www.sciencedirect.com/science/article/pii/S1462901125000140.

[70] Cottam H. Radical help: how we can remake the relationships between us and revolutionise the welfare state. London: Virago; 2019.

[71] GOV.UK. National minimum wage and national living wage rates [Internet]. Gov.uk; 2024 [cited 2025 Feb 6]. Available from: https://www.gov.uk/national-minimum-wage-rates.

[72] Bigland C, Evans D, Bolden R, Rae M. Systems leadership in practice: thematic insights from three public health case studies. BMC Public Health [Internet]. 2020 Nov 17 [cited 2025 Feb 6];20(1). Available from: https://bmcpublichealth.biomedcentral.com/articles/10.1186/s12889-020-09641-1. 10.1186/s12889-020-09641-1 PMC7673088 33203397

[73] Local Trust. Research team briefing #1: community leadership: What does the literature say about what makes an effective community leader? [Internet]. 2018 [cited 2025 Feb 6]. Available from: https://localtrust.org.uk/wp-content/uploads/2021/06/Leadership-lit-review.pdf.

[74] Seeds for Change. Group agreements: a short guide to creating group agreements for workshops and meetings [Internet]. 2021 [cited 2025 Nov 20]. Available from: https://www.seedsforchange.org.uk/downloads/groupagree.pdf.

[75] Luna-Reyes LF, Martinez-Moyano IJ, Pardo TA, Cresswell AM, Andersen DF, Richardson GP. Anatomy of a group model-building intervention: building dynamic theory from case study research. Syst Dyn Rev [Internet]. 2006 [cited 2025 Nov 20];22(4):291–320. Available from: https://onlinelibrary.wiley.com/doi/abs/10.100.

[76] Andersen DF, Richardson GP. Scripts for group model building. Syst Dyn Rev [Internet]. 1997 [cited 2025 Nov 20];13(2):107–29. Available from: https://onlinelibrary.wiley.com/doi/abs/10.1002/%28SICI%291099-1727%28199722%2913%3A2%3C107%3A%3AAID-SDR120%3E3.0.CO%3B2-7.

[77] Scriptapedia. Creating Causal Loop Diagram from Connection Circles - Wikibooks, open books for an open world [Internet]. Wikibooks.org; 2022 [cited 2025 Nov 20]. Available from: https://en.wikibooks.org/wiki/Scriptapedia/Connection_Circle.

[78] Scriptapedia. Connection circle - Wikibooks, open books for an open world [Internet]. Wikibooks.org; 2022 [cited 2025 Nov 20]. Available from: https://en.wikibooks.org/wiki/Scriptapedia/Connection_Circle.

[79] LeRouge C, Ma J, Sneha S, Tolle K. User profiles and personas in the design and development of consumer health technologies. Int J Med Inform [Internet]. 2019 Nov [cited 2025 Nov 20];82(11):e251–68. Available from: https://www.sciencedirect.com/science/article/abs/pii/S1386505611000724. 10.1016/j.ijmedinf.2011.03.006 21481635

[80] Salminen J, Wenyun Guan K, Jung SG, Jansen B. Use cases for design personas: a systematic review and new frontiers. CHI Conference on Human Factors in Computing Systems [Internet]. 2022 Apr 29 [cited 2025 Nov 20];1–21. Available from: https://dl.acm.org/doi/pdf/10.1145/3491102.3517589.

[81] Design Method Toolkit. Desi meth toolkit: lotus blossom [Internet]. toolkits.dss.cloud; 2024 [cited 2025 Nov 20]. Available from: https://toolkits.dss.cloud/design/method-card/lotus-blossom-2/.

[82] Service Design Tools. Journey Map | Service Design Tools [Internet]. Servicedesigntools.org. 2017 [cited 2025 Nov 20]. Available from: https://servicedesigntools.org/tools/journey-map.

[83] He F. An introduction to service design and a selection of service design tools: design methods for developing services [Internet]. www.academia.edu. [cited 2025 Nov 20]. Available from: https://www.academia.edu/24477788/An_introduction_to_service_design_and_a_selection_of_service_design_tools_Design_methods_for_developing_services.

[84] Naeem M, Ozuem W, Howell KE, Ranfagni S. A step-by-step process of thematic analysis to develop a conceptual model in qualitative research. Int J Qual Methods [Internet]. 2023 Nov 8 [cited 2025 Feb 6];22(1):1–18. Available from: https://journals.sagepub.com/doi/10.1177/16094069231205789.

[85] Hagell A, Benniche S. Engaging young people in health research and service design: key constructs and ethical challenges [Internet]. Nuffield Foundation; assoc Youn People’s Health; 2022 Mar [cited 2025 Feb 6]. Available from: https://www.nuffieldfoundation.org/wp-content/uploads/2020/08/main-report-Engaging-young-people-in-health-services-research-and-service-design.pdf.

[86] Vella-Brodrick D, Patrick K, Jacques-Hamilton R, Ng A, Chin TC, O’Connor M, et al. Youth Experiences of co-designing a well-being intervention: reflections, Learnings and recommendations. Oxford Review of Education [Internet]. 2023 May 2 [cited 2025 Feb 6];1–20. Available from: https://www.tandfonline.com/doi/pdf/10.1080/03054985.2023.2194621.

[87] Paterson-Young C, Adhikari J, Lee L, Maher M, Wright LHV. Creating ownership: Strengths and Tensions in co-production with children, Young people, and Adults across Contexts. Childhood [Internet]. 2024 Jul 22 [cited 2025 Feb 6]; Available from: https://pure.northampton.ac.uk/ws/portalfiles/portal/71225603/Paterson-Young_et_al_2024_Creating_ownership_Strengths_and_tensions_in_co-production_with_children_young_people_and_adults_across_contexts.pdf.

[88] Sendra P. The ethics of co-design. J Urban Des [Internet]. 2023 Feb 8 [cited 2025 Feb 6];29(1):1–19. Available from: https://www.tandfonline.com/doi/full/10.1080/13574809.2023.2171856#d1e119.

[89] Barker R, Plackett R, Price A, Canvin K, Hartwell G, Bonell C. Ethical challenges confronted in non-clinical, public health research with young people in England. Qualitative Research [Internet]. 2025 Jan 16 [cited 2025 Jul 1]. Available from: https://journals.sagepub.com/doi/10.1177/14687941241306241.

[90] Conway-Moore K, Knai C, Finegood D, Johnston L, Brinsden H, Aguiar A, et al. Co-creating obesity prevention policies with youth: policy ideas generated through the CO-CREATE project. Obes Rev [Internet]. 2023 Sep 1 [cited 2025 Feb 6];24(S2). Available from: https://onlinelibrary.wiley.com/doi/10.1111/obr.13623. 10.1111/obr.13623 37753599

[91] Santana de Lima E, Rehill N, Potter K, Preece C, Davis G, Bulmer S, et al. Addressing the social determinants of youth mental health in Newham and Northern Devon: reflections on co-design and systems change. BMC Public Health [in press]. 10.1186/s12889-026-26586-z PMC13019972 41721304

[92] Lipton B, Dickinson H, Bailie J, Hewitt B, Kavanagh A, Aitken Z, et al. Collaborating with young people: identifying the barriers and facilitators in co-designed research. Health Expect [Internet]. 2025 May 27 [cited 2025 Aug 7];28(3). Available from: https://pmc.ncbi.nlm.nih.gov/articles/PMC12117193/#hex70308-sec-0260. 10.1111/hex.70308 PMC12117193 40432242

[93] Kellett M. Small shoes, big steps! Empowering children as active researchers. Am J Community Psychol [Internet]. 2010 Jun 4 [cited 2025 Feb 7];46(1-2):195–203. Available from: https://onlinelibrary.wiley.com/doi/abs/10.1007/s10464-010-9324-y. 10.1007/s10464-010-9324-y 20524150

[94] Bailey S, Boddy K, Briscoe S, Morris C. Involving Disabled Children and Young People as Partners in research: a systematic review. Child: Care, Health and Development [Internet]. 2014 Oct 16 [cited 2025 Feb 7];41(4):505–14. Available from: https://onlinelibrary.wiley.com/doi/10.1111/cch.12197. 10.1111/cch.12197 25323964

[95] Pirinen A. The barriers and enablers of co-design for services. Int J Design [Internet]. 2016 Dec 31 [cited 2025 Feb 7];10(3):27–42. Available from: https://research.aalto.fi/en/publications/the-barriers-and-enablers-of-co-design-for-services.

[96] Benz C, Scott-Jeffs W, McKercher KA, Welsh M, Norman R, Hendrie D, et al. Community-based participatory-research through co-design: supporting collaboration from all sides of disability. Res Involv Engagem [Internet]. 2024 May 10 [cited 2025 Feb 6];10(1). Available from: https://link.springer.com/article/10.1186/s40900-024-00573-3. 10.1186/s40900-024-00573-3 PMC11084036 38730283

[97] Bradbury-Jones C, Isham L, Taylor J. The complexities and contradictions in participatory research with vulnerable children and young people: a qualitative systematic review. Soc Sci Med [Internet]. 2018 Oct [cited 2025 Feb 6];215:80–91. Available from: https://pubmed.ncbi.nlm.nih.gov/30218806/. 10.1016/j.socscimed.2018.08.038 30218806

[98] Raman S, French T. Enabling genuine participation in co-design with young people with learning disabilities. CoDesign [Internet]. 2021 Jan 26 [cited 2025 Feb 6];18(4):1–17. Available from: https://www.tandfonline.com/doi/full/10.1080/15710882.2021.1877728.

[99] Chavez-Ugalde Y, Toumpakari Z, White M, de Vocht F, Jago R. Using group model building to frame the commercial determinants of dietary behaviour in adolescence – proposed methods for online system mapping workshops. BMC Med Res Methodol [Internet]. 2022 Mar 27 [cited 2025 Feb 6];22(1). Available from: https://link.springer.com/article/10.1186/s12874-022-01576-y#citeas. 10.1186/s12874-022-01576-y PMC8961487 35350996

[100] Forrester-Bowling T, Lucas J, Brown A, Bennetts S, Carolin R, Hayward J, et al. Adapting group model building for mental healthcare: a participatory co-Design approach. Int J Ment Health Nurs [Internet]. 2024 Oct 11 [cited 2025 Feb 6]. Available from: https://onlinelibrary.wiley.com/doi/full/10.1111/inm.13451. 10.1111/inm.13451 PMC11771732 39394636

