# Supplementary material for: An adaptive, youth-centred co-design methodology: place-based co-design centring youth and community participation
Source: Res Involv Engagem. 2026 Jan 24;12:33. doi: 10.1186/s40900-025-00833-w (PMC12994241; doi:10.1186/s40900-025-00833-w)
Supplement: Supplementary file 1 — Supplementary Material 1 [file 40900_2025_833_MOESM1_ESM.docx]

*The Kailo Team Structure*

| **Kailo Consortium** | Academics, mental health researchers, public health specialists and designers from the following organisations:   - University College London - Dartington Service Design Lab - University of Exeter - UCLP | |
| --- | --- | --- |
| **Kailo Site Team (KST)** | The Kailo Site Team engaged with community members and facilitated sessions across the different circles in both pilot sites. The team included 4 researchers, 2 system dynamicists, 1 designer and a public health professional who were spread across the sites. This team also included 4 Community Researchers and Designers (KCRD) - 2 per site - who were young people living in the Kailo pilot sites with relevant lived experiences of the OAs prioritised in the Early Discovery phase and explored in the Small Circles of Co-design.  Per site there was 1 main facilitator (Senior researcher and designer), 1 support researcher, 1 KCRD and 1 system dynamicist. The other members would participate when required, or as part of the evaluation team. | |
| **Kailo Community Partners (KCP)** | Local community organisations across the Kailo pilot sites with interest and experience of working directly or indirectly to support CYP’s mental health and wellbeing. These community organisations were selected to contribute to the Deeper Discovery phase in two key functions:  **Co-pilot**: local organisations playing an integral role alongside the Kailo team in leading the co-design process and supporting young people and community members to participate in a ‘Small Circle’ co-design team.  **Navigator**: local organisations playing a lighter-touch supporting and participatory role in a Small-Circle co-design team. | |
|  | Northern Devon KCP | |
|  | OA: Diverse Opportunities Opportunity Area | |
|  | Co-pilots | Navigators |
|  | 1. Youth centre provider | 1. Organisation supporting neurodiverse CYP |
|  | OA: Mental Health literacy and informal community networks | |
|  | Co-pilots | Navigators |
|  | 1. Local organisation supporting the voluntary sector | 1. Organisation supporting neurodiverse CYP (same as above) 2. Local organisation providing mental health and wellbeing support for CYP and families 3. Youth-led organisation aimed at providing mental health education and support for CYP 4. Organisation promoting equality, diversity and social inclusion in the local area |
|  | Newham KCP | |
|  | OA: Violence and Crime | |
|  | Co-pilots | Navigators |
|  | 1. Local partnership organisation that promotes across-organisation collaborative working | 1. Organisation working with CYP who have experienced violence, crime, trauma or abuse and support them to access education, employment or training 2. Organisation providing training, education and sports activities for the local community |
|  | OA: Community Infrastructure and Activities for Wellbeing | |
|  | Co-pilots | Navigators |
|  | 1. Community-led organisation that focuses on the regeneration of Newham for local people | 1. Organisation offering training and employment opportunities for vulnerable and disadvantaged young people 2. Organisation offering vulnerable and disadvantaged young people access to opportunities, experiences and programmes that usually they would be unable to access |
