# Supplementary material for: An adaptive, youth-centred co-design methodology: place-based co-design centring youth and community participation
Source: Res Involv Engagem. 2026 Jan 24;12:33. doi: 10.1186/s40900-025-00833-w (PMC12994241; doi:10.1186/s40900-025-00833-w)
Supplement: Supplementary file 2 — Supplementary Material 2 [file 40900_2025_833_MOESM2_ESM.pdf]

Our Kailo Principles

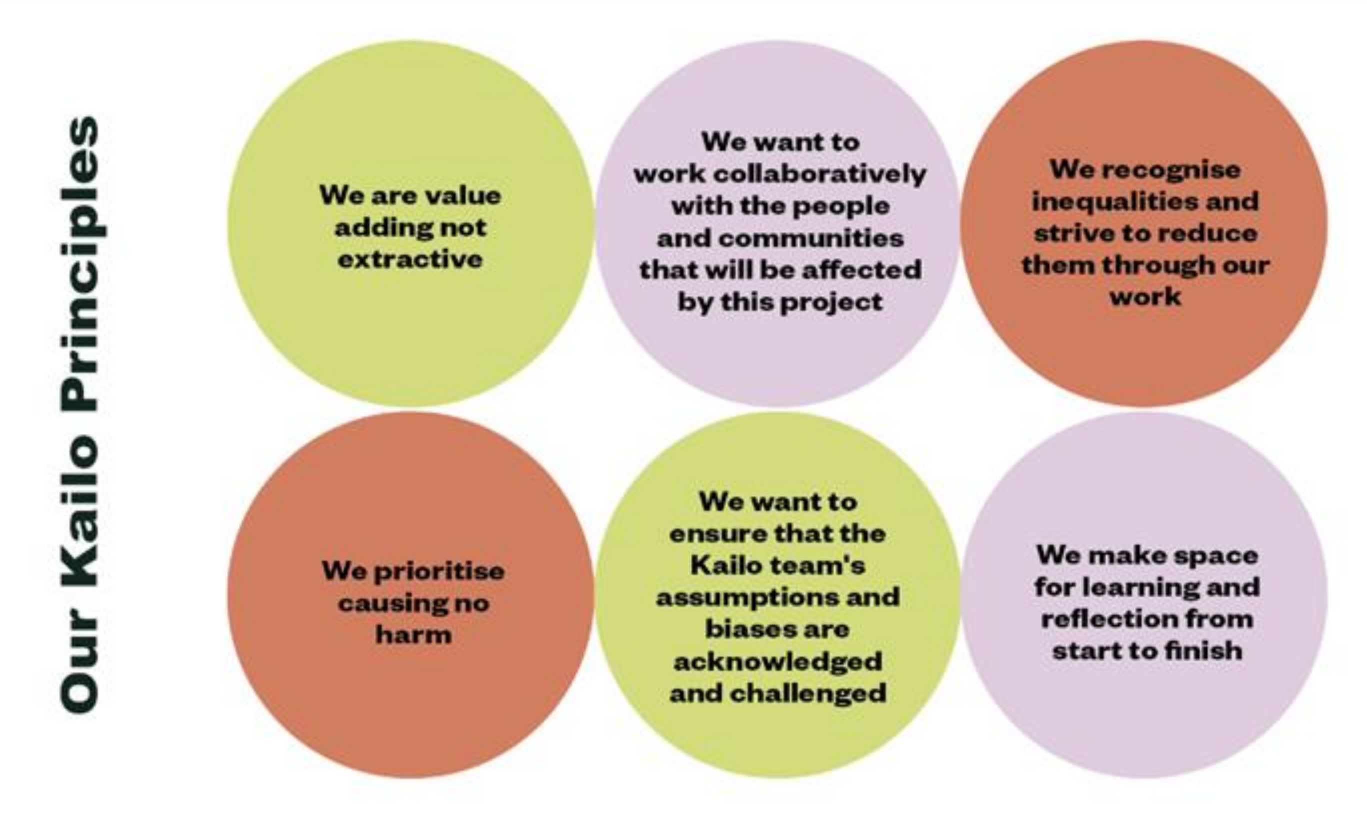

Our Kailo Design Process

The diagram on the right aims to give an overview of the Kailo process, bringing together systems thinking & participatory design processes.

We'll mostly be using it to help our internal Newham team understand and structure the process; but we may to use a more simple version of this to share back with the Small Circle & Community Partners.

The following slides start to give a bit more detail about each step and what we're trying to achieve.

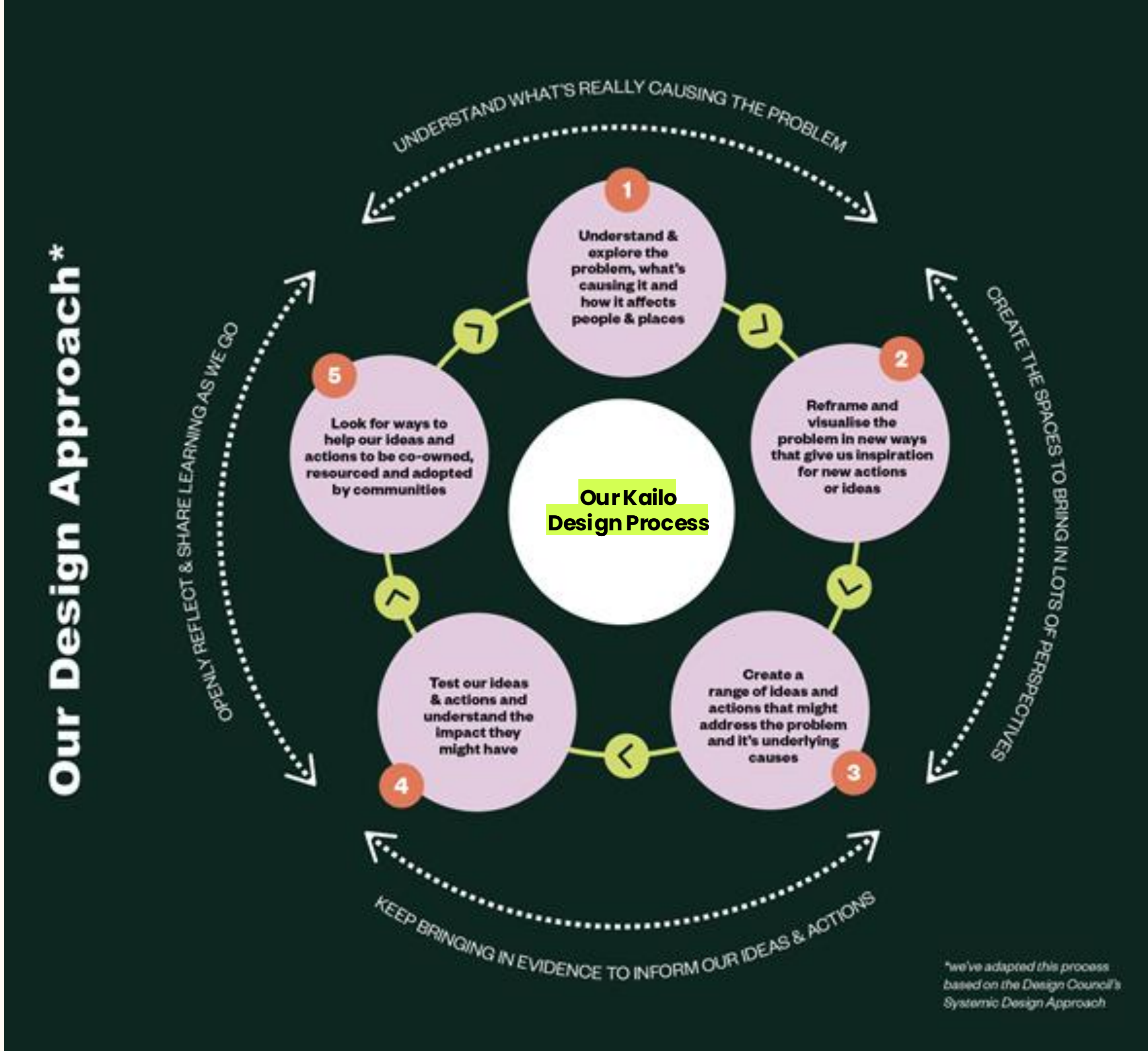

Step 1:
Understand & explore the problem, what's causing it and how it affects

What is this step of the co-design process all about?

Throughout the process we need to make sure that we are trying to deeply understand the problem that we are trying to address and its underlying causes (root causes).

It means trying to understand how the problem is impacting people and communities directly; as well as building an understanding of what ideas and resources might already exist to help address it.

During this part of the process we'll be trying to create a bold vision for what we might want the future to look like; identify areas where new interventions might have the most impact on the most people; and identifying where we might have gaps in our knowledge and understanding that can't just be addressed through the experiences of those in the big or small circles.

We'll be working together to understand the research we have gathered to date, through Kailo and from other sources, and try to make sense of what it means and how it might guide our approach to designing and testing new ideas.

What is this step of the co-design process all about?

Sometimes we need to break out of our current ways of thinking/ acting about a problem or challenge. We need ways and angles to look at the issues and its causes so that we can think differently about how we might begin to address it.

We also need to be cautious not to always think about a problem or challenge in the context of what has or hasn't worked previously. Although this is important, we need to bring in local context, knowledge and nuance to understand what might work here and now.

This means working to reframe the problem and see it in new and different ways. Remapping the wider system in which it sits to see where there might be opportunities to address the problem from a different angle or perspective.

During this part of the process to really want to unpack what is really causing the problem, and visualise the system that underpins it. We want to think about the opportunities where new ideas or actions might have the most impact, and prioritise some specific problem/ opportunity areas that we can start to think creatively about.

Step 2:
Reframe and visualise the problem in new ways that give us inspiration for new actions or ideas

Step 3:
Create a
range of ideas and
actions that might
address the
problem and its
underlying
causes

What is this step of the co-design process all about?

Based on what we know so far, this part of the process is about coming up with a whole range of ideas and actions that might help us address some of the underlying challenges we've uncovered.

It doesn't just have to be new ideas or actions, it might be thinking about how existing services or interventions come together in new ways. It can be a small idea that might make a big change, or it could be something big and bold.

It's really important at this stage to think big, and know that not all ideas or actions will be implementable. We'll work to prioritise these ideas/ actions and think about which we might take forward to test out, but big ideas can help inspire us and think about how the future might look and feel different.

During this part of the process we'll be using lots of creative methods to stretch our imagination and think differently about the problem.

What is this step of the co-design process all about?

During this part of the process we will be prioritising which ideas and actions to take forward and test out with people and communities. We will be developing criteria that will help us narrow down our ideas and actions to the most promising ones, but importantly help us prioritise based on the needs of those that might not be present in our small circle.

We'll want to be testing our ideas quickly. That means finding ways to bring the ideas to life as quickly as possible so we can put them in front of people to get their thoughts, feelings and reactions. This might mean storyboarding how the idea might work, creating a paper mock-up of how something might look, or even role playing how a new scenario or process might work. This process is called prototyping and is a really important part of our co-design process.

We'll be finding creative and engaging ways to take our ideas out into the wider community; and gathering feedback before bringing the feedback together and using it to make our ideas better.

Lastly we'll also want to think about the impact that these ideas might have on people and communities. Without knowing, sometimes the ideas we might suggest might have the potential to negatively impact people and communities. That's why important we test them with a diverse group as possible, and particularly those that could be impacted by the problems we're talking about the most.

Step 4:
Test our ideas & actions and understand the impact they might have

Step 5:
Look for ways to help our ideas and actions to be co-owned, resourced and adopted by communities

What is this step of the co-design process all about?

Throughout the process we'll be bringing our ideas and actions to life through making. Visualising the problem and bringing our ideas to life in ways that people can engage with them is an important part of the process.

It helps us build buy-in from people along the way, gets people excited about our ideas and actions and helps people see how something might work.

This is important when we're thinking about 'who' might own and take some of our ideas forward and actually deliver them in the longer-term.

Through our connections with the Big Circle and others in the community, we'll be working to understand who might want to co-own some of our ideas in the longer term. We'll be working in this part of the process to really understand the time, money and energy that ideas might take to make happen and importantly how we might the type of positive impact they are having on people and communities that are most impacted by the problem.

This part of the process isn't about 'handing' things over, as those who might help our ideas scale will be part of the process and be engaged along the way. It's more about thinking through the practicalities and constraints in helping our ideas really connect into the system and with our communities.

Participant Storyboards

Kailo

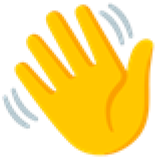

Kailo

Hello!

We're really excited that you might be interested in becoming a Kailo co-designer!

We've created this storyboard to help give you a sense of how the Kailo programme might work and feel. We know you might still be considering whether to join, so we hope it will help you understand more about what to expect and make a decision about taking part.

With our support you'll be working together with other young people, community organisations and our team. We'll be developing and testing out new, creative ideas that can have a positive impact on the health and wellbeing of other young people across Newham.

We hope it will be a fun process to be part of, where you can bring your thoughts and ideas; and learn lots along the way.

Introducing you to Kailo

Kailo

1

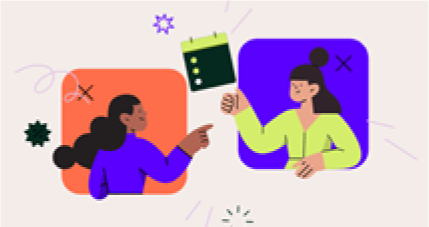

Firstly someone from the Kailo team will give you a ring or a video call to talk to you about Kailo. You'll talk through things like what you'd like to get from the programme, and answer your questions.

2

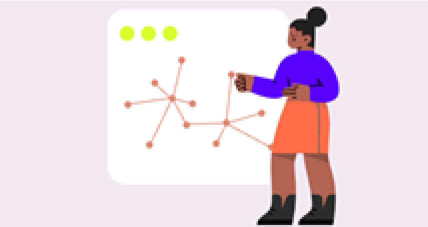

After this chat, you can decide whether you'd like to take part in Kailo or not.

3

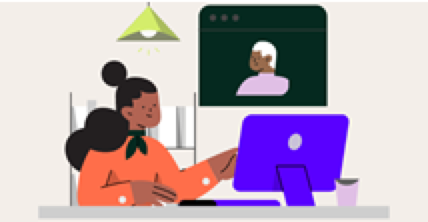

If you'd still like to take part, you'll be invited to a session to meet the other young people that are taking part. You'll have chance to ask more questions, get to know each and share what you'd like to get from taking part.

4

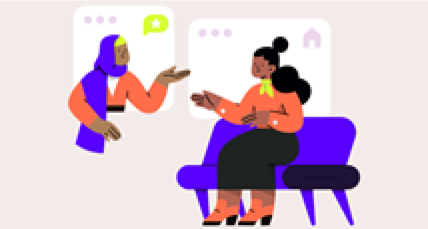

Throughout the whole process you'll be able to chat to any of the Kailo team to ask questions, share ideas and discuss any worries. All you'll need to do is give us a call, a text or an email.

Introducing you to Kailo

Kailo

5

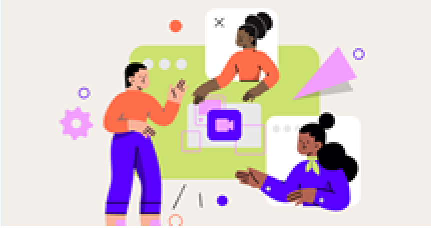

We'll then properly start our work together!

6

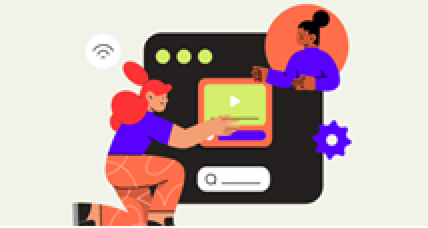

Together with the young people you've met so far, and people from some community organisations, we'll form a 'Small Circle' who you'll work with over the next 6 months.

7

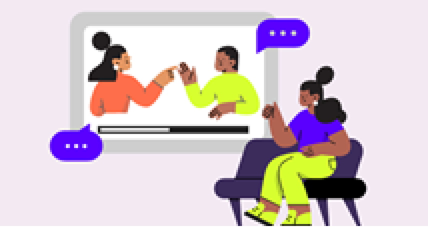

A 'Small Circle' is just what we call the group of up to 12 people (including you) coming together to share and develop creative ideas. Don't worry though we can call ourselves something more fun!

8

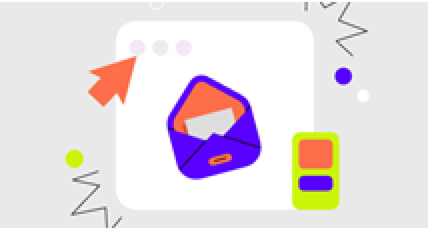

In our first 'Small Circle' session together, we'll decide how we would like to communicate with each other, both during and outside of the sessions.

Introducing you to Kailo

Kailo

9

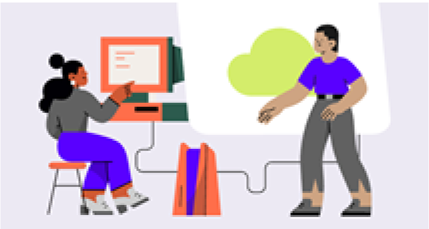

We'll then be regularly coming back every few weeks in person or online where the Kailo team will be helping guide us through a design process, which will include lots of fun and creative activities.

10

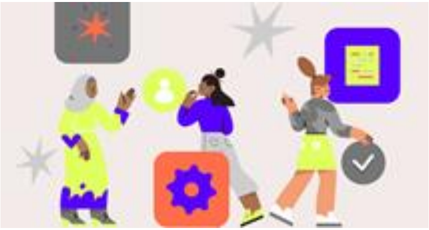

During some of the early Small Circle sessions we'll have chance to better understand some of the issues and challenges that young people might be facing across Newham.

11

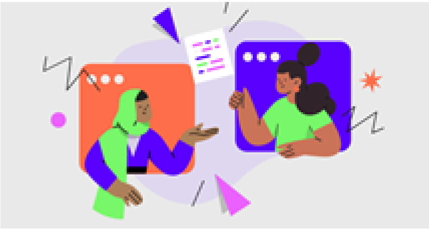

This will be based on research that's been done so far. But you will also have chance to share your experiences, but only if you feel comfortable to do so!

12

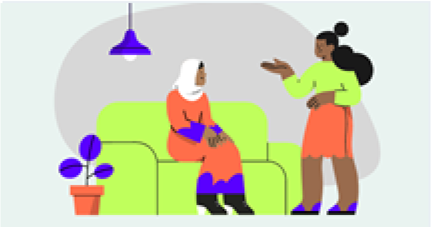

It's really important too, to say that we know that some of the topics we might talk about might be sensitive for you or others. And that it is ok if there are conversations or sessions you'd rather not be involved in.

Introducing you to Kailo

Kailo

13

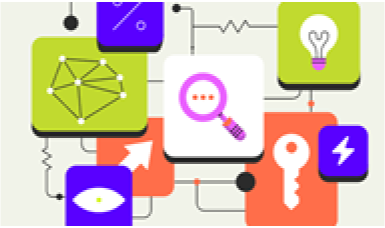

Once we've had chance to explore some of these problems in more detail it'll be time to think about what we might do about them, and what might help!

14

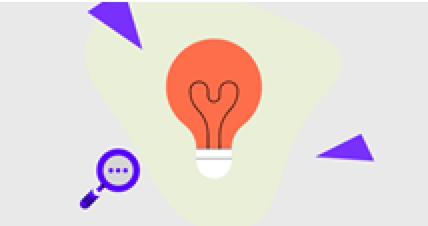

Together we'll be creating lots of new ideas that we can go and test with other young people, their families, carers or wider communities (schools, community/ youth groups, faith groups for example) to see if they'll work or not.

15

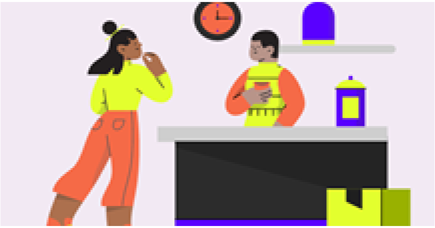

That might mean going out into our communities to get some feedback from other young people, their families or people who work in the area. Don't worry, we'll be doing it together as a team, with lots of support and encouragement.

16

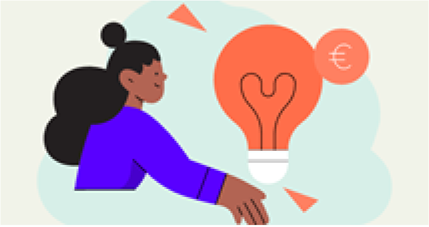

We'll want to think about ideas that we could turn into a reality quickly; and ideas that might take a bit more time and energy to make real.

Introducing you to Kailo

Kailo

17

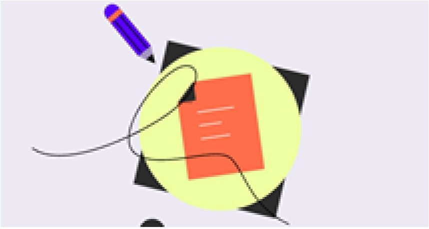

Throughout the whole process, we'll be working together to capture and share the story of our work and what we're learning. That might mean writing blogs or sharing videos as we go.

18

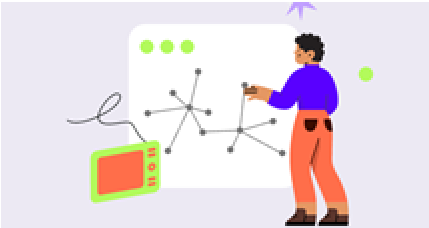

We want others to be able to learn from what we're doing, and think about trying some of our ideas out in their own communities or places.

19

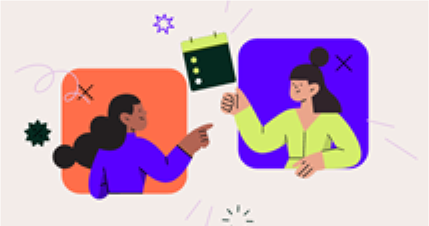

By the end of this part of the programme, we'll have worked together to have developed and tested some brilliant and creative ideas.

20

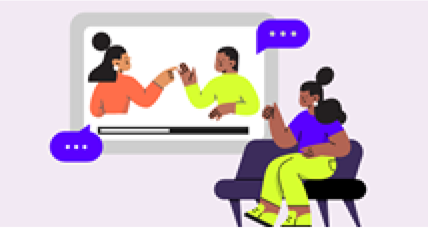

And we'll have engaged lots of different people along the way who can hopefully help turn our ideas into a reality and benefit young people like you across Newham!

What happens next?

Kailo

We hope this storyboard has given you more of a sense of what it might feel like to become a Kailo co-designer!

We know you'll still have lots of questions, and you might still be deciding if this project is something you want to be part of.

We also imagine you'll still have questions. All you need to do is email us with your questions; mention them in our introduction call; or bring them along to one of our first meetings.

We hope to see you soon!

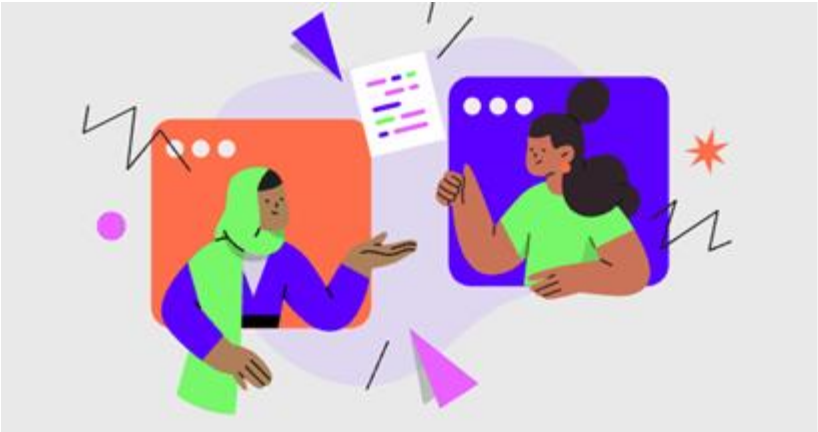

Small Circle Overview

Small Circle Session:	Introductory 1-2-1 calls	Prep Session #2 - Young People	Prep Session #3 - Community Partners		Session #1	Session #2
Tentative dates:	*From 21/08*	*1 week before 1st Small Circle*	*week before 1st Small Circle*	***SMALL CIRCLE PROCESS BEGINS***	*W/C 11th or 18th September*	*W/C 2nd October*
Session title	**N/A**	**Getting to know each other**	**Reflecting on power & setting the ground rules**		**Understanding one another & the Kailo process**	**Reflecting on our Opportunity Areas**
Session aims:	**Aim:** To get to know the young people involved in the process, answer their questions and better understand their needs so we can make sessions inclusive.	**Aim:** To bring Young People together to get to know one another, agree how they'd like to work together, reflect on power dynamics in the group and get them comfortable with the Kailo co-design process.	**Aim:** To bring our community partners together to reflect on their role in the Small Circle, power dynamics and discuss any ground rules we would like to set before we come together as a whole Small Circle.		**Aim:** To bring the whole group together to get to know one another, understand the Kailo design process and introduce our Opportunity Areas and learning so far.	**Aim:** To further deep dive into our Opportunity Areas and begin to lay the foundations for our Systems thinking work and process in the coming sessions.

Small Circle Overview

Small Circle Session:	Session #3	Session #4	Session #5	Session #6	Session #7
Tentative dates:	*W/C 16th October*	*W/C 30th October*	*W/C 13th November*	*W/C 27th November*	*W/C 11th December*
Session title	**Deeper understanding of our opportunity areas**	**Mapping and understanding the system**	**Spotting opportunities for change**	**Pause & reflect**	**Creating a vision**
Session aims:	**Aims:** To better understand the Opportunity Areas in more detail, and understand the trends that may be affecting the opportunity area and the differences in our knowledge and experience relating to it.	**Aims:** Better understand the connections within the system and surface mental models relating to them. Find patterns and connections, and begin to create a systems map identifying leverage points.	**Aims:** Better understand the connections and leverage points relating to the opportunity area and begin ideation for co-design.	**Aim:** To take a pause in the process, celebrate our work so far and reflect on what we've learnt. We'll start to think about the next part of the process and what we think we'd like to achieve.	**Aim:** To begin to think about a shared vision for the future. Together we'll be looking to develop an inspiring vision for the future of mental health for YP in Newham, and begin to think about ideas and ways we can get others excited about our vision.

Small Circle Overview

Small Circle Session:	Session #8	Session #9	Session #10	Session #11	Session #12	Session #13
Tentativ e dates:	*W/C 2nd or 15th January*	*W/C 29th January*	*W/C 12th February*	*W/C 27th February*	*W/C 11th March*	*W/C 25th March*
Session title	**Developing ideas**	**Prototyping our ideas**	**Testing & feedback**	**Sharing our ideas (a Kailo Ideas Marketplace)**	**Refining our ideas & interventions**	**Wrap up & celebrations**
Session aims:	**Aim:** To develop a range of creative ideas that are a mixture of pragmatic solutions that can be implemented in the short term, and bigger ideas that we need to test with others.	**Aim:** Use prototyping techniques and methods to bring the group's ideas to life quickly so they can tested. Here we should focus on testing our 'biggest assumptions'.	**Aim:** Developing ways of getting/ gathering feedback on our ideas from a range of people/ peers. This might include other Young People or with systems influencers who can help make ideas reality.	**Aim:** To run a 'Kailo' marketplace where the group invites people & peers connected or interested in Kailo to share our ideas, get feedback and gain buy-in from systems influencers.	**Aim:** Bringing feedback together to refine and tweak our ideas. We'll be focusing on thinking about what the next steps might be to take these ideas forward and our 'ask' of system influencers.	**Aim:** To celebrate our work together and what we've achieved with friends, family and peers!

What might be happening between sessions?

What might be happening between sessions?

Between sessions there will be work required to move things forward, and prepare for the next session.

The following slides look at the ‘common’ acitivities that might be happening between each session.

We will then agree specific activities between sessions and who is doing which as we go.

What might be happening between sessions?

Core facilitation team
To be agreed but may include:<br><br>● Write-up of session - notes to be captured as we go<br>● Synthesis of participant feedback (gathered at the end of sessions)<br>● Tweak/ revise agenda for next session based on what we've learnt<br>● Comms between sessions with participants (i.e. logisitical changes)<br>● Check-ins with SC participants as needed<br>● Feedback to Kailo programme<br>● Coordinate with other Kailo stakeholders (evidence, systems-mapping, Devon counterparts)

What might be happening between sessions?

Small Circle Participants
Where appropriate and where SC participants are interested they may continue to develop the work in a number of ways:<br><br>● Background reading & desk research<br>● Gathering feedback and thoughts from peers, community links etc.<br>● Testing ideas and gathering feedback<br>● Supporting to write and develop programme comms - sharing weeknotes etc.<br>● Co-present show and tell sessions with core team

What might be happening between sessions?

Community Partners
Where appropriate and where Community Partners have interest/ time:<br><br>● Helping SC make wider connections into community/ with peers<br>● Feedback and iteration of SC session agendas<br>● Briefings/ debriefings with core Newham team<br>● Testing of ideas and gathering of feedback outside of sessions<br>● Attend show and tell sessions

What might be happening between sessions?

Community Designer/ Researcher
Where appropriate and where Community Partners have interest/ time:<br><br>● Helping SC make wider connections into community/ with peers<br>● Feedback and iteration of SC session agendas<br>● Briefings/ debriefings with core Newham team<br>● Testing of ideas and gathering of feedback outside of sessions<br>● Attend show and tell sessions

What might be happening between sessions?

Big Circle
Playback of learning and progress via:<br><br>● ‘Small circle notes’<br>● Attend show and tell sessions - frequency to be agreed<br>● Marketplace testing event - to be agreed if this a suitable format<br>● Ad hoc feedback on emergent ideas

Small Circle Session Agendas

Kailo

Prep Sessions 2 & 3 (Young People & Community Partners)

Kailo

Group Prep session 2:
Young People
Aims

Understanding Kailo:
- Begin to build a bit more of an understand of Kailo and what we're trying to achieve together

Building relationships:
- Start to build relationships between young people in the group and help make them feel comfortable with one another

Understand skills, strengths and comfort zone:
- Outline what the young people can expect in the Small Circle sessions and get them to reflect on what they are comfortable with; where their 'stretch zone' is, and what might make them feel uncomfortable, how they can recognise this, and what support they might need.
- Get young people to reflect on their expertise, skills and strengths they are bringing to the group

Understanding needs:
- Work with young people to understand their needs and use to develop support strategies for later sessions a needed
- Consider how we support psychological safety when discussing sensitive issues

Timing	Activity	Aim(s)	Facilitation notes	Content to create/ materials	Facilitator
10 minutes<br><br>***5:00***	**Welcomes**	Everyone arrives and gets settled and fills in their name badge	To think about refreshments/ food etc. and when it should feed into the agenda	N/A	N/A
10 minutes<br><br>***5:10***	**Ice breaker**	A fun activity to get to know one another	(to decide on the day)<br>2 Truths & A Lie<br>The Name Game	N/A	Josh
15 minutes<br><br>***5:20***	**Introductions**	Welcome everyone to the session and briefly chat through the plan for our time together	Facilitator gives a really quick overview of what will be covered in the session and what to expect; and answers any questions the group might have.<br><br>Also to cover consent forms & reimbursement timelines for the group.<br><br>Everyone has an opportunity to introduce themselves, and one thing that they are most looking forward to today.<br><br>Facilitator also makes clear to the group that it is ok to not be involved in all the conversations and activities they they don't feel comfortable to do so, and that they just need to mention to one of the facilitators in the room.	Name badges & pens<br><br>Expenses Form<br><br>Consent Forms & Info Sheets	Josh
15 minutes<br><br>***5:35***	**Check-in**	Use check-in 'postcards' to share how we're showing up today	A range of different colours are printed off and spread across the table. Invite everyone to pick a colour that best represents how they are feeling coming into the session today. Where comfortable people are asked to share why they've picked that colour and what it represents for them.<br><br>Ideally, the facilitator would go first in sharing.	Print-offs with various colours	Tanya

Timing	Activity	Aim(s)	Facilitation notes	Content to create/ materials	Facilitat or
15 minutes<br><br>***5:50***	**How we want to work together**	Agreeing how we want to work together throughout the process	Facilitator introduces the idea that we'll be working together as one team for a number of months. Both as this group but also with the wider small circle.<br><br>And that we want to create some words/ phrases that help us describe how we want to work together, and how we want to feel throughout the process.<br><br>Each person is invited to have a look at the words on the table (i.e. respectful, listened to, fun) and pick 3 or 4 that are important to them OR write down their own.<br><br>Each person takes 3 dot stickers and puts dots on the ones which are most important to them - and the group is invited to discuss which they've picked and why in small groups.	Post-its and example words to dot vote on, dot vote stickers	Sophie
30 minutes<br><br>***6:05***	**BREAK**	Take a longer break	This should include food etc. but we may want to make the start longer upfront to account for eating if an early evening session.	N/A	
10 minutes<br><br>***6:35***	**Overview of Kailo**	Provide an overview of what Kailo is, what we're trying to achieve	Facilitator gives a really quick overview of Kailo, reminding the group what we're trying to achieve and what to expect from some of the Small Circle sessions.<br><br>Using the example that Tanya & Amelia have created to explain the process to young people.	Simple overview of Kailo process and outcomes to talk to	Tanya

Timing	Activity	Aim(s)	Facilitation notes	Content to create/ materials	Facilitator
15 minutes<br><br>***6:45***	**Identity Shield activity**	Think about our individual strengths and skills we might be bringing to the process	There is a table (or space) in the middle of the room with lots of craft/ drawing/ writing materials.<br><br>Everyone is invited to pick up a 'Identity Shield Template' and the facilitator introduces the activity by saying that everyone in the group has strengths and experiences (skills, experiences etc.) to add the process.<br><br>Everyone is then asked to spend some time either in small groups on individually creating their 'visual identity shield' using whatever materials they feel most comfortable with (write, draw etc).<br><br>The shield template has question prompts to it: What I am good at? What do I enjoy doing? What do I not enjoy? What else what I like others to know about me?<br><br>Provide notice that the doc will be put on the wall for everyone to see<br><br>Everyone is then invited to pin their template to the wall, and everyone can have a read during the break.	Templates for identity shields and completed example	Mez
10 minutes<br><br>***7:05***	**BREAK**	A quick and shorter break	This should include food etc. but we may want to make the start longer upfront to account for eating if an early evening session.	N/A	TBC
20 minutes<br><br>***7:15***	**My comfort & stretch zone**	To get the group to think about which bits of the process are in their 'comfort' zone and which are in their 'stretch zone'	The 'Kailo small circle storyboard' is printed out and pinned to the wall. Everyone takes a bit of time to have a look over it.<br><br>Using post-it notes everyone is asked to think individually a) which bits of this make you feel excited? b) which bits of this make you feel worried and anxious at all?<br><br>Small groups (3-4) then come together to talk through their thoughts, and think about what do we need to think about to make sure that everyone in the group remains in their comfort zone, and what we do if we ever feel uncomfortable.	Print offs of Kailo storyboard<br><br>Stickers and post-it notes	Sophie

Timing	Activity	Aim(s)	Facilitation notes	Content to create/ materials	Facilitator
5 minutes<br>***7:30***	**Check-out**	Use check-in postcards for people to share how they are leaving the session and how they are feeling	Colours are spread out on table/ floor and everyone is invited to pick a postcard that best represents how they are feeling at the end of the session. Anyone comfortable to is invited to share why they've picked their colour.	Postcards with various colours on	Josh
N/A	**Session close**	Facilitators debrief on the Small Circle Session, agree actions etc.	Thank everyone and mention next steps.	N/A	TBC

Group Prep session 3: Community Partners

Aims

Understanding Kailo:

- Build more of an understanding of Kailo and what we're trying to achieve together

Understanding skills, experience, knowledge & networks

- To begin to map and understand the joint skills and experience that we have in the room; and how we might use it throughout the process

How we want to work together

- To think about how we want to work together as a Small Circle Group - think about what values, principles we might want hold ourselves to account to
- Think about how we want to communicate with one another, as well as engage people beyond the Small Circle group

Power dynamics:

- Think through power dynamics that might emerge during the Small Circle process, and what things we might need to put in place to make sure the balance is right between everyone in the group

Timing	Activity	Aim(s)	Facilitator notes	Content to create/ materials	Facilitator
10-10:05 (5 minutes)	**Welcome & Introductions**	Quick introductions and session overview	Someone to quickly run through high-level session agenda and round robin style introductions	Name badges	Mez
10:05-10:15 (10 minutes)	**Check-in**	Use check-in postcards to share how we're showing up today & everyone to share one thing they'd like to get from the session	Everyone is asked to look at the different colours in front of them & select one that best represents how they are showing up today.<br><br>Each person takes it in turns to share back:<br>1) why they've picked the post card<br>2) one thing they would like to get out of the session today (facilitator notes down all the things to get from the session so we can come back to them at the end)	Postcards with different colours	Tanya
10:15-10:25 (10 minutes)	**Overview of Kailo**	Give an overview of Kailo, objectives and talk through/ discuss the overall process in relation to Small Circles	Facilitator gives a quick overview of Kailo (5-10 minutes)	Print off of Kailo Overview of kailo aims and process	Niran
10:25-10:45 (20 minutes)	**Hopes & fears**	Surfacing and addressing any hopes and fears that we might have about the process<br><br>How do we make sure we achieve our hopes for Kailo, and avoid our fears?<br><br>What are our individual and collective roles in this?	We then move straight into a hopes and fears exercise. This will be the chance to share any concerns or outstanding questions that we have.<br><br>Everyone is given 2 coloured post-it notes. We are then given 5 minutes to individually write down hopes on one colour and fears on the other.<br><br>Everyone is then invited to add their post-its to the wall and talk to them as they stick them up.	Post it notes (2 colours one for hopes and one for fears)	Sophie

Timing	Activity	Aim(s)	Facilitator notes	Content to create/ materials	Facilitat or
10:45-11 (15 minutes)	**Break**	Short break for people to get some air, walk or take a comfort break	N/A	Reflection wall for anyone to note feedback throughout the session	
11-11:10 (10 minutes)	**Introducing the OAs**	Introduction to the OAs and discussion on any questions, concerns, sensitivities we may need to account for	The Opportunity area is printed out and everyone is invited to move into small groups (where appropriate), read over the Opportunity Area and think about:<br>- What sensitivities should we account for in regards to the OA?<br>- What should we be aware/ mindful of in relation to the OA?<br>- How should we account for these sensitivities in the process?	Opportunity area overview to print off	Mez
11:10-11:30 (20 minutes)	**Mapping our skills, knowledge & networks**	Mapping our skills, knowledge and relationships in relationship to the OAs, and discuss how we imagine this showing up in the process	The facilitator then says that we want to make the most of everyone's skills, experience & networks throughout the process. And that we want to spend a bit of time mapping this out together.<br><br>Everyone is given different coloured post-it notes. One colour each to represent:<br>- Skills/ expertise<br>- Relationships/ people<br><br>Everyone then individually writes down what they can/ would like to bring to the process & as facilitators we start to map connections/ similarities.	Post-its, pens & paper	Robbie
11:30-11:50 (20 minutes)	**Power Dynamics**	Understanding the power that we bring into a space, and how we acknowledge and be mindful of it	Individually or in small groups everyone is invited to reflect on the the question:<br>***'What does power mean to me, and how do I think my power (consciously or subconsciously) will show in this process?'***<br>In small groups everyone is then invited to share their own reflections, and think about what we might put in place to make sure the power dynamic between young people and adults in the group is balanced.	Power question printed out and post-its, pens	Sophie

Timing	Activity	Aim(s)	Facilitator notes	Content to create/ materials	Facilitator
11:50-12:05<br><br>*(15 minutes)*	**How do we want to work together**	Agreeing how we want to work together throughout the process - what values, principles or things are important to keep hold of	Facilitator introduces the idea that we'll be working together as one team for a number of months. Both as this group but also with the wider small circle.<br><br>And that we want to create some words/ phrases that help us describe how we want to work together, and how we want to feel throughout the process.<br><br>Each person is invited to have a look at the words on the table (i.e. respectful, listened to, fun) and pick 3 or 4 that are important to them OR write down their own.<br><br>Each person takes 3 dot stickers and puts dots on the ones which are most important to them - and the group is invited to discuss which they've picked and why in small groups.	Post-its and example post-its to dot vote on	Robbie
12:05-12:20 (15 minutes)	**Safeguarding, confidentiality & risks**	To cover anything we've missed around safeguarding and confidentiality	Additional time to make sure we've covered any risks or concerns that we have. Thinking through any scenarios that we may want to plan or account for.<br><br>To print and use our current risk log as a starting point if needed.	Risk log, post-its, pens	Niran
12:20-12:25 (5 minutes)	**Check-out**	Quick check-out using our check-in postcards to get a sense of how people are feeling about the process	Everyone is asked to look at the postcards or image in front of them & select one that best represents how they are feeling at the end of the session & how they are checking out.	Check-in/ out postcards	Tanya
N/A	**Session close**	Facilitators debrief on the Small Circle Session, agree actions etc.	Thank everyone and mention next steps.	N/A	

Small Circle Sessions 1-13

Kailo

Small Circle #1

Kailo

Small Circle 1

Aims

- Introduce the adults and the YP in one space
- Get to know each other better - our interests and what's drawn us to Kailo
- Forming a sense of a team amongst the group - and how we want to work together
- Understanding more about Kailo and the underlying social determinants of health
- Discussing safeguarding processes

Timing	Activity	Aim(s)	Facilitator notes	Content to create/ materials	Facilitator
10 minutes	**Arrive & welcome**	Everyone joins and begins to settle	Possibly quiet music playing in the background to welcome everyone and drinks etc.<br><br>Everyone is invited to fill in a name badge when they arrive.	Drinks, snacks etc.	TBC
5 minutes	**Aims & expectations of the session**	Outline of the agenda and what we're going to be covering today	Facilitator welcomes everyone and reminds everyone why we are here and what we're hoping to achieve in our session today.	Agenda/ aims printed on the wall	TBC
10 minutes	**Ice breaker**	A fun icebreaker activity to continue building relationships within the group	To be shaped and agreed with the team	TBC	Josh to lead/ Tanya to input on ideas
10 minutes	**Check-in**	Using post card format for people to share how they are joining the session today and how they are feeling	A range of different colours are printed off and spread across the table. Everyone is invited to pick a colour that best represents how they feeling after the ice breaker & starting the kailo process. Where comfortable to, people are invited to say why they have picked the colour they have.	Print offs with different colours	Sophie

Timing	Activity	Aim(s)	Facilitator notes	Content to create/ materials	Facilitator
	**Recapping on the Kailo approach & aims**	To give a quick reminder of what Kailo is and what it's trying to achieve	A quick way of introducing Kailo again and what we're trying to achieve through the process together.<br><br>This should be high-level and give everyone a sense of what we might be able to achieve together.	Slide/ print offs summarising Kailo process & aims, post-its to capture thoughts	TBC
10 minutes	**Social determinants of health**	To begin to 'lightly' introduce the idea of social determinants of health	To give an accessible and easy to understand definition of the social determinants of health<br><br>Split the group in two and ask them to work together to come up with as many SDH as they can - the more specific the better.<br><br>Each team takes it in turns to give one example and they get a point for every valid answer. All examples are written down to create a diagram showing the SDH that we came up with as a group.<br><br>Facilitator to then make the point that our work together could look to explore and understand the social determinants of health.	Social determinants definition & paper for recording example and points	Tanya
30 minutes	**BREAK FOR FOOD**	Everyone has chance to eat now that some introductions have been done	As needed use some of the time to regroup on activities/ plans	N/A	N/A
30 minutes	**Gallery of Us**	An help the group get to know one another a bit more, mixing the group of adults and young people together	To ask the group to get into pairs with someone they don't know. Each person takes it in turns to draw one another with their eyes closed.<br><br>They then 'swap their portraits back', and they then take it turns to ask them 5 questions to get to know one another more. Questions are printed on the page already - what do you enjoy doing outside of work/ school, your favourite food/ music etc.<br><br>Portraits are then pinned up onto the wall.	Template, pens and other craft materials	Josh

Timing	Activity	Aim(s)	Facilitator notes	Content to create/ materials	Facilitator
30 minutes	**Our Ways of Working**	Reflect back on our shared agreement for how we'll work together	Print-offs of our 'group ways of working pledge' that's been created are pinned to the wall or on the table.<br><br>The facilitator shares that this has been created based what both groups shared in sessions before, and we've brought them together as one set of words/ principles.<br><br>The group is invited to take a look and share their reflections, and think about how they will play a role in making sure that we can all help make these a reality.<br><br>Each group member takes it in turns to add their name to the pledge of working together.	Print off of synthesised ground rules<br><br>Flipchart & pens to create shared manifesto	Sophie
10 minutes	**Check-out**	Quick check-out using our check-in postcards to get a sense of how people are feeling about the process	A range of different colours are printed off and spread across the table. Invite everyone to pick a colour that best represents how they are feeling leaving the session today. Where comfortable people are asked to share why they've picked that colour and what it represents for them.	Check-in/ out postcards	TBC
N/A	**Session close**	Facilitators debrief on the Small Circle Session, agree actions etc.	Facilitator thanks group and does any housekeeping for next session	Facilitation notes & templates	TBC

Small Circle #2

Kailo

Small Circle 2
Aims

- Continue building relationships and trust within the group
- Start to understand more about the opportunity area and what it means to the group
- Understand our limitations in relation to the opportunity area - including where our knowledge gaps might be and others we might want to get involved
- To introduce systems modelling and the team facilitating it

Timing	Activity	Aim(s)	Facilitator notes	Content to create/ materials	Facilitator
10 minutes	**Arrive & welcome**	Everyone joins and begins to settle	Possibly quiet music playing in the background to welcome everyone and drinks etc.<br><br>Everyone is invited to fill in a name badge when they arrive.	Drinks, snacks etc.	TBC
5 minutes	**Aims & expectations of the session**	Outline of the agenda and what we're going to be covering today	Facilitator welcomes everyone and reminds everyone why we are here and what we're hoping to achieve in our session today.	Agenda/ aims printed on the wall	TBC
10 minutes	**Check-in**	Using post card format for people to share how they are joining the session today and how they are feeling	A range of different colours are printed off and spread across the table. Everyone is invited to pick a colour that best represents how they feeling after the ice breaker & starting the kailo process. Where comfortable to, people are invited to say why they have picked the colour they have.	Post-cards with colours for check-in	TBC
30 minutes	**FOOD BREAK**	Food			
5 minutes	**OA introduction**	To provide a recap of the OA area and 'the journey to get to this point'	Short summary of the opportunity area and more detail on the work and research that has been done to get us to this point and how they'll been selected.	Print off of the OAs	TBC
20 minutes	**OA deeper discovery**	To begin to allow the group to more deeply reflect on the OA	Initial introduction and small group discussions around the HMW question. What does this OA mean to everyone, what feels important, what feels different to their experience and the experience of others?	Post-its to capture reflections	TBC

Timing	Activity	Aim(s)	Facilitation notes	Content to create/ materials	Facilitator
15 minutes	**Break**	Short comfort break and a chance for people to share feedback/ reflections so far			
20 minutes	**Mapping our limitations**	To spend time spotting where our gaps in knowledge, relationships and experiences may be in this OA. Map out how we might address these gaps and the steps we should be taking.	Each part of the opportunity area is printed out and positioned across the room.<br><br>Break the group into small groups of 3-4, and ask them to each stand around one of parts of the opportunity area.<br><br>With post-it notes each group then start to think about any questions relating to the opportunity area that we may not be able to answer, and begins to think about who else we might need to include.	Mapping template with prompts	TBC
20 minutes	**Introduction to systems thinking**	An overview and outline of the next 3-4 sessions from the GBM team, and an opportunity for the group to ask questions.	Activity TBC - to discuss with Megan and team	Content for introduction to systems thinking	TBC
10 minutes	**Check-out**	Quick check-out using our check-in postcards to get a sense of how people are feeling about the process	A range of different colours are printed off and spread across the table. Invite everyone to pick a colour that best represents how they are feeling leaving the session today. Where comfortable people are asked to share why they've picked that colour and what it represents for them.	Check-in/ out postcards	TBC

Small Circle #3

Kailo

Small Circle 3 (systems session 1):
Aims

(1) Build shared understanding of **“trends over time”** related to the opportunity area.

(1) Identify the factors that contribute to the trends related to the opportunity areas

(1) Begin to **build connections** between the factors identified

(1) Discuss, expand, negotiate **differences in experience and knowledge** related to the opportunity area

Small Circle 3
Agenda

Date: XYZ

Time: ~ 2.5 hours

Location: TBC

Facilitators (& roles):

1) Site facilitators(s)

2) System specialist

3) Young researcher(s)/ Peer or community facilitator

4) Production coordinator/note taker

** To note every session should have a briefing and debriefing for facilitators*

Timing	Activity	Aim(s)	Content to create/ materials	Facilitators
10 minutes	**Check-in and introductions**	Recap of group agreement from previous sessions.<br><br>Reminder of rules and expectations.<br><br>Opportunity to flag anything important at the beginning of the session.	Information from previous session.<br><br>Space for writing reminders of group expectations (either paper, whiteboard, screen, etc).	Site facilitator
5 minutes	**Aims and expectations**	Review agenda for the day, open the space for any (quick clarifying) question.	Agenda written somewhere so people can check in	System specialist
15 minutes	**Session 2 recap**	Remind group about learning surrounding the opportunity area from session 2 (and 1?)<br><br>Site lead reflections shared<br><br>Allow space for additions from small circle	Inputs from previous session	Site facilitator, supported by young researcher/community facilitator
10 minutes	**Break :)**		snacks !	

Small Circle 3
Agenda

Date: XYZ

Time: ~ 2.5 hours

Location: TBC

Facilitators (& roles):

1) Site facilitators(s)

2) System specialist

3) Young researcher(s)/ Peer or community facilitator

4) Production coordinator/note taker

** To note every session should have a briefing and debriefing for facilitators*

Timing	Activity	Aim(s)	Content to create/ materials	Facilitators
25 minutes	**Trends over time**	Identify/uncover historical trends related to the opportunity area.<br><br>Reflect on how things have changed (in order to inform what makes something improve or get worse)<br><br>(Possibly) theme the trends and agree upon what is more important to investigate	Paper and pens/markers, tape/adhesive for walls	Site facilitator + System specialist (supported by young researcher(s) and community facilitator)
25 minutes	**Variable elicitation**	Identify factors related to the opportunity area, ideally specifically related to the trends over time<br><br>Develop "word bank" for future exercises	Paper and pens/markers, tape/adhesive for walls	Site facilitator + System specialist (supported by young researcher(s) and community facilitator)
30 minutes	**Connection circles**	Learn about how to identify relationships between factors<br><br>Begin to think about how factors are related<br><br>(Ideally) start to identify some loops in the system	Large sheets of paper, pens/markers	Site facilitator + System specialist (supported by young researcher(s) and community facilitator)
10 minutes	**Closing and next steps**	Reconnect, wind-down, have idea of next steps		Site facilitator + System specialist

Small Circle #4

Kailo

Small Circle 4 (systems session 2):
Aims

(1) **Explore connections** within the system to identify the underlying drivers of the challenges related to the opportunity area(s)

(1) **Surface various mental models** (ways of knowing/lived experiences) from different members of the small circle

(1) Introduce causal loop diagrams, **thinking in feedback patterns**

(1) **Develop causal loop diagrams** that can be used to consider how to **impact change** in the system

Small Circle 4
Agenda

Date: November 2nd, 2023

Time: 5 - 7.30pm

Location: Fight For Peace

Facilitators (& roles):

1) Sophie - session recap and table facilitation

2) Megan - system activity lead

3) Tanya - session recap and table facilitation

4) Josh - convene, close, and coordinate room

5) Mez - table facilitation

6) Niran and Tamsin - note taking

Timing	Activity	Aim(s)	Content to create/ materials	Facilitators
5 - 5.15pm	**Arrivals**	Select a blob on the blog tree<br><br>Collective playlist	Blob tree	
5.15 - 5.25pm	**Check-in and introductions**	Recap of group agreement from previous sessions.<br><br>Reminder of rules and expectations.<br><br>Opportunity to flag anything important–box for no-go topics	Space for writing reminders of group expectations (either paper, whiteboard, screen, etc)--or print out<br><br>Blog tree	Josh
5.25 - 5.30pm	**Icebreaker**	Get group moving		Young Person (Josh to set up via whatsapp group beforehand)
5. 30 - 5.35pm	**Aims and expectations**	Review agenda for the day, open the space for any (quick clarifying) question.	Agenda written on a whiteboard	Megan
5.35 - 5.50pm	**Session 3 recap**	Remind group about learning surrounding the opportunity area from session 3<br><br>System specialist reflections shared<br><br>Discussion with other people in the room	Inputs from previous session (typed up)<br><br>Drawn trend over time<br><br>Blank	Site facilitator, supported by young researcher/community facilitator

Small Circle 4
Agenda

Date: November 2nd, 2023

Time: 5 - 7.30pm

Location: Fight For Peace

Facilitators (& roles):

1) Sophie - session recap and table facilitation

2) Megan - system activity lead

3) Tanya - session recap and table facilitation

4) Josh - convene, close, and coordinate room

5) Mez - table facilitation

6) Niran and Tamsin - note taking

Timing	Activity	Aim(s)	Content to create/ materials	Facilitators
5.50 - 5.55 pmin	**Break / energiser - rearrange tables**			Megan (or anyone else)
5.55 - 6.10pm	**Feedback / systems mapping learning**	Introduction to feedback thinking<br><br>Preparing everyone for the CLD exercise, group activity on the whiteboard	White board/paper/slides	Megan
6.10 - 6.55pm	**Causal loop diagrams**	Develop causal loop diagrams related to the opportunity area<br><br>Explore connections between factors identified as related to the OAs	Large sheets of paper, markers/pens, white boards if possible	Megan (floating to help)<br><br>Mez, Tanya, Sophie as table facilitators
6.55 - 7.05pm	**Reflections and next steps**	Space to close out and reflect as a large group about the exercises		Megan
30 min	**Food and check out**	Eat, fill in expense forms, check out activity with the blobs / 1:1 conversations	Blob tree and expense forms	Full team
