# Supplementary material for: An adaptive, youth-centred co-design methodology: place-based co-design centring youth and community participation
Source: Res Involv Engagem. 2026 Jan 24;12:33. doi: 10.1186/s40900-025-00833-w (PMC12994241; doi:10.1186/s40900-025-00833-w)
Supplement: Supplementary file 3 — Supplementary Material 3 [file 40900_2025_833_MOESM3_ESM.pdf]

Kailo Deeper Discovery Co-Design Teams: Info for Young People

Hey!

You've been invited, or expressed an interest in working with the Kailo team to think about how we could improve young people's mental health and wellbeing in [place].

If you are interested, please read this information sheet carefully before deciding whether you would like to take part. Got questions? Contact: [Include Primary Contact/Kailo Coordinator]

What is Kailo?

Kailo is a research and design project where young people and communities work together to understand and improve things that affect young people's mental health and wellbeing.

We have recently spoken to lots of young people and adults in [place] and we have identified some broad themes that need to be changed or improved.

These are: [to be added below]

1.
2.

Why do we want to talk to you?

We now want to work with young people, and other people in the community with different experiences, perspectives and ideas, to start thinking about what this could look like in practice. We want to hear your ideas and make sure they reach decision-makers who can help make those changes happen.

We want to work with young people who are:

- Between the ages of [add age brackets]
- Living in [place]
- Passionate about improving the experiences of young people in relation to the identified themes:
  - [add broad themes identified from early discovery]
  - [add broad themes identified from early discovery]

We are particularly keen to work with young people who often feel like their voices are underrepresented, for example:

- [add under-represented groups identified in early discovery]
- [add under-represented groups identified in early discovery]
- [add under-represented groups identified in early discovery]

What would I be signing up to?

<u>Key Activities:</u>

- Attend group sessions to explore ways to improve young people's mental health and wellbeing.
- Share your experiences and problem-solve with the team to develop solutions.
- Help raise awareness and connect the work to the community.
- Optional individual or small group tasks between sessions (voluntary).

If you don't want to answer particular questions, talk about particular topics that is totally fine. There will always be space to go and someone to support you if you want to take time out of any of the group discussions.

If you want to stop taking part in the activity completely, that is also fine, you just need to let us know.

<u>Who You'll Work With:</u>

You will be working with a group of 10-15 people, this will include:

- The Kailo team
- Young people aged [11-25] from [place]
- Adults from youth charities, community organizations, schools, mental health services, local government, or parents/carers

<u>Time Commitment:</u>

- Group sessions, once every 2 to 3 weeks, for 6 months (possibly longer)
- Each session lasts 2 to 3 hours, likely on weekday evenings
- Optional additional tasks between sessions (voluntary)

What info will we collect about you?

- Your name, email, address, phone number, date of birth, dietary requirements to help us organise the sessions and also to pay you your voucher afterwards.
- We may also ask questions such as your gender, race, where you live, neurodiversity, sexuality. You don't have to answer any of these questions if you chose not to, it won't affect your participation in the activity. It just helps us to know we are working with people who have a diverse range of experiences.
- Notes from the [workshop/conversation] (we'll keep them anonymous)
- We might record sessions and take photos to remember what was said (with your permission- if you don't want us to, that's fine, and if we want to use the photos or videos we will always check with you first)
- We combine everyone's ideas into a "big picture" so your particular comments/contributions will be kept anonymous. This combined information might be shared others and might include quotes you have said (but they will always be kept anonymous).

Your details will be stored securely, and we will only use them for this purpose, and everything will be deleted within [5 years of Kailo finishing]. Where possible, we will always try and share the information we collect back with you, so you can check that it is accurate. We'll only share your personal details with other people if what you say makes us worried about yours, or anyone else's safety.

If you want us to delete any data or things you have shared we will do our best to do so (as long as we've not already made it anonymous). We may be unable to delete group workshop data (if it contains other people's data too), but we can delete any other information we have about you such as your name and contact details.

What will you get for taking part?

Payment

As a valued member of the Kailo team, you will receive payment in the form of [voucher/BACS] at the rate of [£p/h] as well as all reasonable expenses, including travel. Food will also be provided in the sessions.

Skills Development

- Learn and develop skills in **social research** and **design** and apply them in practice
- Group work and facilitation skills
- Increase your contacts and networks with youth and community organisations in [Site name]
- Be part of a process from an early design phase through to implementation and delivery in the community.
- Gain valuable work experience, that demonstrates key transferable skills to other settings (e.g. teamwork, communication, problem solving, critical thinking).

What support will you get?

- Regular check-ins with Kailo leads [insert names]
- Sessions tailored to group and individual needs
- Group agreements to create a safe and supportive environment
- No pressure to contribute—participate only when comfortable
- Personalised support to help you develop and grow
- Sensitivity to mental health topics, with opportunities to talk to the Kailo team before the sessions begin to share any concerns or triggers for better support within the sessions.

Further Information

If you are interested in taking part and would like to find out more about how to get involved, please get in touch with the Project Lead [name] at [email].

To make a complaint, or raise any concerns, compliments and other feedback related to this project, contact [name] at [email].

You can find out more about our privacy and data sharing policy on our website at: [link to privacy notice]

Thanks for reading—hope to see you soon! 🎉
