# Supplementary material for: An adaptive, youth-centred co-design methodology: place-based co-design centring youth and community participation
Source: Res Involv Engagem. 2026 Jan 24;12:33. doi: 10.1186/s40900-025-00833-w (PMC12994241; doi:10.1186/s40900-025-00833-w)
Supplement: Supplementary file 4 — Supplementary Material 4 [file 40900_2025_833_MOESM4_ESM.docx]

# **Co-design Group: Information Sheet for Young People**

**What is Kailo?**

Kailo is a new research and design project working with young people and communities to learn about the local issues that impact on young people’s mental health and wellbeing and work together to improve them.

Over the past 8-10 months we have been speaking to lots of young people and adults in Newham/North Devon to understand what they think impacts young people’s mental health and wellbeing the most, and what needs to change or improve.

The key areas that were identified and prioritised in North Devon as key questions or challenges that need to be addressed were:

1. How might communities be places where young people can feel accepted, supported and belong?
2. How might we increase mental health awareness, literacy and strategies for young people, families and other key people in YP's lives, so they can build stronger, more supportive relationships?
3. How might we inspire, support and connect young people to a diverse range of opportunities, jobs and careers?

**What is Co-design?**

Co-design is at the heart of Kailo and is the main way that we are working with local communities to bring about positive change for young people’s mental health and wellbeing.

One way to describe co-design is as “an approach to **designing with, not for, people**. While co-design is helpful in many areas, it typically **works best where people with lived experience, communities and professionals work together** to **improve something** that **they all care about**. Overall, the primary role of co-design is elevating the voices and contributions of people with lived experience.” Kelly-Ann McKercher | Beyond Sticky Notes ​

As part of Kailo, we are bringing together groups of people with different experiences to work together to design new ways to improve young people’s mental health and wellbeing by working on the three prioritised areas outlined above.

Everyone working on the project will have a common interest or passion to improve young people’s mental health and wellbeing in North Devon, but everyone will bring different experiences, perspectives and ideas that we will bring together, combine and develop to create meaningful, sustainable change for young people.

**About you**

To work with us as part of the Kailo co-design group, you need to be:

- Be between the ages of 12-25
- Be living in North Devon or Torridge
- Be passionate about improving mental health and wellbeing for young people
- Have experience of being impacted by one of the key areas that are identified below
  - Identity and belonging in the community
  - Accessing more diverse job and career opportunities
  - Increasing awareness of mental health and wellbeing in the wider community

We are particularly keen to hear from underrepresented groups of young people, for example if you:

- Live in a remote, and rurally isolated area
- Live in a neighbourhood with a low rate of progression to higher education or a high level of socio-economic deprivation
- Are not in employment education or training
- Identify as part of a minority group (e.g. LGBTQIA+, neurodivergent, race or ethnicity)
- Have cared experienced and/or are currently in care.
- Are from traditionally working-class backgrounds and communities

**What am I signing up to?**

*Key Activities*

- Attending group sessions with the co-design team to work with Kailo to explore and understand more about the Opportunity Areas, drawing on your own experiences (where appropriate, and where you feel comfortable to do so)
- Problem solve and generate ideas with the co-design team to come up with solutions to the challenges identified under the Opportunity Areas
- Support with raising awareness and integrating the work into the wider community.
- Possible individual or smaller group tasks to complete between sessions (based on your availability and willingness. This is not mandatory)

*Who will I be working with?*

You will be working with a group of 10-15 people. This group will consist of:

- The North Devon Kailo team
- Young people between the ages of 12-25 who are interested in improving mental health and wellbeing across North Devon and Torridge.
- Adults who work in North Devon. They might be working in youth charities, community organisations, mental health and wellbeing services, teachers, local government or they could be parents/carers or other adults interested in improving young people’s mental health.

*Time Commitment*

We are asking people to commit to attending group sessions every 2 to 3 weeks for 6 months. Each session will last between 2 to 3 hours.

The day and time of the sessions will be based on the majority of the group’s availability. *The sessions will most likely take place on a weekday evening to enable young people who are in education, employment, or training to participate, but this is subject to change depending on who is in the small circle.*

If you have time, and want to do more, there is a possibility of additional individual or smaller group tasks to complete between sessions. (This is not mandatory! If you didn’t want to do this, it wouldn’t affect your participation in the co-design group)

**What will I get out of it?**

*Payment*

Payment will be given in the form of a Love2Shop voucher £15p/h

All reasonable expenses will be covered in addition to the voucher payment, including travel. We will also provide food for you during the in-person sessions.

*Skills development Opportunities*

- Learn and develop skills in **social research** and **design** and apply them in practice
- Group work and facilitation skills
- Increase your contacts and networks with youth and community organisations in North Devon
- Be part of a process from an early design phase through to implementation and delivery in the community.
- Gain valuable work experience, that demonstrates key transferable skills to other settings (e.g. teamwork, communication, problem solving, critical thinking)

**What support will I get?**

Our main priority is supporting anyone who wants to be involved in Kailo, feel comfortable, supported and confident to be involved.

This will involve (but is not limited to):

- Regular check-ins with the Kailo lead in North Devon/Newham throughout the whole process.
- Adapting our sessions to suit your needs, and other needs in the group
- Working with the group at the very start of the process to develop group agreements regarding how we work together, so everyone feels safe and confident to contribute.
- Absolutely no expectation that you have to contribute or input into every discussion or exercise. We will only ask you to contribute when you feel confident and happy to do so. If you decide at any point that you don't want to be part of the project anymore, that is totally fine.
- We will make sure we have a good idea of what you, as an individual, want to get out of the sessions so that we can support you to really develop and excel in these areas.
- Kailo is focused on improving mental health and wellbeing. We know this isn't always the easiest thing to talk about. Before we start the work, we will get to know each other, and ask you to think about if there is anything that you might find difficult to discuss in the group, or that you might find triggering and you can let us know how we can best support you if you need us to.

**What information will you collect about me?**

We’ll collect basic contact details (name/email/phone number/age) to help us organise the conversation/workshop/activity, invite you and pay your voucher afterwards.

We may also ask some other questions about you so that we can make sure we are working with people who have a diverse range of experiences. This would include: Gender, Race, questions about where you live, neurodiversity, sexuality. You don’t have to answer any of these questions if you chose not to, it won’t affect your participation in the activity.

We will keep these details while the project is still running in Newham/North Devon. We’ll delete them 3 months after the project has finished.

Your details will be stored securely and we will only use them for this purpose or if you have agreed that we can pass on your details to the evaluation team.

If you want us to delete them at any time just email and let us know

**What will you do with the info I share in the conversation/workshop/activity?**

- We will take notes about what you tell us but will keep it anonymous so that no one will know it was you that said it - we may give you a fake name in the notes!
- We may also video or voice record the session, so that we can remember what happened, but we won’t share these with anyone outside the Kailo project team. We’ll check with you before we do this.
- We will combine the information you share with other people’s feedback to help us understand what needs to change, and how we might do that.
- We might share this combined information in reports, infographics, on our website and in presentations. These might include specific quotes that people have said, but they will always be anonymous.
- We will always try to share the information we collected back with you, so that you can give us feedback about if it is accurate.
- The only time we will share what you tell us with other people is if we are worried for yours or someone’s else's safety because of it.
- We will keep all notes and recordings in a secure folder, only accessible by the project team.

For more information please see our privacy statement:

<https://kailo.community/privacy-policy-2/>

**How can I find out more and sign up?**

If you are interested in getting involved, that’s great!!

If you have any questions or want to have a chat to find out a bit more, then please feel free to text/call/WhatsApp/email ……….

If you are keen to get involved, then we need you to answer a couple of questions for us. The spaces are limited in the co-design group, so if there are lots of people who want to join we might have to make a decision about who joins the co-design group.

1. What is your name and how old are you?
2. Why do you want to get involved with Kailo? What strengths do you think you would bring to the project?
3. Out of the three opportunity areas, which one are you drawn to the most? Why?

You can answer these questions in any way you like (video, email, WhatsApp message, text, voice note). Send either to PHONE NUMBER or email them to ……

Once you have sent us the answers to these questions, we will arrange a time to have a call with you, to go over the requirements and expectations of the role, give you the opportunity to ask any questions, and so we can get to know each other a bit better, including what your availability to join the group is.

**We can’t wait to hear from you!**

For more information about the project, please see our website:

<https://kailo.community/about/>
