# Supplementary material for: An adaptive, youth-centred co-design methodology: place-based co-design centring youth and community participation
Source: Res Involv Engagem. 2026 Jan 24;12:33. doi: 10.1186/s40900-025-00833-w (PMC12994241; doi:10.1186/s40900-025-00833-w)
Supplement: Supplementary file 5 — Supplementary Material 5 [file 40900_2025_833_MOESM5_ESM.docx]

Supplementary Material 5: Types of organisations represented by participating Big Circle members

| **Types of organisations represented by participating Big Circle members** | **% Across the two sites** |
| --- | --- |
| Health (includes primary care, mental health, specialist services, both charities and NHS services) | 12 |
| Delivery organisations working directly with CYP and families (including youth work organisations, family support charities, organisations supporting SEN and neurodiverse CYP, and organisations supporting CYP's mental health needs) | 30 |
| Community organisations (includes organisations working within sectors such as local heritage, environment, and community networks) | 7 |
| Funders and commissioners (including clinical commissioning groups of local health services) | 2 |
| Voluntary services (organisations supporting and providing advice to charities to represent, support and champion community action within their local area) | 7 |
| Local businesses (including cafes, activity centres, music and theatre companies) | 3 |
| Research organisations (universities, independent research organisations) | 3 |
| Education (primary, secondary, further and higher education providers) | 16 |
| Local government and council members (public health, community engagement, economic development) | 16 |
| Statutory services | 3 |
