# Supplementary material for: An adaptive, youth-centred co-design methodology: place-based co-design centring youth and community participation
Source: Res Involv Engagem. 2026 Jan 24;12:33. doi: 10.1186/s40900-025-00833-w (PMC12994241; doi:10.1186/s40900-025-00833-w)
Supplement: Supplementary file 6 — Supplementary Material 6 [file 40900_2025_833_MOESM6_ESM.docx]

| **Timeline of Small Circle Sessions** | | |
| --- | --- | --- |
|  | **Details on focus and methods** | **Proposed outputs/outcome** |
| Small Circle recruitment with the support of KCP | Young people were recruited into the Small Circles via opportunity/convenience sampling [1] and existing connections with Young People from community partners (word of mouth).  Recruiting participants for Small Circle sessions involved conducting initial conversations with young people and community members. The conversations covered a series of important questions aimed at achieving the following objectives:   - Assessing whether the young person was interested in participating in the Small Circle sessions. - Ensuring that the young person understood what Small Circle sessions entailed, how they could benefit from them, and what would be required of them. - Supporting the design of the Small Circle sessions by assessing the needs of young people, identifying who needed to be present in the room for them to feel comfortable, and determining the type of facilities and materials that would be required. | - Young people recruited into the Small Circles - Signed consent forms and necessary personal information collected - Making changes to the planning of future sessions to ensure young people’s needs are being met (i.e. making reasonable adjustments) |
| Session 1 | The session aimed to bring the recruited groups of participants together to explore team building, group dynamics and creating a safe space. This was achieved through delivering an:   - Introduction to Kailo and the project aims - Introduction to the purpose of the Small Circle group and how it aligns with Kailo’s project aims - Discussion on the groups expectations for being part of the small circle, and the ways that they will work together to ensure everyone feels safe and confident in the space i.e. development of a group agreement [2]. - Introduction to OAs and their purpose within Kailo | - Participants leave the group with a shared understanding of the group’s purpose and aims within Kailo - Group Agreements developed based off the conversations with the participants as what is needed within the group for the space to be safe.   **Example group agreements from Northern Devon Small Circles**:  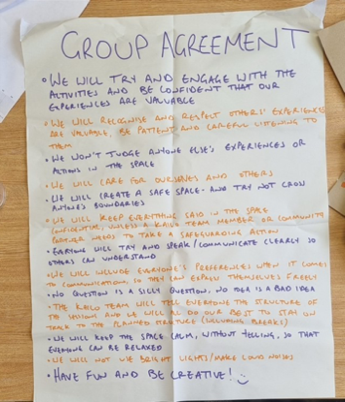  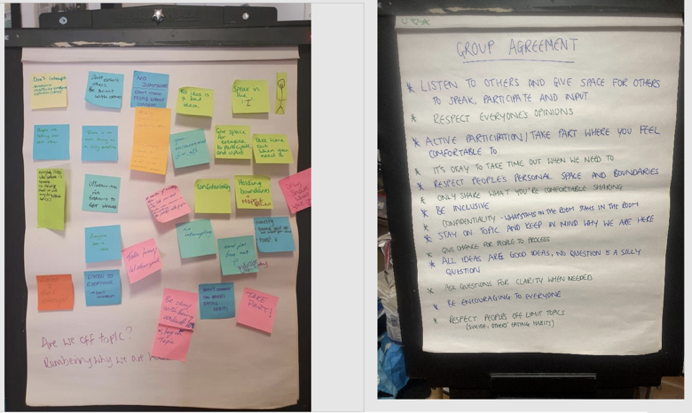  **Example group ‘Rules of Engagement’ from Newham Small Circles:**  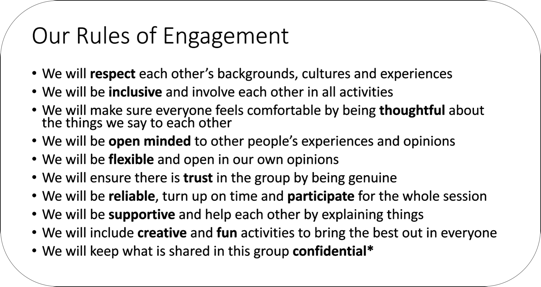   - An understanding of what an OA is and the process that was conducted to surface and prioritise them during Early Discovery   **Example: Summary of OA discussion in a Newham Small Circle**  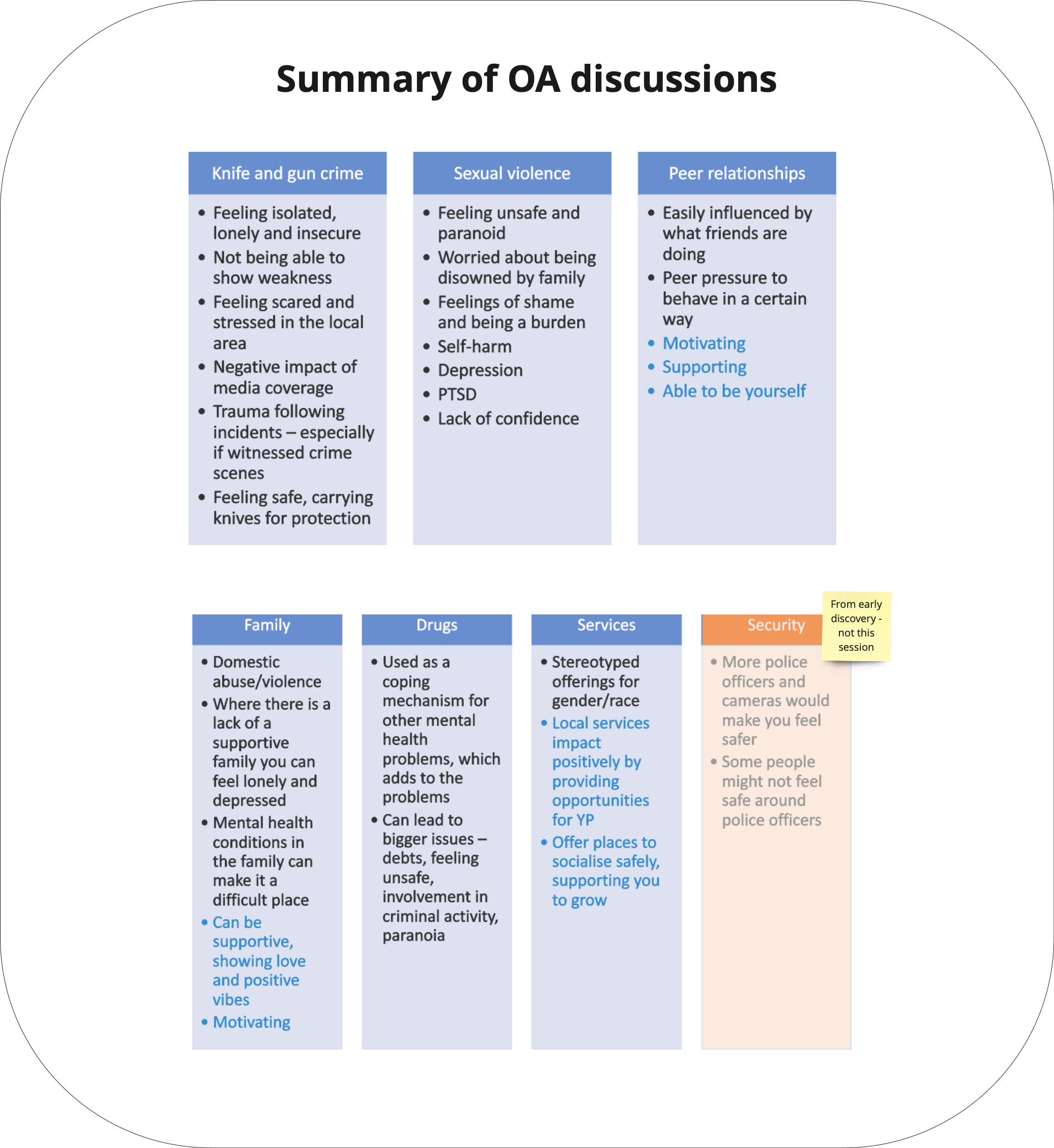 |
|  | **Example: Creative/artistic methods used (such as colleagues, picture making)** to allow the young people to express themselves and define themselves to the rest of the group (Northern Devon) | - A visual representation of the young people within the Small Circles through collages   **Example: Collages created by young people within the Northern Devon Small Circles**  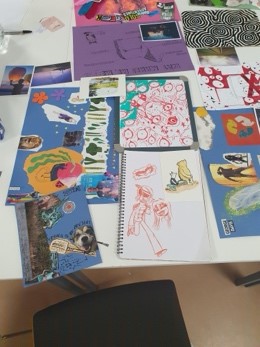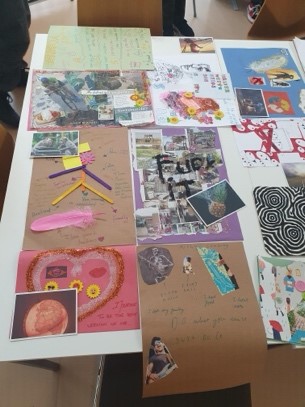 |
| Session 2 | The session aimed to build on the basic awareness of OAs developed through session 1. This was achieved by introducing the participants to the specific OAs that were surfaced and prioritised during the Early Discovery phase. It also started the preparation for the future Group Model Building (GMB) sessions, which included conducting a ‘Hopes and Fears’ exercise [3], as a means of developing group cohesion and surfacing any potential risks for future codesign stages.  **Image of template used during the session to explore awareness of OAs:**  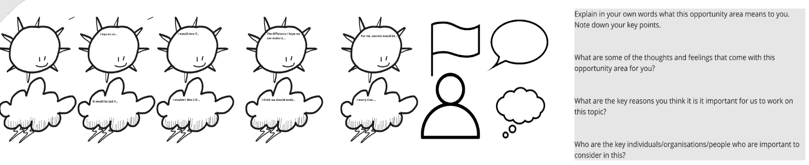  The Kailo team and community researchers summarised these outputs through clustering and thematic analysis. | - Shared understanding by participants of the OAs prioritised in the Early Discovery   **Example: Insights gained from the young people in Northern Devon Small Circles which helped to build their collective understanding of the OAs:**  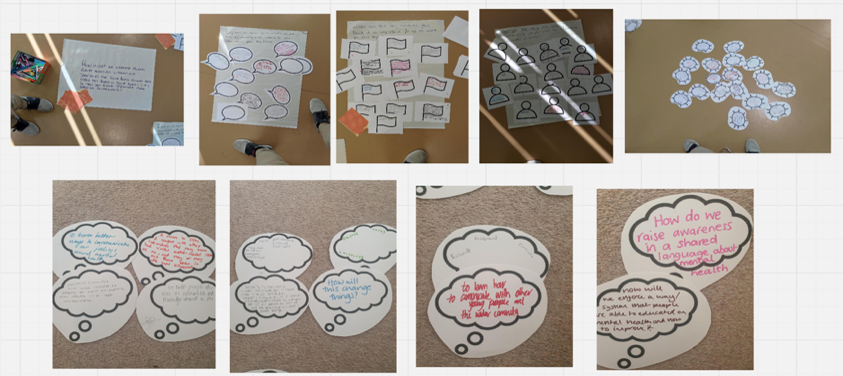  **Example: Insights from Newham Small Circle based on group and personal reflections on the OA**:  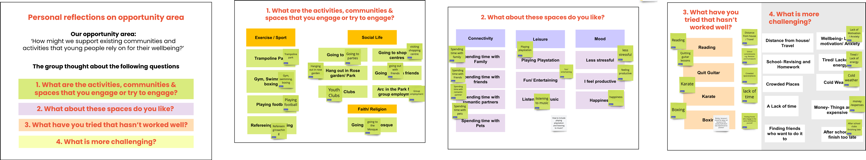  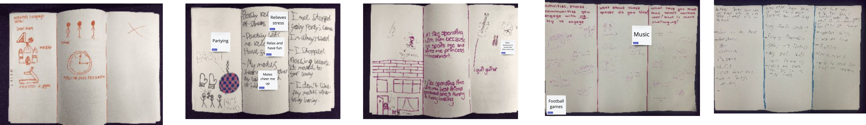   - List of ‘Hopes and Fears’   **Example: Newham Small Circle ‘Hopes and Fears’ output:**  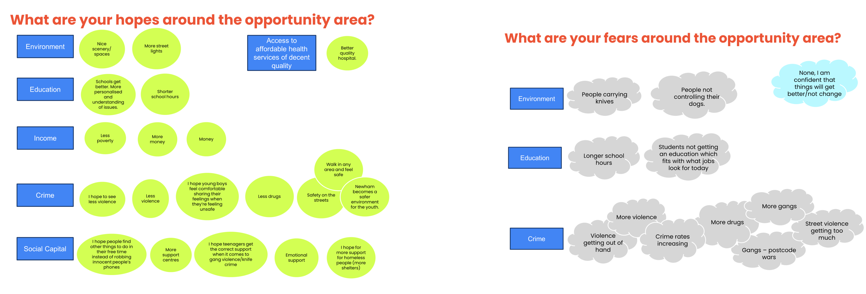  **Example: Image of the clustering and thematic analysis done by the community researchers and Kailo team (Northern Devon):**  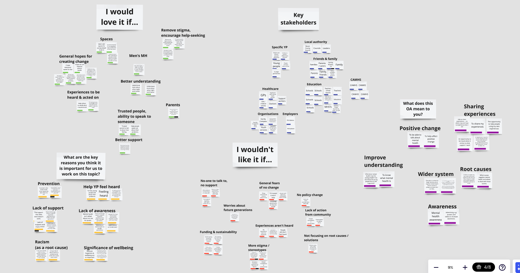 |
| Session 3 | **GMB Session I**  The session aimed to build on the introduction to GMB outlined in session 2. Activities were primarily focused on historical behaviour within the system, and included the development of:   - **Graphs over time [**4] to start framing the problems that exist regarding young people's mental health and wellbeing, and provide a starting point for discussions about the important variables to be considered - **Variable Elicitation** [3], including insights from [Evidence Briefings](https://brandplatform.annafreud.org/share/WwebLCcamsUFtWcW4WfE) [5] (in Newham) to explore which variables are impacting the identified problems. - **Connection Circles** [6] to explore the relationships between variables that are impacting the problem   Analysis of the variables surfaced through these activities was completed by the Kailo team and community researchers | - Development of Graphs over time - List of variables relevant to the Opportunity Area explored in the Small Circle - Use of potential variables to create Connection Circles   **Examples of graphs over time, connection circles and variables developed during the sessions within Northern Devon Small Circles**:  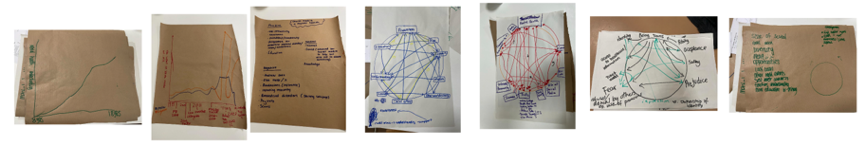  **Example: Group graph over time (Newham Small Circle):**  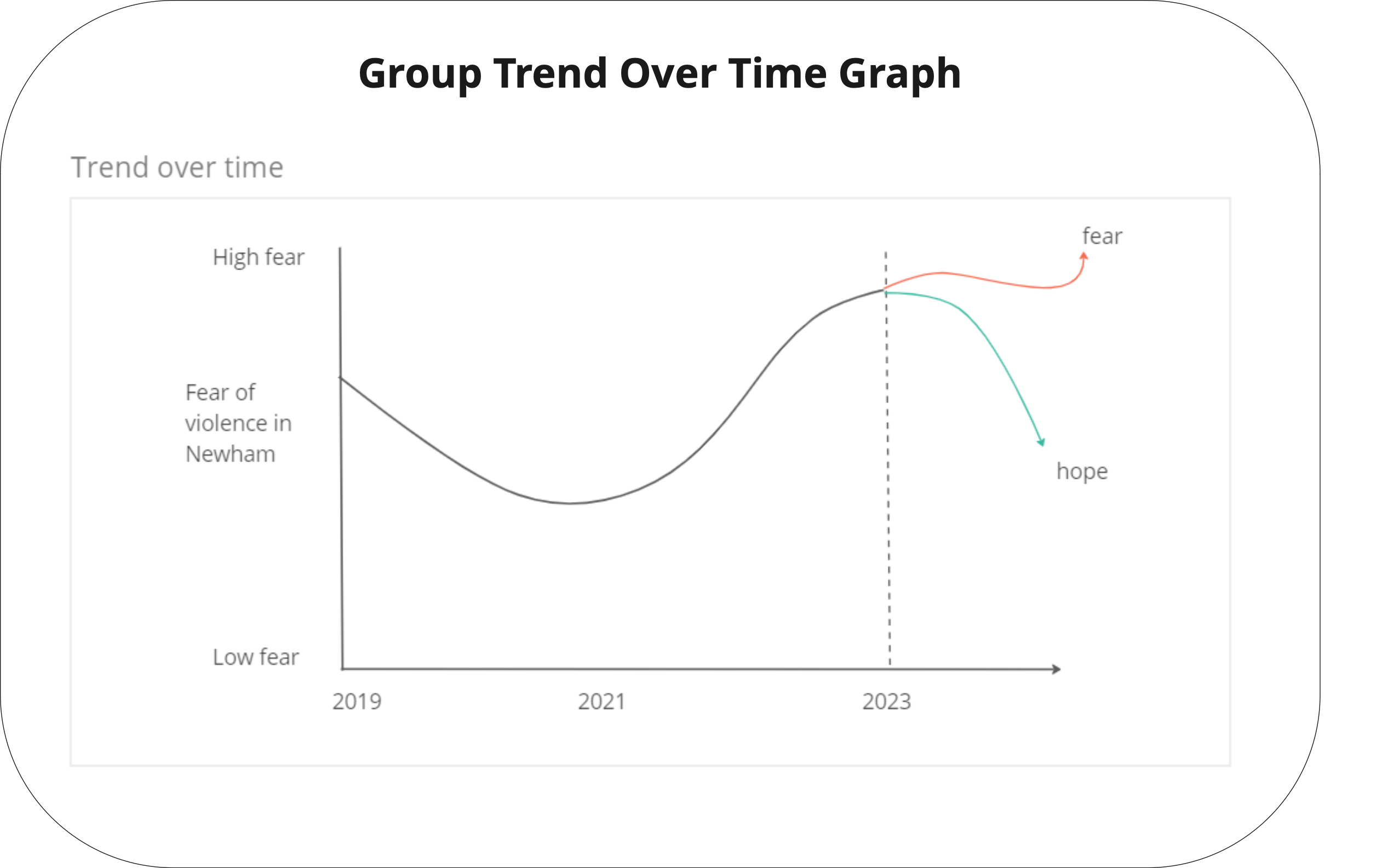  **Example: Variable elicitation activity (Newham)**  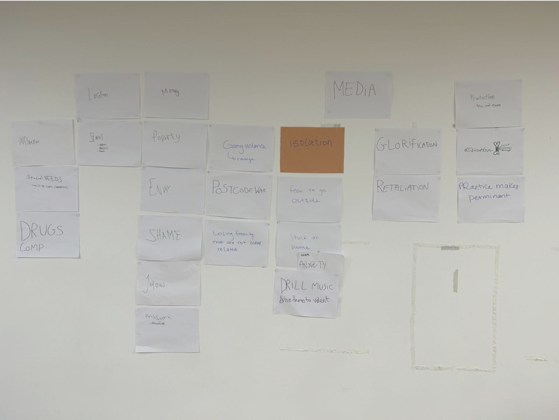  **Example: Image of variable analysis (Northern Devon):**  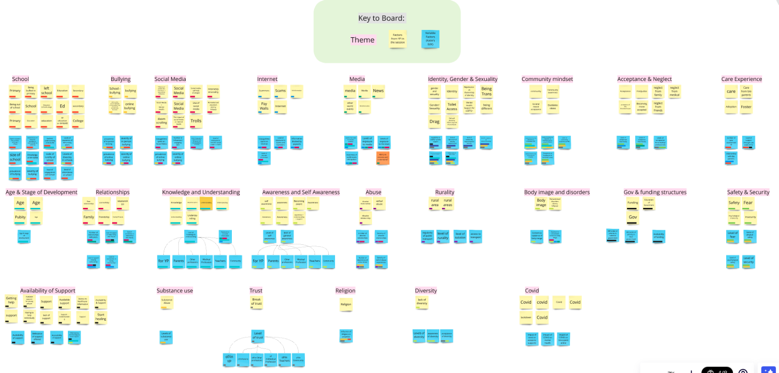  **Example:** **Image of variable analysis (Newham)**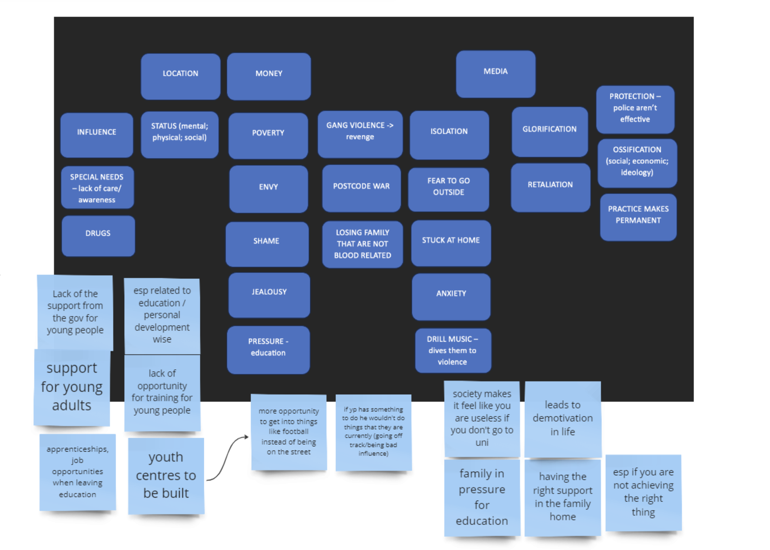 |
| Session 4 | **GMB session II**  The session aimed to build on the previous GMB session by further identifying and exploring the connections within the system. Activities were primarily focused on surfacing **Mental Models** to produce **Causal Loop Diagrams (CLDs)** [7] using the outputs of the previous session (Graphs over time and surfaced variables). This helped participants to further identify and explore the important variables and their relationships within the system  CLDs were presented to sessions with the Big Circle for feedback and refinement i.e. feedback input [8]. | - Refinement of list of relevant variables - Development of Causal Loop Diagrams [7],   **Examples of CLDs created during Northern Devon Small Circle sessions**:  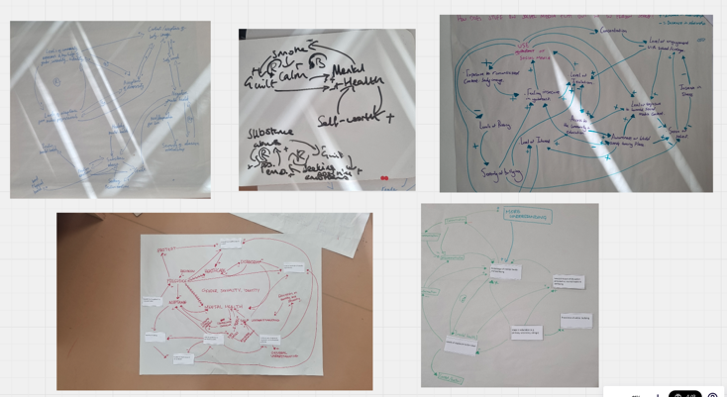  Examples of CLDs created during Newham Small Circle sessions:  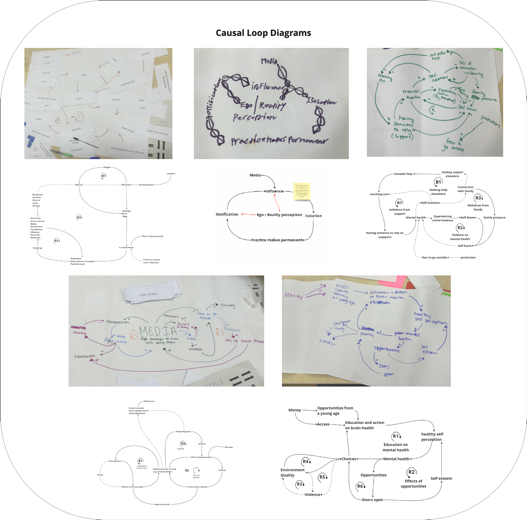  **Map 1: Informal community of support around mental health (Northern Devon)**  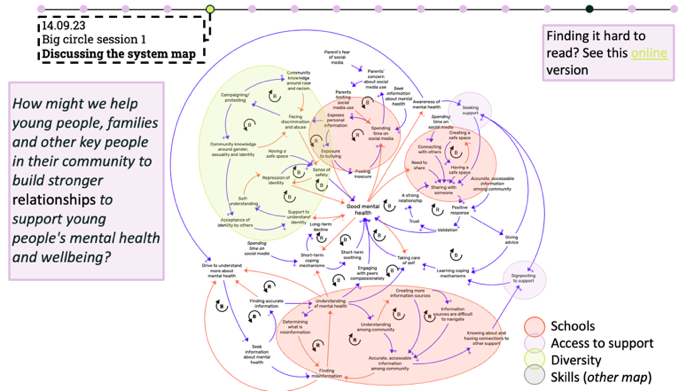  **Map 2: Diverse opportunities (Northern Devon)**  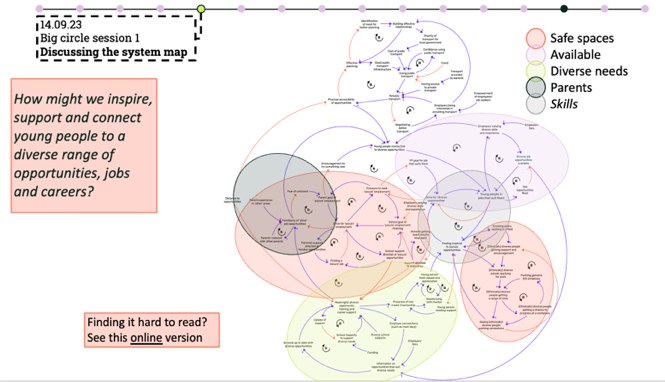  **Examples of CLDs created during Newham Small Circle sessions:**  **Map 3: Violence and Crime (Newham)**  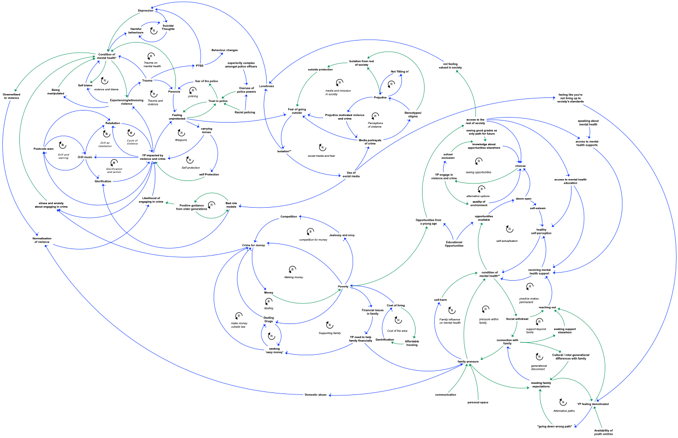 |
| Session 5 | **GMB session III**  This session was primarily focused on prioritising the themes and areas of focus for the next stage of the co-design process through considering places to intervene [9] and power dynamics (and what control/influence we have in the system). Using the **Causal Loop Diagrams** developed during GMB session II, and the information surfaced and explored through the creation of the **Graph over time** and **Connection Circles** activities in GMB session I, the Small Circle participants were asked to consider which variables and relationships should be prioritised when considering their OA. | - Prioritised the specific areas for the next stage of co-design in relation to their OA of focus - Shared understanding and consent around prioritised areas |
|  | **Example: ‘Ripple Effect’- areas to intervene (Newham)**  Identifying the promising areas for change through the questions below:  **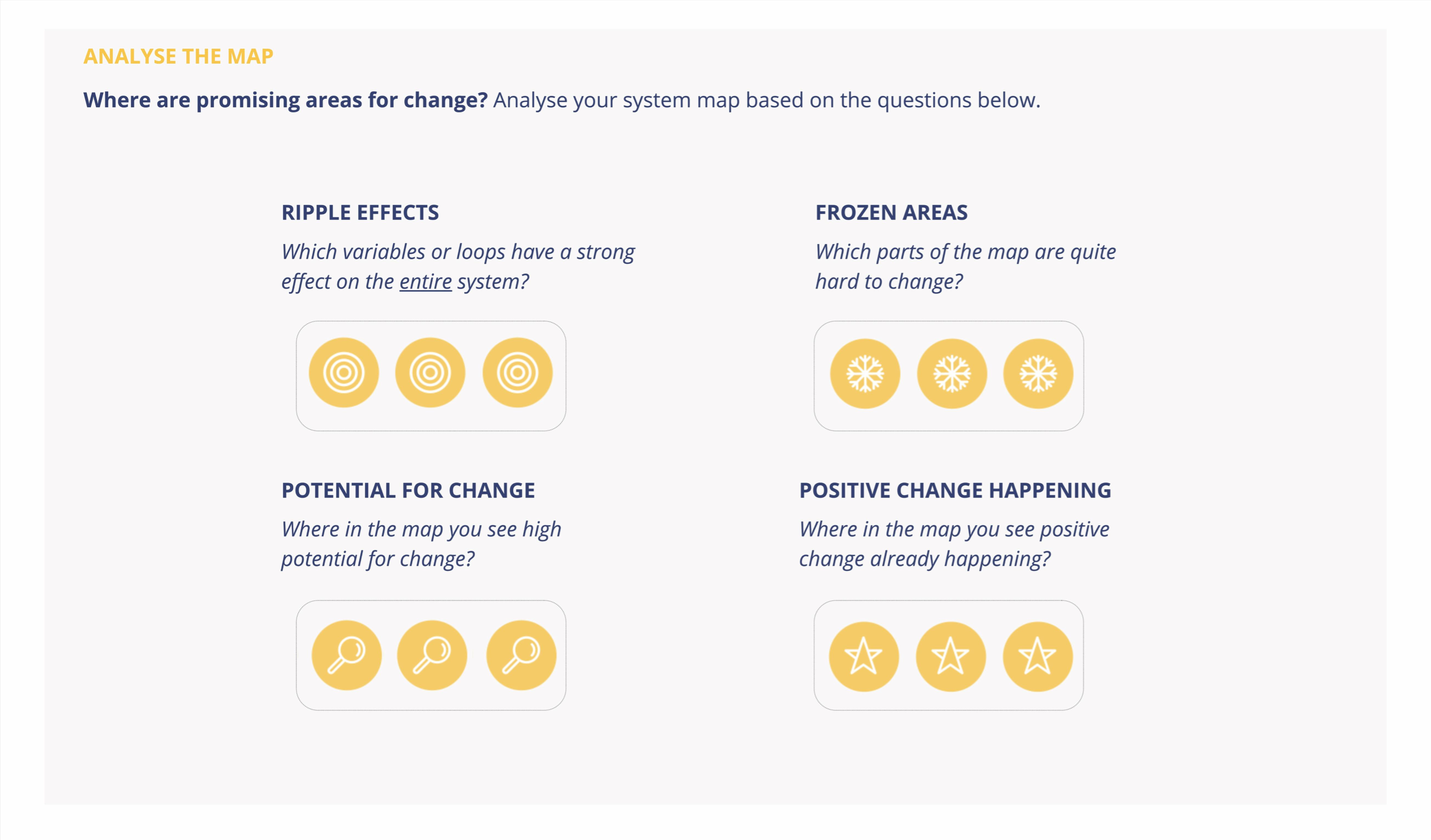** | 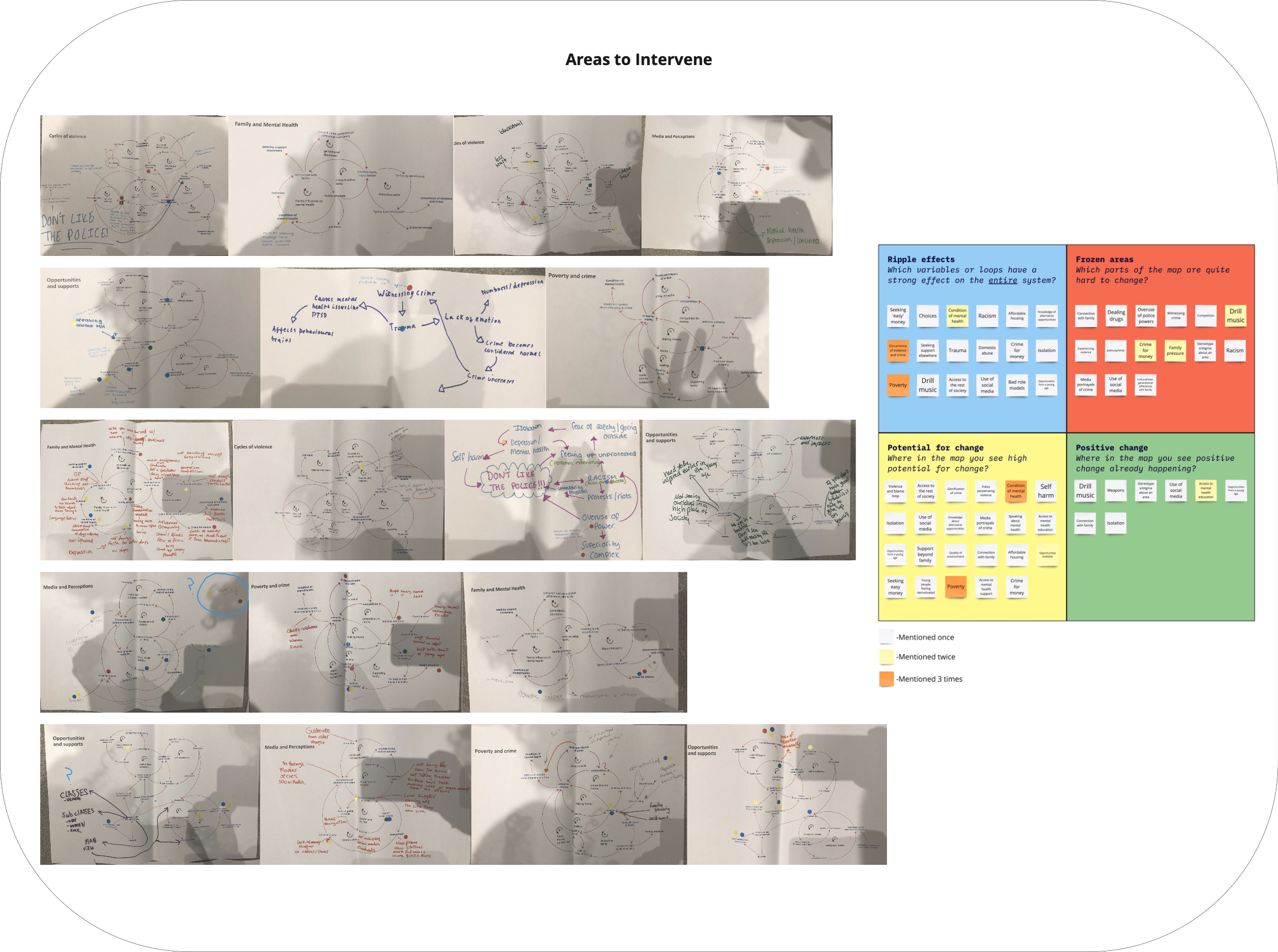 |
| Session 6 | **Pause and Reflection session**  This session’s purpose was to review the Small Circles process and learning up to this point.  Methods to facilitate this session included the creation of mood boards [10], and Avatars (whereby the Young People designed someone/thing that represented them, their thoughts, ideas, experiences etc.).  *Previous sessions had an identical protocol across the sites and Small Circle groups. However, from this point, we anticipated there would be adaptations based on the characteristics and requirements of each Small Circle. The aim was to reach the final session with a well-developed strategy to support young people's mental health, based on the OAs across each Small Circle, and therefore had to be reflective of the contextual factors of each Small Circle.* | - Visual representation of young people’s thoughts and feelings on the process to date   **Example from Northern Devon:**  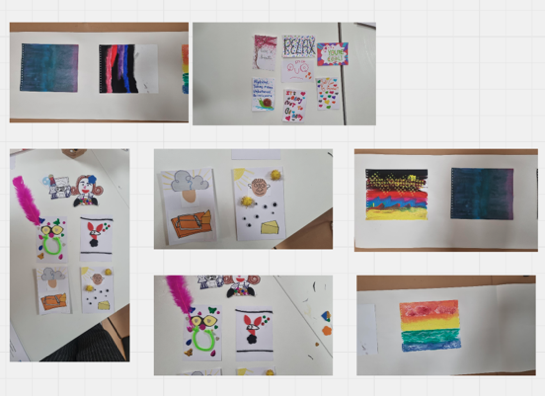   - Review of group agreement and adaptations made where needed - Refined outputs from previous sessions to inform prioritisation stage of the codesign process |
| **After this point, sessions were regularly adapted to fit the needs and pace of the different Small Circles. However, all Small Circles went through some version of the activities mentioned below in one or more sessions.** | | |
|  | **Prioritisation I** This activity's purpose was to use the **Causal Loop diagrams** created in the previous sessions to decide which loops should be prioritised and used to inform the next stage of co-design. This was achieved by using a prioritisation exercise, where Small Circle participants voted for the loops they felt should be taken forward (facilitated through Dot Voting/’Dotmocracy’ [11, 12] and group discussions). This exercise was conducted alongside the provision of insights from Evidence Briefings [13] in Northern Devon.  Once selected, a ‘Hopes’ exercise was used to explore potential solutions and strategies related to the prioritised loop. | - Prioritised loops to inform next stage of co-design   **Example: Image of the dot voting exercise to establish key areas of impact (Northern Devon):**  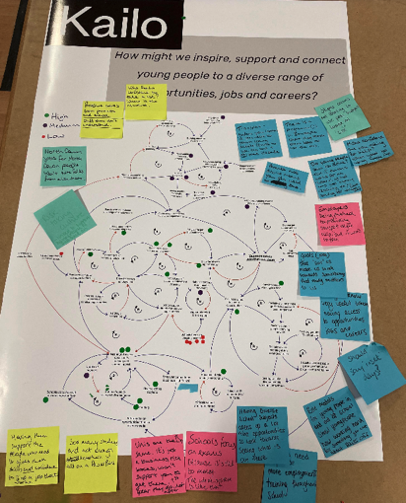  **Example: Image of the dot voting exercise to establish key areas of impact (Newham):**  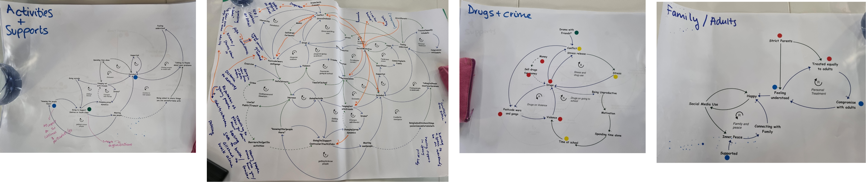  **Example: Prioritisation exercise used within Newham Small Circles**  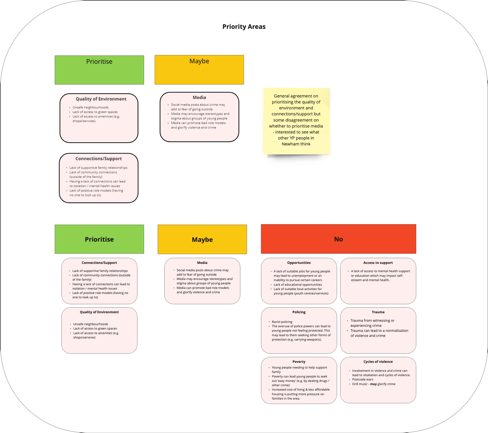 |
|  | **Prioritisation II**  Using the ‘Hopes’ exercise from the previous prioritisation session, the Small Circle participants were encouraged to think about the potential impacts is of the ‘Hopes’ they surfaced by using impact scales. These were used to prioritise the key design opportunity/impact areas through group consensus of what had the highest and lowest impact within the OA of focus.  **Scale used within Newham**:  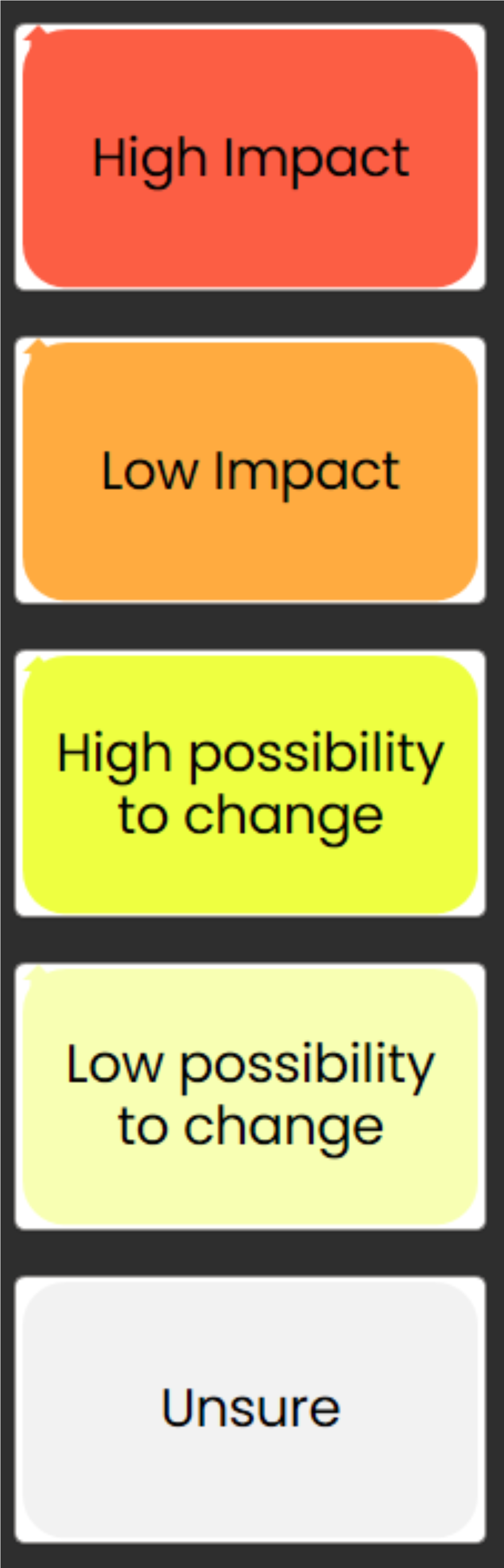 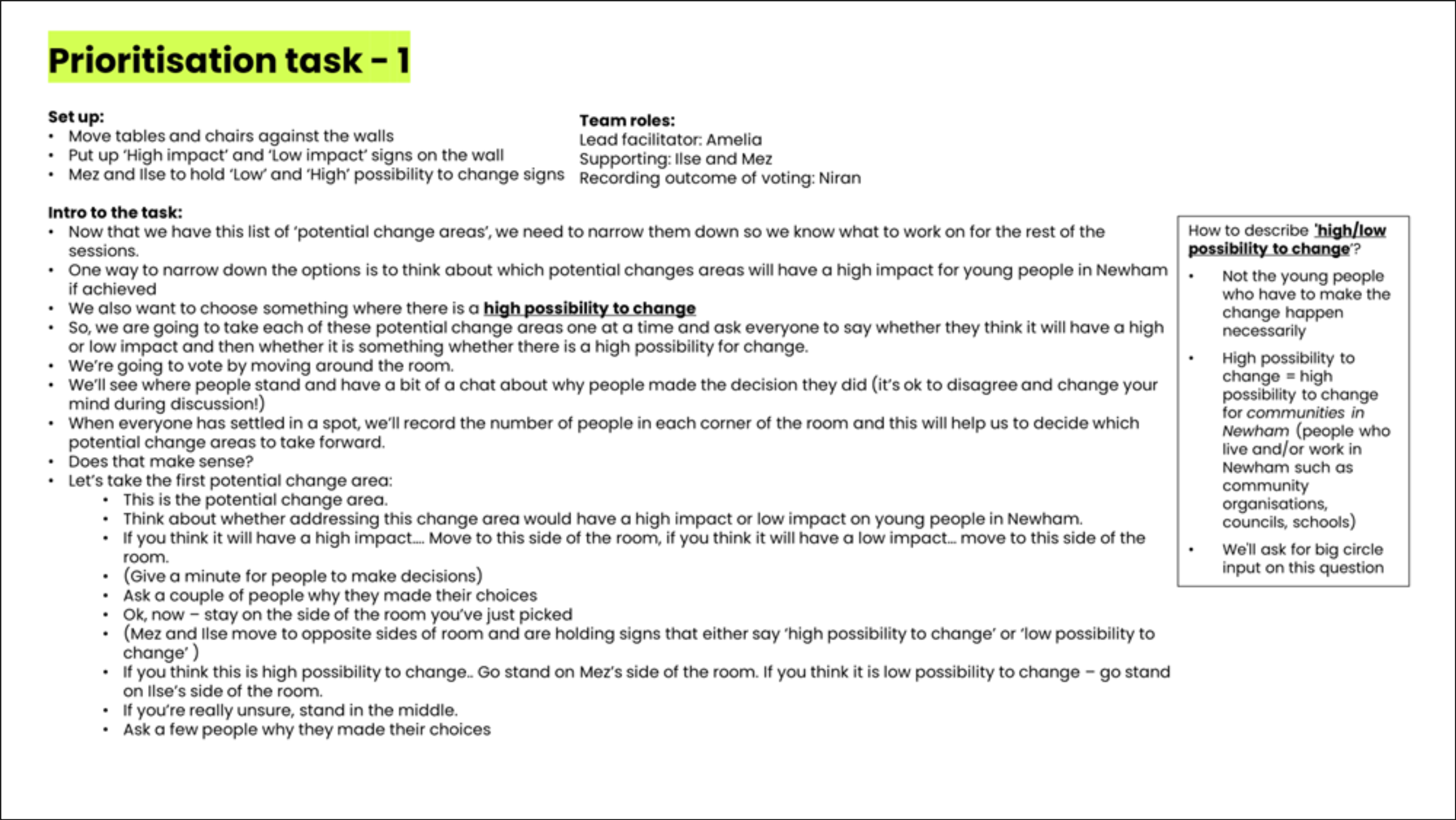  Through this exercise a list of the ‘Hopes’ (interventions, activities, solutions, changes etc) was developed; These are the prioritised design opportunities which will be further developed in future sessions  Using the ‘**How Might We’ [14] question tool**, the Kailo team reframed the challenges the Small Circle Participants had identified for young people’s mental health during GMB sessions into design opportunities. | - Impact scale   **Example of impact scale developed by Bideford Small Circle**:  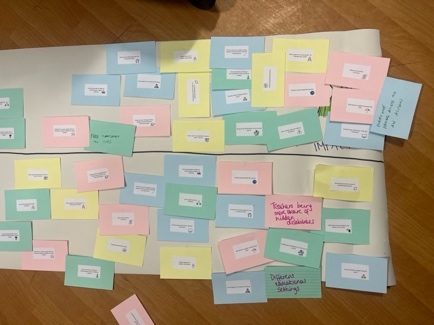  **Example if impact scale results from Newham Small Circle**:  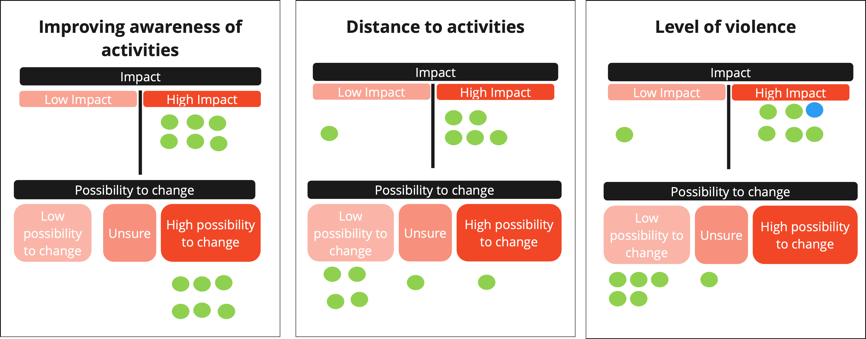   - List of ‘How Might We’ questions   **Example: Development of ‘How Might We’ questions (Bideford):**  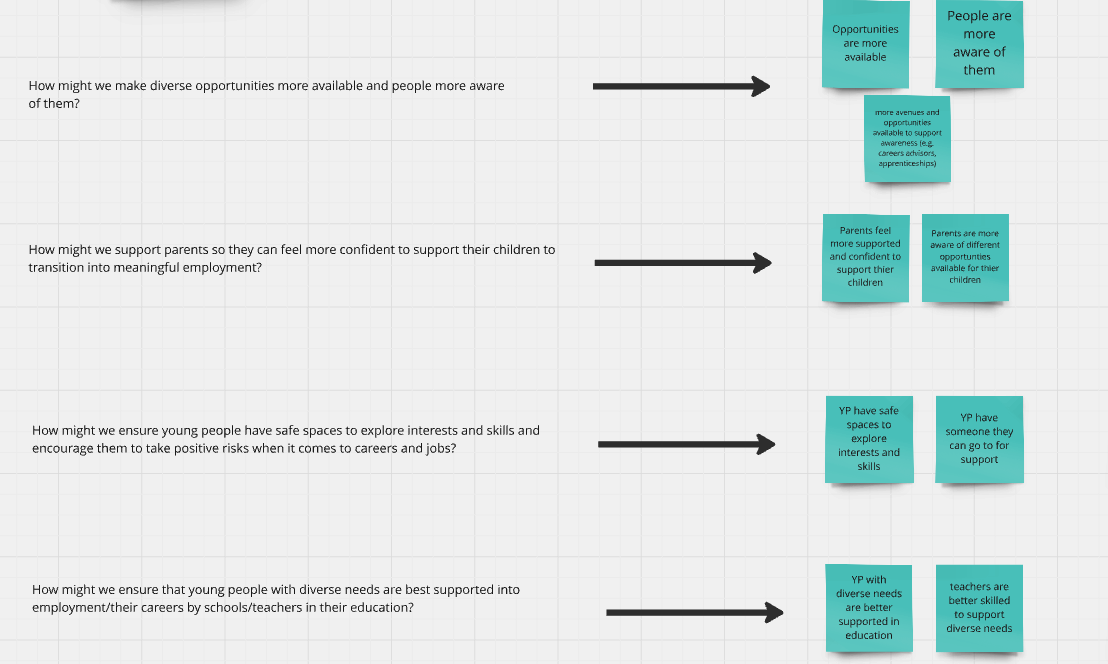 |
|  | **Stakeholder and actor mapping around prioritised hopes**  This session focused on identifying and mapping the key practitioners needed to be involved in future Small Circle sessions, in relation to the prioritised design opportunities surfaced in the previous session. By using **stakeholder mapping** to identify the most relevant practitioners to be involved in the sessions it was hoped that there would be greater community involvement and the prioritised design opportunities would benefit from their professional expertise and local knowledge of contextual factors [15] | - List of key practitioners to be invited to following Small Circle sessions   **Example: Stakeholder maps of key practitioners developed in Barnstaple and how this was incorporated into the system map:**  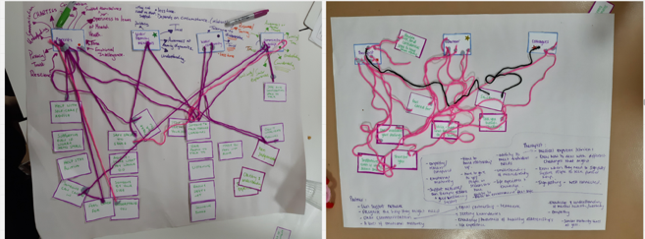  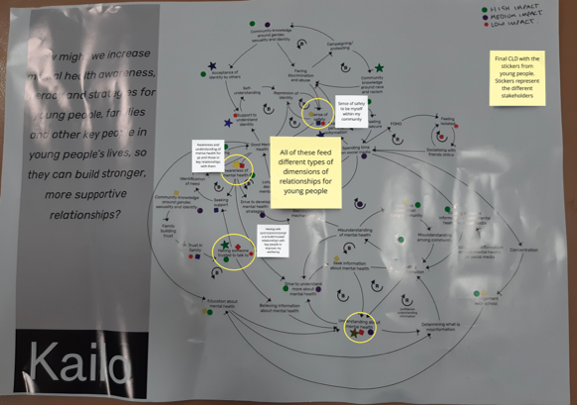 |
|  | **Future visioning around prioritised hopes** This activity focused on deciding what were the tangible options from the prioritised design opportunities developed in the prioritisation activities. This was informed by surveys and big circle sessions across both sites, as well as input from the KCP. Their input was taken back to the Small Circles to make the final decisions.  Through determining what was tangible and possible within the specific sites, this provided the vision for the future co-design opportunity which would guide the future Small Circle sessions. | - Vision for co-design opportunity |
|  | **Example: Taking opportunity areas to professionals outside of the Small Circle (Newham)**  In this activity members of the KST team in Newham engaged in conversations with professionals already working in the codesign areas developed by members of the Small Circle | - Feedback on the co-design areas developed by the Small Circle which was used to refine the vision for the co-design opportunity |
|  | **Example: Creating a vision of a ‘Perfect Newham’ (Newham)** | 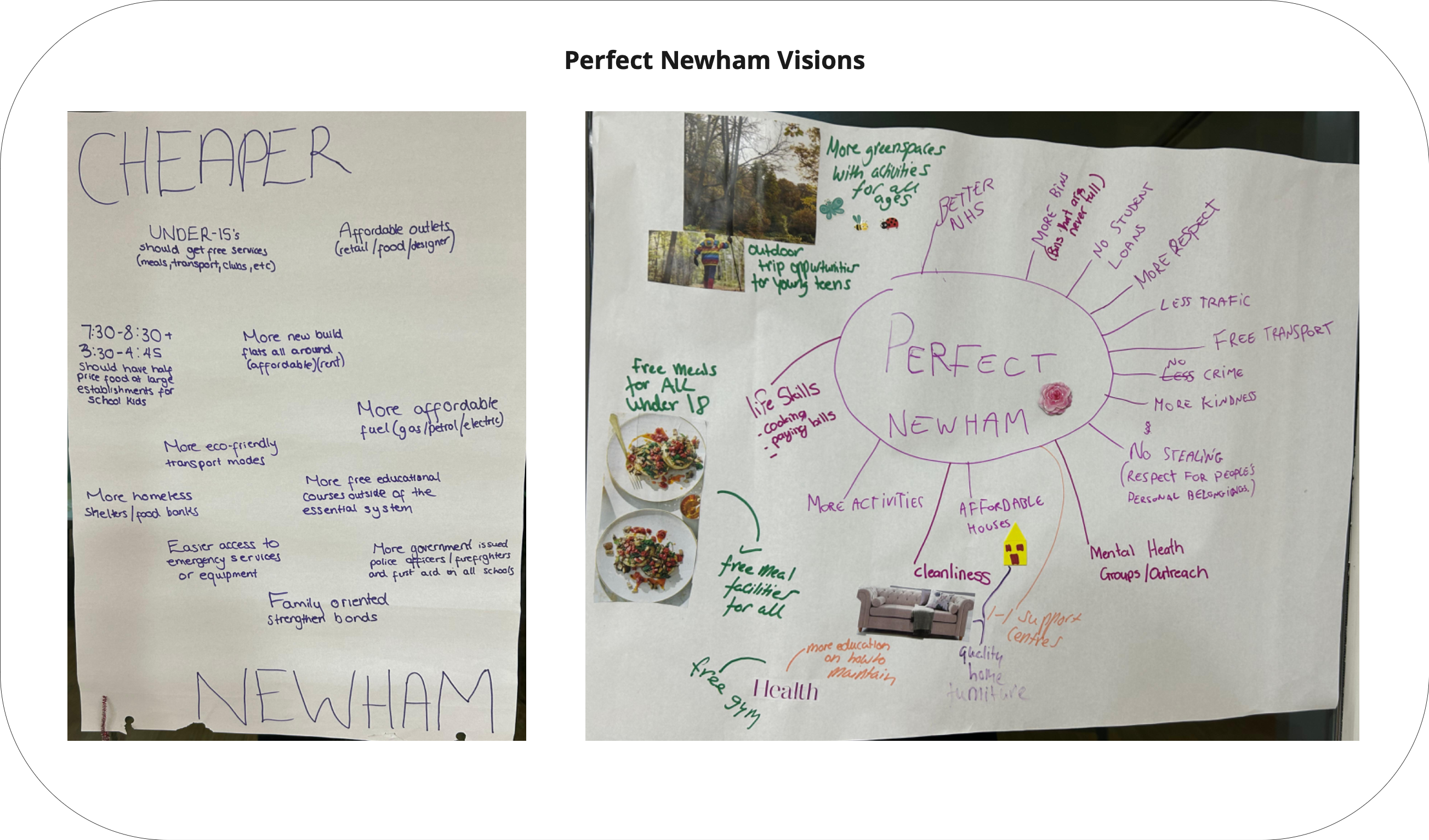 |
|  | **Example: Relationship building with professionals** **(actors that joined the** **Bideford Northern Devon Diverse Opportunities Small Circle**)  This session aimed to establish trusting relationships between Small Circle participants and actors that subsequently joined the Bideford Small Circle following the **stakeholder mapping** exercise in session 9. Activities in this session included building a shared vision for future co-design sessions, helping to create the foundations for a safe, collaborative and trusting space which are essential elements for enabling the co-design process [16]. | - Shared understanding and vision between young people and invited practitioners |
|  | **Ideation**  This session was focused on further developing the prioritised design opportunities surfaced in session 8. The Small Circle participants developed a long list of key elements required in the design through thinking about questions such as ‘What do we want the experience to look like?’. This long list will be used in the further development of the design opportunity in future sessions. | - Long list of key elements required in the design |
|  | **Example activity: Lotus flower ideation (Bideford and Barnstaple) [17]**  This framework was used to generate ideas, encourage creative thinking around a central theme and then breaking these down further into subthemes and elements.  This framework also provided a platform for collaborative idea generation; by using a World Café approach [18] in Barnstaple, Small Circle participants were able to build on the ideas of others | - List of different elements to consider that all relate to a central theme (**example from Bideford Small Circle**)   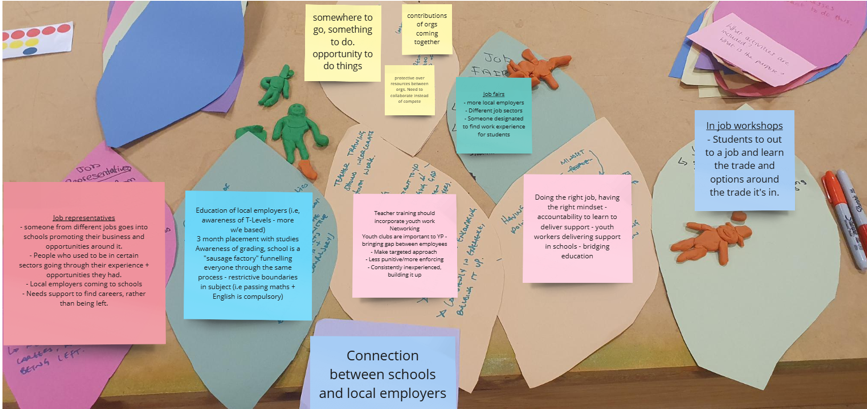 |
|  | **Example activity: Generating ideas of what could improve the two priority areas and shortlisting activity (Newham)**  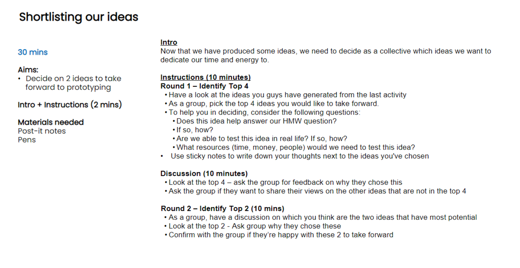  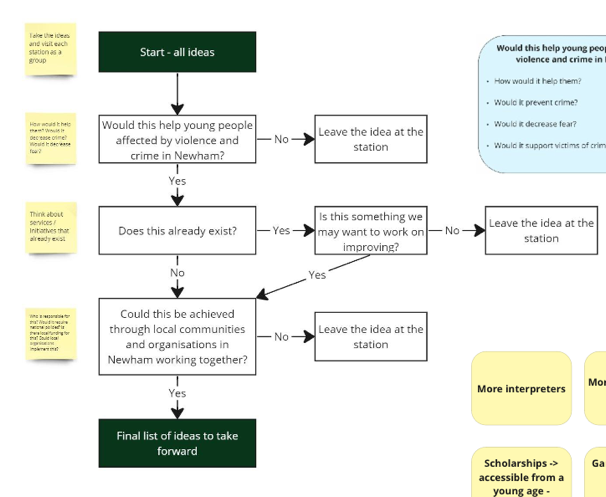 | - Ideas for improving the priority areas (**example from Newham Small Circle**)   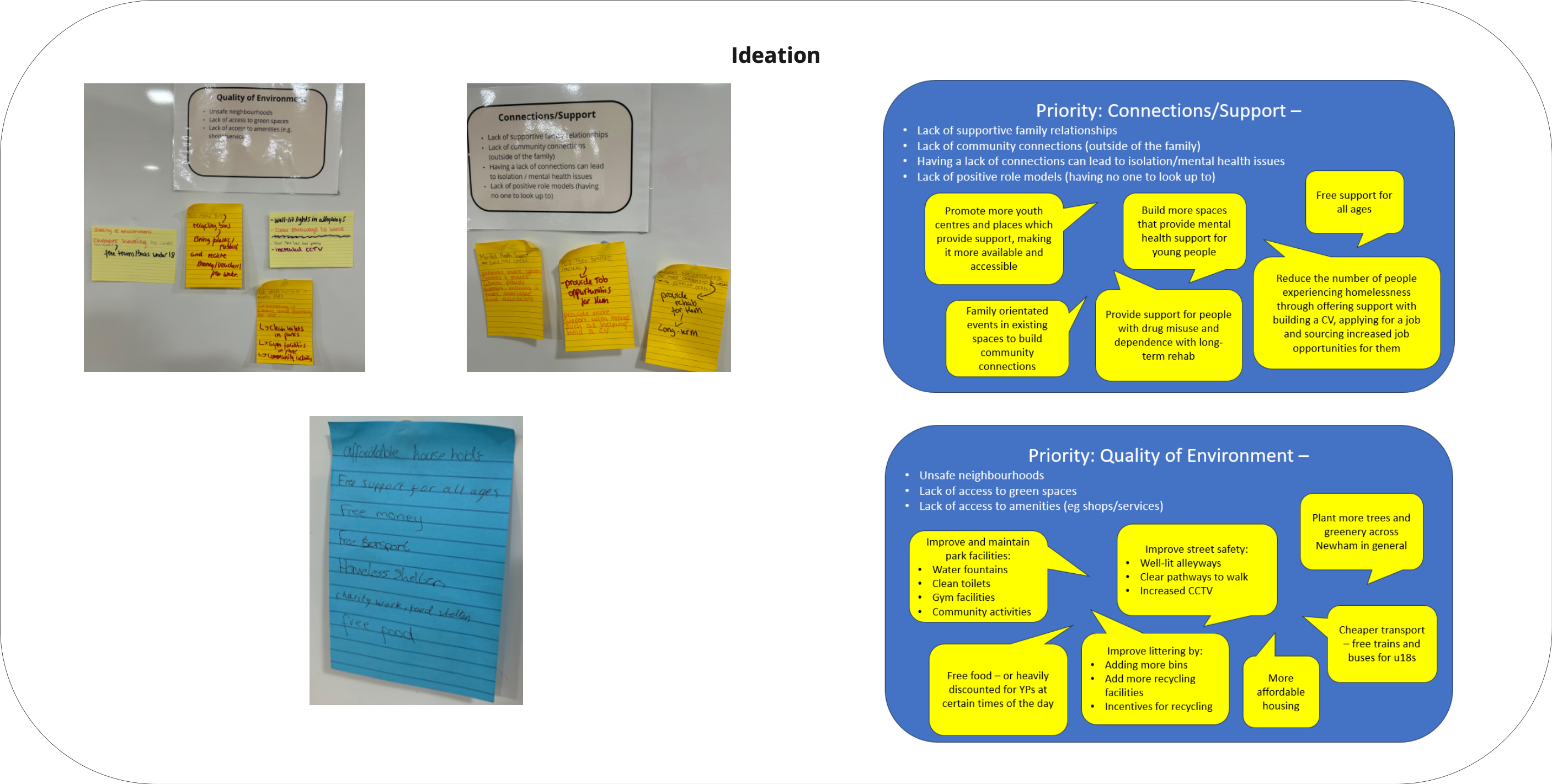   - Shortlisted ideas generated through the shortlisting activity **(Newham)**   **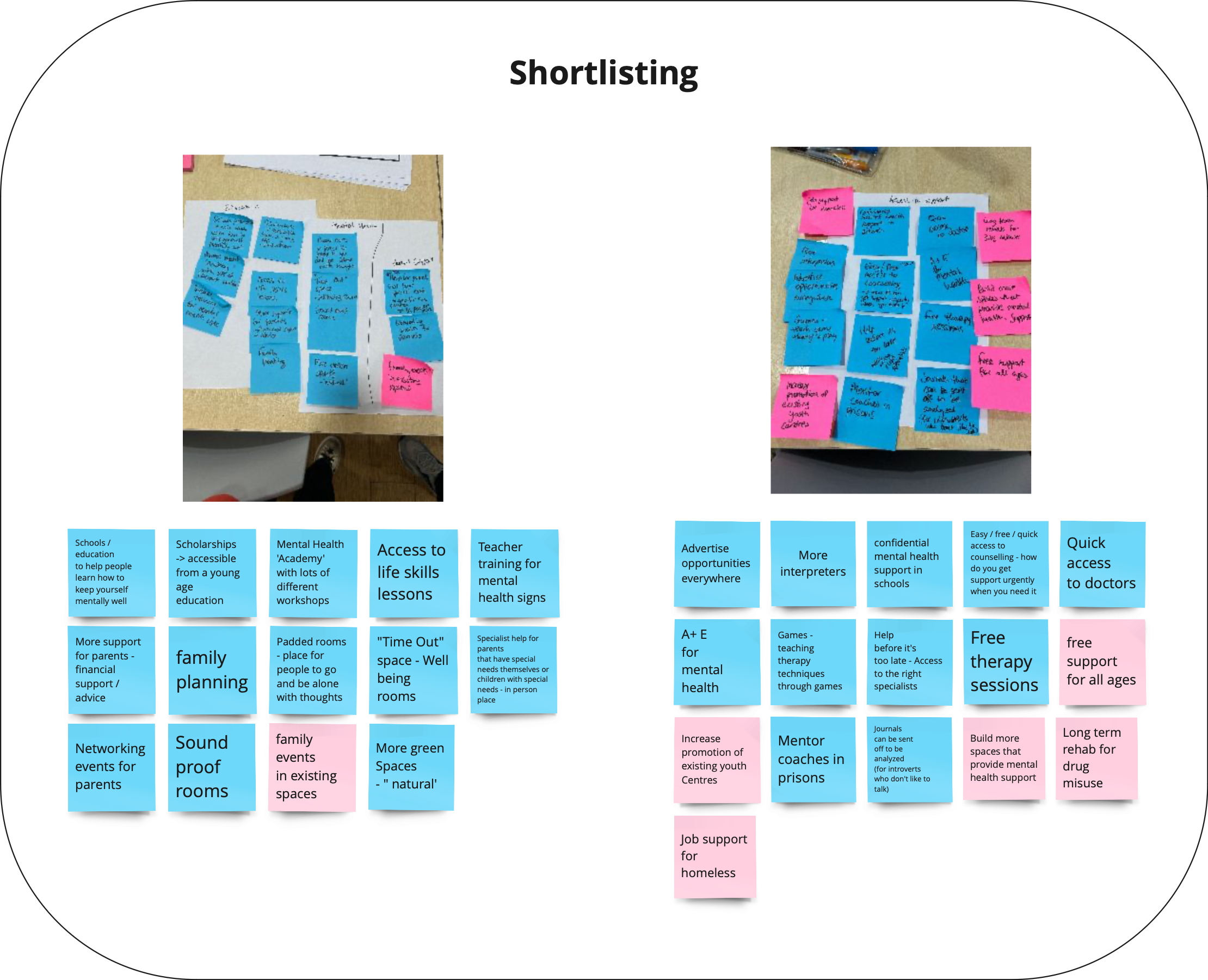**  Example: Flowchart to determine what to take forward for prototyping **(Newham)**  **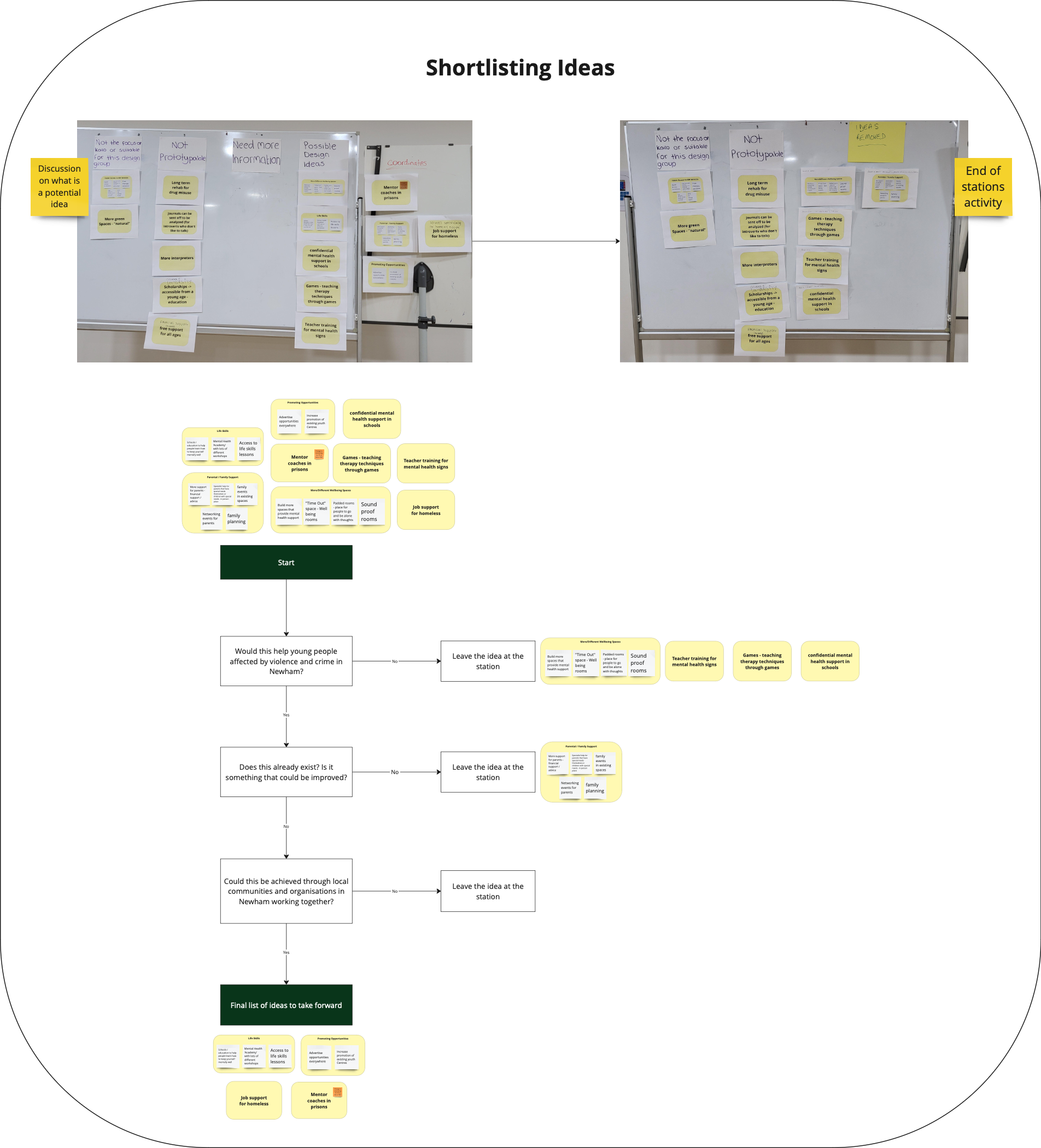** |
|  | **Vision: Persona Development Activity (Who, What, Why?)**  Creation of archetypes (Howard, 2014) who represent the intended key stakeholders in the design ideas i.e. ‘user-centred design’ [19, 20] and therefore can be used by the Small Circle participants to test their design ideas on and consider key features and elements that need to be incorporated into the prototypes [21]. This activity was facilitated by the following set of questions:   - Who are they? - What do they do? - What kind of person? - Why do young people interact with them?   **Vision: Mapping the journey**  This activity focused on further developing the vision of the design opportunity through the Small Circle participants thinking about the young person’s/stakeholder's experience through the potential intervention/ solution/ change via experience or journey mapping (Nielsen and Bruselius-Jensen, 2021; Howard, 2014)):  . 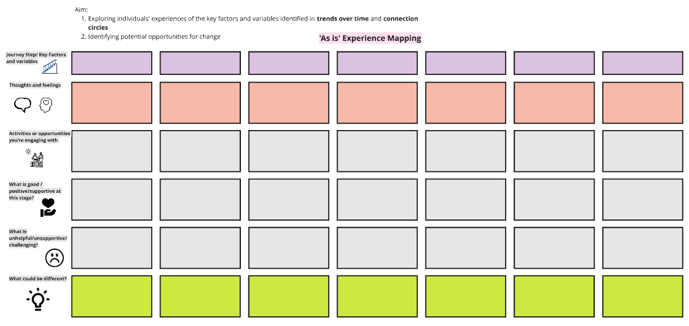  This activity enabled the Small Circle participants to consider where the touchpoints, obstacles and barriers may be within their design idea [22]  Analysis of these activities was completed outside of the sessions by the Kailo Team and community researchers and led to the development of a list of key outcome areas | - Persona development activities   **Examples shown from Northern Devon**  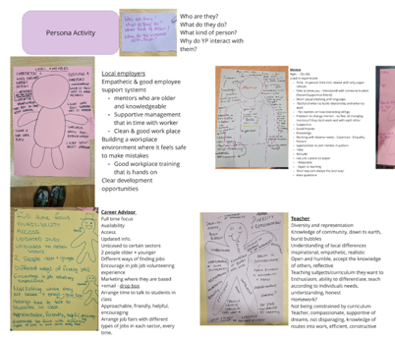  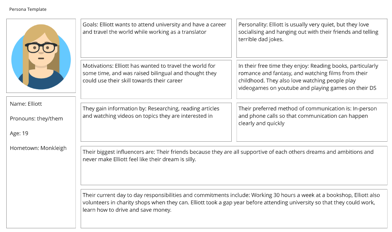  **Examples of persona development from Newham:**  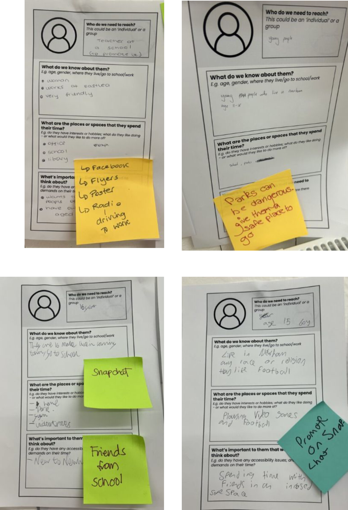   - Journey Map of young person/ user experience   **Example: Completed Journey Map from Bideford Small Circle:**  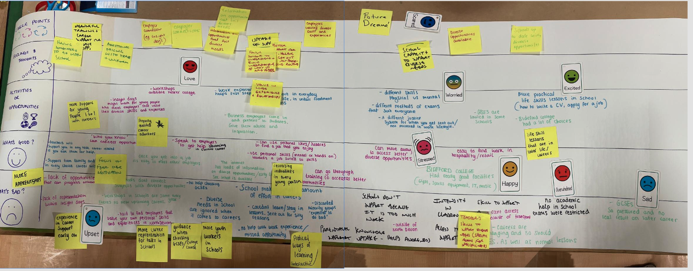  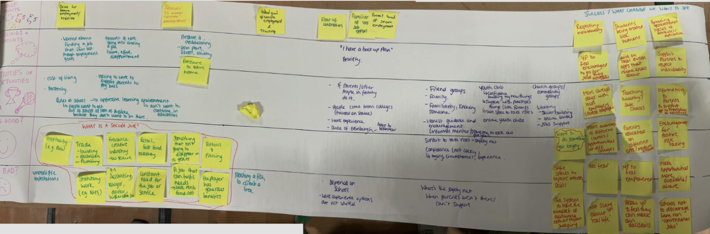  **Example: Vision for the diverse opportunities OA (Bideford) (compiled from persona development and journey mapping activities):**  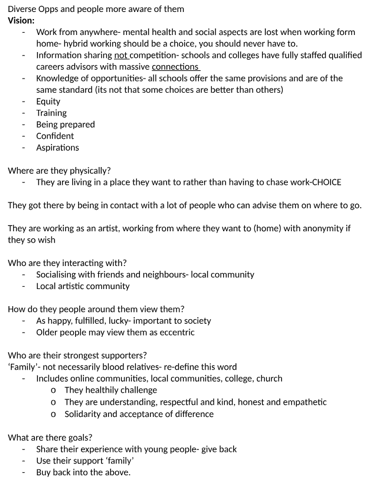  **Example: Analysis of the vision activities completed for Northern Devon (analysed by the Kailo Team and community researchers)**  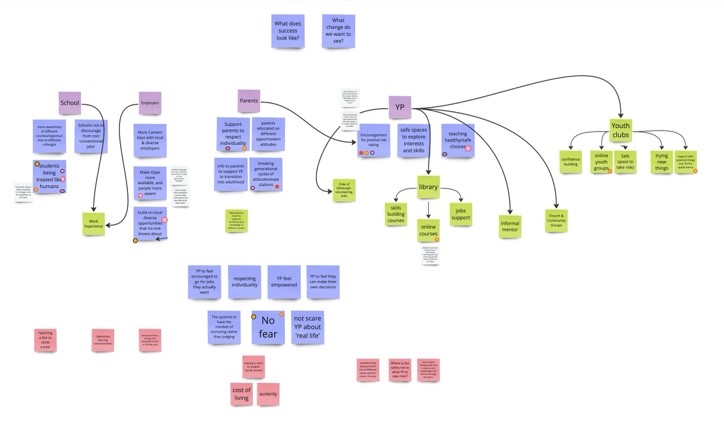  **Example: Key Outcome Areas from the thematic analysis completed by community researchers and Kailo team (Northern Devon)**  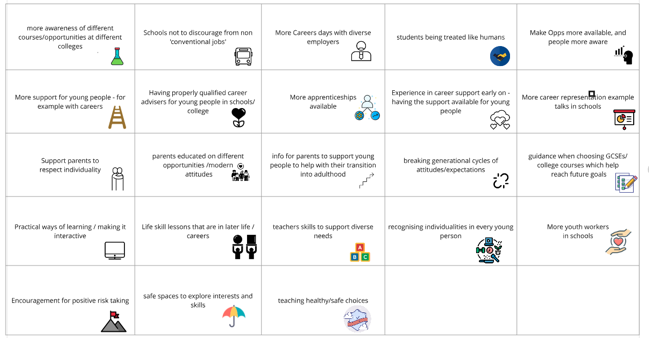 |
|  | **Northern Devon Specific: Joint Small Circle session**  Both the Small Circles came together to:   - Showcase their work (**Use of concept boards to present, highlight, and outline design ideas and interventions in individual groups)**, - Better consider diverse experiences of their design ideas and potential barriers and enablers through persona creation activities [23], and - Gain feedback across the two Small Circles as to any improvements that could be made to their design ideas.   **Example feedback questions:**  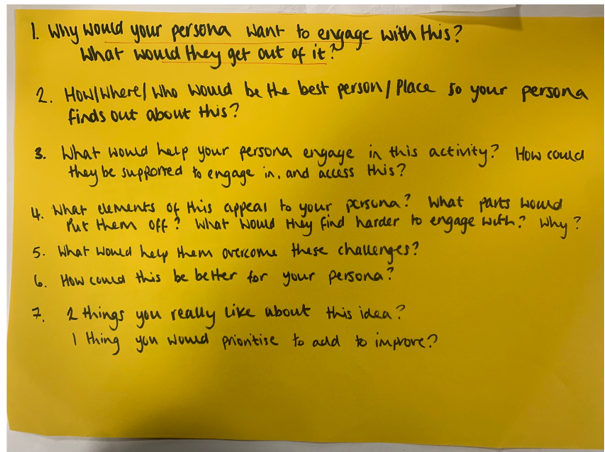 | - Concept boards   **Example:**  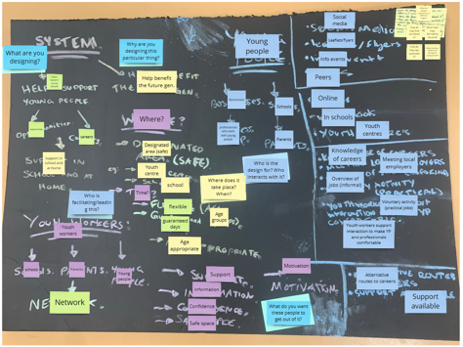   - Refinement of design ideas and consideration of diverse perspectives   **Example feedback**:  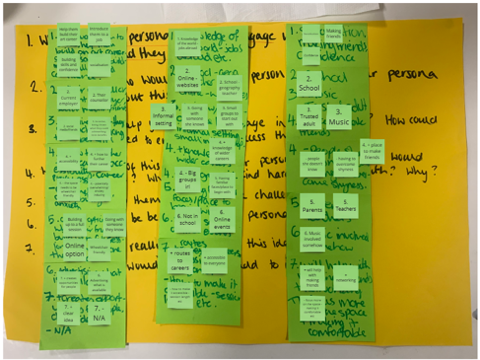   - Potential personal benefits for young people within the Small Circles i.e. pride in their work and participation within co-design activities have been seen to have personal benefits for young people [16] |
|  | **Developing Prototypes and Blueprints**  **Prototypes**  This activity focused on developing prototypes of specific elements of the design (that were surfaced in previous activities) to test within the Small Circle. By developing these prototypes and presenting them back within the group space, Small Circle participants were able to discuss the key elements of their design ideas, the experience of their intended stakeholder, and what they felt was necessary within the design opportunity for it to function as expected or planned [24].  **Example activity: Headline Challenge**  **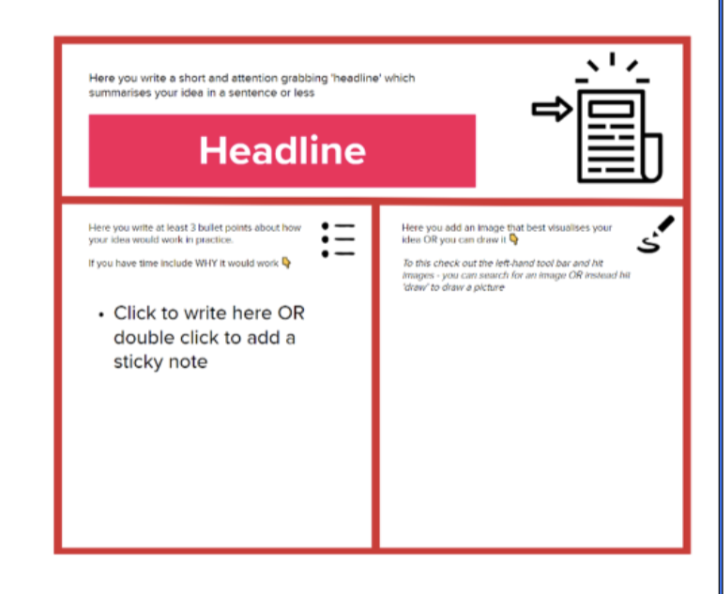**  Feedback on these prototypes allowed further refinement of the elements so they aligned better with the vision for the design opportunity and young person/user experience.  **Blueprints**  Through these discussions Service Blueprints could be developed which provides a visual representation of the design idea, the key touchpoints and stages, and the essential elements and components needed to make the service function as planned [24]. | - Prototypes of elements of the design   **Example: Headline challenge example (Newham)**  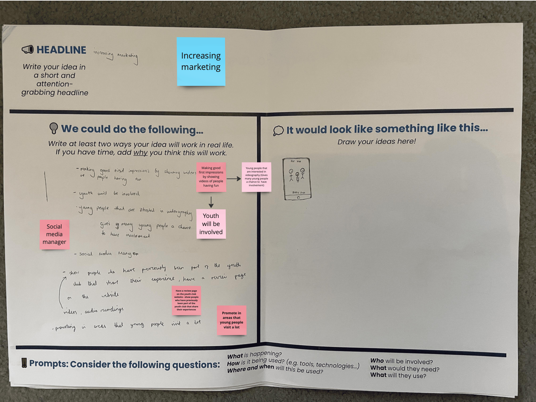  **Example: Blueprint for Diverse Opportunities (Northern Devon)**  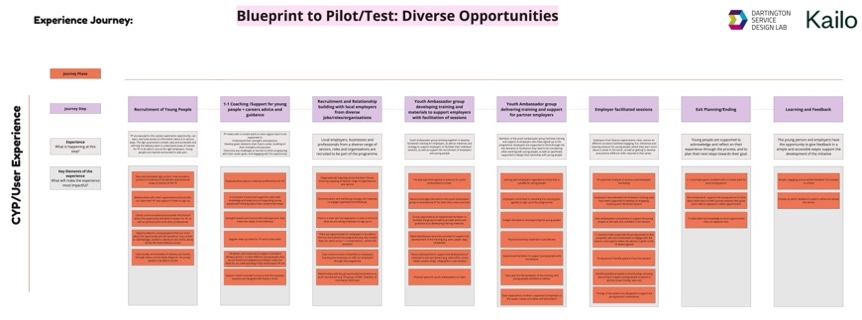 |
|  | **Example activity (Newham): ‘Dragons Den Pitch’**   - **Creation of a prototype idea pitch that the young people then presented to each other, receiving feedback and questions on each other’s ideas** | **Example: Dragons Den Pitch (Newham):**  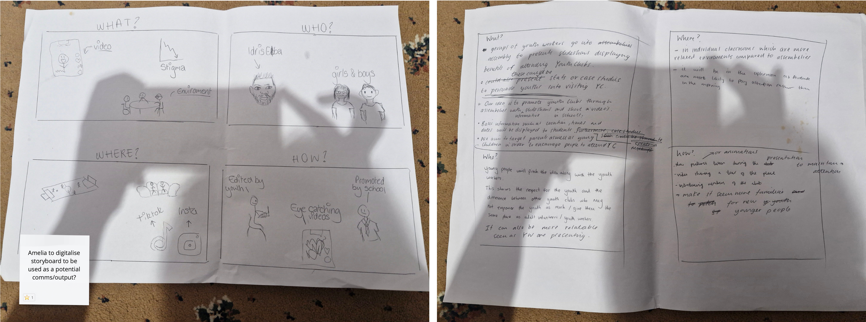 |
|  | **Refinement of outputs to support implementation**  Through the feedback collated across the sessions refinement of the outputs to support implementation could be made. A journey map using these refined outputs was created which the Small Circle participants felt best reflected the young person’s experience through the proposed intervention/solution/ change. | - Refined Journey Map of young person’s experience |
|  | **Example (Bideford):**   - **Visualisation of how design outputs can be shared and embedded within the local community** - **Planning for different sessions being carried out across different sectors, highlighting who the target group are, the key focus of the session and how it’s to be delivered** | 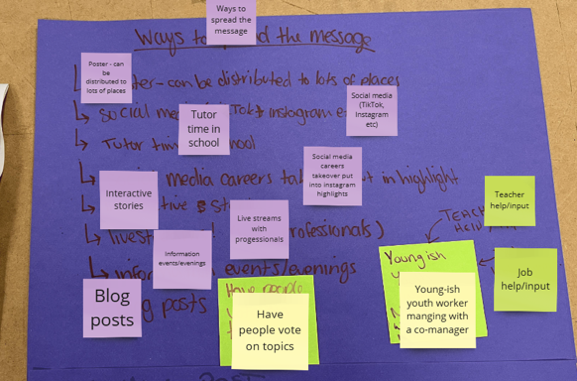  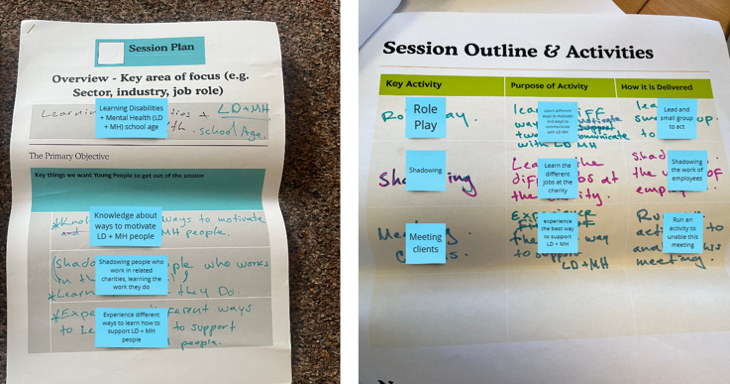  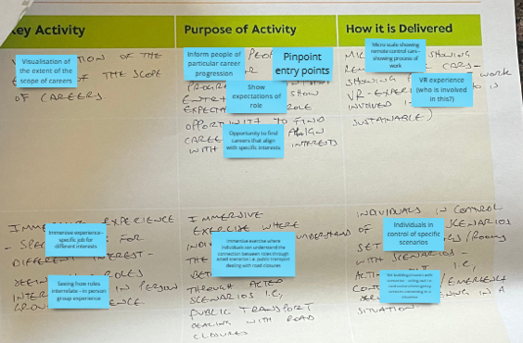 |
|  | **Example: Idea posters (Newham)**   - **Development of a prioritised idea through considering ‘who, what, where, when’ questions** | 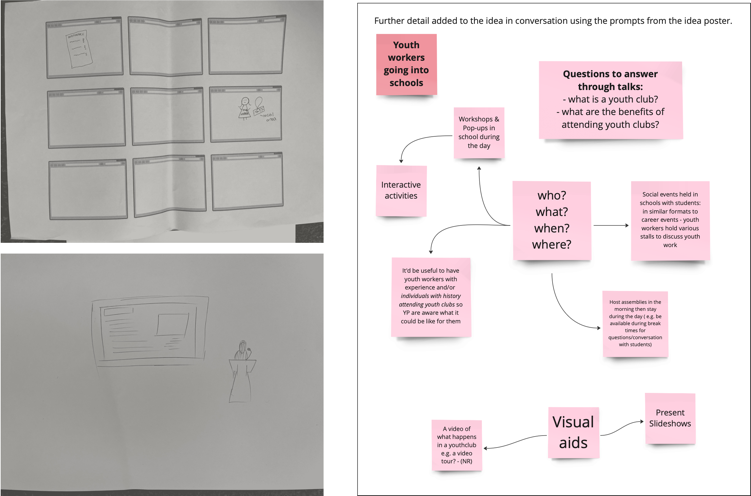 |
|  | **Example: Designing a marketing campaign strategy (Newham)**   - **Connect young people’s ideas to a wider marketing campaign** - **Considering ‘What we want to say’ and ‘what we others to feel’** | 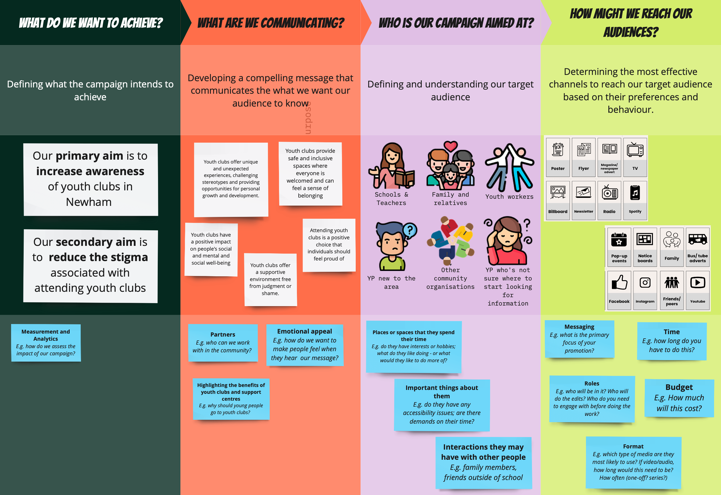 |
|  | **Celebration**  The final session of the codesign process focused on creating blueprints of the strategies to support young people’s mental health in the local area, based on the site-specific OAs  Activities within this session included:   - Free writing their ‘Journey of Kailo’   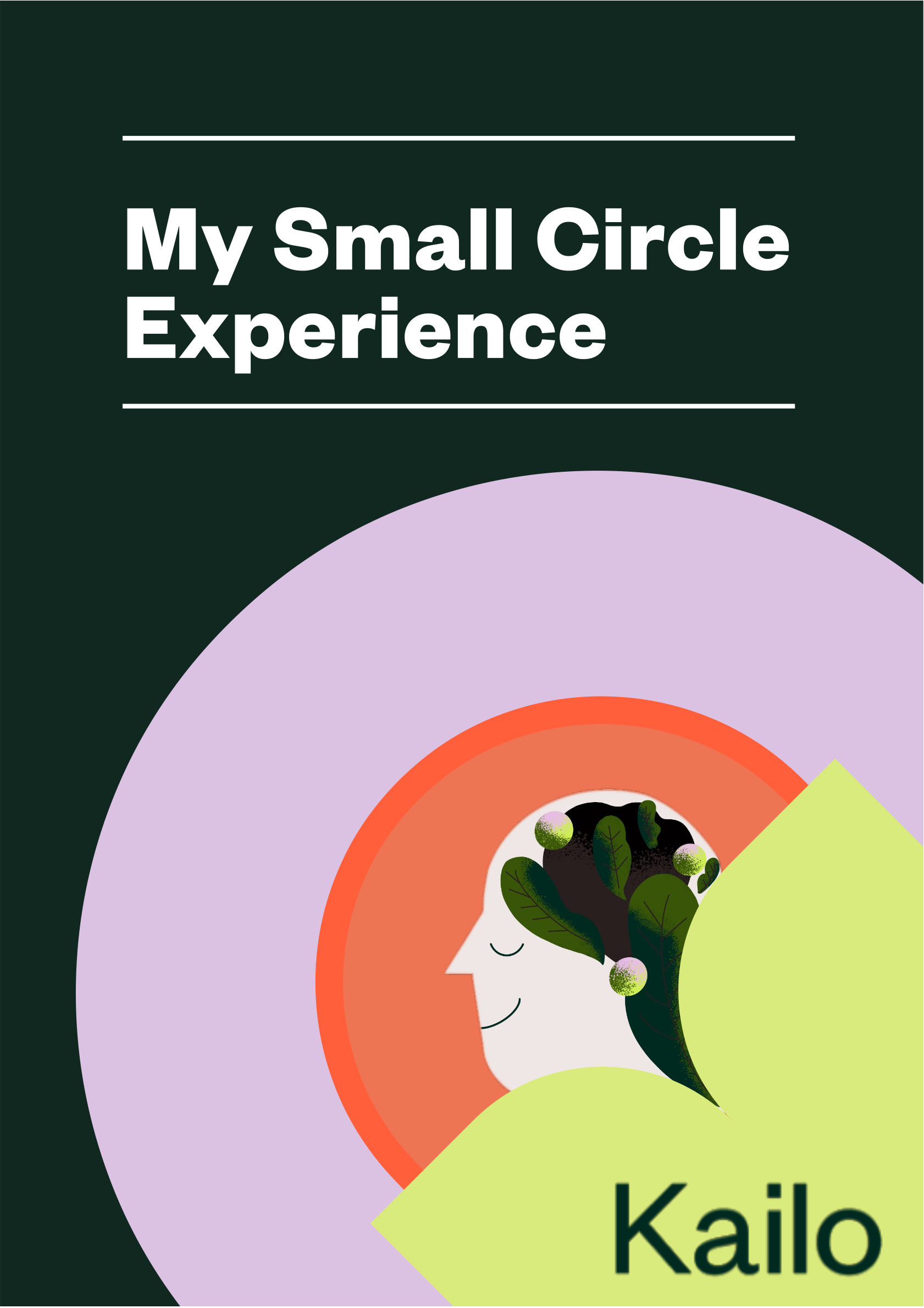 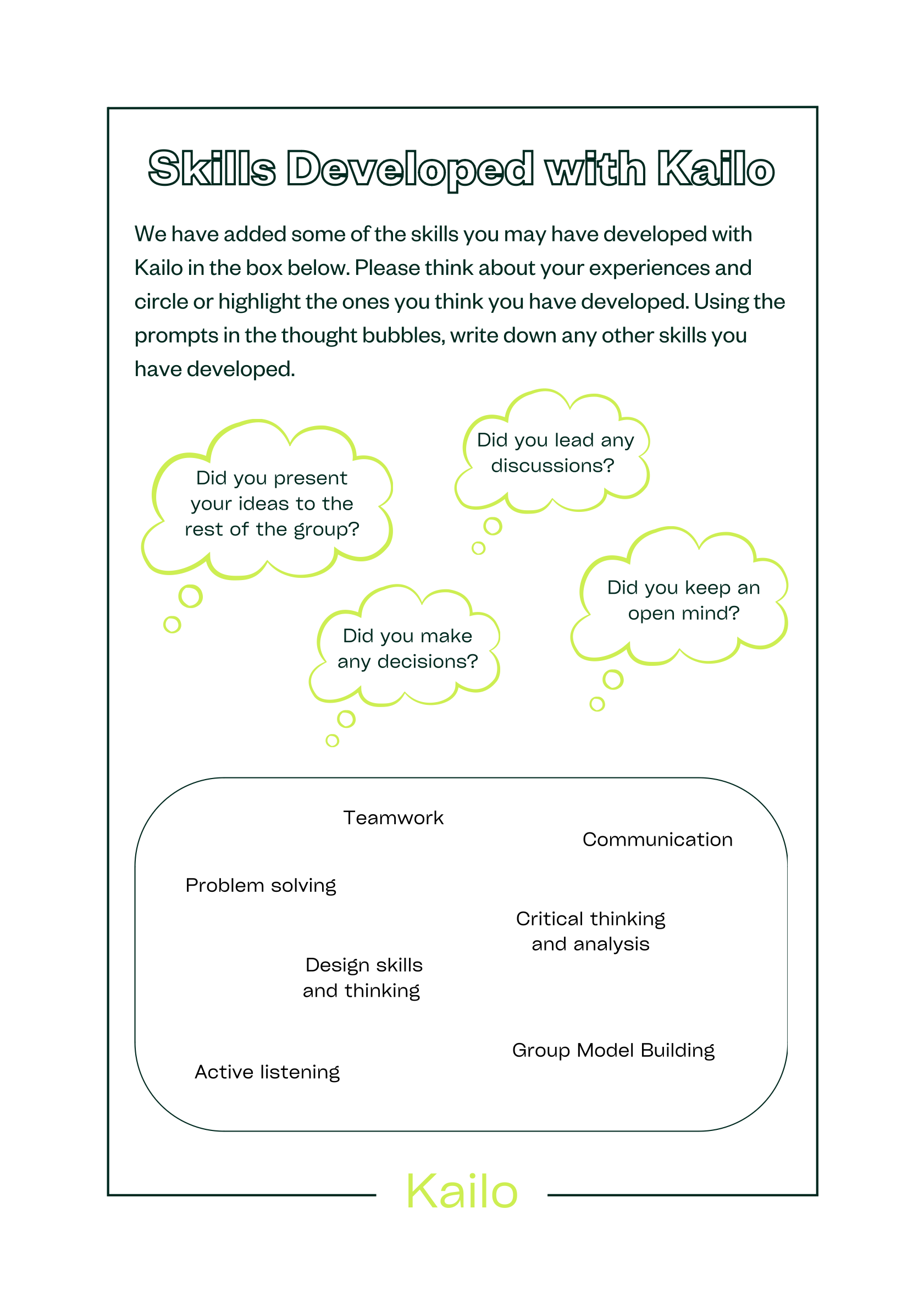   - Stars and Hearts (**to assess** **what the young people were proud of relating to themselves, the group, and one other person in the room)**   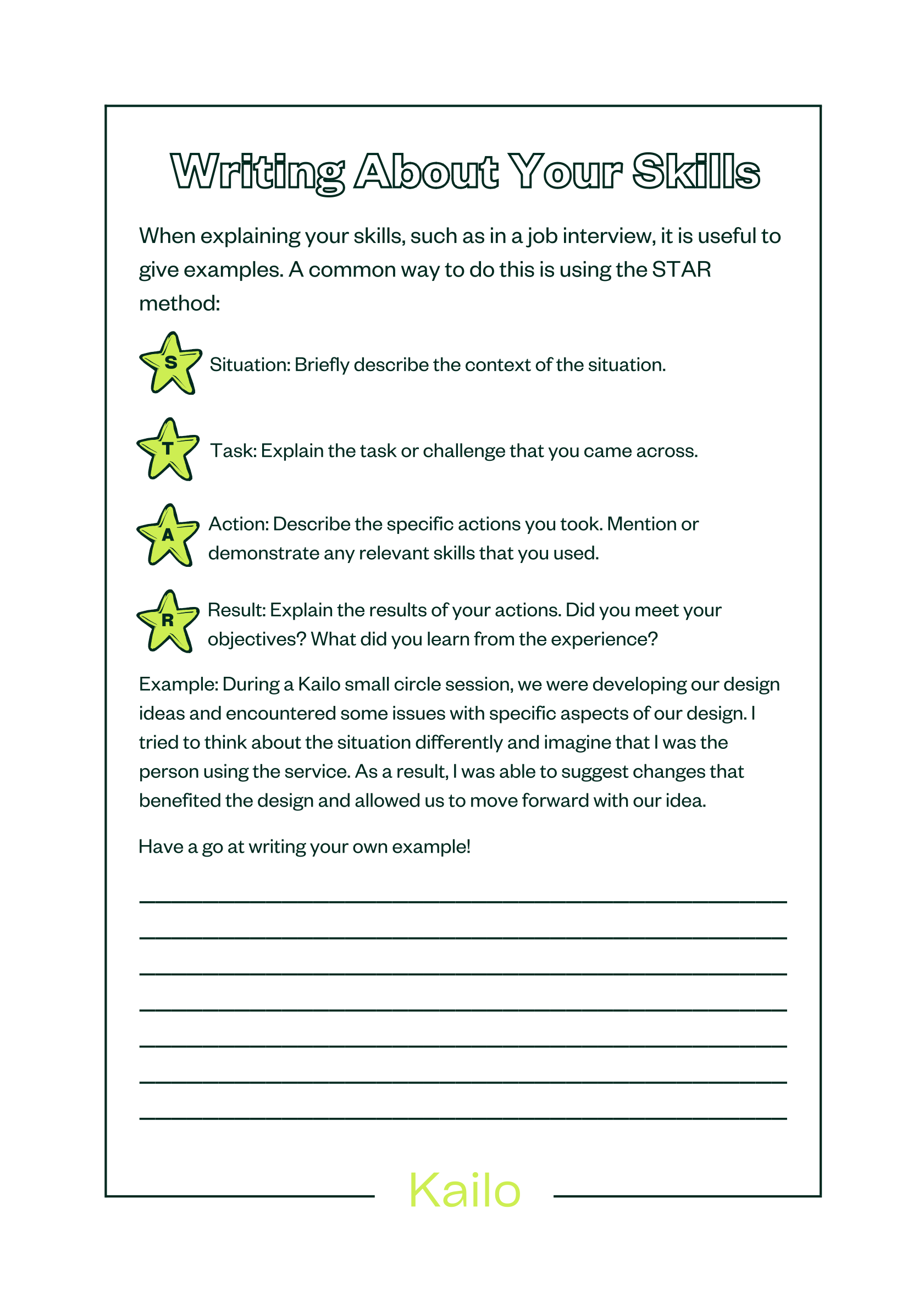   - Reflective practice methods (comparing how the Young People felt at the start and end of the design process, collectively creating a timeline of key moments)   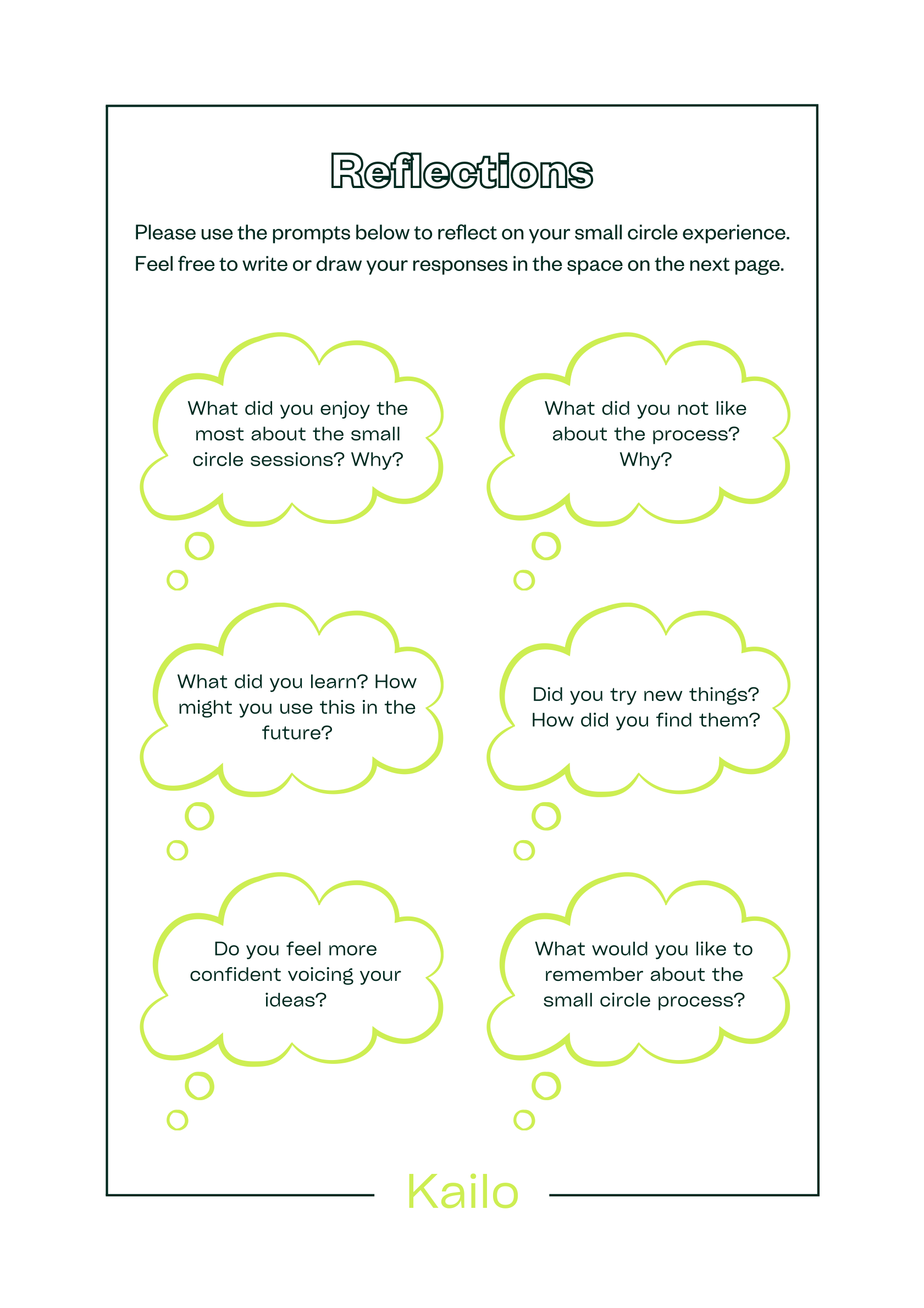  The evaluation team also conducted interviews with the young people who participated in the Small Circles about their experience of the codesign process. | - Strategies to support young people’s mental health in the local area based on OAs - Feedback from young people about their experience of being a Small Circle participant and being part of the co-design process.   **Example:** **The Stars and Hearts exercise (Northern Devon)**:  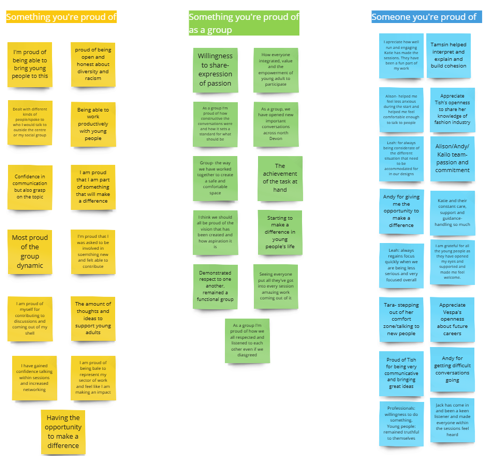 |
|  | **Example activity: Young people presenting their Small Circle experience of the codesign process to community partners (Newham)**   - Creation of narrative/story boards to be used within their presentations | - **Example: Narrative story boards of Small Circle participants experience of the Kailo codesign process**   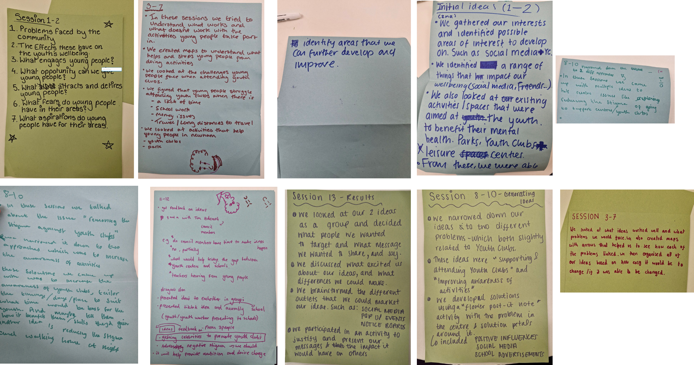 |

**References**

1. Martínez- Mesa J, González-Chica D, Duquia R, Bonamigo R, Bastos J. Sampling: How to Select Participants in My Research study? Anais Brasileiros De Dermatologia [Internet]. 2016 [cited 2024 Aug 1];91(3):326–30. Available from: <https://www.ncbi.nlm.nih.gov/pmc/articles/PMC4938277/>
2. Seed for Change. Group Agreements a Short Guide to Creating Group Agreements for Workshops and Meetings Seeds for Change Short Guide [Internet]. 2021 [cited 2024 Aug 1]. Available from: <https://www.seedsforchange.org.uk/downloads/groupagree.pdf>
3. Luna-Reyes LF, Martinez-Moyano IJ, Pardo TA, Cresswell AM, Andersen DF, Richardson GP. Anatomy of a Group model-building intervention: Building Dynamic Theory from Case Study Research. System Dynamics Review [Internet]. 2006 [cited 2024 Aug 1];22(4):291–320. Available from: <https://onlinelibrary.wiley.com/doi/abs/10.1002/sdr.349>
4. Andersen DF, Richardson GP. Scripts for Group Model Building. System Dynamics Review [Internet]. 1997 [cited 2020 Jun 1];13(2):107–29. Available from: <https://onlinelibrary.wiley.com/doi/abs/10.1002/%28SICI%291099-1727%28199722%2913%3A2%3C107%3A%3AAID-SDR120%3E3.0.CO%3B2-7>
5. Anna Freud Centre. An Evidence Briefing on Activities Available outside School Settings for Young People [Internet]. brandplatform.annafreud.org. 2024 [cited 2024 Aug 1]. Available from: <https://brandplatform.annafreud.org/share/WwebLCcamsUFtWcW4WfE>
6. Cohen J. Scriptapedia/Connection Circle - Wikibooks, Open Books for an Open World [Internet]. Wikibooks.org. 2022 [cited 2024 Aug 6]. Available from: <https://en.wikibooks.org/wiki/Scriptapedia/Connection_Circle>
7. Hovmand P, Kraus A. Scriptapedia/Creating Causal Loop Diagram from Connection Circles - Wikibooks, Open Books for an Open World [Internet]. Wikibooks.org. 2022 [cited 2024 Aug 6]. Available from: <https://en.wikibooks.org/wiki/Scriptapedia/Creating_Causal_Loop_Diagram_from_Connection_Circles>
8. Meadows DH. Thinking in systems: a Primer. Illustrated edition. Wright D, editor. White River Junction, Vermont: Chelsea Green Publishing; 2008.
9. Meadows D. Leverage Points: Places to Intervene in a System [Internet]. The Academy for Systems Change. 1999 [cited 2024 Aug 6]. Available from: <https://donellameadows.org/archives/leverage-points-places-to-intervene-in-a-system/>
10. This is Service Design Doing. #TiSDD Method: Mood Boards [Internet]. www.thisisservicedesigndoing.com. 2024 [cited 2024 Aug 1]. Available from: <https://www.thisisservicedesigndoing.com/methods/mood-boards>
11. Design Method Toolkit. Design Method Toolkit Dot Voting [Internet]. toolkits.dss.cloud. 2024 [cited 2024 Aug 1]. Available from: <https://toolkits.dss.cloud/design/method-card/dot-voting/>
12. Hyper Island Toolbox. Dotmocracy [Internet]. HI Toolbox. 2024 [cited 2024 Aug 1]. Available from: <https://toolbox.hyperisland.com/dotmocracy>
13. Anna Freud Centre. An Evidence Briefing on Young people’s Access to Employment in Rural Communities [Internet]. brandplatform.annafreud.org. 2024 [cited 2024 Aug 1]. Available from: <https://brandplatform.annafreud.org/share/gBUc9fEtLQa32fPsUFJy>
14. Hyper Island Toolbox. How Might We Questions [Internet]. HI Toolbox. 2024 [cited 2024 Aug 1]. Available from: <https://toolbox.hyperisland.com/how-might-we-questions>
15. Skarlatidou A, Suskevics M, Göbel C, Prūse B, Tauginiené L, Mascarenhas A, et al. The Value of Stakeholder Mapping to Enhance Co-Creation in Citizen Science Initiatives. Citizen Science: Theory and Practice [Internet]. 2019 [cited 2024 Aug 1];4(1). Available from: <https://discovery.ucl.ac.uk/id/eprint/10080369/1/Skarlatidou%20et%20al.%20_%202019_The%20Value%20of%20Stakeholder%20Mapping%20to%20Enhance%20Co-Creation%20in%20Citizen%20Science%20Initiatives.pdf>
16. Vella-Brodrick D, Patrick K, Jacques-Hamilton R, Ng A, Chin TC, O’Connor M, et al. Youth Experiences of co-designing a well-being intervention: reflections, Learnings and Recommendations. Oxford Review of Education [Internet]. 2023 May 2 [cited 2024 Aug 1];1–20. Available from: <https://www.tandfonline.com/doi/full/10.1080/03054985.2023.2194621>
17. Design Method Toolkit. Design Method Toolkit Lotus Blossom [Internet]. toolkits.dss.cloud. 2024 [cited 2024 Aug 1]. Available from: <https://toolkits.dss.cloud/design/method-card/lotus-blossom-2/>
18. Hyper Island Toolbox. World Cafe [Internet]. 2024 [cited 2024 Aug 1]. Available from: <https://toolbox.hyperisland.com/world-cafe>
19. LeRouge C, Ma J, Sneha S, Tolle K. User Profiles and Personas in the Design and Development of Consumer Health Technologies. International Journal of Medical Informatics [Internet]. 2019 Nov [cited 2024 Aug 1];82(11):e251–68. Available from: <https://www.sciencedirect.com/science/article/abs/pii/S1386505611000724>
20. Service Design Tools. Personas | Service Design Tools [Internet]. servicedesigntools.org. 2024 [cited 2024 Aug 1]. Available from: <https://servicedesigntools.org/tools/personas>
21. Salminen J, Wenyun Guan K, Jung SG, Jansen B. Use Cases for Design Personas: A Systematic Review and New Frontiers. CHI Conference on Human Factors in Computing Systems [Internet]. 2022 Apr 29 [cited 2024 Aug 1]; Available from: <https://dl.acm.org/doi/pdf/10.1145/3491102.3517589>
22. Service Design Tools. Journey Map | Service Design Tools [Internet]. Servicedesigntools.org. 2017 [cited 2024 Aug 1]. Available from: <https://servicedesigntools.org/tools/journey-map>
23. Dam R, Siang T. Personas – a Simple Introduction [Internet]. The Interaction Design Foundation. 2019 [cited 2024 Aug 6]. Available from: <https://www.interaction-design.org/literature/article/personas-why-and-how-you-should-use-them>
24. He F. An Introduction to Service Design and a Selection of Service Design Tools Design Methods for Developing Services. wwwacademiaedu [Internet]. 2024 [cited 2024 Aug 1]; Available from: <https://www.academia.edu/24477788/An_introduction_to_service_design_and_a_selection_of_service_design_tools_Design_methods_for_developing_services>
