# Supplementary material for: An adaptive, youth-centred co-design methodology: place-based co-design centring youth and community participation
Source: Res Involv Engagem. 2026 Jan 24;12:33. doi: 10.1186/s40900-025-00833-w (PMC12994241; doi:10.1186/s40900-025-00833-w)
Supplement: Supplementary file 7 — Supplementary Material 7 [file 40900_2025_833_MOESM7_ESM.pdf]

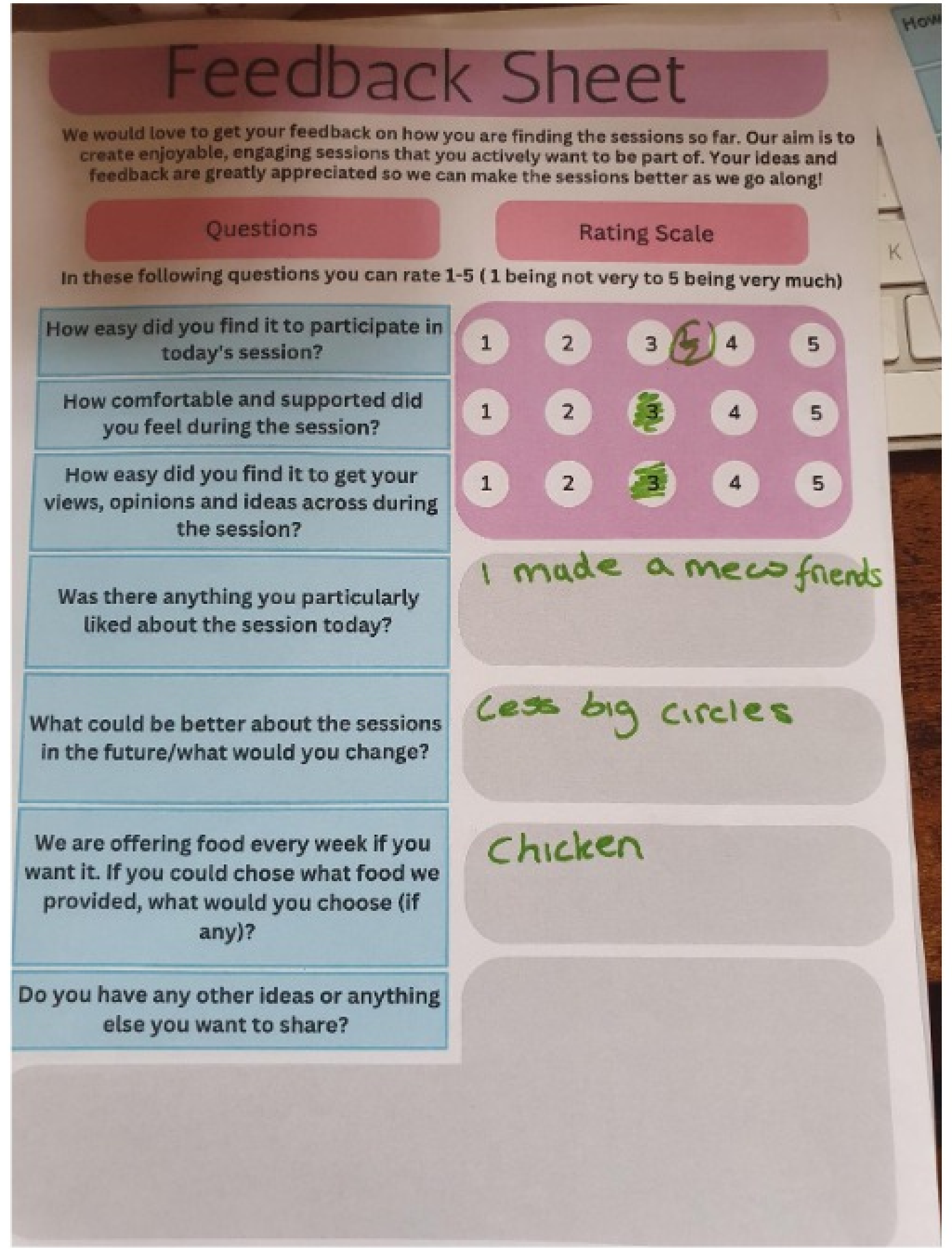

Feedback Sheet

We would love to get your feedback on how you are finding the sessions so far. Our aim is to create enjoyable, engaging sessions that you actively want to be part of. Your ideas and feedback are greatly appreciated so we can make the sessions better as we go along!

Questions	Rating Scale
How easy did you find it to participate in today's session?	1 2 3 4 5
How comfortable and supported did you feel during the session?	1 2 3 4 5
How easy did you find it to get your views, opinions and ideas across during the session?	1 2 3 4 5
Was there anything you particularly liked about the session today?	I made a new friends
What could be better about the sessions in the future/what would you change?	Less big circles
We are offering food every week if you want it. If you could chose what food we provided, what would you choose (if any)?	Chicken
Do you have any other ideas or anything else you want to share?	

In these following questions you can rate 1-5 (1 being not very to 5 being very much)

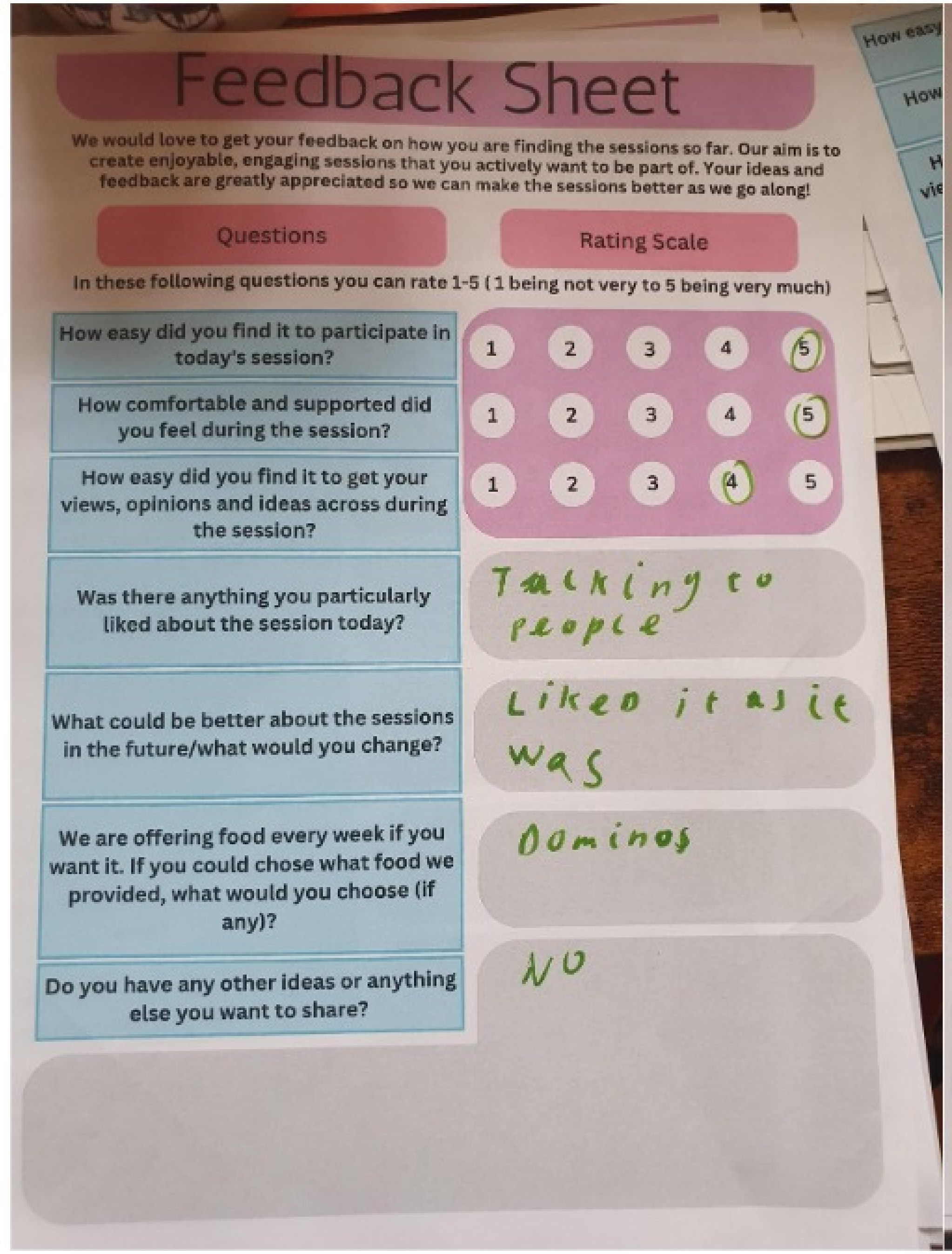

Feedback Sheet

We would love to get your feedback on how you are finding the sessions so far. Our aim is to create enjoyable, engaging sessions that you actively want to be part of. Your ideas and feedback are greatly appreciated so we can make the sessions better as we go along!

In these following questions you can rate 1-5 (1 being not very to 5 being very much)

Questions	Rating Scale
How easy did you find it to participate in today's session?	1 2 3 4 5
How comfortable and supported did you feel during the session?	1 2 3 4 5
How easy did you find it to get your views, opinions and ideas across during the session?	1 2 3 4 5
Was there anything you particularly liked about the session today?	Talking to people
What could be better about the sessions in the future/what would you change?	Liked it as it was
We are offering food every week if you want it. If you could chose what food we provided, what would you choose (if any)?	Dominos
Do you have any other ideas or anything else you want to share?	NO

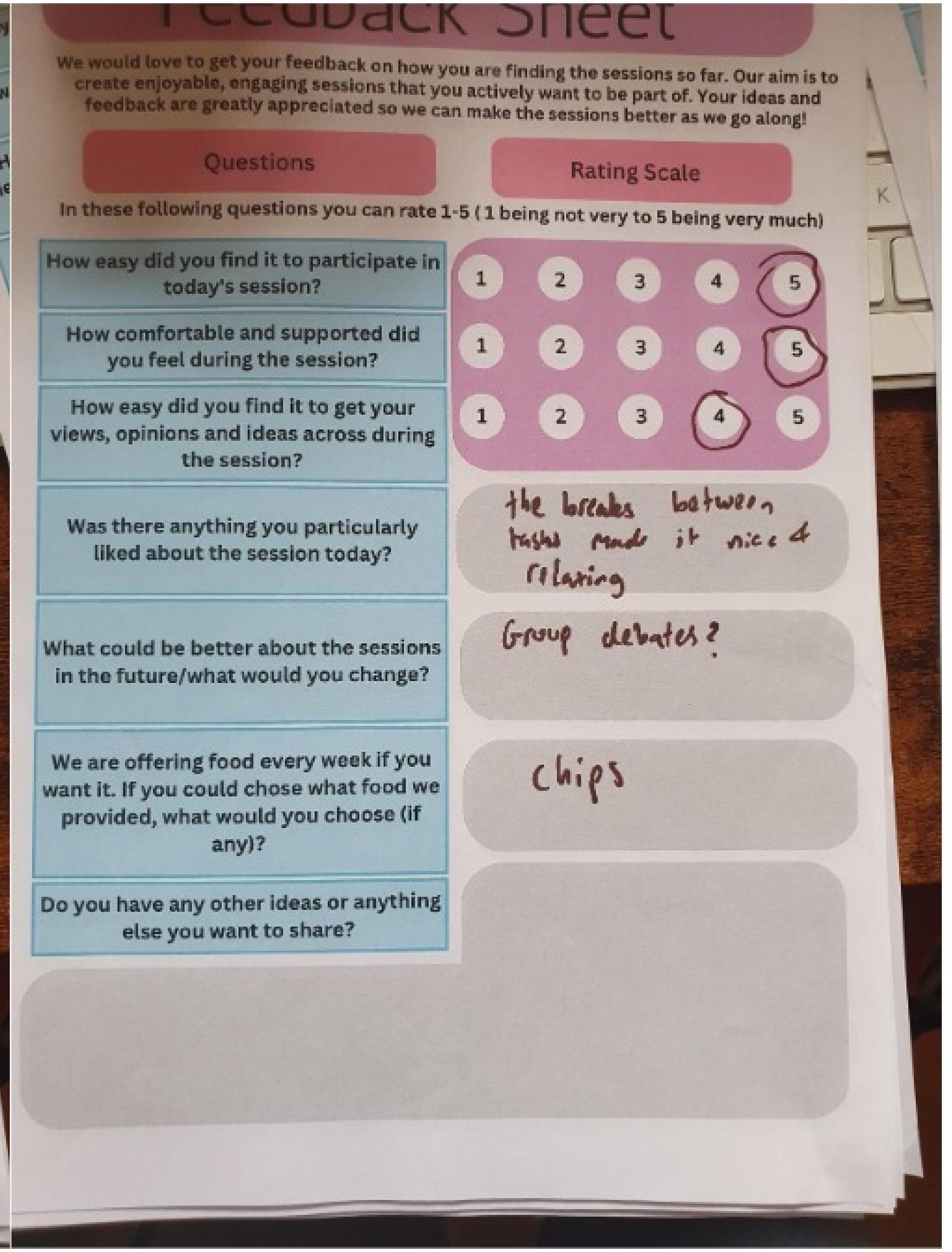

Feedback Sheet

We would love to get your feedback on how you are finding the sessions so far. Our aim is to create enjoyable, engaging sessions that you actively want to be part of. Your ideas and feedback are greatly appreciated so we can make the sessions better as we go along!

In these following questions you can rate 1-5 (1 being not very to 5 being very much)

Questions	Rating Scale
How easy did you find it to participate in today's session?	1 2 3 4 5
How comfortable and supported did you feel during the session?	1 2 3 4 5
How easy did you find it to get your views, opinions and ideas across during the session?	1 2 3 4 5
Was there anything you particularly liked about the session today?	the breaks between tasks made it nice & relaxing
What could be better about the sessions in the future/what would you change?	Group debates?
We are offering food every week if you want it. If you could chose what food we provided, what would you choose (if any)?	chips
Do you have any other ideas or anything else you want to share?	

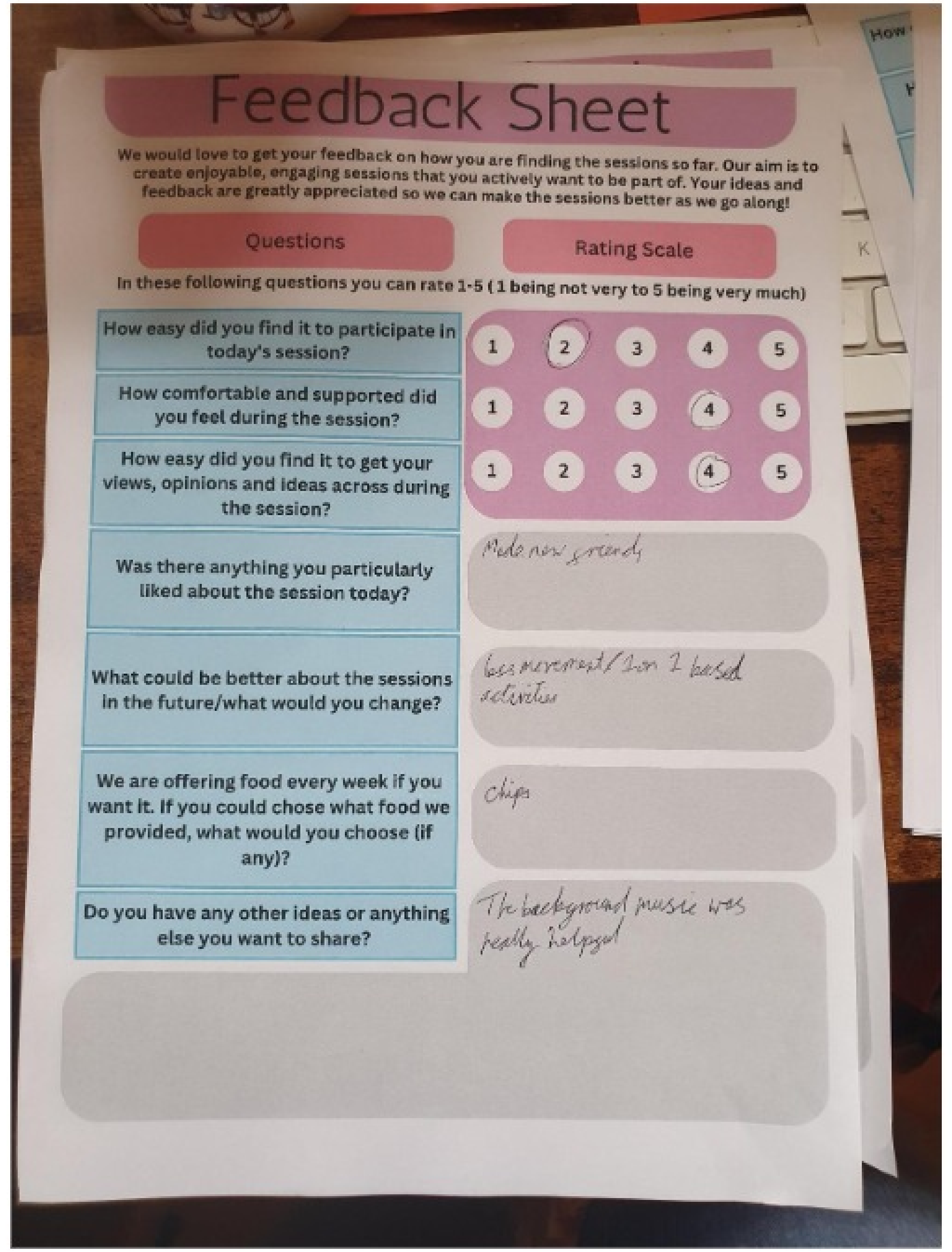

Feedback Sheet

We would love to get your feedback on how you are finding the sessions so far. Our aim is to create enjoyable, engaging sessions that you actively want to be part of. Your ideas and feedback are greatly appreciated so we can make the sessions better as we go along!

In these following questions you can rate 1-5 (1 being not very to 5 being very much)

Questions	Rating Scale
How easy did you find it to participate in today's session?	1 2 3 4 5
How comfortable and supported did you feel during the session?	1 2 3 4 5
How easy did you find it to get your views, opinions and ideas across during the session?	1 2 3 4 5
Was there anything you particularly liked about the session today?	Made new friends
What could be better about the sessions in the future/what would you change?	less movement/1 on 1 based activities
We are offering food every week if you want it. If you could chose what food we provided, what would you choose (if any)?	chips
Do you have any other ideas or anything else you want to share?	The background music was really helpful

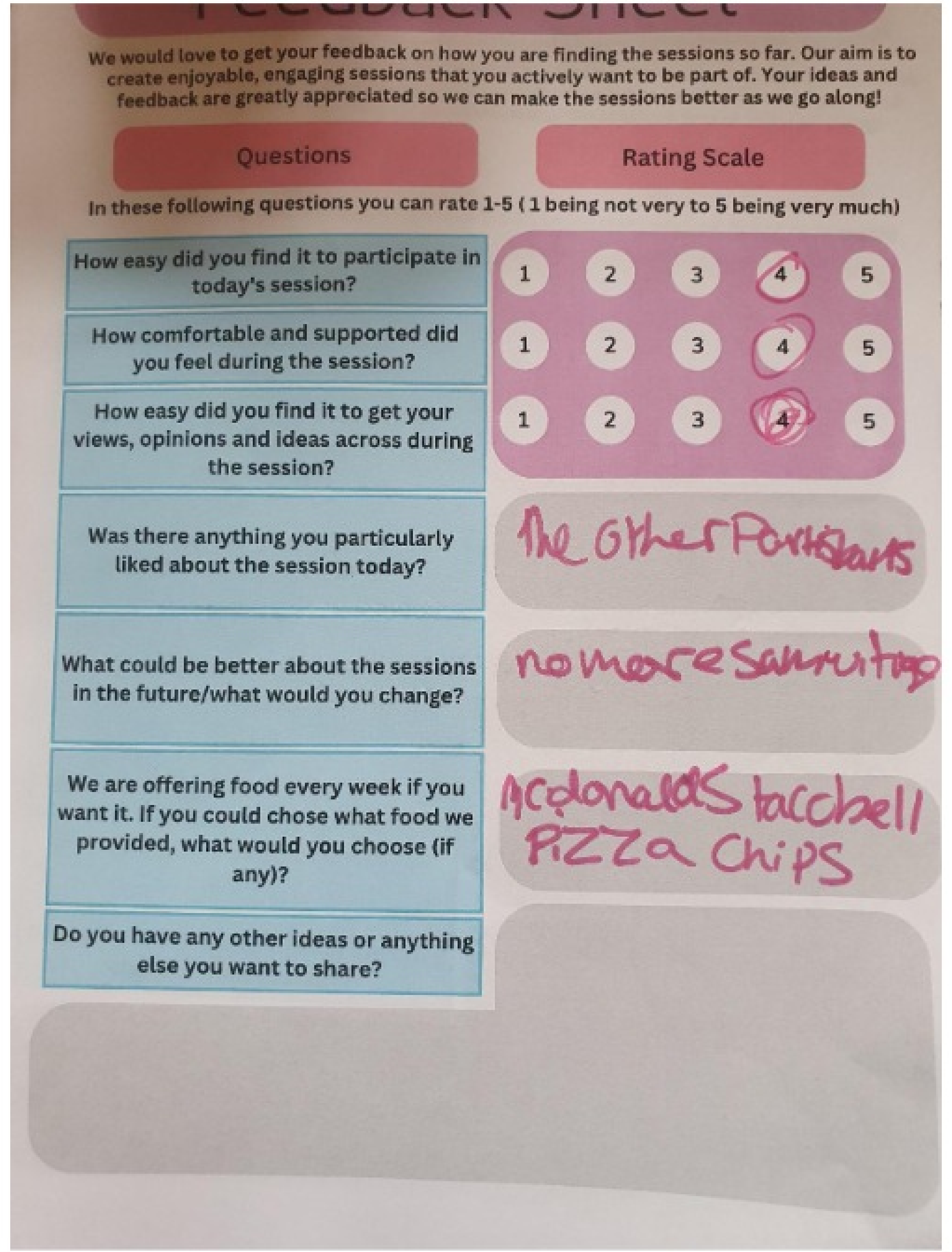

Feedback Sheet

We would love to get your feedback on how you are finding the sessions so far. Our aim is to create enjoyable, engaging sessions that you actively want to be part of. Your ideas and feedback are greatly appreciated so we can make the sessions better as we go along!

In these following questions you can rate 1-5 (1 being not very to 5 being very much)

Questions	Rating Scale
How easy did you find it to participate in today's session?	1 2 3 4 5
How comfortable and supported did you feel during the session?	1 2 3 4 5
How easy did you find it to get your views, opinions and ideas across during the session?	1 2 3 4 5
Was there anything you particularly liked about the session today?	The Other Participants
What could be better about the sessions in the future/what would you change?	no more [illegible]
We are offering food every week if you want it. If you could chose what food we provided, what would you choose (if any)?	Mcdonalds tacobell PIZZA Chips
Do you have any other ideas or anything else you want to share?	

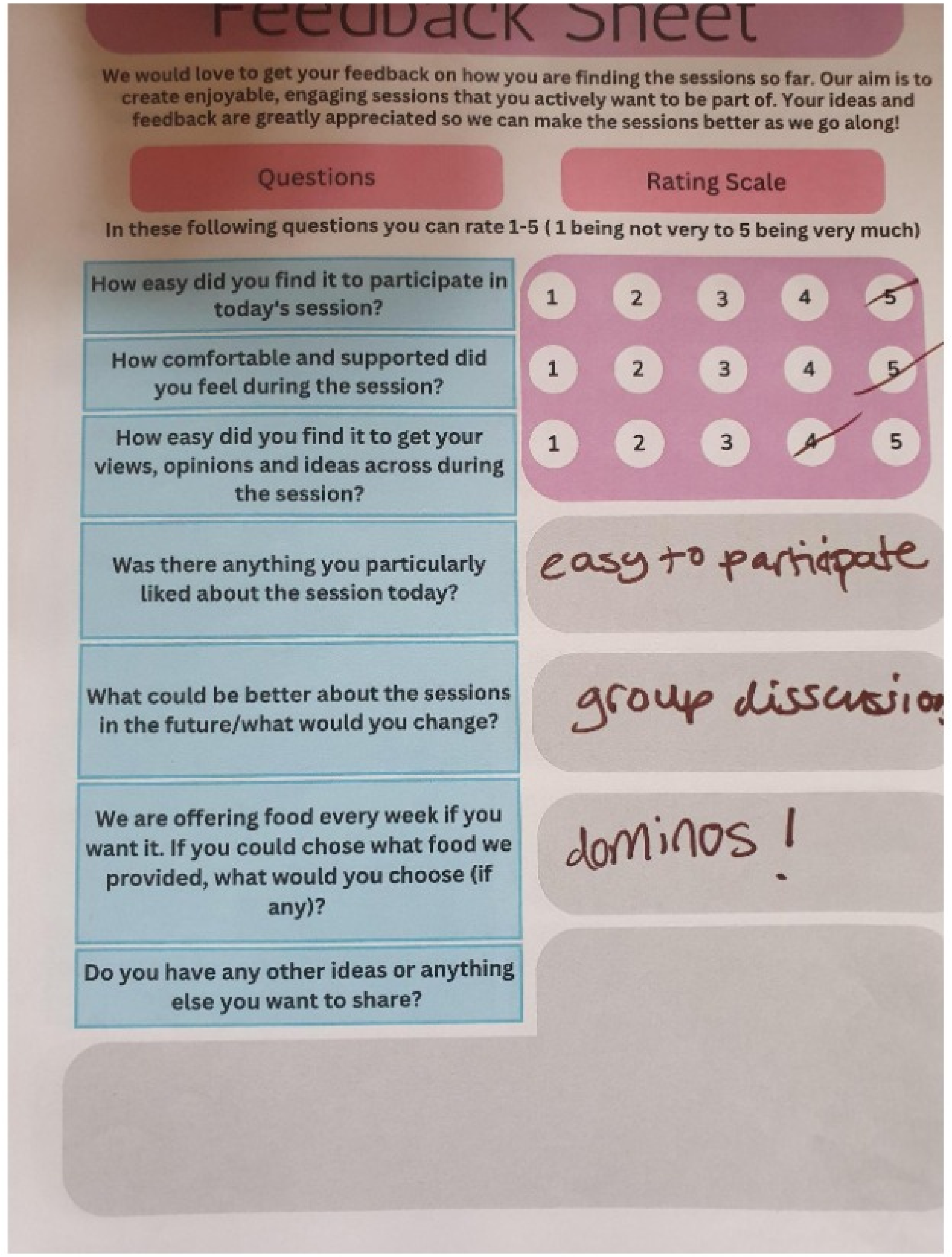

Feedback Sheet

We would love to get your feedback on how you are finding the sessions so far. Our aim is to create enjoyable, engaging sessions that you actively want to be part of. Your ideas and feedback are greatly appreciated so we can make the sessions better as we go along!

Questions	Rating Scale
In these following questions you can rate 1-5 (1 being not very to 5 being very much)	
How easy did you find it to participate in today's session?	1 2 3 4 **5**
How comfortable and supported did you feel during the session?	1 2 3 4 **5**
How easy did you find it to get your views, opinions and ideas across during the session?	1 2 3 **4** 5
Was there anything you particularly liked about the session today?	easy to participate
What could be better about the sessions in the future/what would you change?	group discussio
We are offering food every week if you want it. If you could chose what food we provided, what would you choose (if any)?	dominos !
Do you have any other ideas or anything else you want to share?	

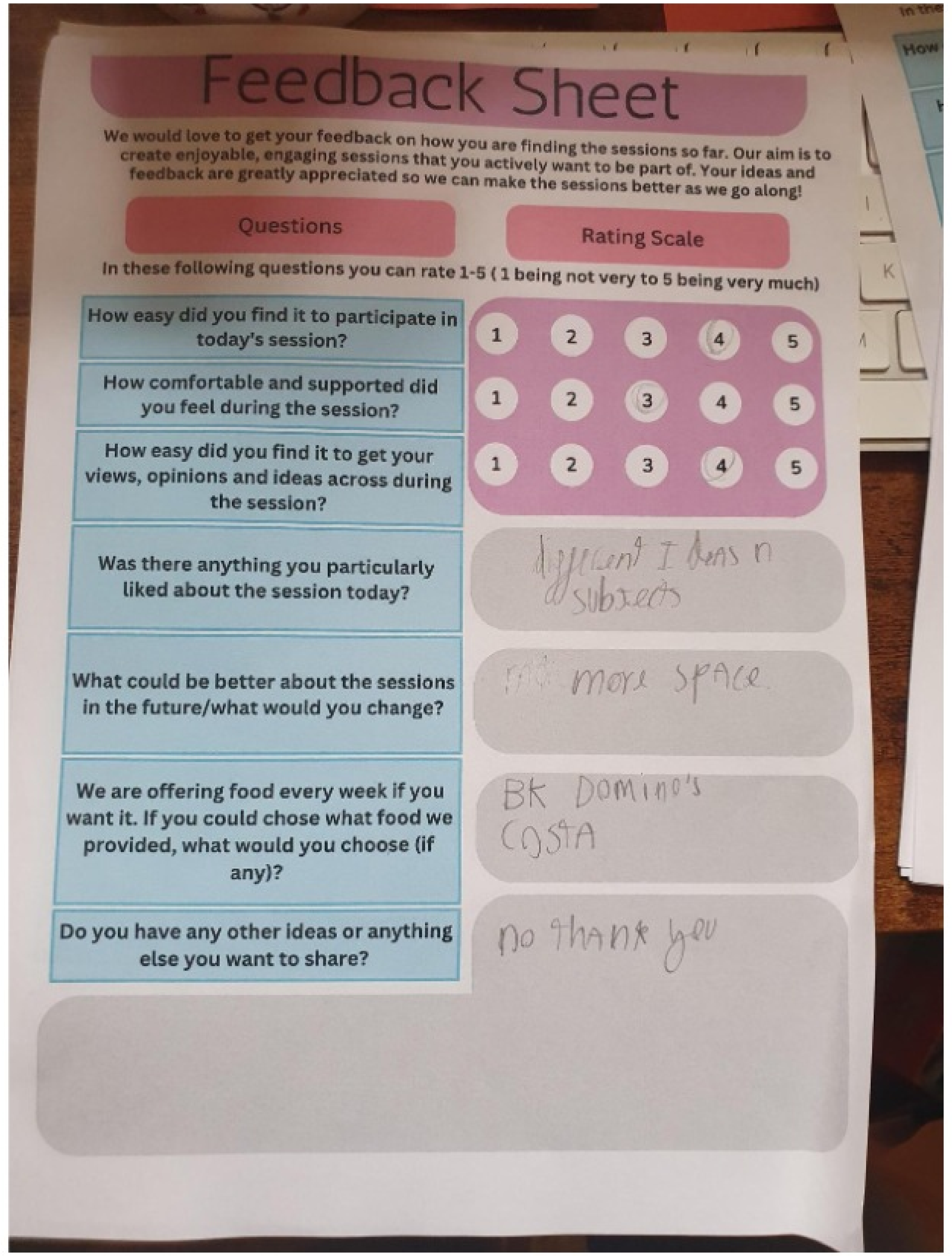

Feedback Sheet

We would love to get your feedback on how you are finding the sessions so far. Our aim is to create enjoyable, engaging sessions that you actively want to be part of. Your ideas and feedback are greatly appreciated so we can make the sessions better as we go along!

Questions	Rating Scale
In these following questions you can rate 1-5 (1 being not very to 5 being very much)	
How easy did you find it to participate in today's session?	1 2 3 **4** 5
How comfortable and supported did you feel during the session?	1 2 **3** 4 5
How easy did you find it to get your views, opinions and ideas across during the session?	1 2 3 **4** 5
Was there anything you particularly liked about the session today?	different Ideas n subjects
What could be better about the sessions in the future/what would you change?	more space
We are offering food every week if you want it. If you could chose what food we provided, what would you choose (if any)?	BK Domino's Costa
Do you have any other ideas or anything else you want to share?	no thank you

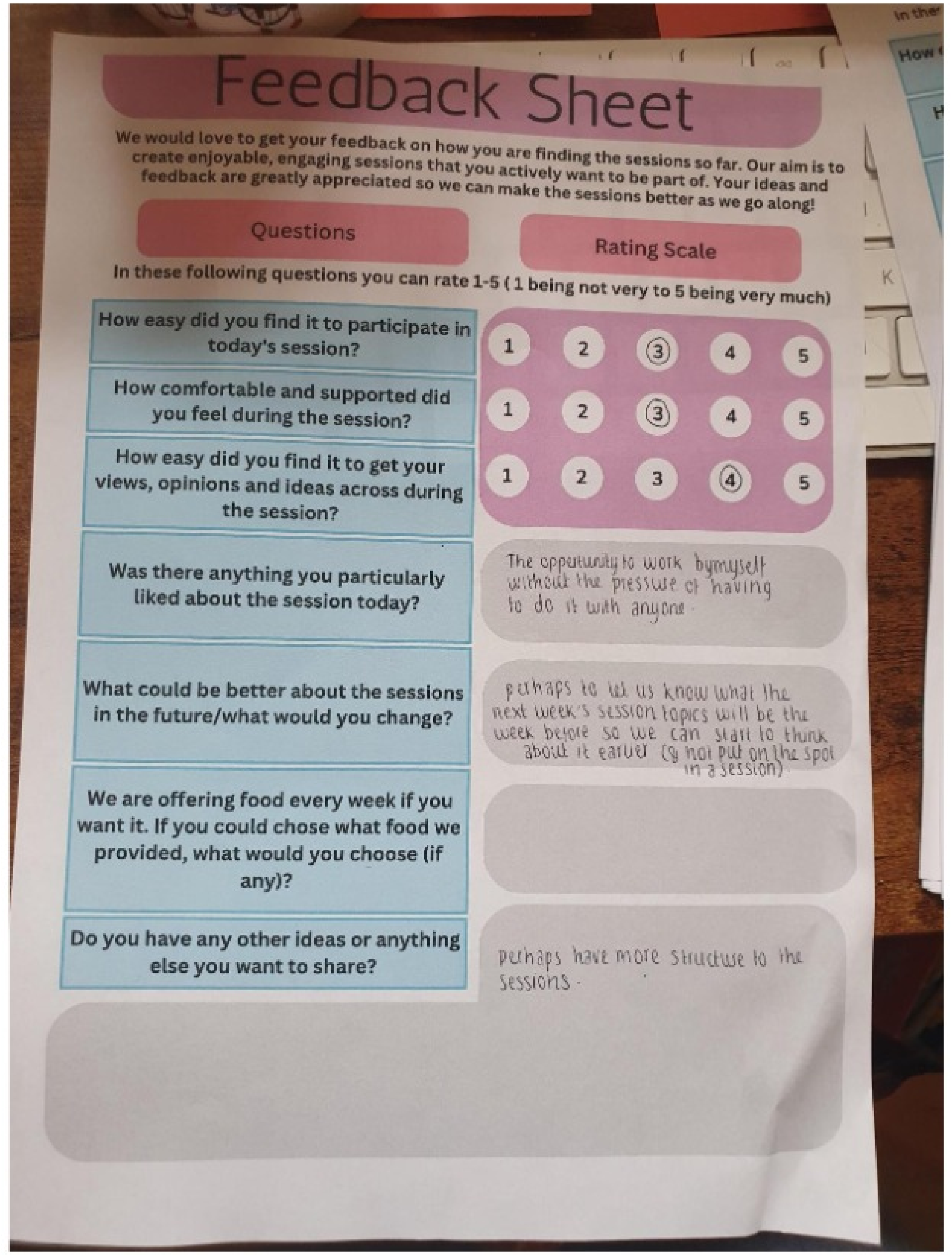

Feedback Sheet

We would love to get your feedback on how you are finding the sessions so far. Our aim is to create enjoyable, engaging sessions that you actively want to be part of. Your ideas and feedback are greatly appreciated so we can make the sessions better as we go along!

Questions	Rating Scale
In these following questions you can rate 1-5 (1 being not very to 5 being very much)	
How easy did you find it to participate in today's session?	1 2 **3** 4 5
How comfortable and supported did you feel during the session?	1 2 **3** 4 5
How easy did you find it to get your views, opinions and ideas across during the session?	1 2 3 **4** 5
Was there anything you particularly liked about the session today?	The opportunity to work by myself without the pressure of having to do it with anyone.
What could be better about the sessions in the future/what would you change?	perhaps to let us know what the next week's session topics will be the week before so we can start to think about it earlier (& not put on the spot in a session)
We are offering food every week if you want it. If you could chose what food we provided, what would you choose (if any)?	
Do you have any other ideas or anything else you want to share?	perhaps have more structure to the sessions.

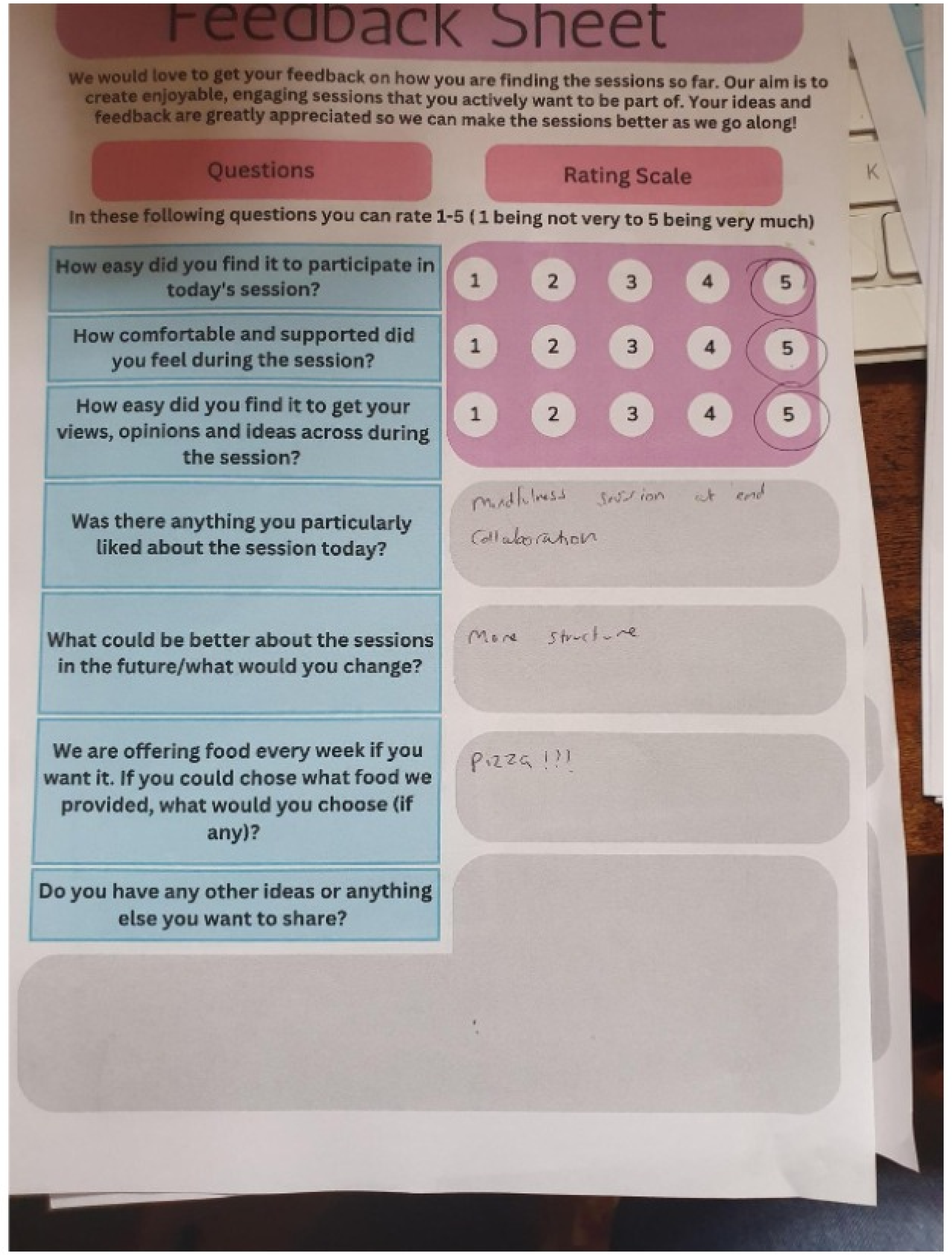

Feedback Sheet

We would love to get your feedback on how you are finding the sessions so far. Our aim is to create enjoyable, engaging sessions that you actively want to be part of. Your ideas and feedback are greatly appreciated so we can make the sessions better as we go along!

Questions	Rating Scale
In these following questions you can rate 1-5 (1 being not very to 5 being very much)	
How easy did you find it to participate in today's session?	1 2 3 4 **5**
How comfortable and supported did you feel during the session?	1 2 3 4 **5**
How easy did you find it to get your views, opinions and ideas across during the session?	1 2 3 4 **5**
Was there anything you particularly liked about the session today?	mindfulness session at end collaboration
What could be better about the sessions in the future/what would you change?	More structure
We are offering food every week if you want it. If you could chose what food we provided, what would you choose (if any)?	Pizza !!!
Do you have any other ideas or anything else you want to share?	

Prompt Table

Prompt	Theme	Post-its
What does this OA mean to you?	Sharing experiences	To be able to talk about mental health<br>To help affect positive change
	Positive change	Able to share experiences ... so others may not have to experience what I did<br>To share my experiences<br>The opportunity to help people using my own experiences<br>[illegible]<br>An opportunity to be heard when it comes to what support I need
	Improve understanding	What does mental health mean for young people? How and where do young people find or feel supported?<br>To know what mental health is
	Root causes	A chance to explain my own issues with mental health and how there are many catalysts to create poor mental health like segregation, exclusion & racism.<br>What causes negative mental health and whats to deal with these causes.
	Wider system	Education in schools & larger facilities about negative mental health and its causes so people are aware and can help...<br>[illegible]
	Awareness	Mental health awareness<br>Advertising, e.g. posters and videos to increase awareness.
What are the key reasons you think it is important for us to work on this topic?	Prevention	To prevent other mental health issues appearing later down the line<br>To help decrease / prevent more people from going years without understanding themselves, and to help increase ??? systems ???
	Help YP feel heard	To help young people talk about their problems<br>Feeling heard<br>To understand from young people how mental health impacts and where they can feel supported, seen and heard
	Lack of support	The support that does exist doesn't work.<br>There is not enough support or general awareness regarding mental health in younger generations.<br>Lack of support and more ??????<br>Example: Lack of support in school from teachers, classmates can lead to young people becoming isolated
	Lack of awareness	Mental health isn't talked about nor are its causes.<br>There is not enough support or general awareness regarding mental health in younger generations.<br>Because it is a topic that has been overlooked by society for so long, until recent times<br>To help the awareness of mental health for younger generations<br>So other people / organisations can understand
	Racism (as a root cause)	In my case I feel like racism can be a cause for my mental health being bad and I feel often that is not accepted.<br>Living in North Devon which is a predominantly white area causes more racism.
	Significance of wellbeing	Because happiness & wellbeing are so important<br>[illegible]<br>[illegible]
Key stakeholders	Specific YP	People with mental / physical disabilities.<br>Children in poverty / difficult home situations<br>Young people<br>People stressed about school<br>Younger generations
	Local authority	Devon County Council<br>Councils<br>Leaders
	Friends & family	Families<br>Parents / carers<br>Family, friends, ???<br>Family<br>Parents<br>Family, friends<br>Family members, friends, partners
	Healthcare	GPs<br>Healthcare staff, mental health workers<br>Support workers<br>GPs / medical centres<br>Doctors
	Education	Schools<br>Teachers, school boards<br>Educators<br>MHSTs<br>Teachers, college lecturers & staff, uni staff
	CAMHS	CAMHS
	Organisations	Place2Be, Young Minds, Mind, Kooth<br>Family Compass<br>Charities and other organisations that work with people of all demographics.<br>Youth services<br>Charities & CICs like Spark and Family Compass
	Employers	Workplaces
Thoughts and feelings	Lived/shared experience	tainted by my experience in education, mental health services (both camhs and adult), local doctors surgeries and society in general. it reminds me of my negative experiences, and makes me feel disheartened, but hopeful that this work/group will make a positive change<br>A chance to listen and compare with other individuals that may have had similar mental issues as me, and may or may have been in the same situations<br>Nervous/anxious but also relieved to finally be able to express my issues in a safe environment<br>To help people who were as vulnerable and mentally unwell as me
	Opportunities to help	How will this change things?<br>excited - good opportunity to help with MH<br>Happy to help precent more trauma from being developed
	ways of learning	To learn better ways to communicate our feelings around MH<br>to learn how to communicate with other YP and the wider community<br>How do we raise awareness in a shared language about mental health<br>how will we enforce a way/system that people are able to educate on mental health and how to improve it
	General feelings	Excited, happy, curiosiity<br>excitement<br>curiosity<br>passionate
Hopes	Spaces	More safe spaces for young people to go to<br>A community hub for young people to go and feel free to be themselves.<br>A place everyone feels comfortable to talk about mental health (for example, boys as it is not as accessible for them).
	General hopes for creating change	I hope everyone wants to live<br>I hope we can improve mental health<br>[illegible]<br>Find ways to support local young people and organisations and remove pressure from CAMHS.<br>We can help improve how we manage our mental health & wellbeing for future generations.<br>[illegible]
	Men's MH	[illegible]
	Remove stigma encourage help seeking	Remove the stigma around young people with mental health issues<br>To create a positive outlook on mental health, help stopping the negative stigma over psych wards<br>To make it more encouraged to ask for help instead of pushing that help away.
	Experiences to be heard and acted on	Help others to avoid what I experienced<br>For my experiences and struggles to be respected and the support I need to be heard
	Better understanding	Understand more about what might work better
	Trusted people, ability to speak to someone	Trusted person to talk to about how feeling.<br>Help people speak out about their problems.
	Parents	[illegible]
	Better support	[illegible]
Fears	No-one to talk to, no support	Other young people felt like they had no one to talk to about their mental health.<br>People of all ages feel like there is nobody they can talk to about mental health.<br>Young people feel they are on a negative spiral without help or anywhere safe to go.
	General fears of no change	Things remained the same as present times.<br>I'm scared it won't help<br>Mental health support for young people doesn't change.<br>I'm worried that we won't be impactful enough
	Worries about future generations	[illegible]
	No policy change	Local and social priorities don't reflect MH
	Experiences aren't heard	My experiences were not respected.<br>[illegible]<br>People still don't feel heard
	Funding and sustainability	Avoid creating something that needs funding.<br>The companies can't find funding then they can't help young people<br>[illegible]<br>Creating something that is not sustainable or does not have longevity
	Not focussing on root causes/soilutions	Distraction rather than thinking about the root cause and actual solution
	More stigma/stereotypes	[illegible]<br>Stereotypes / stereotypical reasons why you might have ??? (ageist??)<br>Creating more stigma for reaching out for mental health<br>Closed-mindedness in communities
	Lack of action from community	Closed-mindedness in communities<br>Communities not taking action

CLUSTERING ANALYSIS - Session 2

What are the key reasons you think it is important for us to work on this topic?

Some of the key reasons that are repeated and stand out - ' Lack of support' this is lacking in a number of sectors that are mentioned, such as schools, mental health help, general awareness, general organisations such as workplaces. Another reason that is prominent in working in this opportunity area is that ' understanding mental health, as mental health has been overlooked by society for so long until recently' . 'We need to help people, everyone should have a place, not be judged, no one should be damaged from school or work. '

Who are the key individuals/ organisations/ people who are important to consider in this?

The main key people that are important to consider in this are; Specific young people such as younger generations, schools pupils, disadvantaged young people in poverty or difficult home situations, people with mental or physical disabilities. Peer support groups, such as youth-led organizations or online communities, can provide a safe space for young people to connect with others who may be experiencing similar mental health challenges, but this also stretches out to youth workers, councillors, medical professionals and teachers.

What are some of the thoughts and feelings that come with this opportunity area for you ?

The thoughts and feelings around this opportunity area are very mixed, after reviewing the information that has been gathered a strong lean towards the lack of education in relation to mental health in schools/education, as well as lack of support availability, some have found resources that have increased mental health knowledge and positive mental health but are rather stretched due to funding or geography, its a common theme that due to the travel in North Devon being very 'hit or miss's that young people haven't had access to support or have been given opportunities to access organisations, therapy/counselling or mental health support groups that feel that they are out of reach or too far to travel as a young person.

Explain in your own words what this opportunity area means to you.

A lot of talk over 'to be able to talk about mental health'.' to be able to share our experiences and learn from them, there are flaws in the systems, from education to mental health care'. Another comment which is repeated is that 'education in schools and larger facilities' such as workplaces to have better mental health awareness. There are some questions that leave us to think what can Kalio do in the future. ' What does mental health means? How and where do young people find and feel supported?
