# Supplementary material for: An adaptive, youth-centred co-design methodology: place-based co-design centring youth and community participation
Source: Res Involv Engagem. 2026 Jan 24;12:33. doi: 10.1186/s40900-025-00833-w (PMC12994241; doi:10.1186/s40900-025-00833-w)
Supplement: Supplementary file 8 — Supplementary Material 8 [file 40900_2025_833_MOESM8_ESM.pdf]

Kailo Feedback Form

We would love to get your feedback on how you are finding the sessions so far. Our aim is to create enjoyable, engaging sessions that you actively want to be part of. Your ideas and feedback are greatly appreciated so we can make the sessions better as we go along!

When you submit this form, it will not automatically collect your details like name and email address unless you provide it yourself.

* Required

1. Which small circle group are you a part of?

◯ Barnstaple

◯ Bideford

2. The following statements relate to your feelings on the sessions, please rate them on a scale of 1-5 (where 1 is not at all, and 5 is completely) *

	1 not at all	2	3	4	5 completely	I'd rather not say
How easy did you find it to participate in the last session?	◯	◯	◯	◯	◯	◯
How comfortable and supported did you feel during the session?	◯	◯	◯	◯	◯	◯
How easy did you find it to get your views, opinions and ideas across during the session?	◯	◯	◯	◯	◯	◯

3. Has there been anything you have particularly enjoyed about the last few sessions? *

Enter your answer

4. What could be better about the sessions in the future? *

Enter your answer

5. What could we do differently to best support you in the sessions? *

Enter your answer

6. Which picture best describes your perception of how well connected, in general, the people in the last session appeared to be (tick the box next to the picture)?

For example, the first picture (option 1) would suggest that there was very little connection between individuals – that they did not come together as a group at all. Whereas the final picture (option 7) would suggest that individuals were very closely connected – they very much came together as a group. *

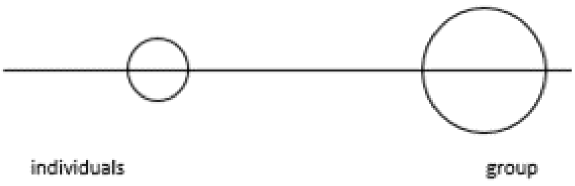

◯ Option 1

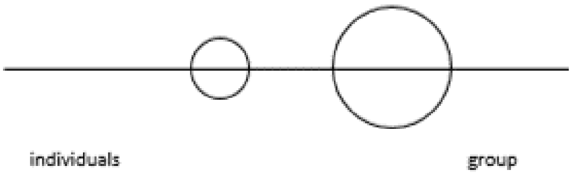

◯ Option 2

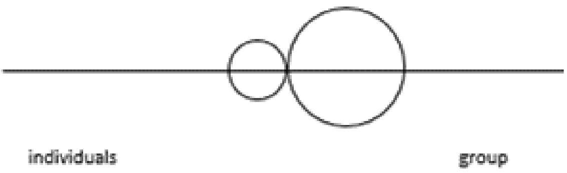

◯ Option 3

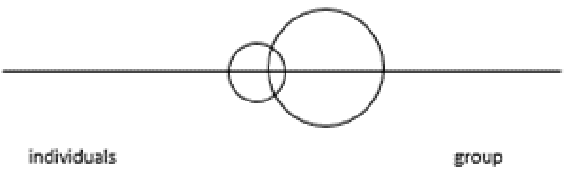

◯ Option 4

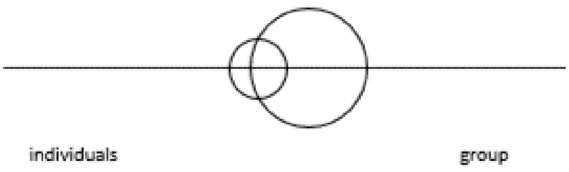

Option 5

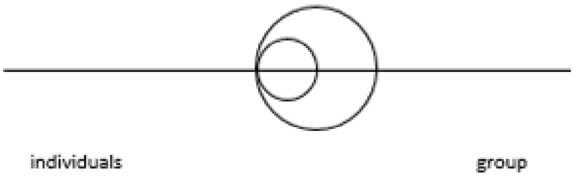

Option 6

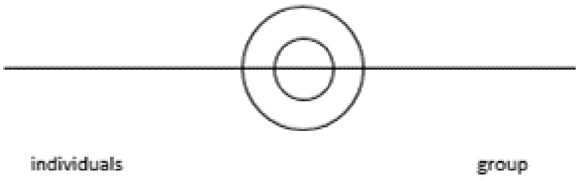

Option 7

7. We are offering food every week if you want it. If you could choose what food we provided, what would you choose (if any)?

Enter your answer

8. Do you have any other ideas or anything else you want to share?

Enter your answer

Never give out your password. Report abuse

Microsoft 365

This content is created by the owner of the form. The data you submit will be sent to the form owner. Microsoft is not responsible for the privacy or security practices of its customers, including those of this form owner. Never give out your password.

Microsoft Forms | AI-Powered surveys, quizzes and polls Create my own form

The owner of this form has not provided a privacy statement as to how they will use your response data. Do not provide personal or sensitive information. | Terms of use
