# Supplementary material for: An adaptive, youth-centred co-design methodology: place-based co-design centring youth and community participation
Source: Res Involv Engagem. 2026 Jan 24;12:33. doi: 10.1186/s40900-025-00833-w (PMC12994241; doi:10.1186/s40900-025-00833-w)
Supplement: Supplementary file 9 — Supplementary Material 9 [file 40900_2025_833_MOESM9_ESM.docx]

## **Summary of meeting:**

We discussed the three finalised key outputs from the co-design groups. The first, Design for Diverse Opportunities, is ready for implementation with potential support from Space and other wider support in ND. Outputs two and three involve designing neurodiversity training for professionals and building mental health awareness for young people. The Local VCSE Lead will promote these initiatives at the next North Devon Partnerships meeting, and funding opportunities are being explored.

Key learnings/reflections focussed on the importance of community partners, time, trust, and adaptability to influence systemic change and support cohesion in the local community. The Devon Mental Health Alliance will host Kailo with pooled funding, emphasising transparency and accountability to young people’s voices. Effective collaboration with community partners has been crucial, and future efforts should focus on active listening, balanced representation, transparency, and regular stakeholder consultations.

**Agenda Main items**

1. Welcome & Introductions
2. Review of Co-design Outputs
3. Implementation Update – Output 1
4. Development Update – Outputs 2 & 3
5. Key Learnings & Reflections
6. Kailo Framework & Future Planning
7. Community Partnership Reflections
8. Next Steps & Next Meeting

## **Implementation Update**

### **Output 1: Design for Diverse Opportunities**

**Current status:**

- Blueprint for the intervention/activity finalised.
- Ready for implementation in partnership. Spoken to Space for potential support/contacts (piloting specific elements).
- Exploring partnerships with DCC library hubs and career-focused organisations in North Devon.

**Discussion points:**

- **Local VCSE Lead:**
  - Contact Wings in Bideford, a charity working with young people who have fallen out of the educational system.
  - Reconnect with contacts at Petroc (noting recent senior leadership changes).
  - Push for systems thinking to be promoted at board level.
  - Key contacts include leaders at TTVS and local Trans resilience support.
  - Keen to showcase the importance and impact of these designs by piloting and testing in locations such as Torridge.
  - Provided an update on Levelling Up in Torridge: board representation confirmed; elections have halted progress.

**Next steps:**

- Partner with organisations to pilot and implement the design elements.
- Reach out to DCC library hubs and career-focused organisations in North Devon.
- Reconnect with key contacts for coordination.
- Explore partnerships for testing and resource development.

## **Output 2 & 3: Neurodiversity Training and Mental Health Awareness**

**Current status:**

- Development underway for neurodiversity training for health professionals and broader mental health awareness activities for young people in Northern Devon.
- Ongoing discussions with the Family Hubs team; some underspend from co-design work can support these outputs.

**Discussion points:**

- **Lead Facilitator & Local Youth/Community Organisation Lead:**
  - Working on developing training with input from co-design groups.
  - Initial conversations with the Family Hubs team showed interest and available resources.
  - Planning to progress co-design outputs into training and awareness activities.
- **Local VCSE Lead:**
  - Suggests promoting these initiatives at the next North Devon Partnerships meeting.
  - Exploring funding options, including Awards for All and Lottery.
- **Co-investigator for the Kailo Project:**
  - Highlighted the value of using lottery resources for community initiatives.
  - Noted challenges linked to multiple hubs (mental health, family, youth) without sufficient integration.
- **Young Person Leader at Local Youth Organisation:**
  - Raised concerns about a lack of responses from local MPs on youth mental health inquiries.
  - Noted political interest in expanding walk-in hubs.
- **Lead Facilitator:**
  - Suggested developing learning resources or cards for professionals.
  - Identified a need for neurodiversity tools for health professionals, especially GPs.
- **Local Youth/Community Organisation Lead:**
  - Emphasised inviting professionals to send one or two team members for training to support internal communication and growth.
- **Local VCSE Lead:**
  - Mentioned existing training (e.g., myth-busting complex needs) that could be integrated, potentially led by NDVS.
- **Local Youth/Community Organisation Lead (additional role):**
  - Highlighted the need for integrated support and practical therapeutic spaces.

**Next steps:**

- Finalise training plans and pilot approaches.
- Collaborate with the Family Hubs team and other potential recipients.
- Lead Facilitator and Co-investigator to explore funding and collaboration opportunities.
- Develop and distribute learning resources for professionals.

## **Key Learnings**

### **Community Partners**

- Critical for embedding connections with young people and community members.
- Place-based systemic change requires time, trust and strong relationships.
- Emphasis on addressing social determinants and shifting power to young people.

### **Challenges**

- Staying focused on systemic constraints.
- Ensuring young people’s voices meaningfully shape decision-making.

### **Reflections**

- The group is uniquely positioned to lead this innovative work.
- Ongoing alliances and collaboration are essential.

## **Next Steps**

### **Codifying Kailo Framework**

- Convert into an open, web-based model for implementation over the summer.
- Devon Mental Health Alliance to host Kailo going forward.
- Goals include continuing current co-designs, initiating new discovery cycles, and developing a pooled budget for community partners and Kailo work.

### **Accountability**

- Developmental evaluation to ensure adherence to Kailo principles.

## **Reflections on Community Partners’ Role**

### **What Worked Well**

- Diverse perspectives and broad community reach.
- Shared expertise and creativity.
- Youth-centred focus and sense of shared purpose.
- Strong collaboration and open communication.

### **Suggestions for Future Work**

- Active listening and maintaining youth voice.
- Balanced representation and transparency.
- Amplifying smaller voices.
- Regular stakeholder consultations.

## **Next Partnership Meeting**

- Scheduled for September.
- Continue transitioning Kailo locally and address emerging priorities.

If you’d like, I can also produce:

- A more concise anonymised version
- A version formatted for circulation (e.g., bullet summary or RAG update)
- A version removing all place references, not just personal names
