# Supplementary material for: An adaptive, youth-centred co-design methodology: place-based co-design centring youth and community participation
Source: Res Involv Engagem. 2026 Jan 24;12:33. doi: 10.1186/s40900-025-00833-w (PMC12994241; doi:10.1186/s40900-025-00833-w)
Supplement: Supplementary file 10 — Supplementary Material 10 [file 40900_2025_833_MOESM10_ESM.docx]

# Supplementary Material: Risk assessment for safeguarding young people involved in Small Circle co-design sessions

| **What is the risk?** | **Who might be harmed?** | **How might people be harmed?** | **Existing risk control measures** | **Resources required/actions pre-event** | **Risk Level** | **Impact** |
| --- | --- | --- | --- | --- | --- | --- |
| **Ruptures in relationships i.e. between young person and Kailo team member, between young person and local community organisation expected to host them** | Young person | Young person’s emotional wellbeing might be harmed  Young person’s confidence and trust in the Kailo work | Young people should be assigned a safeguarding person who is not involved in day-to-day Kailo activities. This person should be someone they can speak to in case any issues arise. Ideally, they will be introduced to this person during induction. Kailo team members will support host organisations in building and maintaining good relationships with young people. In most cases, the organisation might be better placed to hold and manage relationships than Kailo members. Thus, Site teams should also learn from these organisations and create spaces for this to be led by them. | - Induction and regular line management - Having more than one person that young people can speak to - Safeguarding person appointed - Agreement of roles and responsibilities discussed and completed by Kailo team and organisational hosts (if applicable) - Discussion and understanding of the ethical stance and values of Kailo | Low | High |
| **Conversations and activities focused on mental health and wellbeing cause distress and harm** | Young People and other Kailo team members | Young people may be triggered by the discussions during the event causing emotional distress:  - crying,  - anger,  - being upset,  - re-traumatisation.  There may be further impact on emotional well-being when researching sensitive or traumatic topics | Kailo staff hold recent safeguarding training. YP are clear about what the focus of the event is. Ideally a conversation with all YP attending will take place between them and North Devon Kailo team. It is clear to YP that there is no expectation for them to participate if they don’t want to, and they can stop participating at any point during the event. Space is provided where YP can go if they need a breather. We have someone in the room (a ‘safeguarding lead’) who is prepared and equipped to monitor and support young people throughout the event and to notice any signs of a young person being triggered. Should a YP make a disclosure site leads are to follow DSDL’s safeguarding procedure. If unable to get hold of a DSO, Kailo team call NSPCC hotline 0808 800 5000 or report directly to the North Devon LADO: <https://services.devon.gov.uk/web/lado/form> We are equipped to signpost and support young people before during and after the event. | - Ensure the researcher is adequately trained in dealing with sensitive topics and has the option to opt-out of research that may be emotionally triggering. - Establish a debriefing process after each research session to allow the researcher to process any emotional reactions and seek support if needed. - Ensure that all appropriate documentation of any safeguarding concerns is completed in full and dealt with appropriately     **Potential resources: -** MH and wellbeing guides printed out for YP  - Young minds resources  - Signposting resources  - Safeguarding lead prepped and equipped  - Consent form  - Parents agreement  - Safe space set up for YP to go for some time out. | Medium | Medium |
| **Young people don’t feel comfortable or able to participate in the space** | Young Person | Emotional distress by young person    Kailo initiative receives reputable harm | The facilitation of the event will provide various ways to engage with the conversations- whether this be through individuals/pairs/small groups and the larger groups. There will be different activities from drawing, ones that are more physical as well as some conversations. We will provide prompts and supports to encourage relationship building within the group (e.g. conversations starters and activities) and there will be dedicated time at the start for this. We will send out communications to professionals attending to remind them that YP will also be there and set some ground rules. In all smaller groups there will be a member of the Kailo team to ensure that young people are getting what they need in the group. Optimal layout will be considered to make sure the physical environment is best equipped to reduce potential anxiety (adequate distance etc.). We will minimise the presence of sharp or heavy objects. | - Communicate to professionals and to YP about how we will support them. - Ground rules/agreements from participants to be confirmed - Discussion and understanding of the ethical stance and values of Kailo It is important to ensure back up procedures are in place, permit YP or participants to terminate the session at any time without needing to reason why, plan an early exit strategy in case of safety concerns. | Low | Medium |
| **Disagreements/arguments/conflict within the group (Within or between professionals, YP or parents)** | Young People, Professional Attendees, Kailo/Dartington Staff members | Emotional harm, physical harm | Ground rules set at the start of the session about how we will work together: We don’t have to agree on everything, but we want to respect, listen and understand from everyone’s perspective and then make a decision about priorities. We know that lots of people are very passionate about different aspects of this, so want to make sure we hear from everyone.    Clear that even if there is disagreement within the group, and someone has a different opinion to the majority, there will also be time and space to give individual view at the end Kailo team members will be facilitating /present in all the groups and can guide the conversation in a productive direction.    If things do get too heated, we will have space for people to take a breather with a member of the Kailo team whose responsibility is YP/Safeguarding. | - Helpful to emphasise the openness of space and reinforce a respect for others’ boundaries. - A need for professional's present to be able to read the room and mediate the situation - Discussion and understanding of the ethical stance and values of Kailo |  |  |
| **YP travelling to/from the event get hurt/injured. Potential risk to physical safety throughout research** | Young people | Accidents, injuries, or violence from individuals encountered during research. Offsite injuries can carry several risks, especially if in an isolated or dangerous setting | Aim to carry out work during daylight hours if possible. Plan routes and transport well in advance. Wear appropriate clothing and footwear when travelling to a local organisation. Avoid carrying easily accessible confidential material. Leave and travel on an expected and pre-planned schedule | - Conduct a thorough risk assessment of the research location and ensure appropriate safety measures are in place, such as emergency contact information, first aid kits, and protocols for dealing with emergencies. - Establish a regular check-in system with a designated contact person to ensure the researcher's safety is monitored. | High | High |
| **Medical emergency- asthma, panic attacks, choking, chronic illness etc.** | Any attendee or member of Kailo staff- not specific to any stage in the induction process but useful to highlight in initial stages | Allergic reaction, physical injury (cut, bleeding, broken bone) | Consent forms with emergency contact details of YP Obtained any medical info we need to know from the YP during the event registration process.    Should a medical emergency arise, Kailo staff to follow emergency procedure and contact 999. Share address with operator. | - Consent and information forms sent to YP and completed. - Ascertained the nearest hospital | High | High |
| **Confidential and private data being released** | Young people/ others involved in the research process | Unintentional disclosure of sensitive information that could jeopardise the safety of participants or the researcher | Young researchers will be provided with password protected computers with active VPNs. They will be instructed on what they can access and where and where to safely store any recorded information. Information should always be stored correctly (through any form) and protected correctly. | - Ensure the young social researcher is trained in ethical research practices and understands the importance of maintaining confidentiality. - Establish clear protocols and boundaries for data storage, such as password-protected files, and ensure the researcher has access to secure technology. - Obtain informed consent from participants that outlines the risks associated with the research and how their privacy and confidentiality will be protected. - The young researchers and designers should complete appropriate GDPR training courses.      - From ethics: Everything that we talk about today is private or confidential unless I’m worried that any harm or danger is going to come to you or to anyone else, in which case then I am obliged to speak to [safeguarding contact] about what has worried me, but I would try and tell you if I was worried in this way first. | High | High |
| **DBS check reveals conviction, reprimand, caution or *warning information* (Based on ethics comments discussion)*References:*** [**YFF Future voice JD**](https://youthfuturesfoundation.org/wp-content/uploads/2022/06/YFF-Future-Voices-Group-Recruitment-Guidance-Pack.pdf)**, 2022** | Young person (18+) with ‘negative’ DBS Other young people involved in the project | A young person might already be involved in Kailo activities and could feel disappointed if the information were to end/change their relationship with the project. | People are eligible to apply for roles within Kailo if they have a criminal record- a DBS check will be required for roles where they are working directly with young people i.e. the young researcher and designer roles. For any young people in roles which require a DBS check and something is flagged in the check, things will be discussed in further detail to understand the nature of the record and assess whether the young person can stay i. If a DBS check informs us that someone is on the Children’s Barred list, they will be denied the role and authorities may be informed.    Although DBS checks won’t be required for young people involved in the small circle, site leads will carry out individual preparatory conversations with all young people prior to their involvement in the small circle of co-design. These conversations will help Site leads and young people to better understand their desire to join the group and consider their readiness to engage with such a group. Kailo site leads will also discuss ground rules and policies for what standards and behaviour are expected in these groups, and make it clear what will happen if these rules are broken. Kailo site leads will also have a conversation about the use of DBS checks to understand young person’s situation. | - For those who require DBS checks for their roles i.e. young researchers and designers, site leads will discuss the DBS process and the possible outcomes, concerns and procedures which may arise - Kailo team to familiarise themselves and those applying with what convictions may hinder their opportunity to apply, highlighted below. - We have stated that these are not legally required for young people, that this will be encouraged (not mandatory). Individual conversations will be carried out before small circle activities begin and Site leads should seek to better understand young people's situation. They should also explain how we will be using DBS information, and it's worth noting that not all DBS flags will prevent you from working in these settings. - We won’t be asking anyone who doesn’t have a DBS check to be doing anything unsupervised with other young people | Low | High |
| **Young person may feel overwhelmed with the onboarding process/ work related stress**    ***References:*** [Health and Safety executive- What are the management standards?](https://www.hse.gov.uk/stress/standards/index.htm)**, 2023** | The young person/ other Kailo team members involved | Increased levels of stress and feelings of overwhelm may lead to a drop in motivation to work, increase in negative mood and anxiety, and may cause the individual to withdraw from commitments. | It is important to promote active discussion and working with YP and the site leads to help decide on practical improvements. It will be a priority for site co-pilots and leads to manage the following to alleviate potential risks associated with stress:   - **Demands:** Issues around workload, work patterns, and work environments. - **Control:** letting the YP have a say in the way they do their work. - **Support:** perhaps in the form of encouragement/routine check-ins from line managers and colleagues. - **Role:** making sure the YP fully understands and has a grasp of their role in this work. | - Ensure there is a clear exit route if anyone were to feel they needed to separate themselves from the room | Low | Medium |
| **Use of IT for internet searches**    ***References:*** [St George’s University of London: Young Person’s risk assessment and induction checklist](https://www.google.com/url?sa=t&rct=j&q=&esrc=s&source=web&cd=&ved=2ahUKEwj4xbiW_t39AhUD_rsIHfDEDbsQFnoECA8QAQ&url=https%3A%2F%2Fwww.sgul.ac.uk%2Fabout%2Four-professional-services%2Fsafety-health-and-environment%2Fdocuments%2FYoung-person-risk-assessment-induction-checklist-June-2015.docx&usg=AOvVaw0xXgKFYM2NzStNh28Gqn60)**, 2016** | Young person | Exposure to inappropriate, harmful, or triggering material | Equipment provided to have automatic blocks and filters on to navigate around any inappropriate content. | Supervision needed, onboarding in safeguarding training, Discussion of the IT policy at UCL and Dartington. | Low | Medium |
| **Use of IT/general Office equipment**    ***References:*** [St George’s University of London: Young Person’s risk assessment and induction checklist](https://www.google.com/url?sa=t&rct=j&q=&esrc=s&source=web&cd=&ved=2ahUKEwj4xbiW_t39AhUD_rsIHfDEDbsQFnoECA8QAQ&url=https%3A%2F%2Fwww.sgul.ac.uk%2Fabout%2Four-professional-services%2Fsafety-health-and-environment%2Fdocuments%2FYoung-person-risk-assessment-induction-checklist-June-2015.docx&usg=AOvVaw0xXgKFYM2NzStNh28Gqn60)**, 2016** | Young person and other Kailo team members | If used for prolonged periods of time, people may become fatigues and stressed | Promote the use of regular breaks and actively engage with routine supervision to understand the young person’s capacity and understanding of work | Set up regular check-ins alongside YP’s calendar and plan training in understanding work management and approaches | Low | Medium |
| **Trip Hazards/trailing cables**    ***References:*** [St George’s University of London: Young Person’s risk assessment and induction checklist](https://www.google.com/url?sa=t&rct=j&q=&esrc=s&source=web&cd=&ved=2ahUKEwj4xbiW_t39AhUD_rsIHfDEDbsQFnoECA8QAQ&url=https%3A%2F%2Fwww.sgul.ac.uk%2Fabout%2Four-professional-services%2Fsafety-health-and-environment%2Fdocuments%2FYoung-person-risk-assessment-induction-checklist-June-2015.docx&usg=AOvVaw0xXgKFYM2NzStNh28Gqn60)**, 2016,** [National Youth Agency, 2022: Example risk assessment](https://s3.eu-west-1.amazonaws.com/assets.nya2.joltrouter.net/wp-content/uploads/2022/11/15161927/Example-Risk-Assessment2.pdf)**, 2022** | Young person and other Kailo team members | Young person may gain injuries from slips, trips or falls at their place of work/meeting | Ensure workplace environment is orderly and maintained | May be helpful for staff at the organisation to prepare the space before the YP arrives to make sure there are no potential hazards- any hazards that are present must be clarified to the YP | Low | Medium |
| **Potential emergencies (including fire)**    ***References:*** [St George’s University of London: Young Person’s risk assessment and induction checklist](https://www.google.com/url?sa=t&rct=j&q=&esrc=s&source=web&cd=&ved=2ahUKEwj4xbiW_t39AhUD_rsIHfDEDbsQFnoECA8QAQ&url=https%3A%2F%2Fwww.sgul.ac.uk%2Fabout%2Four-professional-services%2Fsafety-health-and-environment%2Fdocuments%2FYoung-person-risk-assessment-induction-checklist-June-2015.docx&usg=AOvVaw0xXgKFYM2NzStNh28Gqn60)**, 2016** | Young person and other Kailo team members | Potential for physical injury | Ensure there are routine fire drills where necessary at local place of work and there is supervision during emergency situations | Fire safety/exits are clearly outlined during young person’s first day at organisation | Medium | High |
| **Economic risk involved with travel to and from places**    ***References:*** [University of Oregon: Examples of potential risks to subjects](https://research.uoregon.edu/manage/research-integrity-compliance/human-subjects-research/examples-potential-risks-subjects)**, 2023** | Young person | Young person may not be able to financially support the cost of travel/food when working in the local organisation away from home | Kailo will cover all expenses for young people related to events and other Kailo-related appointments, including travel, food, and accommodation costs. However, this does not include costs related to regular working day expenses. | Specify these processes clearly and lay out the budgets available and limits on expenses (covered in the risk assessment of the journey which would be discussed with the young person if required) | Low | Low |
| **Cleanliness**    ***References:*** [Risk assessment and the provision of training](https://app.croneri.co.uk/feature-articles/risk-assessment-and-provision-training)**, 2022** | Young person and other Kailo team members | The training venue may not be appropriate or safe for everyone involved in the training if in person | The training room must be clean and tidy. There must be sufficient space for each person to work safely and comfortably. Floor surfaces must be maintained in a safe condition and suitable for the type of activities being conducted. Steps/stairs/ramps must be in a safe condition with non-slip surfaces, and secure handrails where needed. Trailing cables must be managed by re-routing or protection to avoid tripping hazards. | Ensure training venue is booked and planned ahead of schedule | Low | Low |
| **Training venue is not adhered to support needs and disabilities**    ***References:*** [Risk assessment and the provision of training](https://app.croneri.co.uk/feature-articles/risk-assessment-and-provision-training)**, 2022** | Young person and other Kailo team members | Accessibility may be limited if the venue and training content is not accommodating for all- this may lead to feelings of seclusion and stress | Where necessary, the training facilities must have been adjusted (reasonable adjustments) in accordance with the Equality Act 2010 (<https://www.gov.uk/guidance/equality-act-2010-guidance>). For example, training facilities must be able to accommodate those in wheelchairs. Fire evacuation procedures must take into account those with mobility issues. | Scope of the proposed training venue beforehand to ensure that it fits appropriate criteria | Medium | Medium |
