# Supplementary figures and images for: An adaptive, youth-centred co-design methodology: place-based co-design centring youth and community participation
Source: Res Involv Engagem. 2026 Jan 24;12:33. doi: 10.1186/s40900-025-00833-w (PMC12994241; doi:10.1186/s40900-025-00833-w)

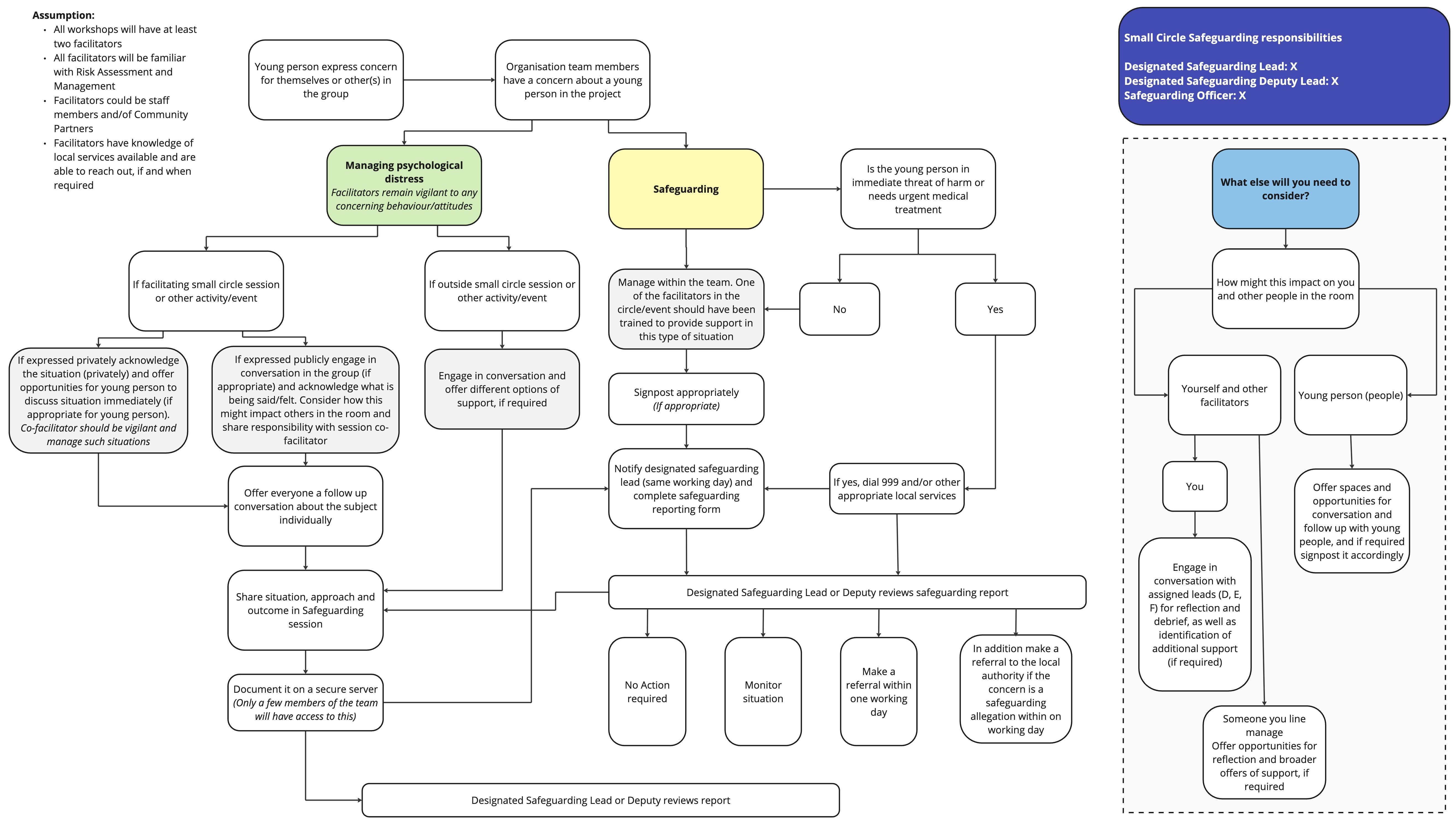

Supplement: Supplementary file 11 — Supplementary Material 11 [file 40900_2025_833_MOESM11_ESM.jpg]
