# Supplementary material for: An adaptive, youth-centred co-design methodology: place-based co-design centring youth and community participation
Source: Res Involv Engagem. 2026 Jan 24;12:33. doi: 10.1186/s40900-025-00833-w (PMC12994241; doi:10.1186/s40900-025-00833-w)
Supplement: Supplementary file 12 — Supplementary Material 12 [file 40900_2025_833_MOESM12_ESM.docx]

**Supplementary Material 12: Researcher Positionality Statement**

The Kailo research, design, and delivery team brought a wide range of lived experiences to the process, including growing up and living in rural, coastal, and urban areas; past and present experiences of poverty, income and job insecurity, unemployment, homelessness and residence in deprived communities; being from ethnic and religious minority backgrounds, lived experience of neurodiversity and mental health challenges; experience of care; and exposure to youth violence.

These perspectives were complemented by extensive professional experience in working with young people and communities facing similar challenges. This included team members who had or had previously had professional roles in youth work, safeguarding, education and employment, public health, and the support of vulnerable and neurodivergent young people. The team also brought professional expertise in qualitative and quantitative research methods, co-design, participatory research, community organising, systems thinking, and systems change.

Collectively, these lived and professional experiences informed our approach to research design, delivery, and interpretation, particularly in recognising and addressing power dynamics between researchers and communities.
