# Supplementary material for: An adaptive, youth-centred co-design methodology: place-based co-design centring youth and community participation
Source: Res Involv Engagem. 2026 Jan 24;12:33. doi: 10.1186/s40900-025-00833-w (PMC12994241; doi:10.1186/s40900-025-00833-w)
Supplement: Supplementary file 13 — Supplementary Material 13 [file 40900_2025_833_MOESM13_ESM.docx]

**Supplementary Material 13:**

**Big Circle Session Outline and activities**

**Big Circle Sessions Overview**

| **Sessions** | **Broad focus** |
| --- | --- |
| Session 1 | Introduction to Small Circle and Big Circle aims |
| Session 2 | Presentation of the system maps produced in the Small Circles and assessment of priorities for next steps |
| Session 3 | Presentation of co-designs developed in the Small Circles and discussion of the Big Circle’s role |

**Example of session outline Big Circle Meeting 2:**

Date and Time

Kailo facilitators: ……

**Purpose:**

- Update attendees on the Kailo process (Northern Devon and wider)
- Input to the design opportunities including prioritisation.
- Buy in to the prioritised areas

| **Time** | **Activity** | **Facilitator** | **Resources** |
| --- | --- | --- | --- |
| **10:00** | Welcome and intro/overview of session today |  | Agenda |
| **10:10** | Overview/Update of Kailo including Future Plans- North Devon and Beyond   - Share academic articles |  | Academic Article Links |
| **10:25** | Update of Co-design Activities and Outputs/Outcomes   - Barnstaple: MH Awareness/education and Relationships - Bideford: Diverse Opportunities |  | Slides/Overview of the Co-design Team journey including prioritised design opportunities for Barnstaple and Bideford |
| **10:55** | Clarifying Questions/Reflections |  |  |
| **11:00** | Break |  |  |
| **11:05** | **Breakout Rooms (x4 or 5?**  Deep dive into prioritised design areas   1. General reflections on this as a prioritised design opportunity, inc. Why do you think this might have been prioritised by our groups? 2. What is already working in North Devon in this area? 3. What do you think is missing/needed? 4. Do you have any examples of best practice in this area that would be helpful for us to consider?   20mins and then the groups swap so they have the opportunity to input into two design opportunities? |  |  |
|  | **Poll:**  Voting on which Design Opportunities they think should be taken forward |  | Poll |
| **11:45** | **Next steps Dec-March**   - Would you be interested in working more closely with our co-design groups on these prioritised areas in the new year? |  | Outline of Next Steps |
| **11:50** | **Any other Questions/Reflections or Input?** |  |  |
| **11:58** | **Close**  One word in the chat/one reflection you have after the meeting today**.** |  |  |
| **12:00** | **End** |  |  |
