# Supplementary material for: An adaptive, youth-centred co-design methodology: place-based co-design centring youth and community participation
Source: Res Involv Engagem. 2026 Jan 24;12:33. doi: 10.1186/s40900-025-00833-w (PMC12994241; doi:10.1186/s40900-025-00833-w)
Supplement: Supplementary file 14 — Supplementary Material 14 [file 40900_2025_833_MOESM14_ESM.docx]

**Supplementary Material 14:** Small Circle Demographics Data Across Two Sites

|  | **Consolidated children and young person demographic data across two sites (%)** | |
| --- | --- | --- |
| **Category** | **Northern Devon (22 young people)** | **Newham (39 young people)** |
| **Ethnicity** | - 59% White- English / Welsh / Scottish / Northern Irish / British - 4.5% Asian / Asian British- Bangladeshi - 4.5% Any other Black / African / Caribbean/ Black British background - 4.5% Latino American - 4.5% Mixed / Multiple ethnic groups-White and Asian - 4.5% Mixed / Multiple ethnic groups- White and Black African - 4.5% Any other Mixed / Multiple ethnic background - 14% Not Reported | - 13% White- English / Welsh / Scottish / Northern Irish / British - 7% Asian / Asian British- Bangladeshi - 3% Mixed / Multiple ethnic groups-White and Asian - 77% Not reported |
| **Nationality** | - 64% British - 9% English - 4.5% Nationality other than British - 4.5% Mixed British and South American - 18% Not reported | - 21% British - 3% Prefer not to say - 76% Not reported |
| **Gender identity** | - 41% Women - 36% Men - 4.5% non-binary - 4.5% Questioning/ unsure - 14% Not reported | - 13% Women - 10% Men - 77% Not reported |
| **Sexual Orientation** | - 64% Straight (heterosexual) - 9% Bisexual - 4% Questioning or unsure - 9% Prefer not to say - 14% Not reported | - 18% Straight (heterosexual) - 5% Bisexual - 77% Not reported |
| **Religion** | - 36% No religion - 32% Catholicism/ Christianity - 9% Not Applicable - 4.5% Islam - 4.5% Jewish - 14% Not reported | - 8% No religion - 2% Catholicism/ Christianity - 8% Islam - 5% Jewish - 77% Not reported |
| **Entitled (or were entitled) to free school meal vouchers** | - 64% No - 22% Yes - 14% Not reported | - 13% No - 10% Yes - 77% Not reported |
| **Parents/carers or guardians' employment situation?** | - 50% Both Parents Employed - 9% Only one parent is employed - 4.5% Both parents unemployed - 4.5% One parent is carer for relatives - 4.5% Prefer not to say - 28% Not reported | - 15% Both Parents Employed - 8% Don’t Know - 77% Not reported |
| **Did parents/carers or guardians attend university** | - 41% Yes - 41% No - 18% Not reported | - 10% Yes - 10% No - 3% Don’t know - 77% Not reported |
| **Care responsibilities** | - 55% No - 31% Yes - 14% Not reported | - 13% Yes - 10% No - 77% Not reported |
| **Physical or mental health conditions or illness** | - 59% No - 23% Yes - 4% Prefer not to say - 14% Not reported | - 18% No - 3% Yes - 2% Prefer not to say - 77% Not reported |

**N.B**. Please note that data was collected from only some of the young people who attended the small circle sessions, with missing information recorded as “Not reported.” Among those who did complete the form, some did not answer every question. As a result, the percentages shown reflect only this subgroup, rather than the full group of participants.
One Newham group chose not to complete the demographic form because they did not feel comfortable doing so.
