# Supplementary material for: An adaptive, youth-centred co-design methodology: place-based co-design centring youth and community participation
Source: Res Involv Engagem. 2026 Jan 24;12:33. doi: 10.1186/s40900-025-00833-w (PMC12994241; doi:10.1186/s40900-025-00833-w)
Supplement: Supplementary file 15 — Supplementary Material 15 [file 40900_2025_833_MOESM15_ESM.pdf]

Big Circle update on systems

Kailo Northern Devon

dartington.org.uk
info@dartington.org.uk
@DartingtonSDL
(01803) 762400

How might we help young people, families and other key people in their community to build stronger* relationships *to support young people's mental health and wellbeing?

Barnstaple Small Circle Opportunity Area

dartington.org.uk
info@dartington.org.uk
@DartingtonSDL
(01803) 762400

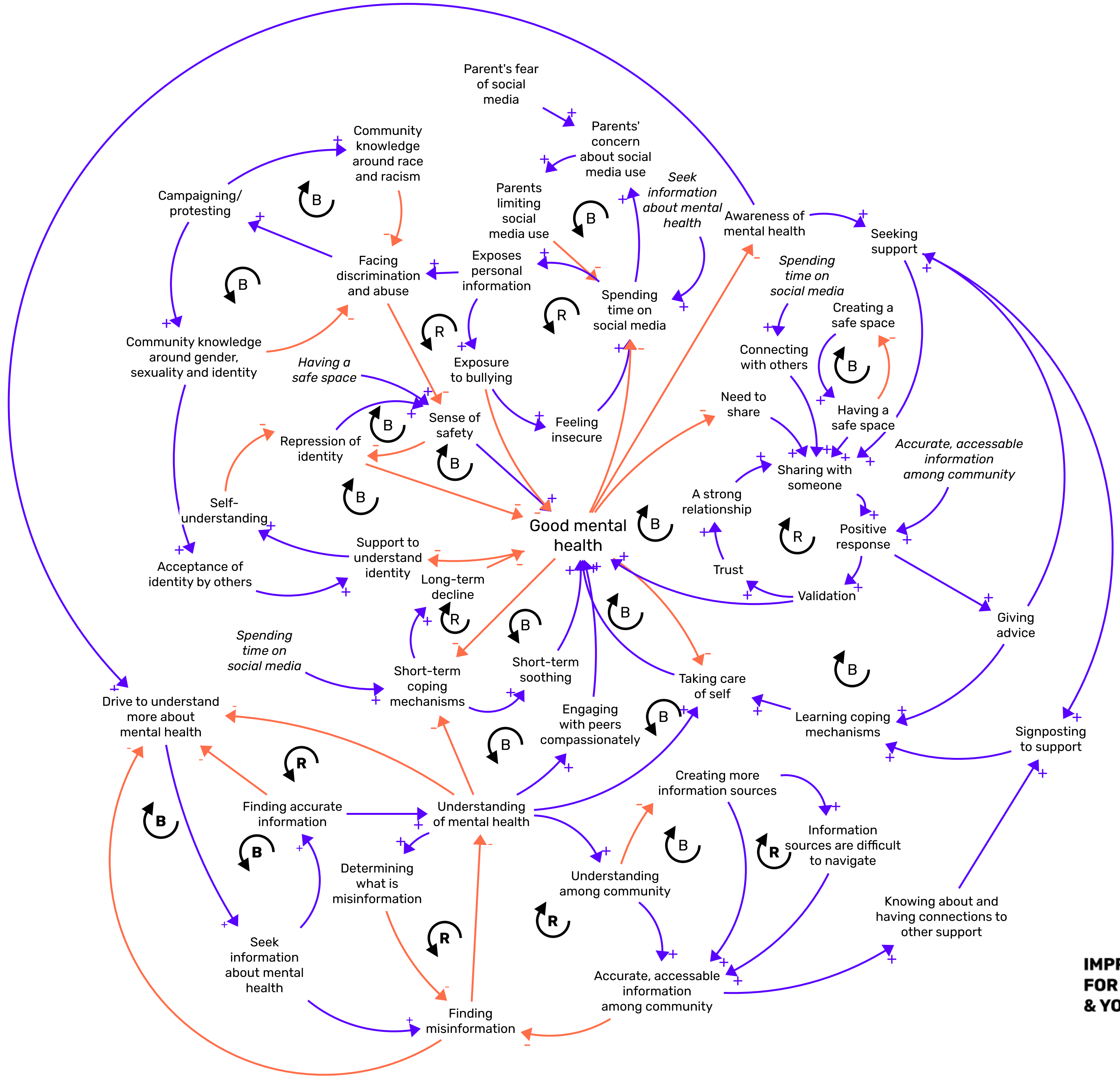

DARTINGTON SERVICE DESIGN LAB

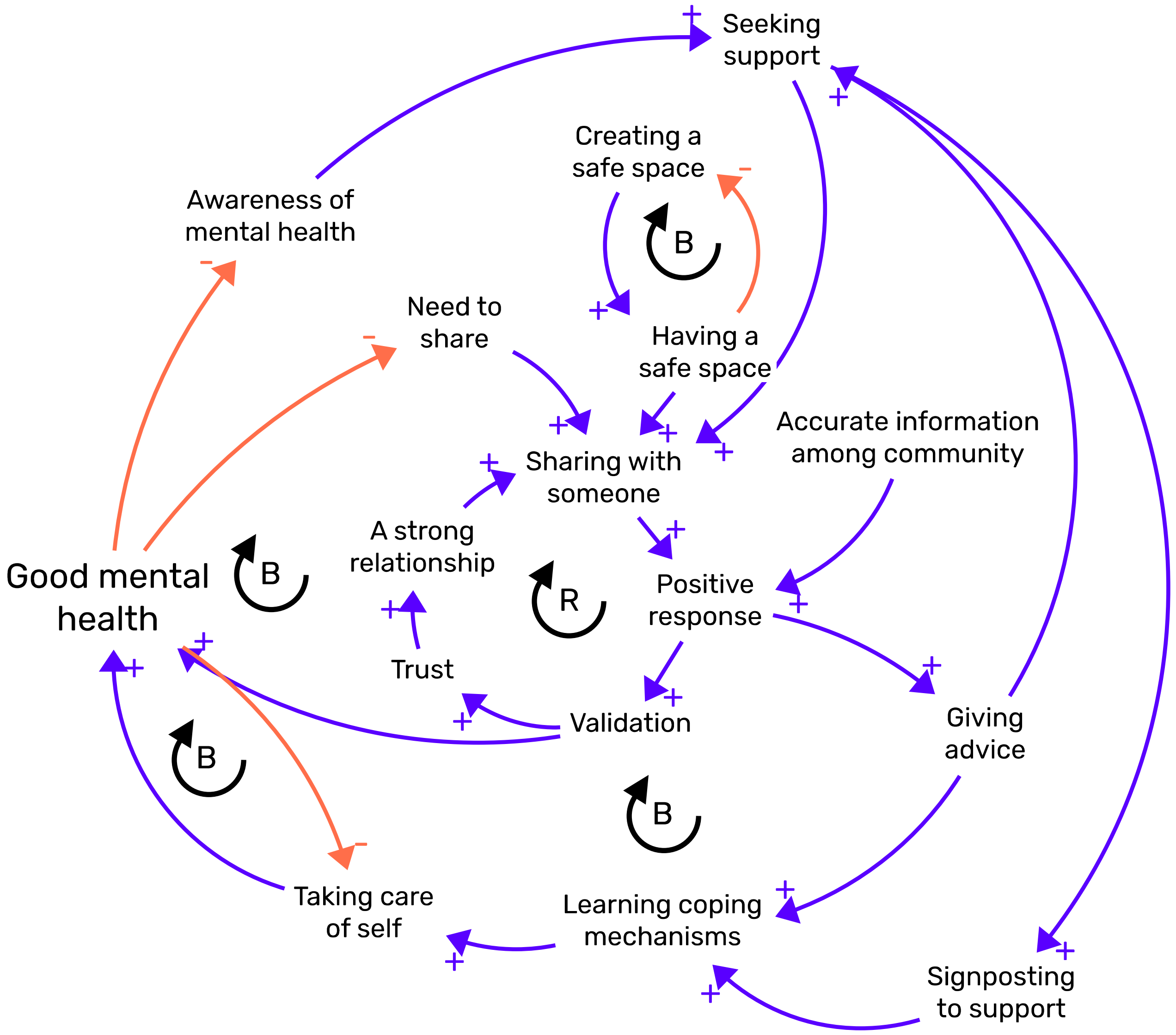

Support and care

The role of trusted relationships was greatly expanded upon by the Big Circle and in later Small Circle sessions.

Members in both groups talked about the importance of building these relationships through positive responses to young people which build their trust. These strong, trusting relationships mean they are more likely to share when their mental health is bad and receive advice, be signposted to additional support, or learn good coping mechanisms.

Once these relationships were built, they were self-reinforcing. This could be positive, where strong relationships made young people more comfortable talking about their mental health, but they could also mean that an early negative reaction was later reinforced.

The Big Circle also emphasised the importance of young people being engaged in a safe space, and highlighted how professionals are proactive in creating and meeting young people in these spaces.

Learning self-care

Members of the Big Circle found the information about coping mechanisms resonated with their experience of working with young people. They emphasised the role of a good understanding of mental health

They also highlighted the social role of short-term or “unhealthy” coping mechanisms, and how they can seem normal to young people.

They also provided more context for the sources of support young people can access other than counselling services. These can focus on the building the young person’s capacity to understand and respond positively when their mental health is bad.

Participants also commented that understanding of mental health is not only important for young people to help themselves, but it can also shift how they treat each other.

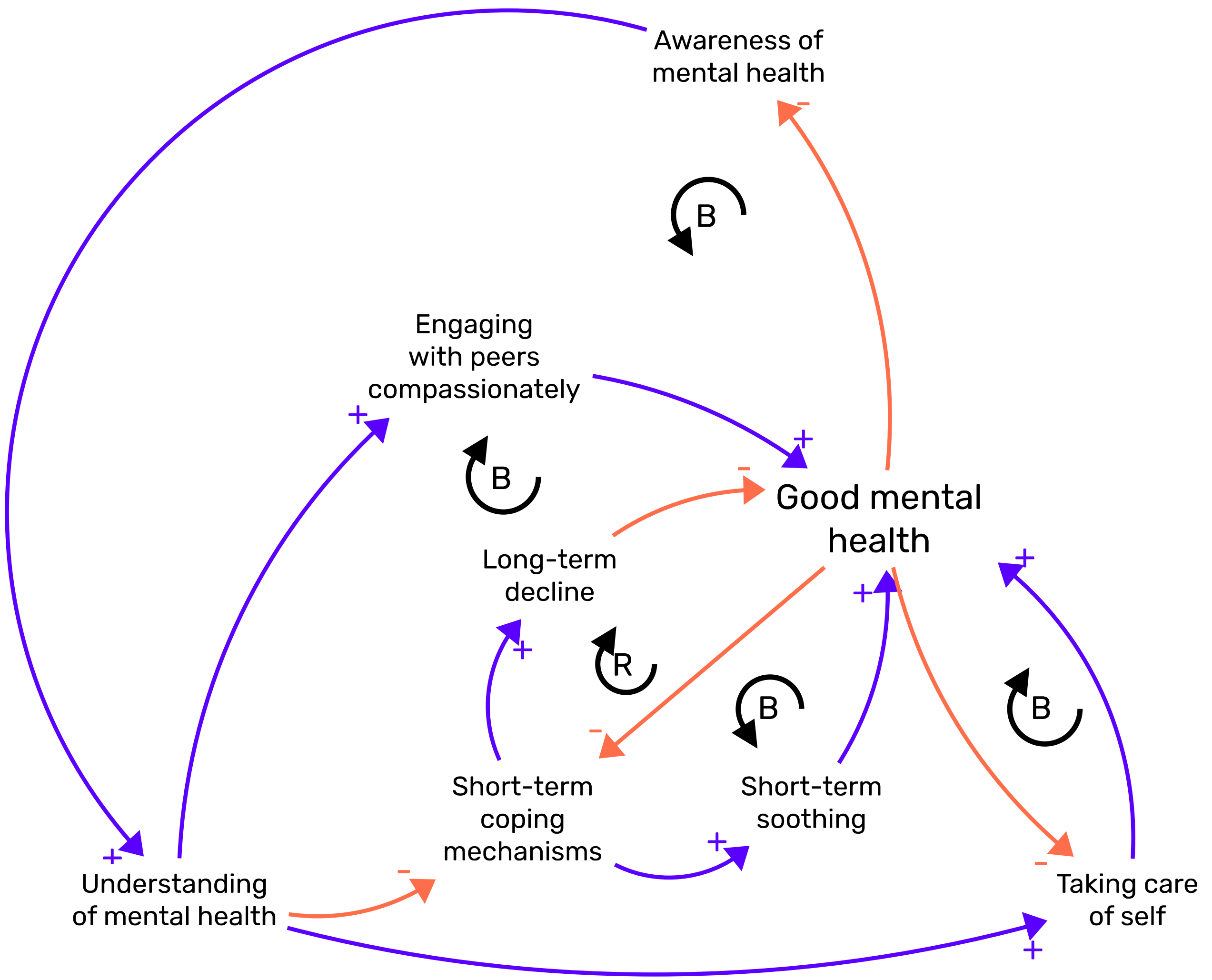

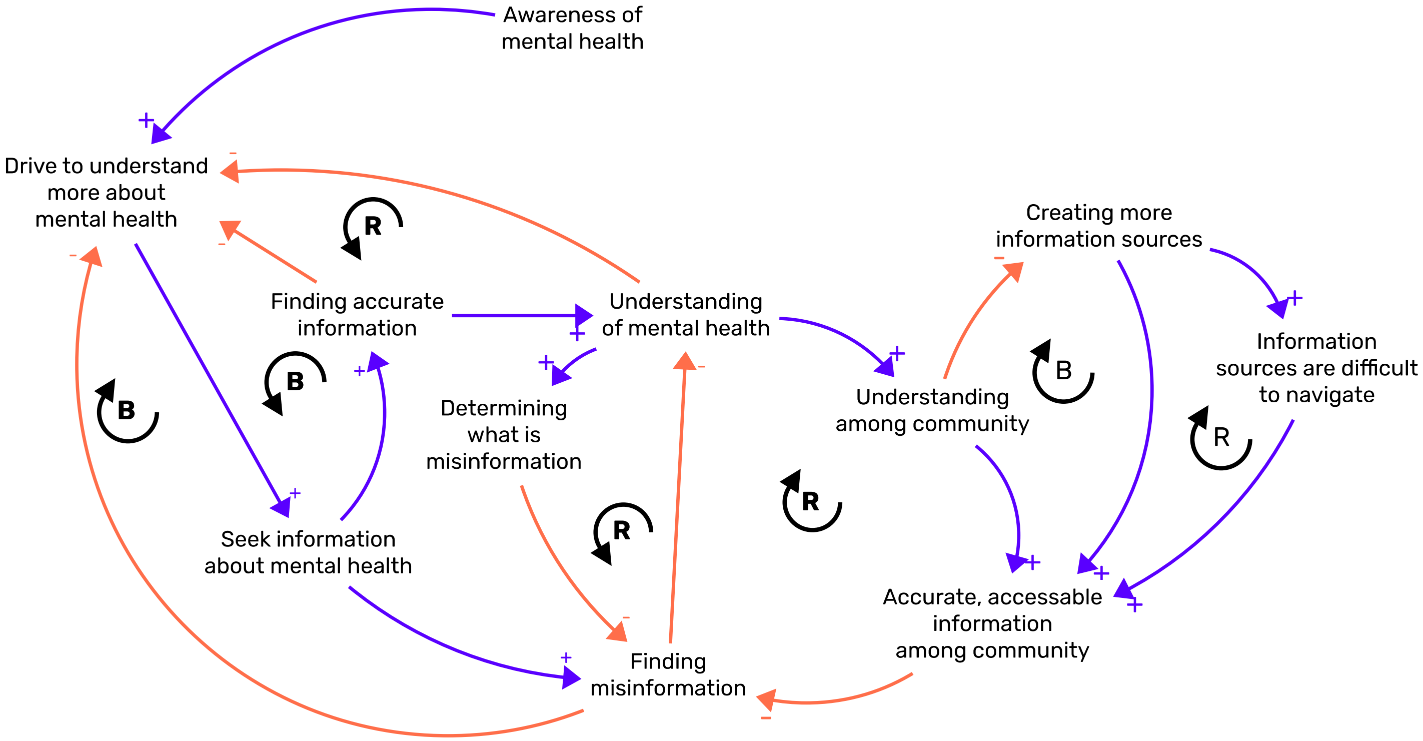

Understanding and awareness

Early in the Small Circle sessions there was a lot of discussion about how young people and others in the community come to understand mental health. Young people and others in the community seek information to understand mental health. Whether they find accurate information depends on the information available to them.

The previous Big Circle meeting flagged the role of medicalisation of mental health as a possible barrier to understanding.

Furthermore, they flagged the bind that those in the community seeking to provide more information can find themselves in when producing new information resources can make it “noisier” and more difficult to assess and process information.

Those in the Big Circle emphasised the importance of the social context young people find themselves in for understanding and processing information. This is explored more in the section on support and care.

Social media

Social media was raised a lot in the Small Circle sessions. Typically, it was brought up in a negative way: where it was used as a short-term coping mechanism or where it exposed young people to bullying.

Parents in the Small Circle and members of the Big Circle talked about how their children's use of social media caused them concern. This might cause them to limit their child's use of social media.

However, young people recognised that social media could play positive roles in young people's lives. Firstly, it could be a way of connecting with other young people and forming relationships would support them in their mental health.

Secondly, social media was a source of information around mental health for young people, though they recognised that this information may not be accurate.

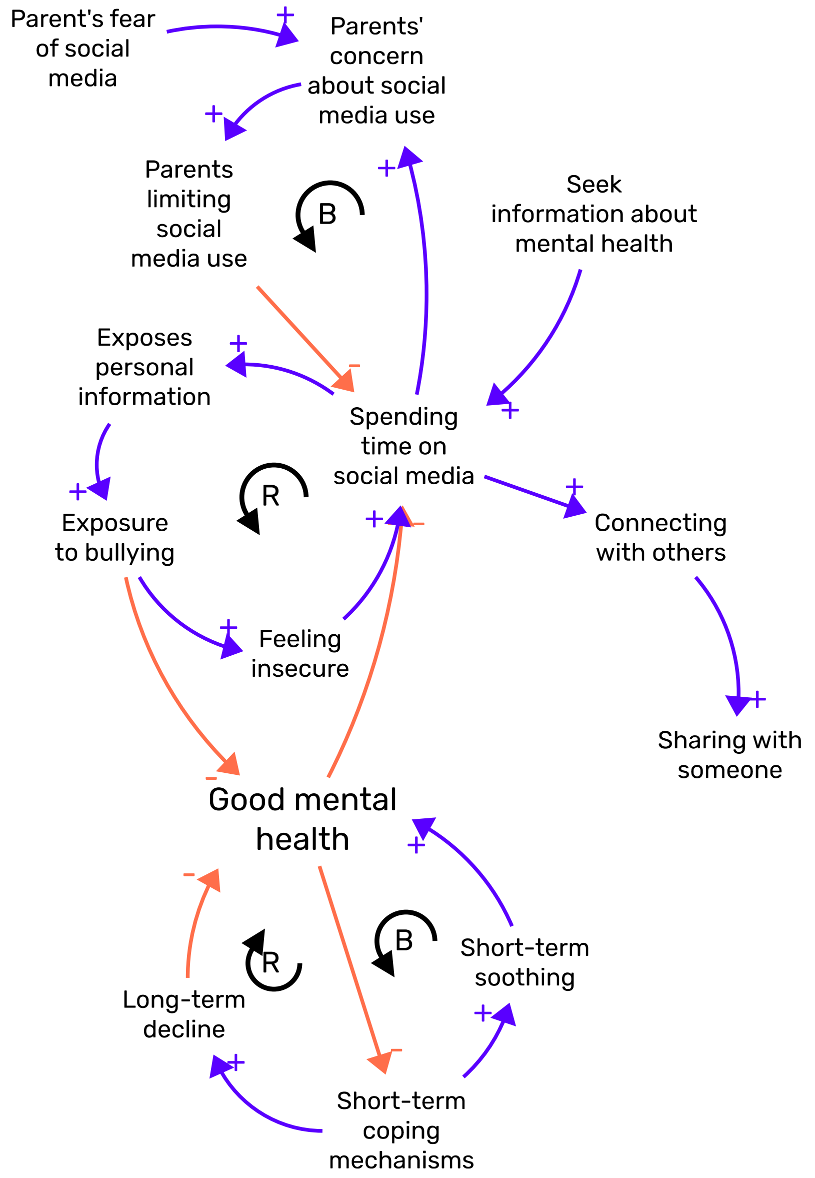

dartington.org.uk
info@dartington.org.uk
@DartingtonSDL
(01803) 762400

How might we inspire, support and connect young people to a diverse range of opportunities, jobs and careers?

Bideford Small Circle Opportunity Area

dartington.org.uk
info@dartington.org.uk
@DartingtonSDL
(01803) 762400

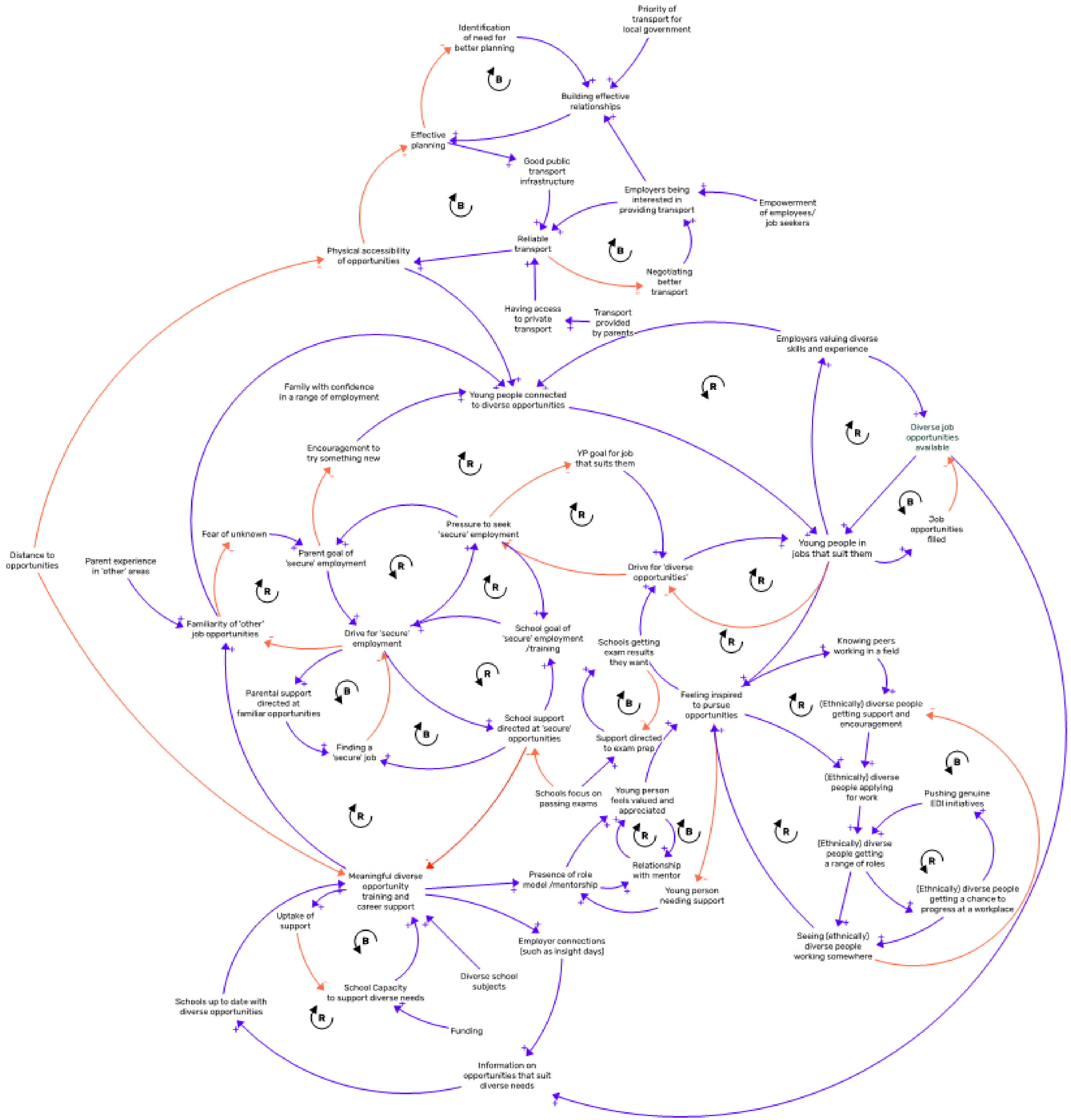

DARTINGTON
SERVICE
DESIGN LAB

IMPROVING SERVICES FOR CHILDREN & YOUNG PEOPLE

dartington.org.uk
info@dartington.org.uk
@DartingtonSDL
(01803) 762400

Employers valuing diverse skillsets

The Big Circle talked about the relationship between employers valuing diverse skills young people being connected with a diverse opportunity.

They raised the role of screening CVs as a barrier to employers meeting young people with skills and experiences which are not represented in qualifications.

They also tied the emphasis on qualifications back to schools' focus on exam results. When employers are focused on this, schools focus more on exams. This creates a narrative for young people early on that exams are the most important factor for finding a job later on in life.

They also expanded how employment fairs can be an opportunity for showcasing wider skillsets to employers.

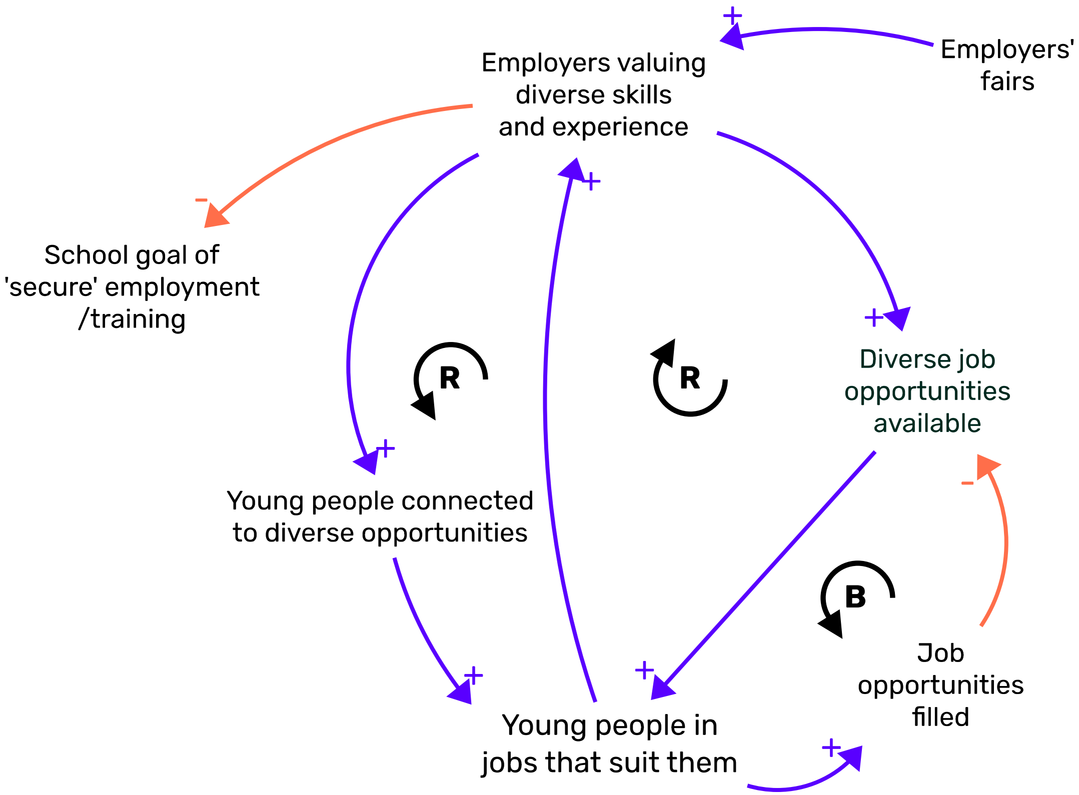

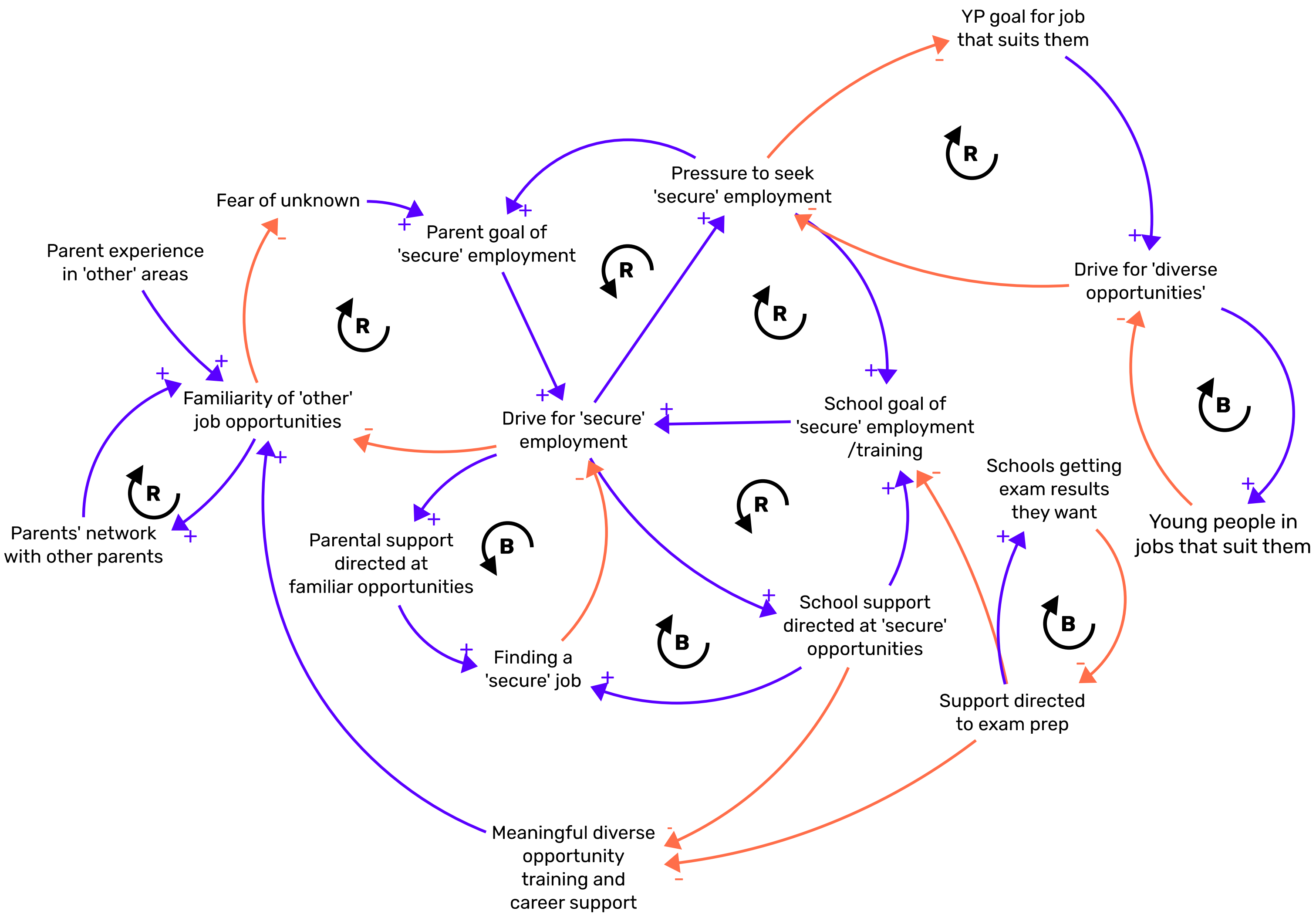

Competing interests

The Big Circle reflected that there wasn't much targeting parents related to employment. Some schools may support parents to help their children find work, but this was not widespread.

They also discussed how parents' wider networks can help them find opportunities for their children, but these networks, but that there weren't system-level interventions looking to improve this.

Counter to the reflections in the Small Circle, members of the Big Circle flagged their concerns about a relative decline in the focus on STEM subjects in schools.

dartington.org.uk
info@dartington.org.uk
@DartingtonSDL
(01803) 762400

Public transport and infrastructure

The Big Circle added to the understanding of public transport by building on a discussion from the Small Circle around the confidence of young people to use public transport. Young people who were used to getting lifts from parents were less likely to feel confident traveling by public transport for work. This use and confidence was also impacted by Covid, historically because of lockdowns but also because of ongoing concerns about the safety of confined public spaces.

Another issue they raised was the impact of schemes to make public transport cheaper or free for young people. This was believed to be positive, but it wouldn't overcome the more impactful issue of the transport being unreliable and therefore untenable for public use.

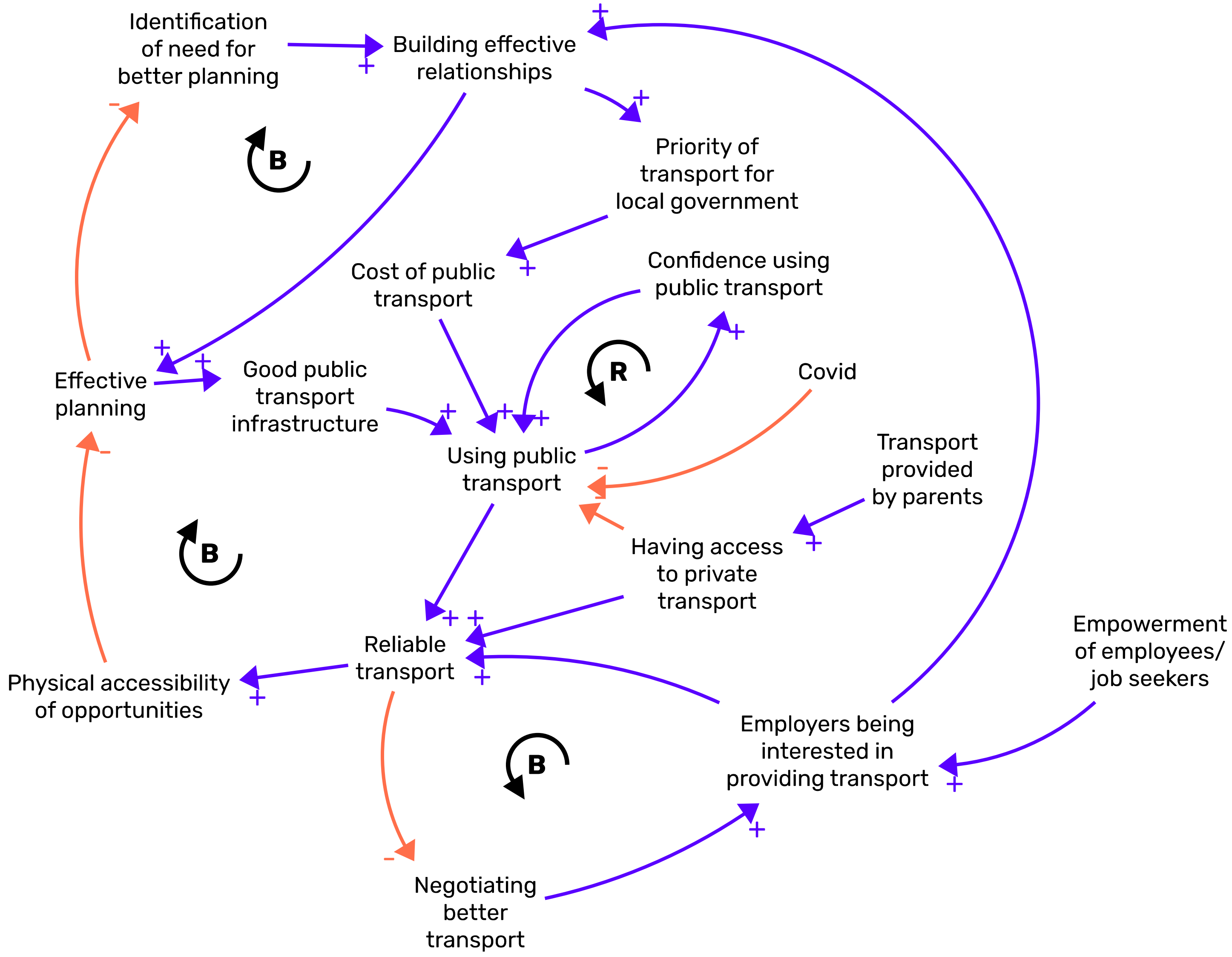

dartington.org.uk
info@dartington.org.uk
@DartingtonSDL
(01803) 762400

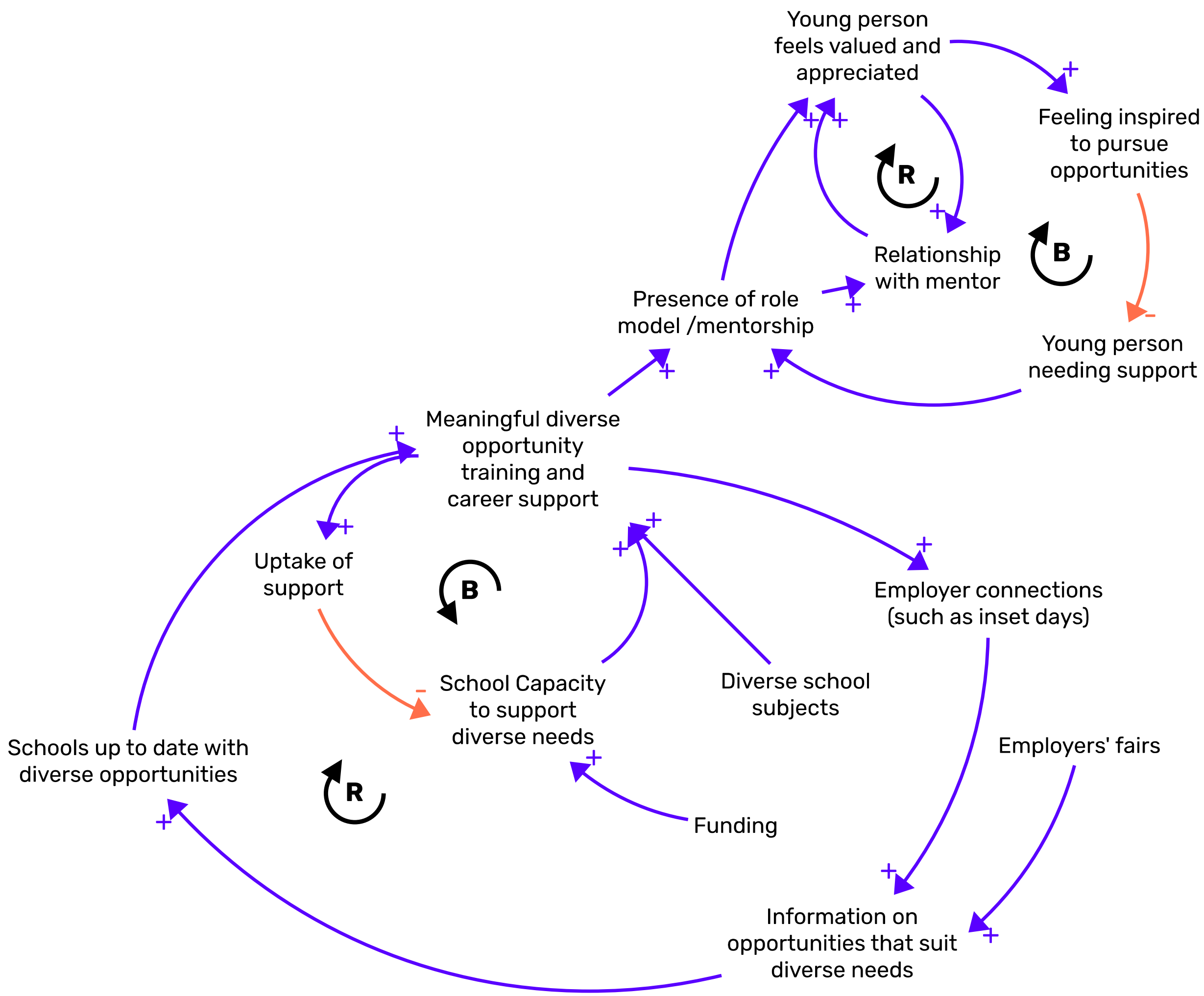

Career support and mentorship

The Big Circle focused on how the support that schools provide to young people is not up to date, not only with the local jobs available, but with the changing nature of careers more widely.

They also highlighted the impact on young people's mental health of the pressure to make good decisions early on. They focused on the importance of young people feeling like choices around employment were seen as opportunities rather than constraints.

Support from peers

The Big Circle talked about how young people might be connected with others who have experience navigating the system. This was seen to be very important not only for inspiring but connecting young people and ‘opening doors’. Knowing others working in a field could come through volunteering initiatives.

They also emphasised the importance of representation for SEND young people (seen in module "inspired diversity”).

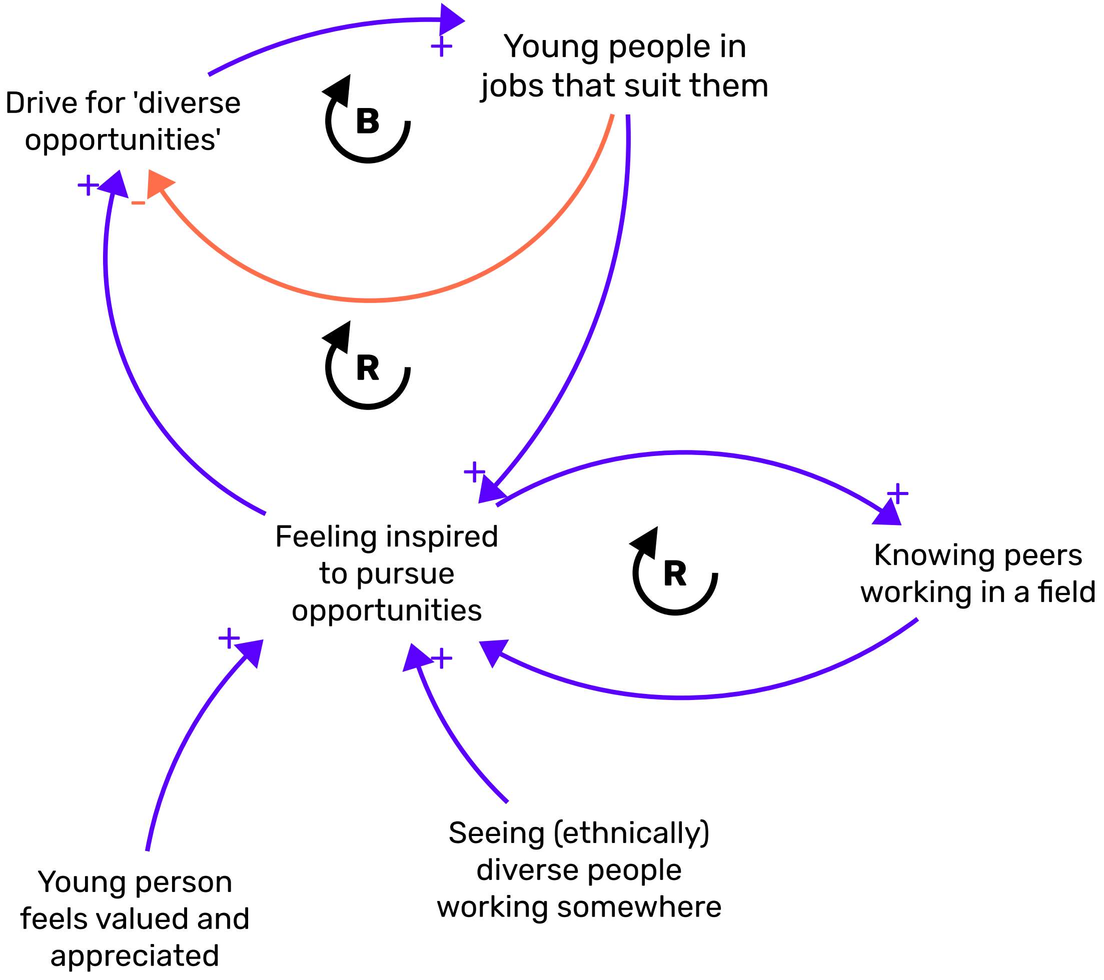

dartington.org.uk
info@dartington.org.uk
@DartingtonSDL
(01803) 762400

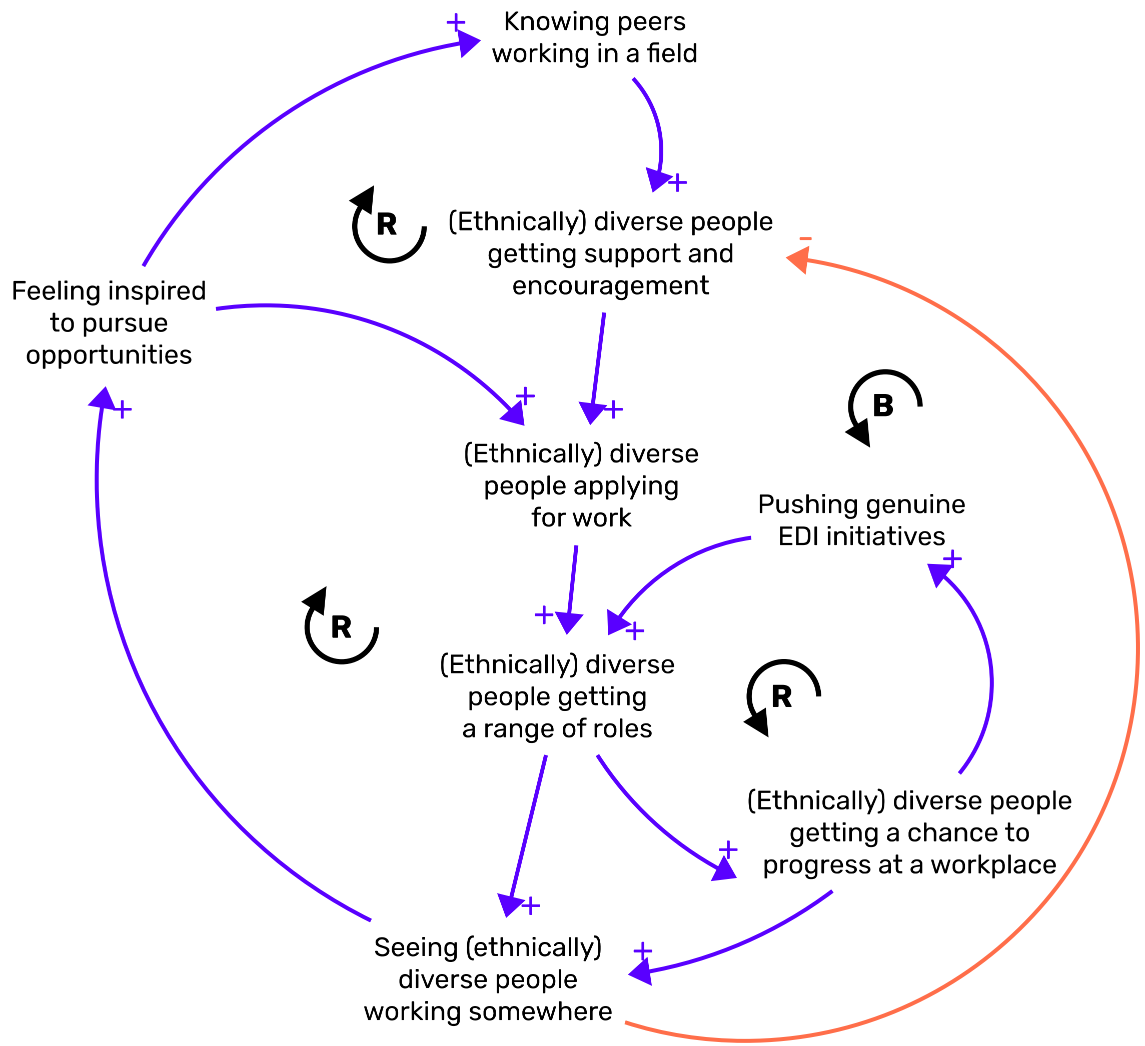

Inspired diversity

The Small Circle provided feedback on the part of the system map about diversity. This included broadening it out beyond just racial or ethnic diversity to include diversity in multiple ways. In particular, one young person mentioned an experience of their friend and disability.

Small Circle members agreed that diversity along one area would translate to other areas (e.g. a disabled person feeling more inspired to work somewhere that was more ethnically diverse).

This relates to the discussion in the Big Circle about the importance of SEND young people feeling like a workplace was representative of people like them.

dartington.org.uk
info@dartington.org.uk
@DartingtonSDL
(01803) 762400
