# Supplementary material for: An adaptive, youth-centred co-design methodology: place-based co-design centring youth and community participation
Source: Res Involv Engagem. 2026 Jan 24;12:33. doi: 10.1186/s40900-025-00833-w (PMC12994241; doi:10.1186/s40900-025-00833-w)
Supplement: Supplementary file 16 — Supplementary Material 16 [file 40900_2025_833_MOESM16_ESM.pdf]

Activities for Wellbeing System Maps

Big Circle Meeting

19.01.2024

Kailo

Group Model Building

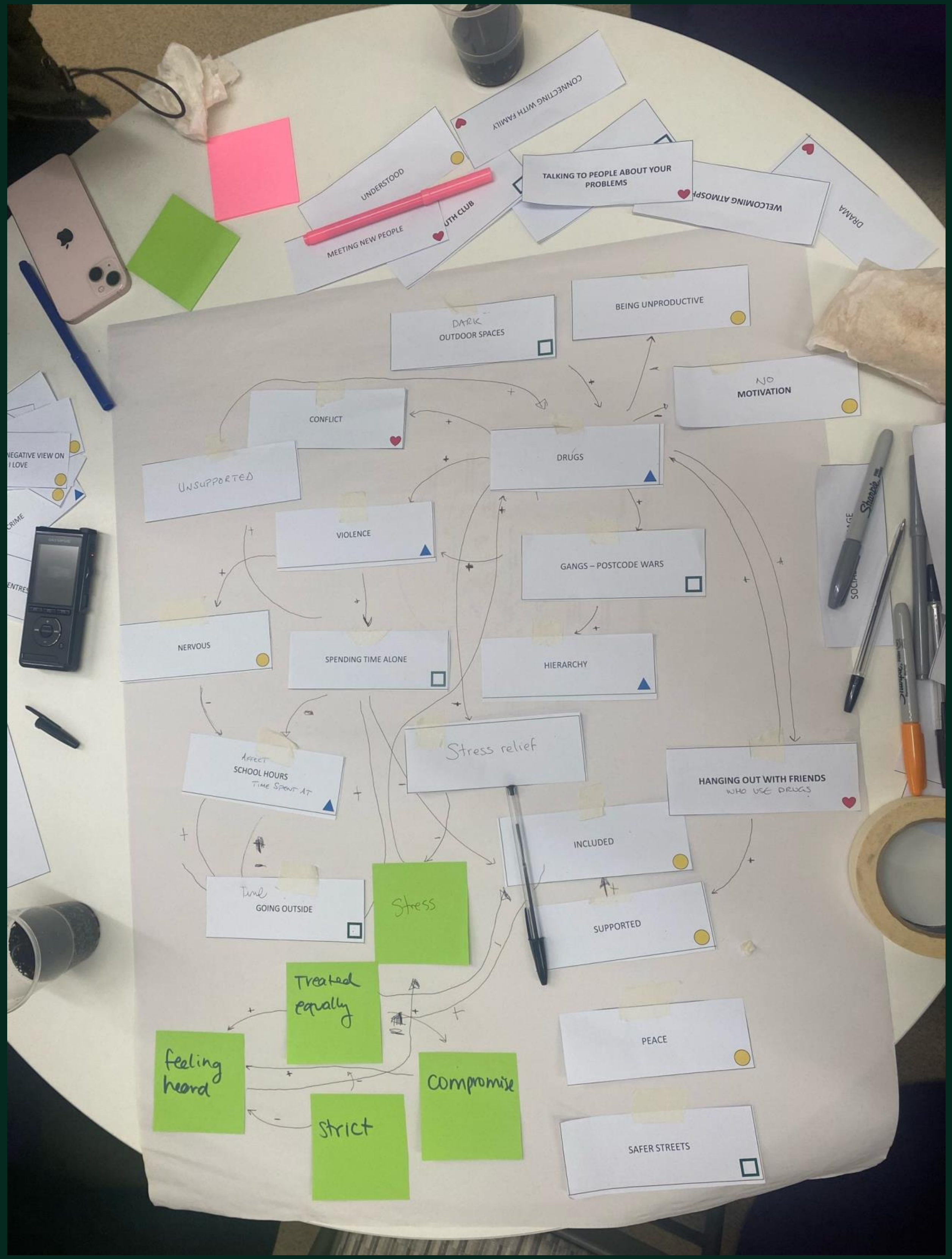

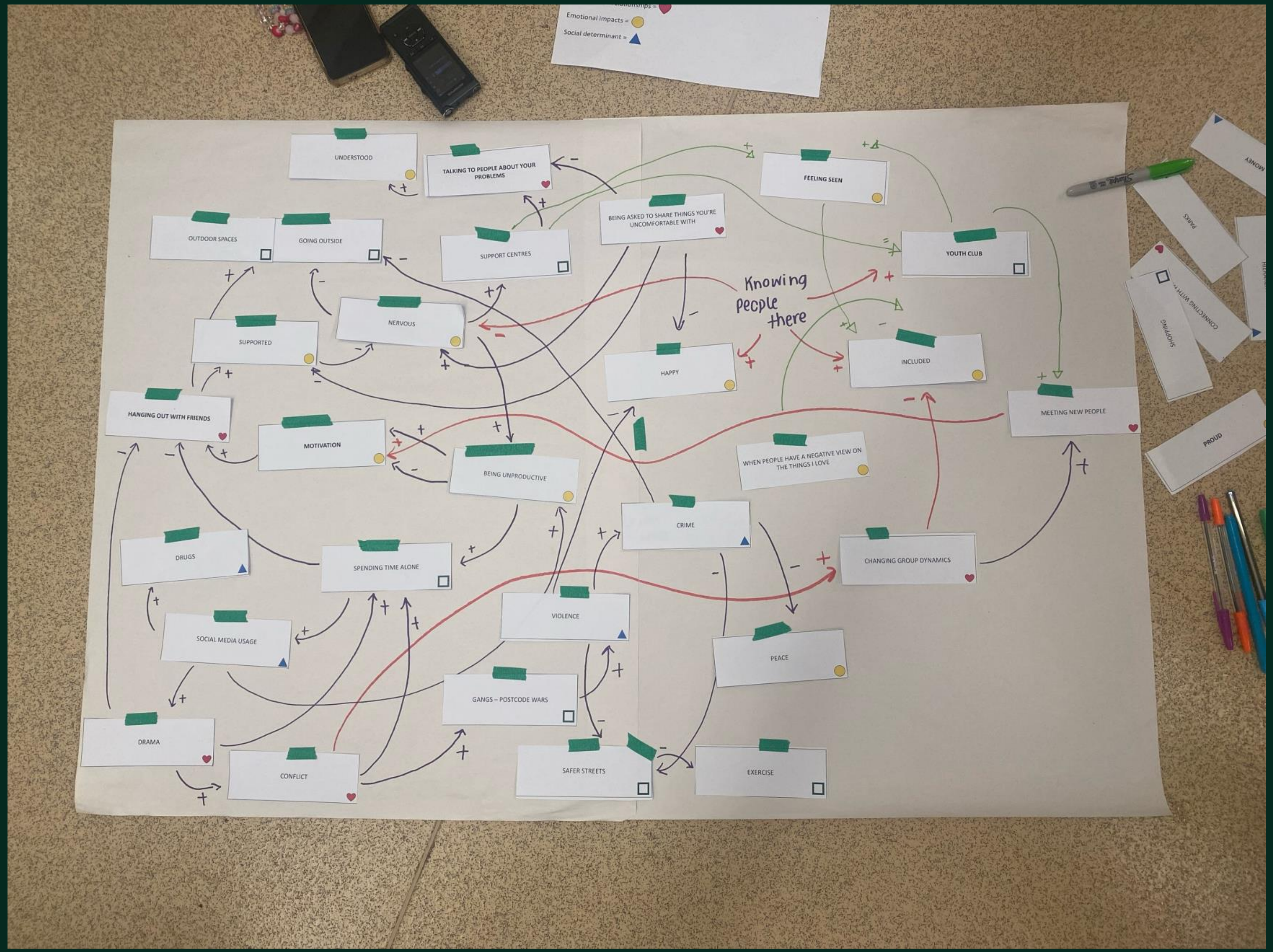

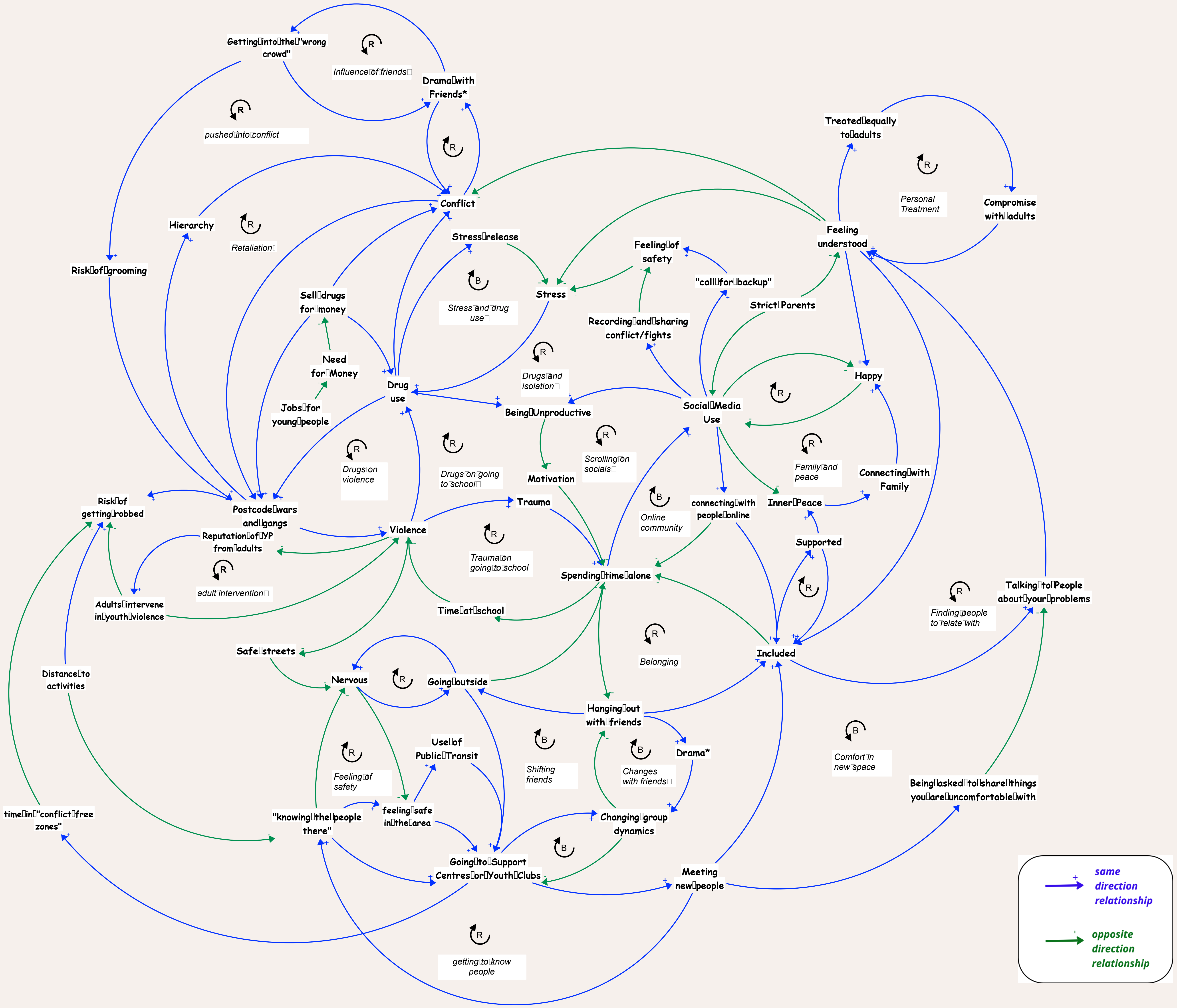

Getting into the "wrong crowd"
Influence of friends
Drama with Friends*
pushed into conflict
Conflict
Hierarchy
Retaliation
Risk of grooming
Stress release
Stress
Stress and drug use
Sell drugs for money
Need for Money
Jobs for young people
Drug use
Feeling of safety
"call for backup"
Recording and sharing conflict/fights
Strict Parents
Treated equally to adults
Personal Treatment
Compromise with adults
Feeling understood
Happy
Drugs and isolation
Being Unproductive
Social Media Use
Drugs on violence
Drugs on going to school
Scrolling on socials
Family and peace
Connecting with Family
Motivation
Online community
connecting with people online
Inner Peace
Risk of getting robbed
Postcode wars and gangs
Reputation of YP from adults
Violence
Trauma
Trauma on going to school
Supported
adult intervention
Adults intervene in youth violence
Spending time alone
Time at school
Talking to People about your problems
Finding people to relate with
Belonging
Included
Safe streets
Distance to activities
Nervous
Going outside
Hanging out with friends
Use of Public Transit
Shifting friends
Changes with friends
Drama*
Comfort in new space
Feeling of safety
Being asked to share things you are uncomfortable with
time in "conflict free zones"
"knowing the people there"
feeling safe in the area
Changing group dynamics
Going to Support Centres or Youth Clubs
Meeting new people
getting to know people
same direction relationship
opposite direction relationship

Kailo

Key Dynamics of the System Map

Kailo

1

01.
Comfort in New Spaces

As more young people attend support centres and youth clubs, they have greater opportunity to meet new people. While allows for them to feel more included, it also increases the risk of ”being asked to share things [they] are uncomfortable with”. When they feel more included it provides them greater opportunity to talk to people about their problems. However, if they are asked to share things they may feel more uncomfortable with it can lead to them being less willing to talk to people about their problems.

Talking to people about their problems is central to feeling understood and included. When young people feel this way they spend less time alone, more time with friends, and are more likely to engage in support centres of youth clubs.

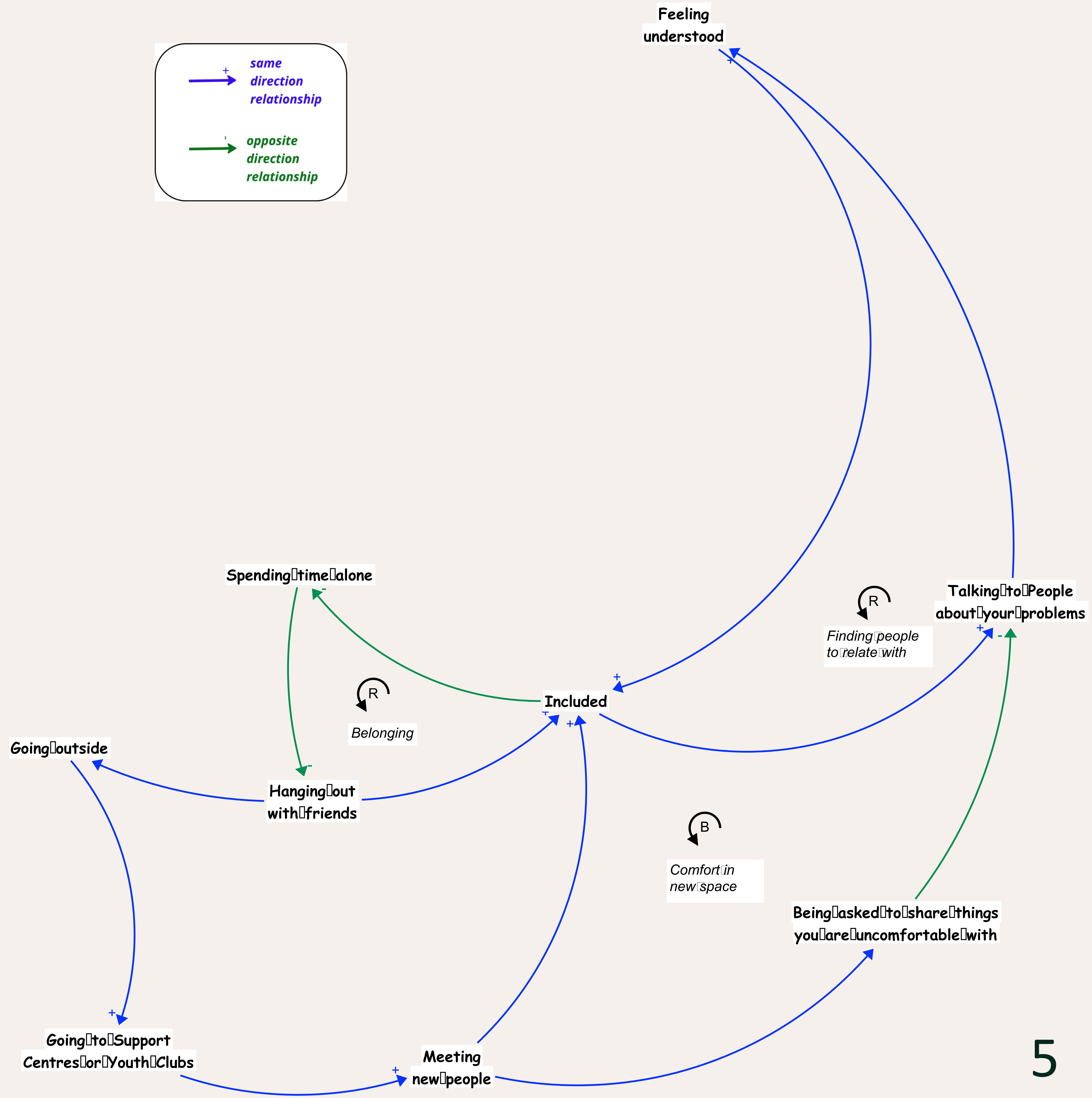

02.
Safety Engaging in Activities

The safety of the area around activities for wellbeing is a key determinant for young peoples' positive experience and engagement in activities and spaces.

Increased violence-–influenced by factors such as postcode wars—makes for less safe streets. This increases young peoples' nervousness and decreases their sense of safety, making them less likely to go to support centres and youth clubs. They may also engage less in public transit if they feel unsafe thereby making it more challenging for them to go to activities for wellbeing.

Distance also has a major impact on young peoples' sense of safety. The further away young people are from their home, the more they are at risk of being robbed and the less likely they are to "know people". This is a barrier to them feeling safe and wanting to engage with activities and supports outside of their hyperlocal area.

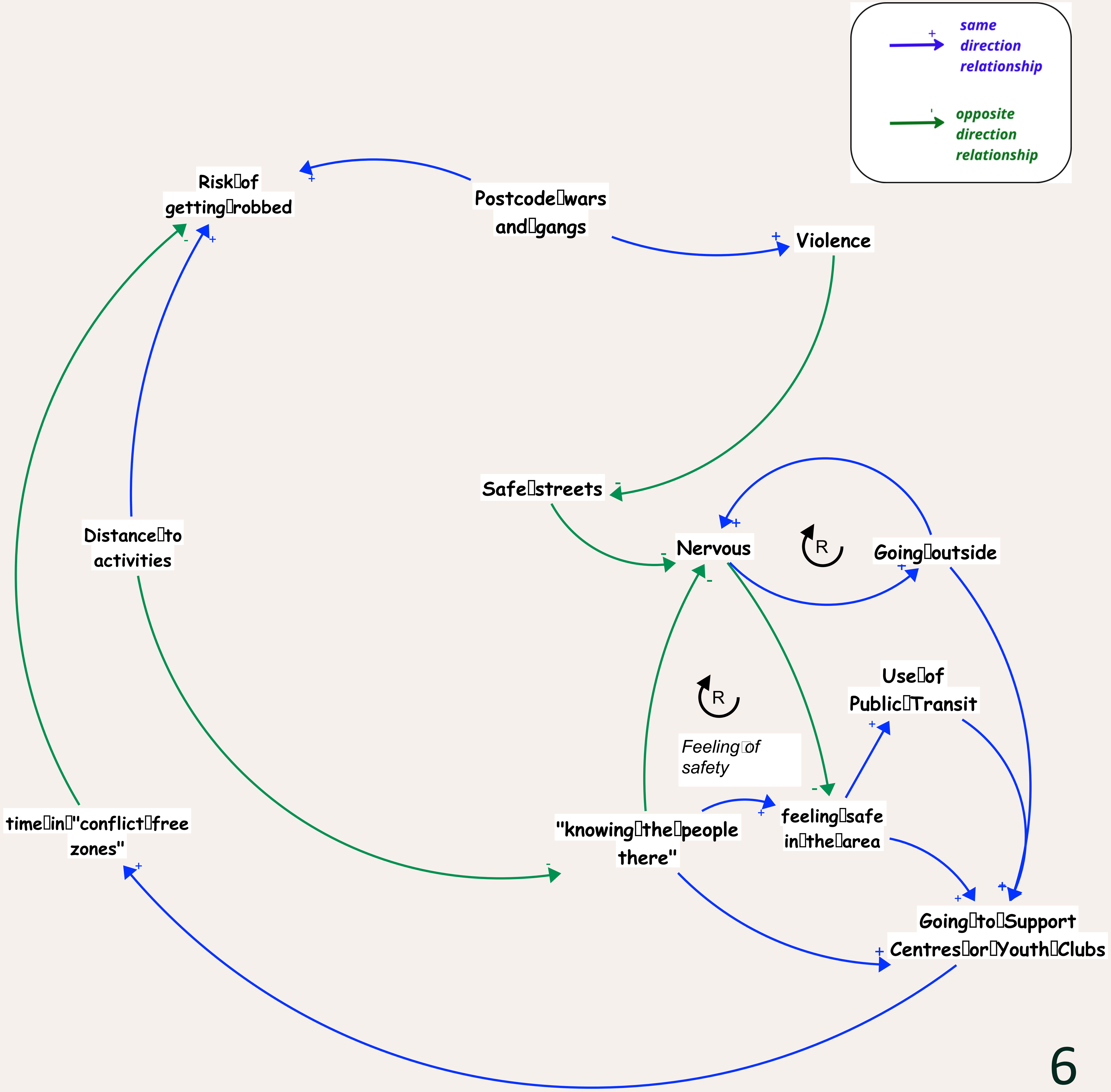

03. Reputation of Young People and Violence

Young people described how the more young people are perceived as violent, the less adults are willing to intervene. This leaves young people more vulnerable things like to violence and being robbed.

This is further complicated by the prevalence of postcode wars and gangs. When there is higher involvement or activity of gangs and postcode wars, there tends to be more violence in the area. This influences the reputation of how young people are viewed by adults and makes them more fearful or less willing to intervene when they see a young person who may be experiencing violence.

As previously described, distance to activities also influences how much young people are at risk of violence in Newham. However, time in “conflict free zones” (such as safe spaces and activities), reduces their risk of being robbed or involved in violence.

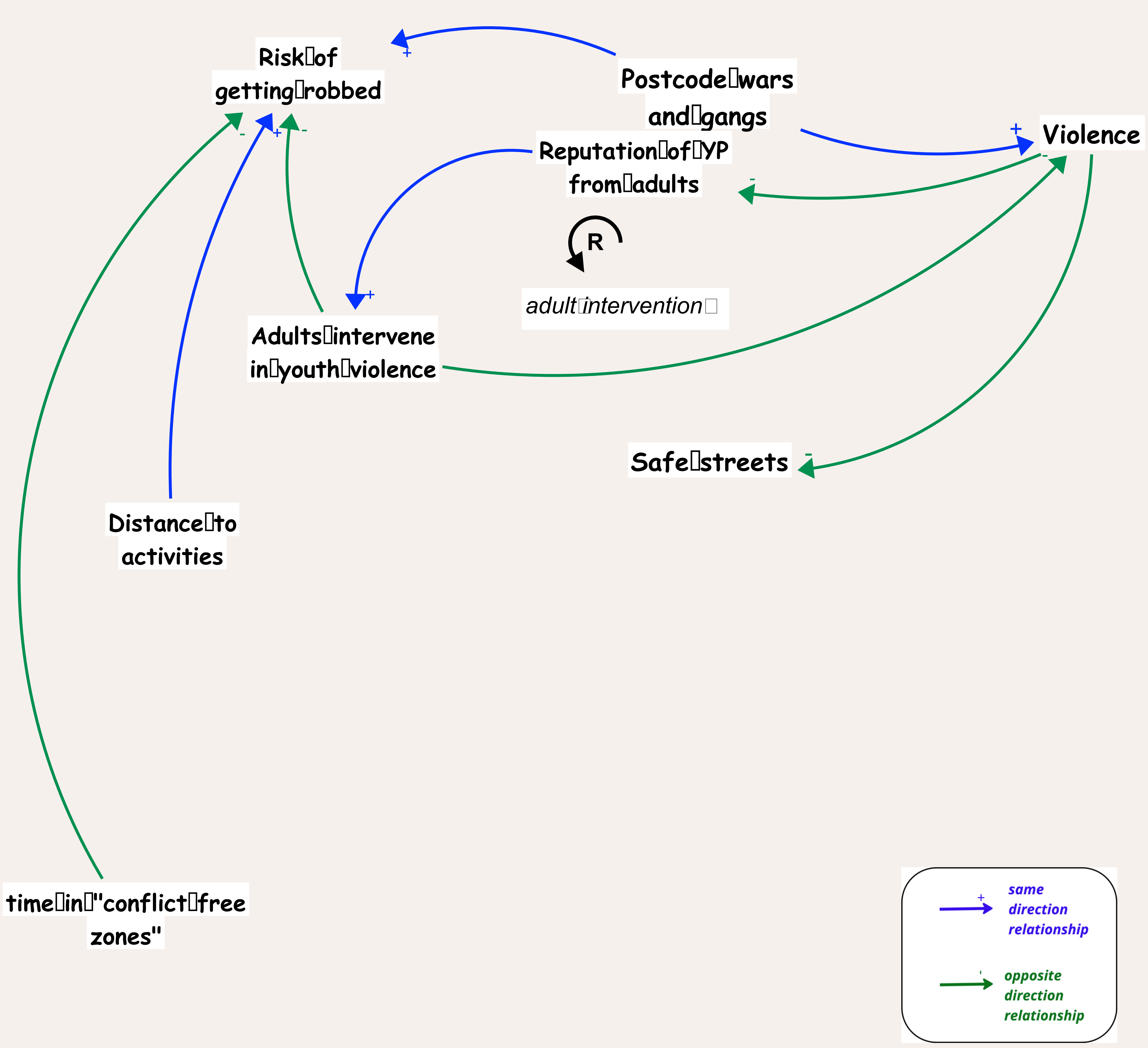

04.
Influences on Engaging in Conflict

Friends, peers, and “getting into the wrong crowd” were described to have an influence on young people getting involved in conflict and violence. When young people are surrounded by the “wrong crowd” they become more at risk for grooming-— which can lead to them becoming involved in postcode wars or gangs. Conflict and drama with friends is one way that young people may be influenced to get into ”the wrong crowd”.

When involved with certain groups, young people can become influenced by power dynamics and hierarchy which can lead to more conflict. This can become a cyclical pattern with conflict leading to retaliation and more conflict.

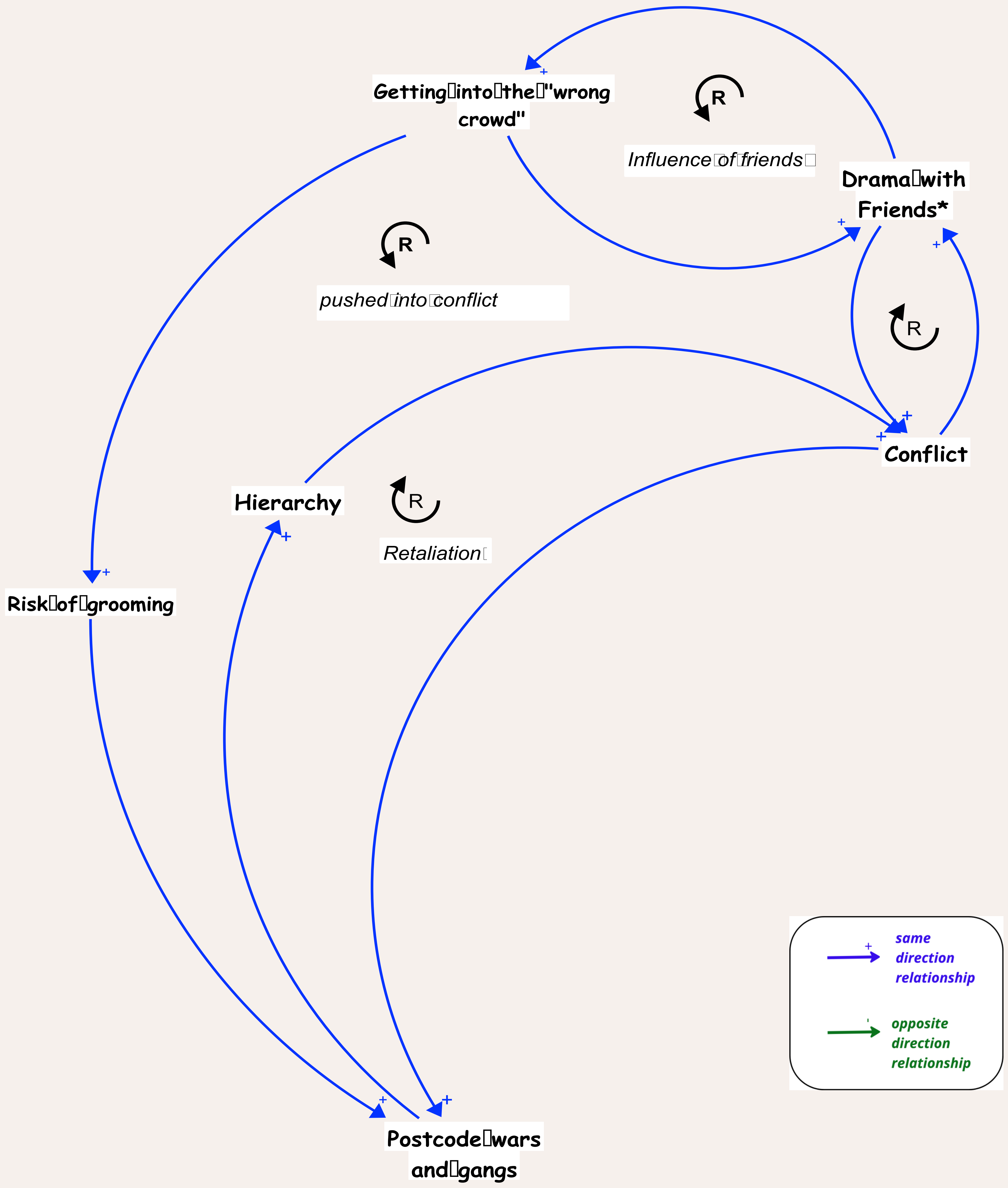

05. Stress, Isolation, and Belonging

A sense of belonging—feeling included, having people to talk about your problems with, and feeling understood—is critical for promoting the wellbeing of young people.

When young people don’t have that sense of belonging, or places and people where they feel understood, they tend to be more stressed, less motivated, and more likely to self isolate. This can lead them to withdraw from friends and school and lean towards things like drug use to release their sense of stress.

Experiences of violence can also perpetuate self isolation, as trauma may lead them to want to spend more time alone. When young people self-isolate they lose out on spaces for connection and inclusion which can may it demotivating to engage and connect with others.

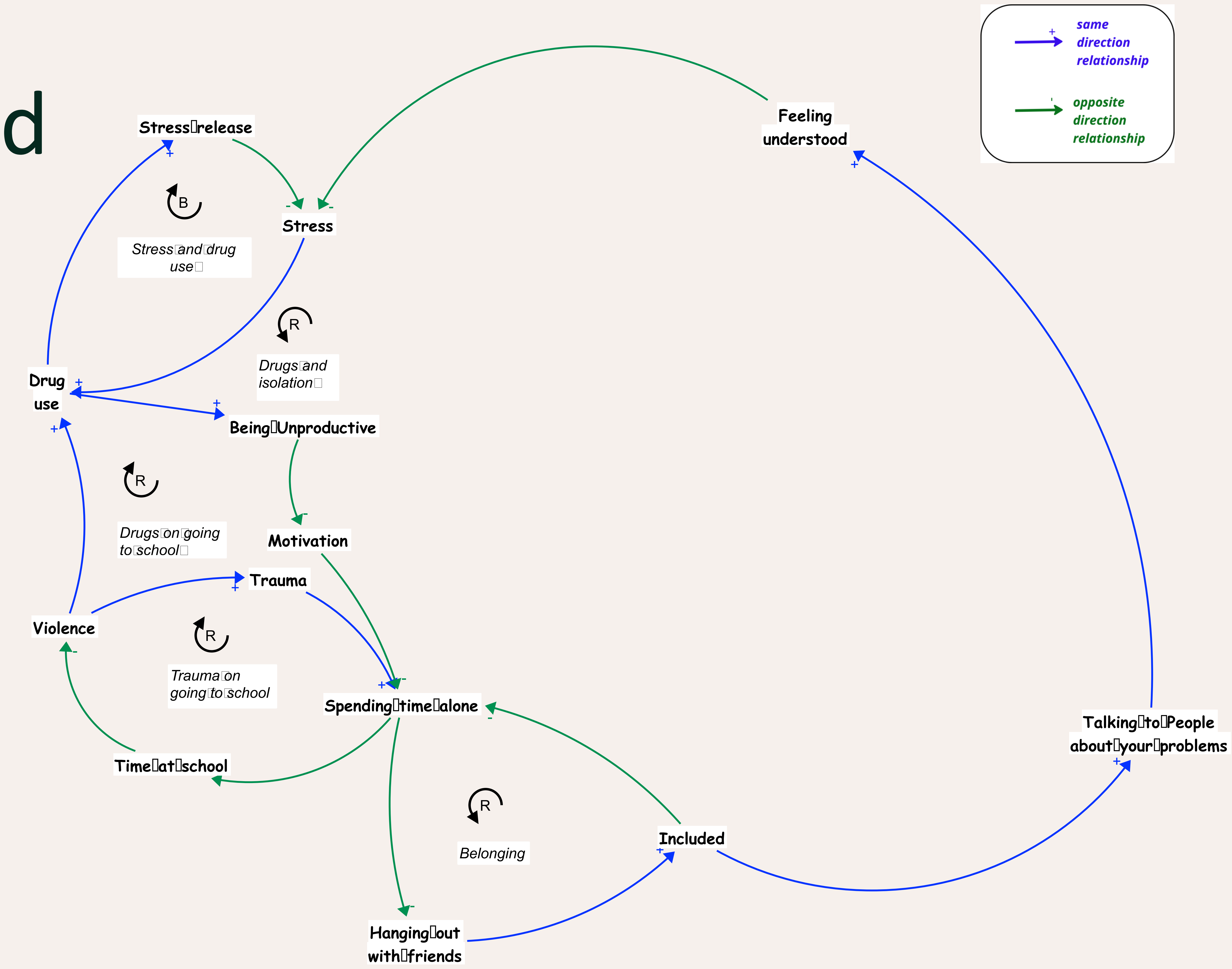

06.
Social Media and Connection

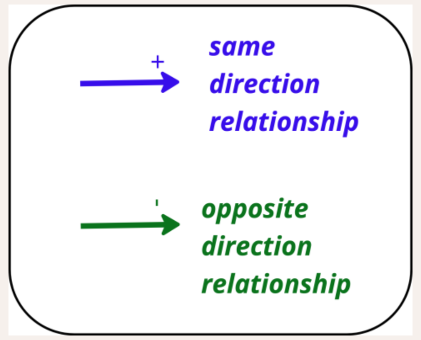

Self-isolation and spending time alone can lead to more time using social media. Young people in the small circle saw the use of social media to have varying influences on a sense of connection. In some ways social media can lead young people to be less productive and lose motivation—leading them to spend more time alone and feel less included and supported by friends.

However, social media can also give young people a space to connect with people online and build online spaces where they can feel supported and included.

Spending more time on social media, however, was also described to make young people feel less happy and lose a sense of inner peace. And the less happy they felt, the more likely they were to engage more with social media.

Finally, social media can also be a tool around experiences of violence. As young people described using snapchat to "call for backup" and also record or share conflict or fights. This impacts upon their feelings of safety and stress levels.

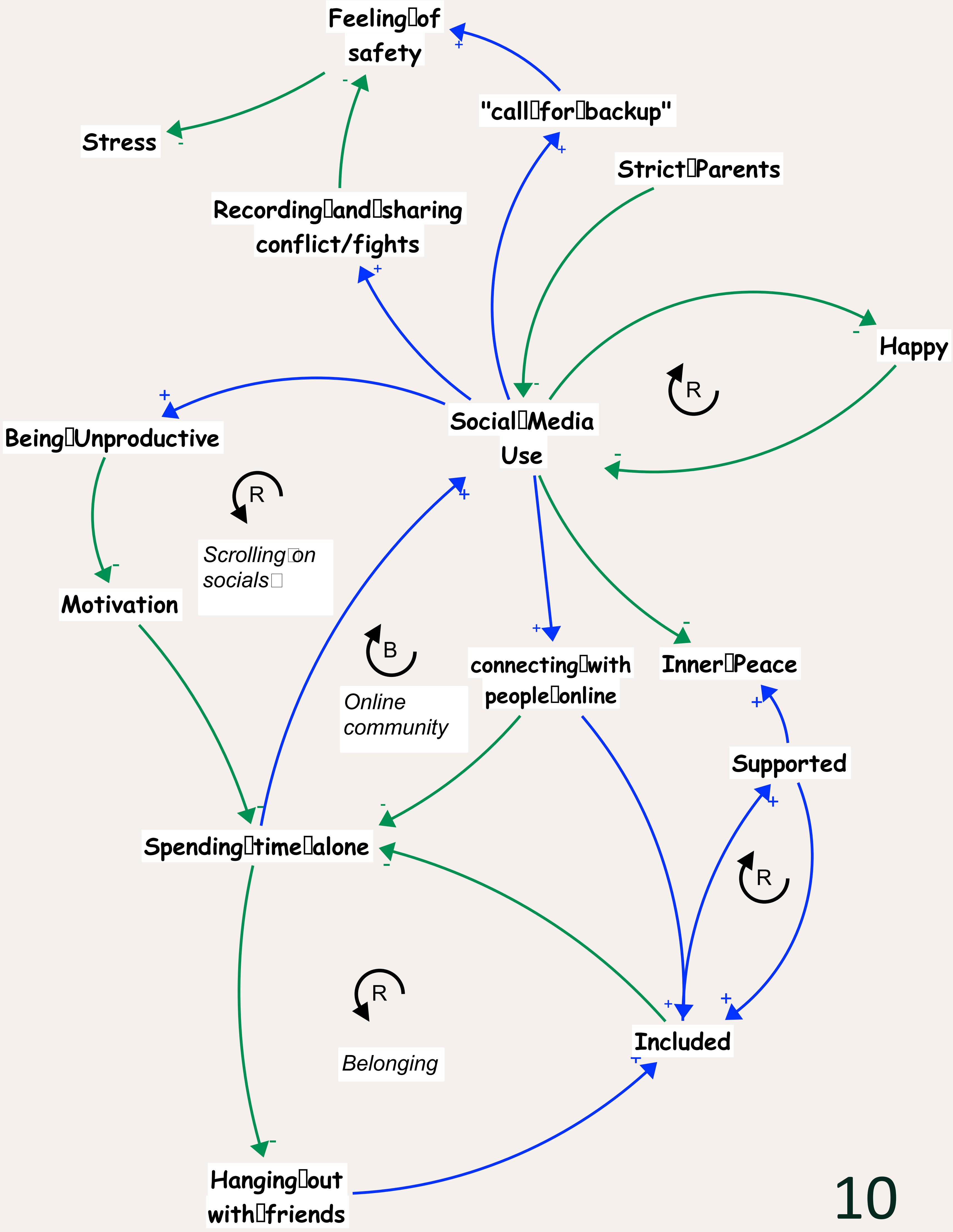

07. Connection with Parents and Adults

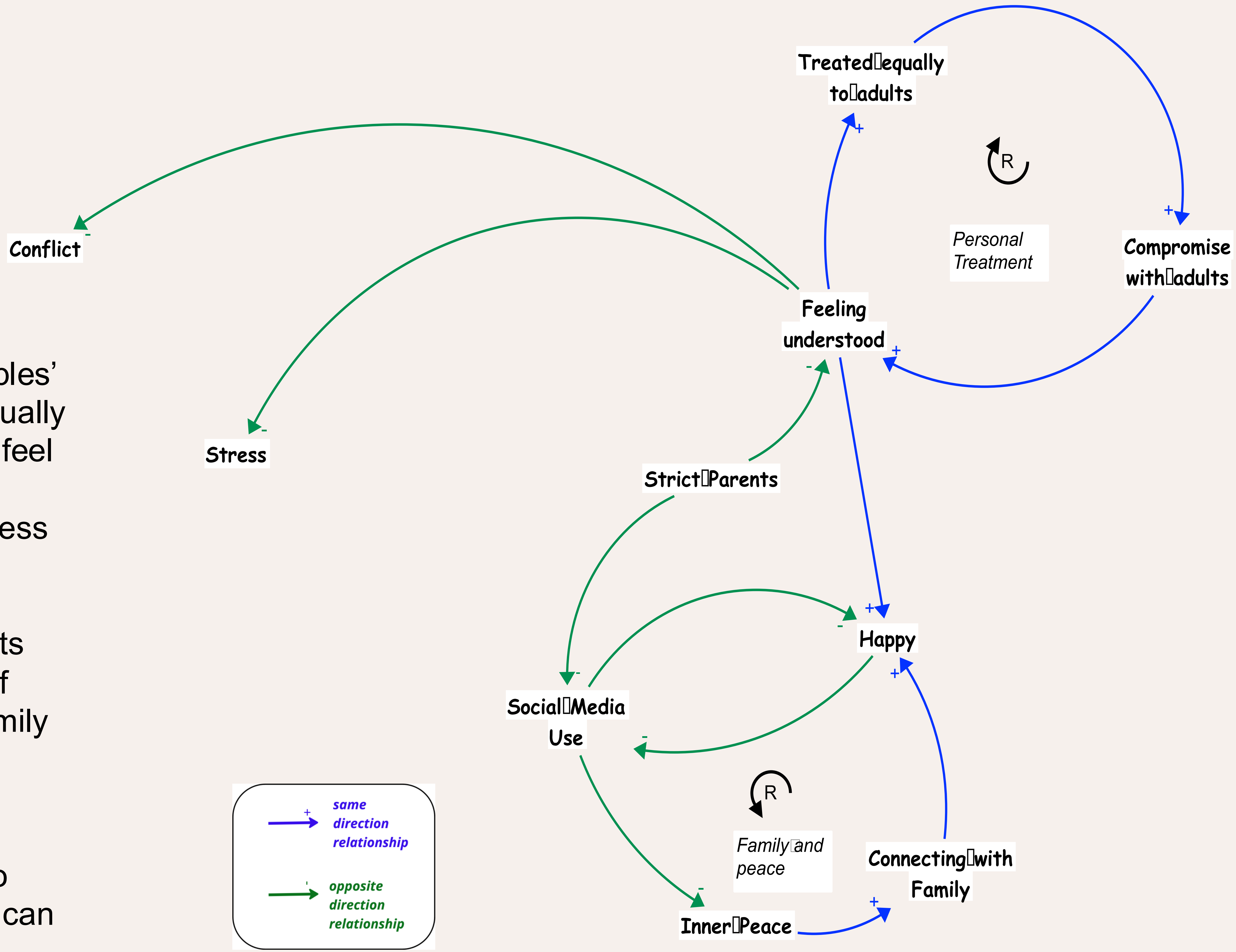

Feeling understood is a central factor shaping young peoples' wellbeing in Newham. When young people are treated equally to adults (and feel respected) then they are more likely to feel that their voice matters and can compromise and feel understood by adults. This supports their sense of happiness and reduces stress and conflict.

When young people feel understood and happy, it supports their sense of inner peace and can increase their sense of connection with their family. Feeling connected to their family is also central to supporting their mental wellbeing.

However, influences like strict parents may make young people feel less understood and may influence for them to engage less in spaces and social media platforms (which can have both supportive and unsupportive impacts on their wellbeing).

02
Key Insights

2

Kailo

Key Insights from the System Map

- **Feeling understood** is central to supporting young peoples' wellbeing in Newham.

- **Experiences and fear of violence** often drives young peoples' ability and willingness to engage in activities in the local area.
- It is harder and riskier to travel to activities and spaces further away from home or the area where you are known
- It is important to know the people you will see in the spaces you are engaging
- Perceptions of young people by adults can influence young peoples' sense of security

- Supporting a **sense of belonging** is essential for both young peoples' wellbeing and physical safety.

Violence and Crime System Maps

Big Circle Meeting

18.1.2024

Kailo

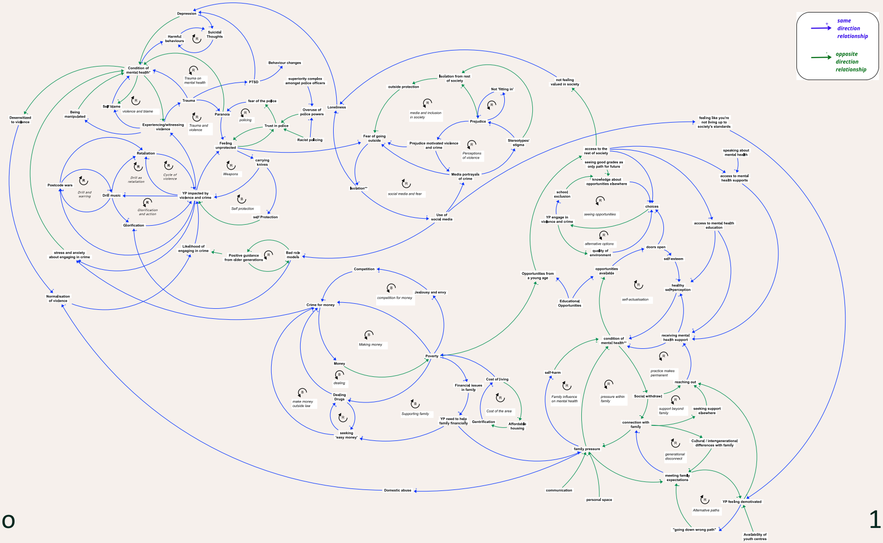
same direction relationship
opposite direction relationship
Depression
Harmful behaviours
Suicidal Thoughts
Condition of mental health*
Behaviour changes
PTSD
Trauma on mental health
superiority complex amongst police officers
Self blame
violence and blame
Trauma
fear of the police
Overuse of police powers
Loneliness
Being manipulated
Desensitized to violence
Experiencing/witnessing violence
Trauma and violence
Paranoia
policing
Trust in police
Racist policing
Feeling unprotected
Retaliation
carrying knives
Postcode wars
Drill and warring
Drill music
Drill as retaliation
Cycle of violence
YP impacted by violence and crime
Weapons
Glorification and action
Self protection
self Protection
Glorification
stress and anxiety about engaging in crime
Likelihood of engaging in crime
Positive guidance from older generations
Bad role models
Normalisation of violence
Isolation from rest of society
outside protection
Not 'fitting in'
media and inclusion in society
Prejudice
Fear of going outside
Prejudice motivated violence and crime
Perceptions of violence
Stereotypes/ stigma
Isolation**
Media portrayals of crime
social media and fear
Use of social media
not feeling valued in society
feeling like you're not living up to society's standards
access to the rest of society
seeing good grades as only path for future
knowledge about opportunities elsewhere
speaking about mental health
access to mental health supports
school exclusion
seeing opportunities
choices
YP engage in violence and crime
alternative options
quality of environment
doors open
self-esteem
access to mental health education
opportunities available
Opportunities from a young age
Educational Opportunities
healthy self-perception
self-actualisation
Competition
competition for money
Jealousy and envy
Crime for money
Making money
Money
dealing
make money outside law
Dealing Drugs
seeking 'easy money'
Poverty
Supporting family
Financial issues in family
Cost of living
Cost of the area
YP need to help family financially
Gentrification
Affordable housing
condition of mental health**
receiving mental health support
practice makes permanent
self-harm
Family influence on mental health
pressure within family
Social withdrawal
reaching out
support beyond family
seeking support elsewhere
connection with family
Cultural / inter-generational differences with family
generational disconnect
family pressure
Domestic abuse
communication
personal space
meeting family expectations
Alternative paths
YP feeling demotivated
"going down wrong path"
Availability of youth centres

Kailo

15

Group Model Building

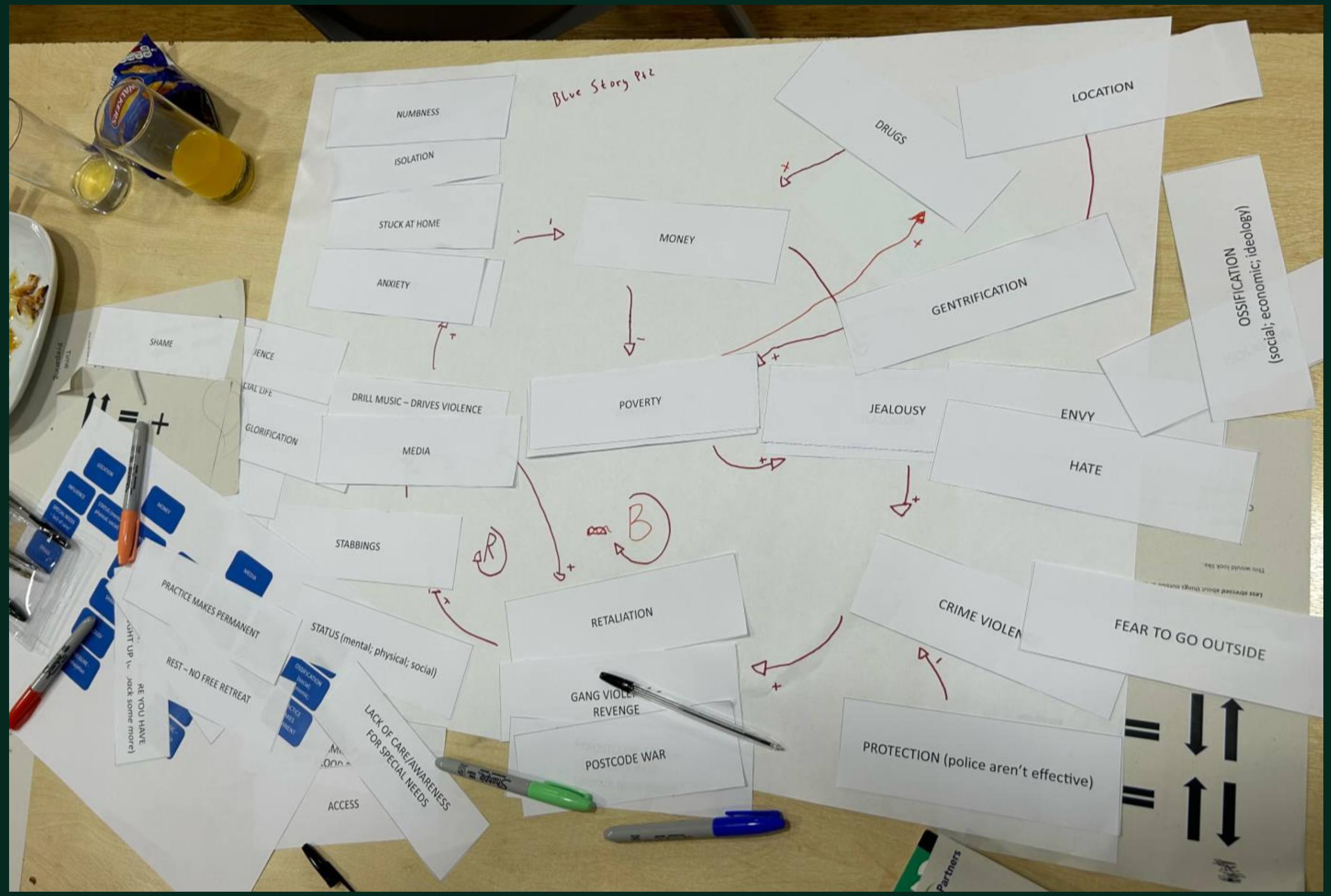

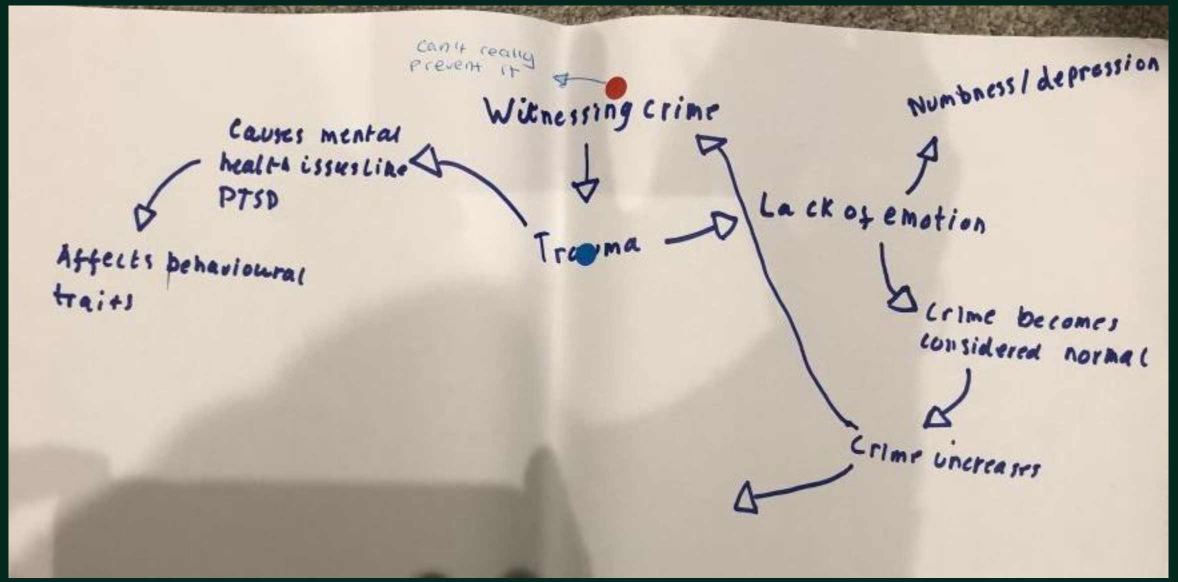

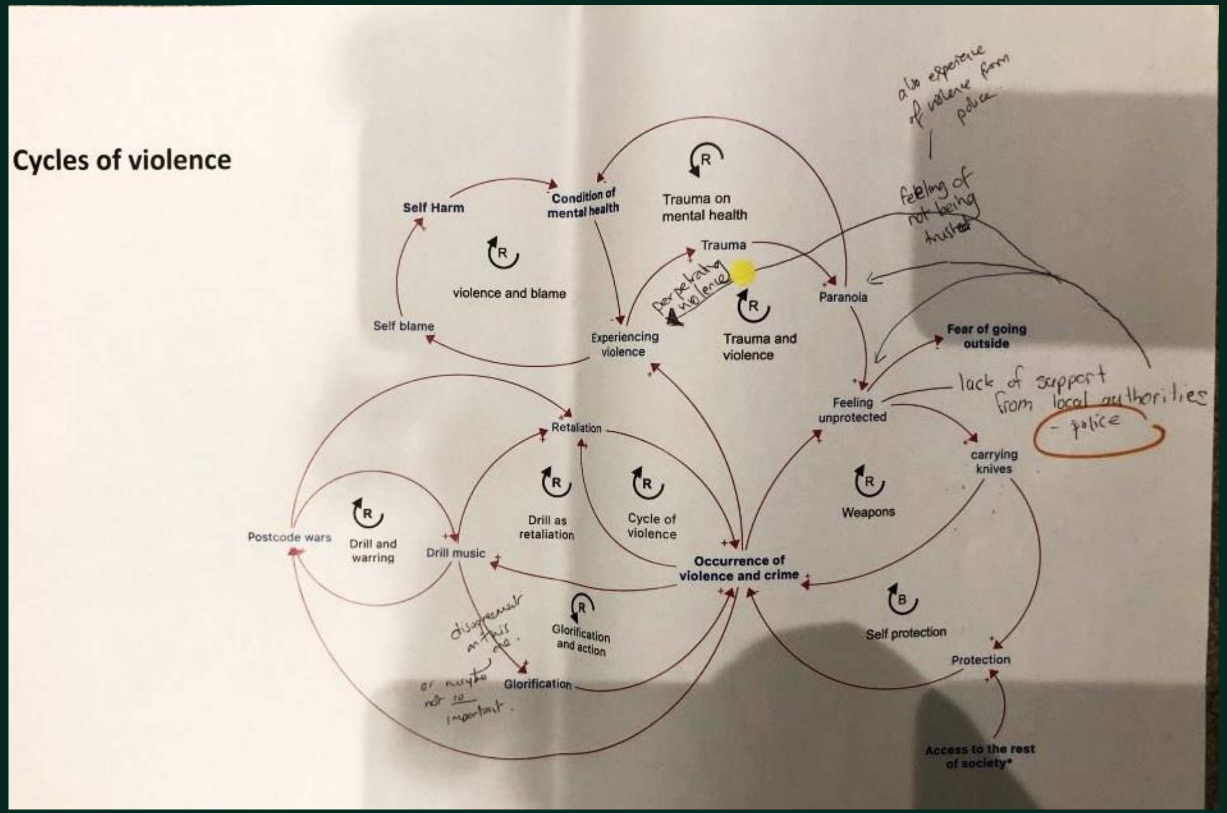

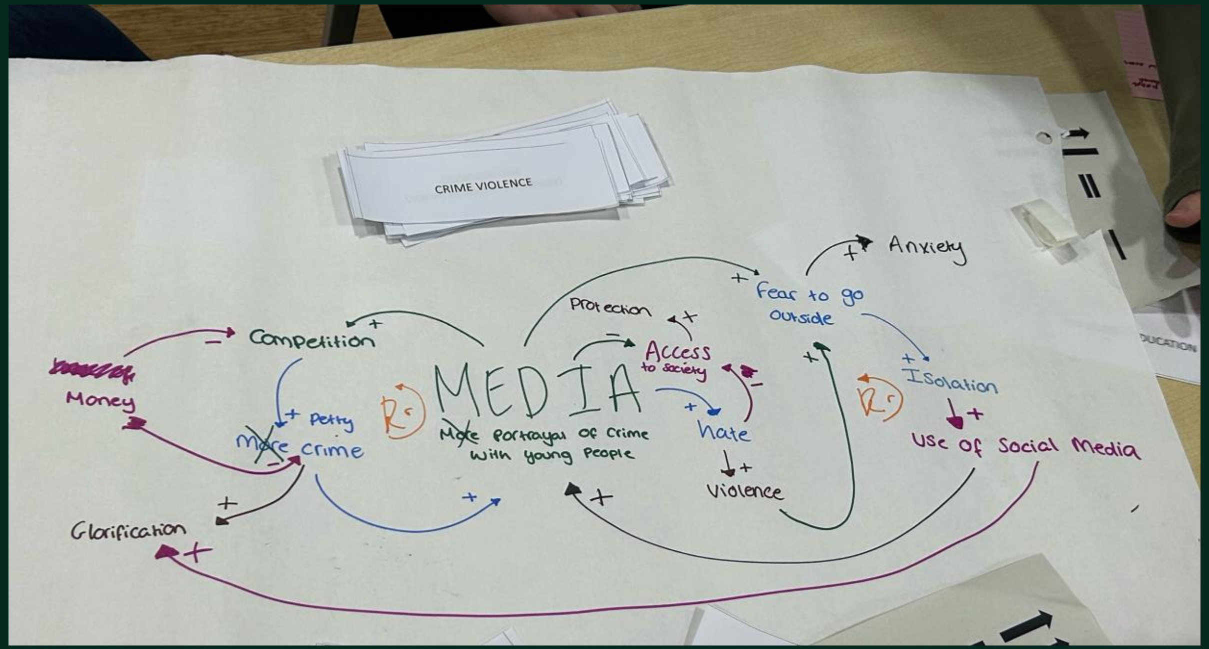

Key Dynamics of the System Map

1

Kailo

01.
Cycles of Violence

Young people in the small circles described how being impacted or involved in violence and crime can lead to retaliation and cycles of violence.

This is further implicated by drill music and the glorification of violence, which also encourages postcode wars.

Together these factors create repeating cycles of violence. However, it is not the full picture.

The wider context around some of the root drivers of violence—and their connection to young peoples' wellbeing—will be explore in the next slides.

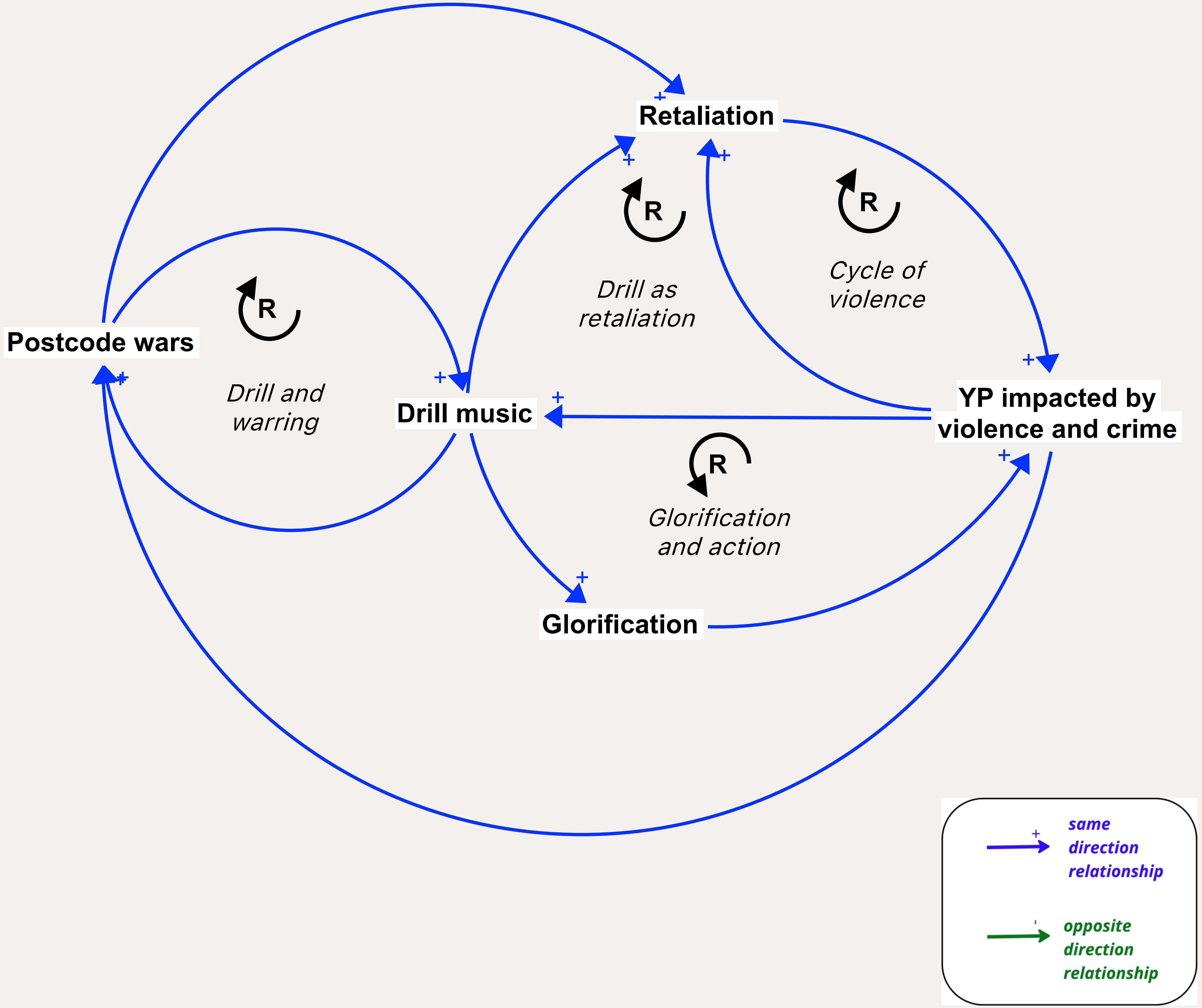

02.
Violence on Mental Health

Experiences and witnessing violence can have many negative impacts on young peoples' mental wellbeing. This includes increased self-blame, trauma, PTSD, depression, paranoia and risk of being manipulated by others. All of which contribute to a worse condition of mental health.

The relationship between these consequences of experiencing and witnessing violence also create patterns and cycles of violence, blame, and trauma. For example, if a young person experiences or witnesses violence they are at higher risk for trauma and paranoia. This may make them feel more unprotected and therefore more likely to carry a knife for protection. This could lead them to become more involved in violence and crime and thereby repeat this cycle.

Importantly, young people also shared the relationship between being involved or impacted by violence and crime and the stress associated with engaging in criminal activity. This also has a negative toll on young peoples' mental wellbeing.

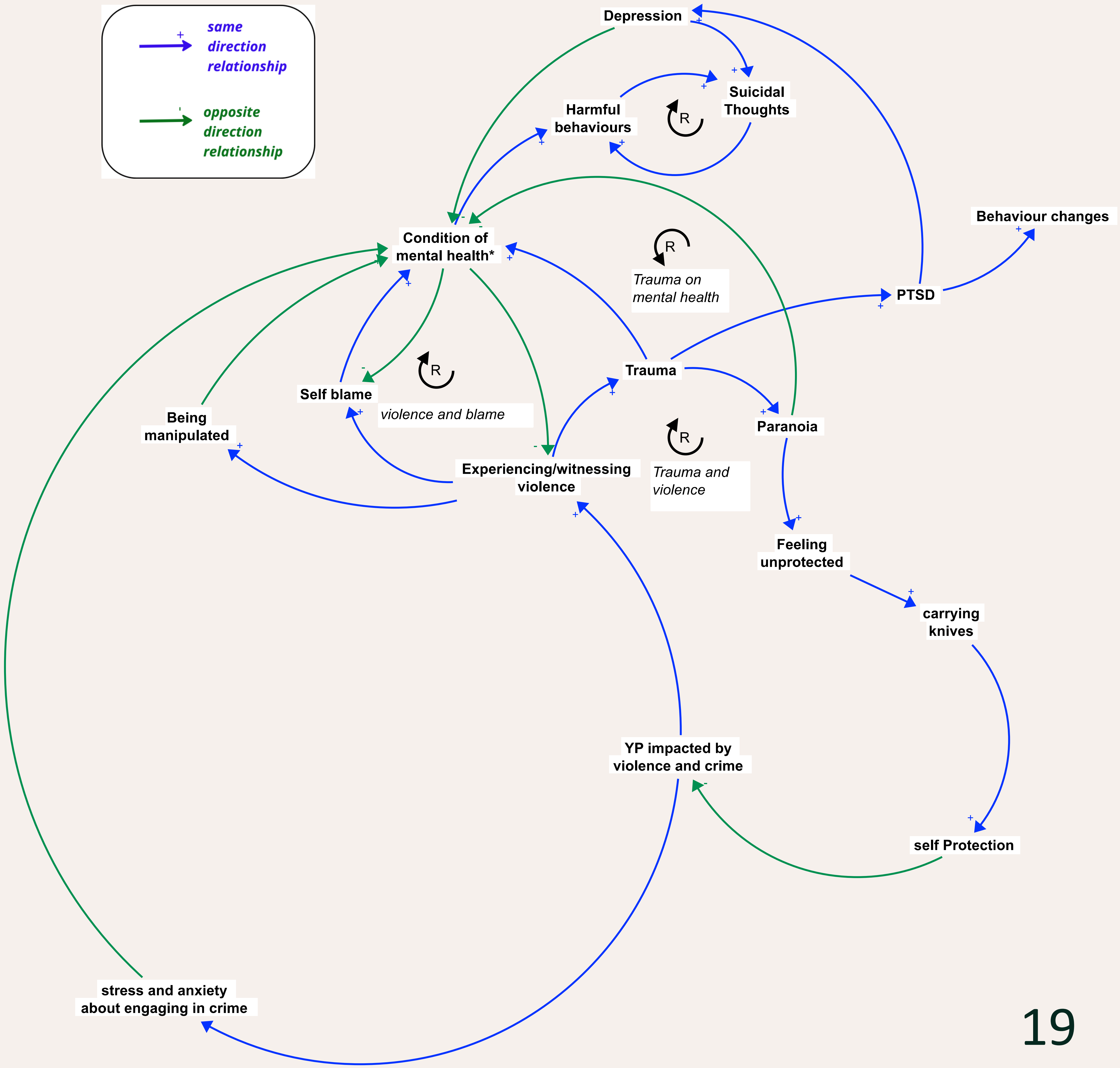

03.
Policing and Protection

Feelings of protection are also implicated by outside institutions and structures, such as policing.

Trust in police is something that young people said had a strong influence on their sense of safety and fear of violence in Newham. The less young people trust the police, the more likely they are to feel unprotected and take matters of protection into their own hands. Mistrust in the police can also lead to fear and paranoia.

Young people articulated that this sense of trust or mistrust comes from experiences of racist policing and the overuse of police powers, which relates back to power and superiority of police officers in Newham.

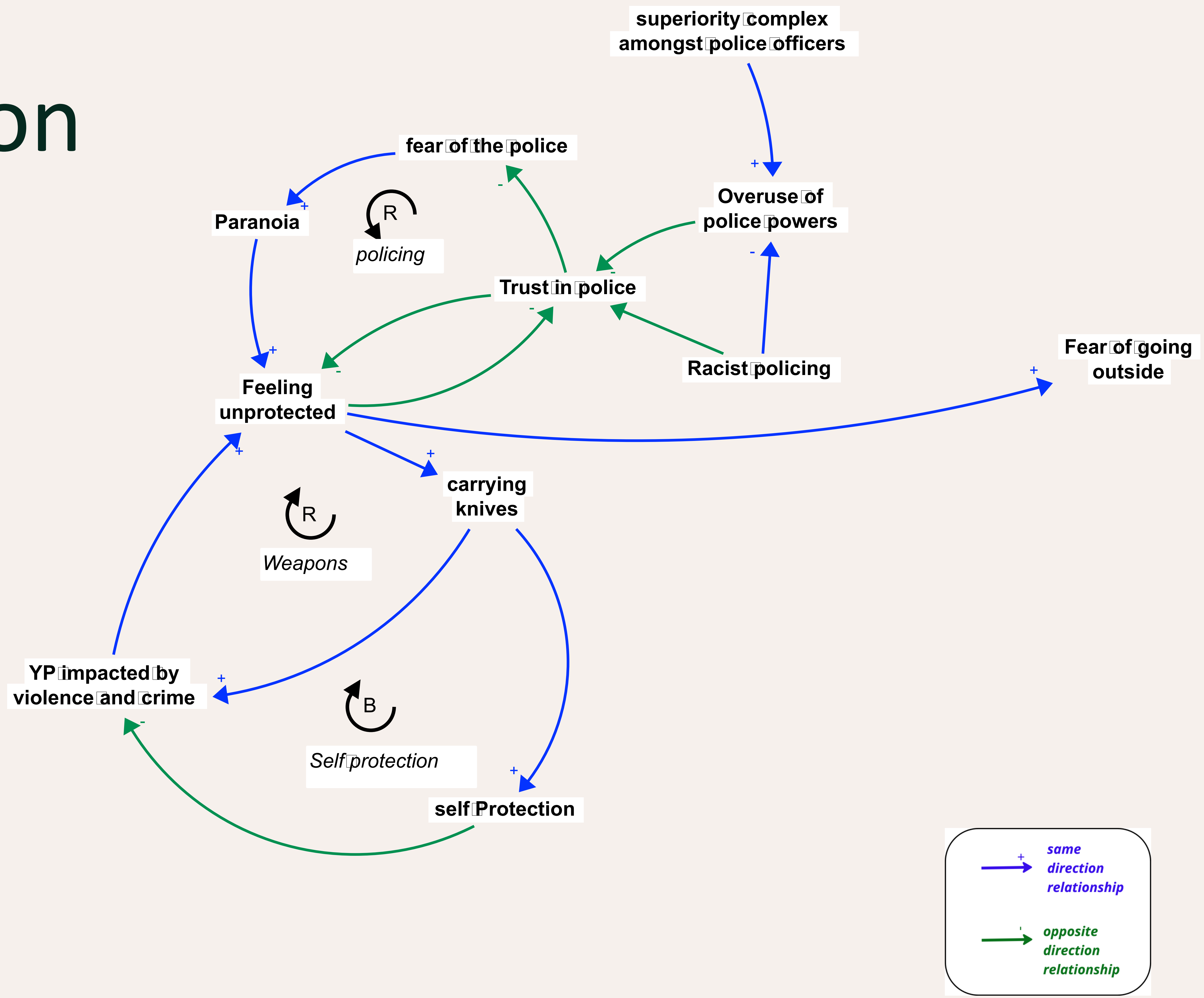

04.
Media and Perceptions of Violence

Media also has a strong role to play within the dynamics of this system modelled by young people in the small circles. The more young people fear going outside the more they tend to isolate or stay at home and experiences higher levels of loneliness. This often leads them to use more social media and witness media portrayals of crime that may add to this fear of going outside.

Young people shared how stereotypes and stigma (around this like race, age, class, etc) often shape perceptions and portrayals of violence by the media. This can have a larger impact on a community level—isolating groups of people from the "rest of society" and "outside protection". This dynamic is similar to what is known as 'spatial stigma', which perpetuates inequalities between communities.

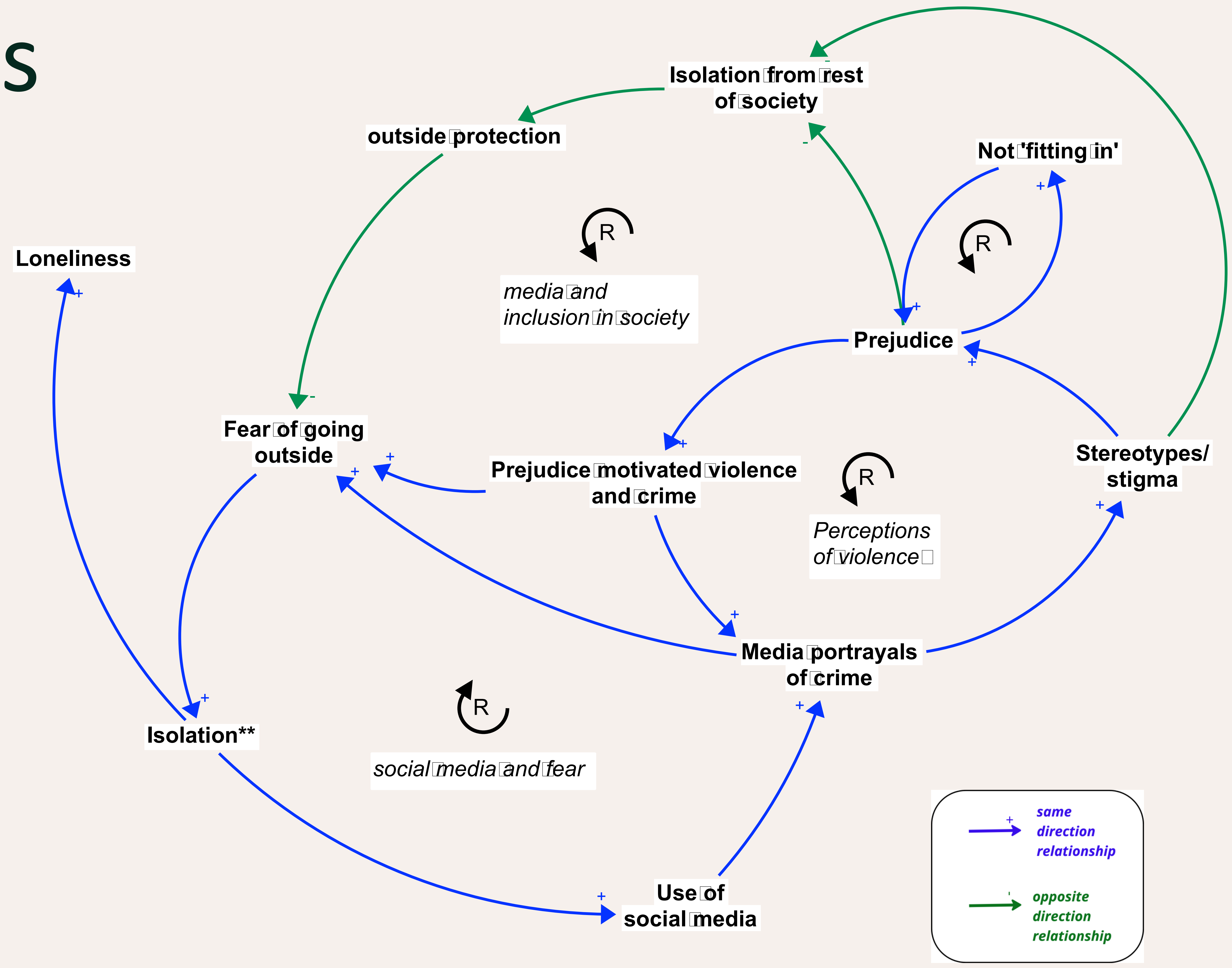

05.
Poverty and Crime

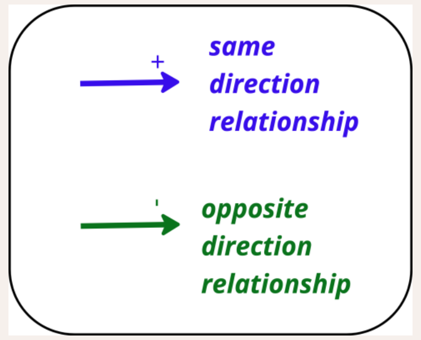

Poverty underpins many of the dynamics related to experiences and involvement in violence and crime. This dynamic is particularly prevalent for young people whose families are struggling financially.

The pressures of poverty can lead young people to seek out more 'easy money' which may involve things like dealing drugs or other non-legal ways of making money. This is done to alleviate themselves and their family from the pressure of poverty. These dynamics can be further complicated by jealousy, envy, and competition.

Young people also spoke about the high levels of gentrification in Newham and how the cost of living rising makes for less affordable housing and is putting more pressure on families in the area. This dynamic is also experienced by young people from both experiences of poverty themselves and additional pressure from their families.

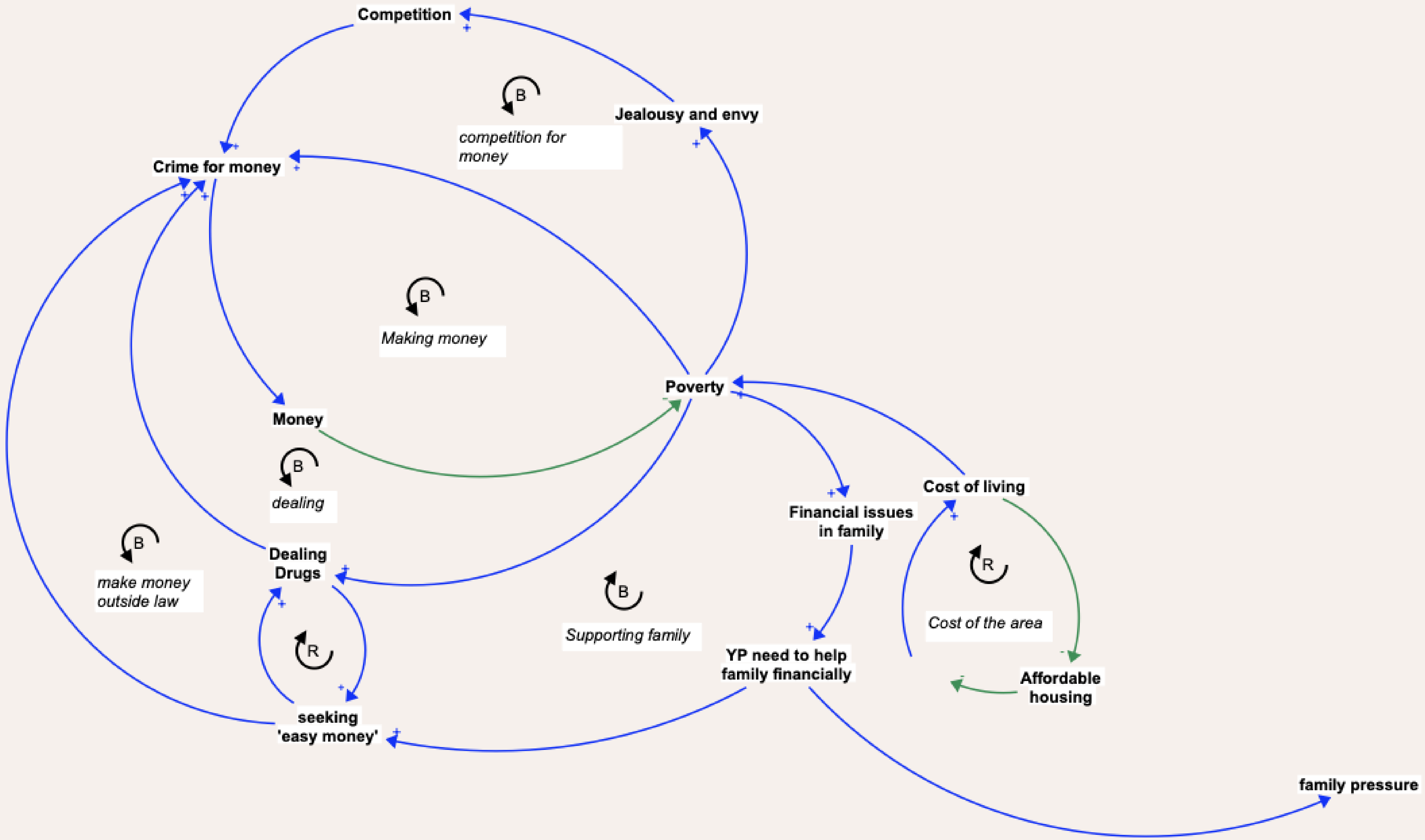

06. Family Dynamics and Support

The relationship that young people have with their families is critical to supporting their mental wellbeing. As described previously, pressures from families because of outside dynamics like poverty can lead to young people feeling as though they are not meeting their family's expectations and can cause for poor mental health and, in some cases, self-harm.

Family pressure may also lead to young people becoming socially withdrawn and less likely to reach out for mental health support or support within their family.

Young people in the small circle also described how cultural or inter-generational differences within families can make them feel more like they are not living up to their families' expectations. This can contribute to a sense of demotivation and "going down the wrong path". One way to help mitigate this experience young people shared was the availability of youth centres and outside spaces of support.

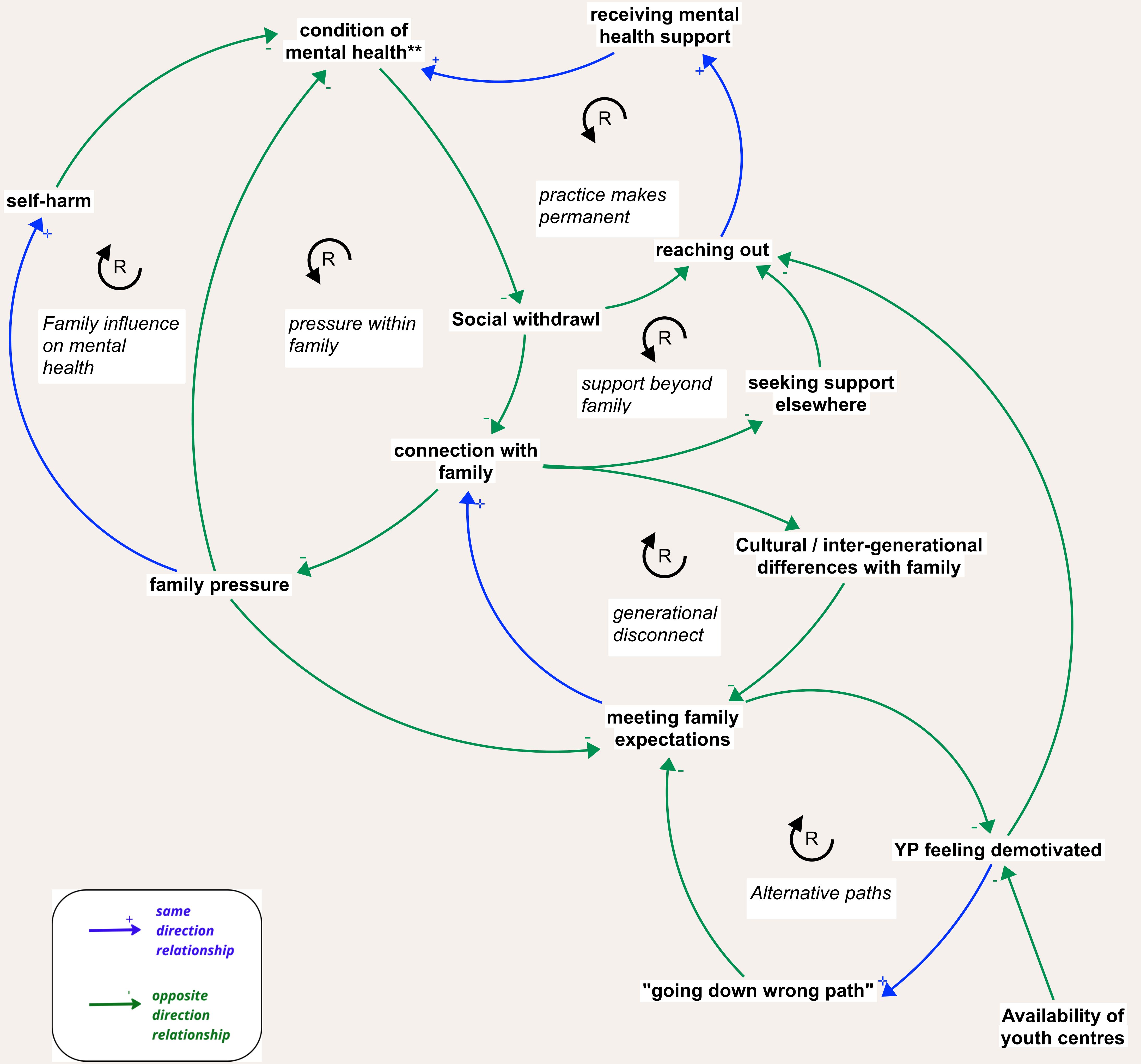

07.
Mental Health Support

Opportunities for mental health support are closely related to young peoples 'access to the rest of society'--including educational opportunities, choices and experiences from a young age, the money to be able to access resources, and psychological and physical safety.

When young people have less access to opportunities, it leads to less choices. This can lead to young people becoming more engaged in violence and crime. Engaging in violence and crime can further reduce choices and knowledge about opportunities as it can lead to young people becoming excluded from school. When these dynamics are at play, it can also reduce the quality of the environment around young people—once again leading to less choice.

When opportunities are available, however, it can lead to doors opening for young people—supporting a healthy self-perception and bettering their condition of mental health.

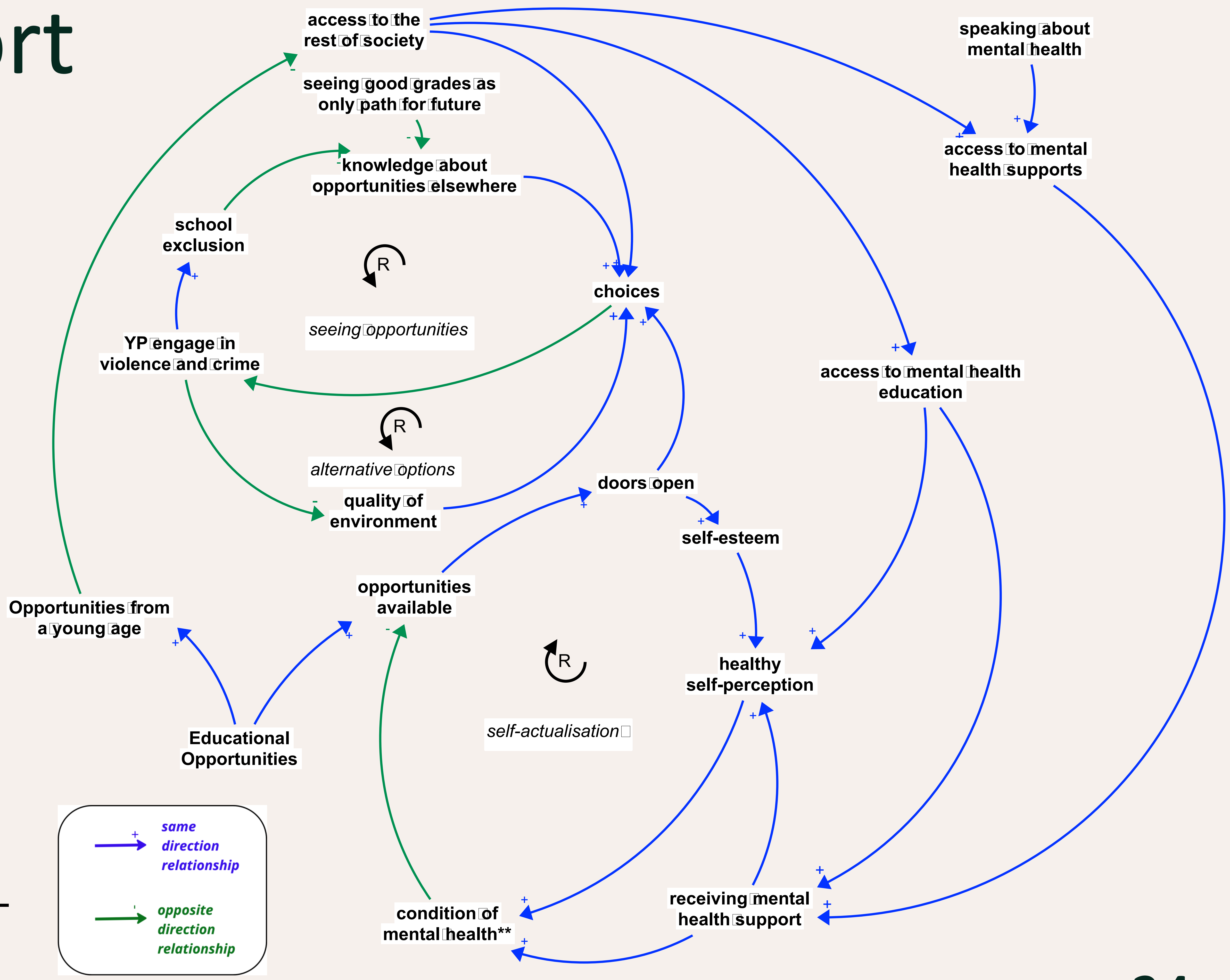

02
Key Insights

Kailo

2

Key Insights from the System Map

- Importance of **choices and access to opportunities** for young people to support their mental wellbeing.

- Pressures from **poverty** and the cost of living have a big impact on the choices that young people make—including experiences of crime and violence.

- **Perceptions** of the area and the community contribute to young peoples' experiences of safety and wellbeing. This includes perceptions from sources of outside protection, such as policing.

- **Role of family** as a source of pressure and support for young people.
